# Are alternative variables in a set differently associated with a target variable? Statistical tests and practical advice for dealing with dependent correlations

**DOI:** 10.1111/bmsp.12354

**Published:** 2024-06-24

**Authors:** Miguel A. García‐Pérez

**Affiliations:** ^1^ Departamento de Metodología, Facultad de Psicología Universidad Complutense Madrid Spain

**Keywords:** dependent correlations, Monte Carlo simulation, power, robustness, statistical tests, Type I error

## Abstract

The analysis of multiple bivariate correlations is often carried out by conducting simple tests to check whether each of them is significantly different from zero. In addition, pairwise differences are often judged by eye or by comparing the *p*‐values of the individual tests of significance despite the existence of statistical tests for differences between correlations. This paper uses simulation methods to assess the accuracy (empirical Type I error rate), power, and robustness of 10 tests designed to check the significance of the difference between two dependent correlations with overlapping variables (i.e., the correlation between *X*_1_ and *Y* and the correlation between *X*_2_ and *Y*). Five of the tests turned out to be inadvisable because their empirical Type I error rates under normality differ greatly from the nominal alpha level of .05 either across the board or within certain sub‐ranges of the parameter space. The remaining five tests were acceptable and their merits were similar in terms of all comparison criteria, although none of them was robust across all forms of non‐normality explored in the study. Practical recommendations are given for the choice of a statistical test to compare dependent correlations with overlapping variables.

## 1 INTRODUCTION

The statistical analysis of correlations among variables has not changed much over decades of psychological research, nor has it been essentially altered by an increasing concern about matching statistical methods to the intended inferences. The most common approach to dealing with large numbers of correlations (e.g., intercorrelations among all pairs of variables in a set or correlations between each variable in a set and each variable in another set) consists of reporting all of them accompanied by a description of which of them, considered in isolation, are significantly different from zero. In addition, inferences about differences in correlation are usually made by comparing the *p*‐values of the respective isolated tests of significance (for a relatively old but representative example, see Thorson & Powell, 1993). The inadequacy of this strategy for comparing correlations is easy to understand on considering that the boundary for a test that a correlation is significantly different from zero is sharp. For instance, with sample sizes of 120 observations and a two‐sided test with *α* = .05, a correlation of .180 in one group will be significantly different from zero whereas a correlation of .179 in another group will not. While these separate claims about each correlation are unquestionable, they cannot sustain a claim that the strength of correlation between the two variables of concern is different in each group, a claim that seemingly calls for a direct test that the two population correlations differ from one another. Similar considerations hold when separate tests reject the null that the correlation is zero or when they do not reject it, whether with similar or with different accompanying *p*‐values.

Empirical scenarios requiring tests of equality of two correlations may have a number of distinct characteristics. The case mentioned in the previous paragraph involves equality of correlation between the same two variables in different populations, a case usually termed the statistical comparison of independent correlations because each correlation is computed using data from a different sample of bivariate observations. A variant thereof involves equality of the correlation between two variables and the correlation between two other variables, all four of them measured in a sample from the same population. This scenario pertains to what is generally termed the statistical comparison of two dependent correlations with non‐overlapping variables, where the term captures the fact that all observations come from the same multivariate sample but none of the observations is used twice (e.g., for the computation of more than one of the correlations of concern). A somewhat different scenario also involving a single multivariate sample is referred to as the comparison of dependent correlations with overlapping variables, because now some observations are used more than once for the computation of all relevant correlations. A prototypical example involves testing whether two variables, *X*_1_ and *X*_2_, are equally correlated with a third variable *Y* (see, for example, Blake & Palmisano, 2021; Guo et al., 2022). In such cases, observations in *Y* are used for computation of its correlation with *X*_1_ and, once again, for computation of its correlation with *X*_2_. Scenarios involving a comparison of independent or dependent correlations can both involve omnibus tests of equality of more than two correlations.

The study reported in this paper focuses on statistical tests for the comparison of two dependent correlations with overlapping variables. Cases in which the comparisons of concern involve more than two correlations will be discussed in Section 6. A large number of statistical tests have been proposed for this purpose, 10 of which are presented in Section 2. In principle, all of them are interchangeable although some technicalities of their computation may affect their performance in terms of accuracy (i.e., Type I error rates), power, robustness to violations of distributional assumptions, or even the feasibility of their computation given the values of the sample statistics on hand. Earlier research on the comparative performance of these tests has been fragmentary and somewhat limited in scope. Some studies have compared a subset of these tests according to their accuracy and power with data drawn from trivariate normal distributions, although none of the studies has considered a broad range of values for the population correlations and a broad range of sample sizes (see, for example, Boyer et al., 1983; Cohen, 1989; Dunn & Clark, 1971; Hittner et al., 2003; May & Hittner, 1997a; Neill & Dunn, 1975; Wilcox & Tian, 2008). In addition, some studies have reported fractional results on robustness under violation of trivariate normality, always using identical non‐normal (univariate) distributions for *X*_1_, *X*_2_, and *Y*. For instance, Boyer et al. (1983) used lognormal distributions, Cohen (1989) used chi‐square distributions with two and four degrees of freedom, and May and Hittner (1997a); see also (Hittner et al., 2003; Wilcox & Tian, 2008) used uniform and exponential distributions. Results reported in the studies just mentioned have shown a relatively small deterioration in the accuracy and power of the tests under violation of normality, but the generalizability of this conclusion to the empirically plausible case of arbitrarily different non‐normal univariate distributions for *X*_1_, *X*_2_, and *Y* is uncertain. A further aspect that has not previously been studied is the comparative applicability of the tests, all of which involve computations that may render the test statistic undefined for certain combinations of the values of sample statistics.

The motivation for conducting a comprehensive comparative study of accuracy, power, robustness, and applicability of alternative tests for equality of two dependent correlations is twofold. A first component comes from the foreseeable replacement of current malpractice (i.e., conducting isolated tests of significance of each of the implied correlations and comparing their outcomes) with the use of proper tests of significance of the difference between dependent correlations. If this is about to come, the choice of statistical test for this purpose must be based on evidence of their comparative performance. The second component comes from the availability of computer programs and web applications that carry out and report the outcomes of a number of alternative tests with no accompanying information regarding which test is more dependable than which other, thus leaving users uncertain about which result should be pulled out from the list (see, for example, Diedenhofen & Musch, 2015; Silver et al., 2006; Silver & Merino‐Soto, 2016). To investigate the usage of the various tests considered in this paper, on 22 December 2023, we used Google Scholar to find papers published in 2023 in journals containing ‘psychology’ or ‘psychological’ in their title and that cited *cocor* (Diedenhofen & Musch, 2015), an R package (https://cran.r‐project.org/web/packages/cocor) and web application (http://comparingcorrelations.org) to conduct tests of equality of dependent correlations with overlapping variables among other comparisons of correlations. The search retrieved 52 papers, but nearly half of them reported using *cocor* to conduct tests of equality of independent correlations or equality of dependent correlations with non‐overlapping variables, whereas others cited the target paper for reasons not related to actual usage of the tests. Twenty‐seven papers remained that reported using *cocor* for comparing dependent correlations with overlapping variables. Table A1 in Appendix A identifies each of these papers and indicates which test was reportedly used. Ten of the papers did not even report which test had been used, one other reported that all tests gave the same results, and the remaining 16 papers varied in their choice of test.

The plan of this paper is as follows. Section 2 describes the 10 tests whose performance will be compared. Section 3 describes the criteria for comparison, which cover the four aspects mentioned above, namely, Type I error rates, power, robustness, and empirical applicability. Section 4 describes the simulation method used to achieve these goals, including a description of the extensive set of scenarios (population correlations, sample sizes, and univariate distributions) under which the performance of the tests is compared. Section 5 reports the results in terms of each of the comparison criteria. Section 6 presents some empirical examples with data from recent papers in which dependent correlations were conventionally compared (i.e., via the *p*‐value of their respective tests of significance against zero). Finally, Section 7 summarizes and discusses the results, also offering practical recommendations regarding the optimal choice of statistical test.

## 2 GENERAL NOTATION AND TEST STATISTICS

Let *X*_1_ and *X*_2_ be the two variables whose individual correlations with variable *Y* are to be compared. We initially assume that all variables have standard normal distributions and a trivariate normal distribution with covariance and correlation matrix

$$Ρ=\left(\begin{array}{ccc} 1 & \rho_{X_{1}X_{2}} & \rho_{X_{1}Y}\\ \rho_{X_{1}X_{2}} & 1 & \rho_{X_{2}Y}\\ \rho_{X_{1}Y} & \rho_{X_{2}Y} & 1 \end{array}\right).$$

We will simplify notation via $\rho_{1Y}\;\;:\;=\rho_{X_{1}Y}$, $\rho_{2Y}\;\;:\;=\rho_{X_{2}Y}$, and $\rho_{12}\;\;:\;=\rho_{X_{1}X_{2}}$. This section describes briefly a number of methods that have been proposed to test the null hypothesis H_0_: *ρ*_1*Y*_ = *ρ*_2*Y*_ using *n* triplets of observations from which sample correlations *r*_1*Y*_, *r*_2*Y*_, and *r*_12_ are computed. We name the tests as in Diedenhofen and Musch (2015), where a full presentation is given. Computation of all test statistics involves some square root whose radicand is determined by sample correlations and, thus, it is not guaranteed to be positive‐valued. Whenever the radicand turns up negative for the sample of concern, the test statistic is undefined.

### 2.1 Pearson–Filon

This statistic (Pearson & Filon, 1898) has a standard normal distribution and is computed as

(1) $$z_{\mathrm{PF}}=\frac{\left(r_{1Y}-r_{2Y}\right)\sqrt{n}\;}{\sqrt{{\left(1-r_{1Y}^{2}\right)}^{2}+{\left(1-r_{2Y}^{2}\right)}^{2}-2r_{12}\left(1-r_{1Y}^{2}-r_{2Y}^{2}\right)+\left(r_{1Y}r_{2Y}\right)\left(1-r_{1Y}^{2}-r_{2Y}^{2}-r_{12}^{2}\right)}}.$$

### 2.2 Olkin

This statistic (Olkin, 1967; with the correct formula in Hendrickson & Collins, 1970) has a standard normal distribution and is computed as

(2) $$z_{O}=\frac{\left(r_{1Y}-r_{2Y}\right)\sqrt{n}\;}{\sqrt{{\left(1-r_{1Y}^{2}\right)}^{2}+{\left(1-r_{2Y}^{2}\right)}^{2}-2r_{12}^{3}-\left(2r_{12}-r_{1Y}r_{2Y}\right)\left(1-r_{1Y}^{2}-r_{2Y}^{2}-r_{12}^{2}\right)}}.$$

### 2.3 Hotelling

This statistic (Hotelling, 1940) has a Student's *t* distribution with *n* − 3 degrees of freedom and is computed as

(3) $$t_{H}=\frac{\left(r_{1Y}-r_{2Y}\right)\sqrt{\left(n-3\right)\left(1+r_{12}\right)}\;}{\sqrt{2\left|R\right|}}$$

with

(4) $$\left|R\right|=1-r_{1Y}^{2}-r_{2Y}^{2}-r_{12}^{2}+2r_{1Y}r_{2Y}r_{12}.$$

### 2.4 Standard Williams

This statistic (Williams, 1959) has a Student's *t* distribution with *n* − 3 degrees of freedom and is computed as

(5) $$t_{W}=\frac{\left(r_{1Y}-r_{2Y}\right)\sqrt{\left(n-3\right)\left(1+r_{12}\right)}\;}{\sqrt{2\left|R\right|+{\bar{r}}^{2}\frac{n-3}{n-1}{\left(1-r_{12}\right)}^{3}}}$$

with $\left|R\right|$ from Equation (4) and

(6) $$\bar{r}=\frac{r_{1Y}+r_{2Y}}{2}.$$

### 2.5 Hendrickson–Stanley–Hills

This statistic (Hendrickson et al., 1970) has a Student's *t* distribution with *n* − 3 degrees of freedom and is computed as

(7) $$t_{\mathrm{HSH}}=\frac{\left(r_{1Y}-r_{2Y}\right)\sqrt{\left(n-3\right)\left(1+r_{12}\right)}\;}{\sqrt{2\left|R\right|+\frac{{\left(r_{1Y}-r_{2Y}\right)}^{2}{\left(1-r_{12}\right)}^{3}}{4\left(n-1\right)}}}$$

with $\left|R\right|$ from Equation (4).

### 2.6 Dunn–Clark

This statistic (Dunn & Clark, 1969) has a standard normal distribution and is computed as

(8) $$z_{\mathrm{DC}}=\frac{\left(Z_{1Y}-Z_{2Y}\right)\sqrt{n-3}\;}{\sqrt{2-2c_{\mathrm{DC}}}}$$

with *Z*_1*Y*_ and *Z*_2*Y*_ given by Fisher's transformation by which

$$Z_{\mathrm{xy}}=\frac{1}{2}\ln\left(\frac{1+r_{\mathrm{xy}}}{1-r_{\mathrm{xy}}}\right)$$

and

(9) $$c_{\mathrm{DC}}=\frac{r_{12}\left(1-r_{1Y}^{2}-r_{2Y}^{2}\right)-\frac{1}{2}r_{1Y}r_{2Y}\left(1-r_{1Y}^{2}-r_{2Y}^{2}-r_{12}^{2}\right)}{\left(1-r_{1Y}^{2}\right)\left(1-r_{2Y}^{2}\right)}.$$

### 2.7 Steiger

This statistic (Steiger, 1980) has a standard normal distribution and is computed as

(10) $$z_{S}=\frac{\left(Z_{1Y}-Z_{2Y}\right)\sqrt{n-3}\;}{\sqrt{2-2c_{S}}}$$

with *Z*_1*Y*_ and *Z*_2*Y*_ given by Fisher's transformation and

(11) $$c_{S}=\frac{r_{12}\left(1-2{\bar{r}}^{2}\right)-\frac{1}{2}{\bar{r}}^{2}\left(1-2{\bar{r}}^{2}-r_{12}^{2}\right)}{{\left(1-{\bar{r}}^{2}\right)}^{2}},$$

with $\bar{r}$ from Equation (6).

### 2.8 Hittner–May–Silver

This statistic (Hittner et al., 2003) has a standard normal distribution and is computed as

(12) $$z_{\mathrm{HMS}}=\frac{\left(Z_{1Y}-Z_{2Y}\right)\sqrt{n-3}\;}{\sqrt{2-2c_{\mathrm{HMS}}}}$$

with *Z*_1*Y*_ and *Z*_2*Y*_ given by Fisher's transformation and

(13) $$c_{\mathrm{HMS}}=\frac{r_{12}\left(1-2{\bar{r}}_{z}^{2}\right)-\frac{1}{2}{\bar{r}}_{z}^{2}\left(1-2{\bar{r}}_{z}^{2}-r_{12}^{2}\right)}{{\left(1-{\bar{r}}_{z}^{2}\right)}^{2}},$$

in which

$${\bar{r}}_{z}=\frac{\exp\left(2\bar{Z}-1\right)}{\exp\left(2\bar{Z}+1\right)}\;\text{and}\;\bar{Z}=\frac{Z_{1Y}+Z_{2Y}}{2}.$$

### 2.9 Meng–Rosenthal–Rubin

This statistic (Meng et al., 1992) has a standard normal distribution and is computed as

(14) $$z_{\mathrm{MRR}}=\frac{\left(Z_{1Y}-Z_{2Y}\right)\sqrt{n-3}\;}{\sqrt{2\left(1-r_{12}\right)h}}$$

with *Z*_1*Y*_ and *Z*_2*Y*_ given by Fisher's transformation and

(15) $$h=\frac{1-f\bar{r^{2}}}{1-\bar{r^{2}}}$$

with

$$\bar{r^{2}}=\frac{r_{1Y}^{2}+r_{2Y}^{2}}{2}\;\text{and}\;f=\frac{1-r_{12}}{2\left(1-\bar{r^{2}}\right)}$$

Meng et al. (1992) stated that *f* = 1 should be used whenever the computation of *f* using sample statistics results in *f* > 1, but the justification for this replacement is unclear. At first glance, the only apparent requirement is that the radicand in Equation (14) is positive‐valued. This implies *h* > 0 or, by Equation (15), $f<1/\bar{r^{2}}$, which can sometimes result in *f* > 1. This study will use this test without replacing any *f* > 1 with *f* = 1, with the consequence that the test statistic is undefined when sample statistics result in $f\geq 1/\bar{r^{2}}$.

### 2.10 Zou

This test (Zou, 2007) is based on a confidence interval. The lower (*L*) and upper (*U*) limits of a 100 (1 − *α*)% confidence interval for *ρ*_1*Y*_ − *ρ*_2*Y*_ are

(16) $$L=r_{1Y}-r_{2Y}-\sqrt{{\left(r_{1Y}-l_{1}\right)}^{2}+{\left(u_{2}-r_{2Y}\right)}^{2}-2c\left(r_{1Y}-l_{1}\right)\left(u_{2}-r_{2Y}\right)},$$

(17) $$U=r_{1Y}-r_{2Y}+\sqrt{{\left(u_{1}-r_{1Y}\right)}^{2}+{\left(r_{2Y}-l_{2}\right)}^{2}-2c_{Z}\left(u_{1}-r_{1Y}\right)\left(r_{2Y}-l_{2}\right)},$$

with

(18) $$l_{i}=\frac{\exp\left(2l_{i}^{*}\right)-1}{\exp\left(2l_{i}^{*}\right)+1},\;i\in \left\{1,2\right\},$$

(19) $$u_{i}=\frac{\exp\left(2u_{i}^{*}\right)-1}{\exp\left(2u_{i}^{*}\right)+1},\;i\in \left\{1,2\right\},$$

(20) $$l_{i}^{*}=Z_{\mathrm{iY}}-\frac{z_{1-\alpha /2}}{\sqrt{n-3}},\;i\in \left\{1,2\right\},$$

(21) $$u_{i}^{*}=Z_{\mathrm{iY}}+\frac{z_{1-\alpha /2}}{\sqrt{n-3}},\;i\in \left\{1,2\right\},$$

where *Z*_*iY*_ is given by Fisher's transformation of *r*_*iY*_, *z*_1−*α*/2_ is the 100(1 − *α*/2)% standard normal quantile, and

(22) $$c_{Z}=\frac{\left(r_{12}-\frac{1}{2}r_{1Y}r_{2Y}\right)\left(1-r_{1Y}^{2}-r_{2Y}^{2}-r_{12}^{2}\right)+r_{12}^{3}}{\left(1-r_{1Y}^{2}\right)\left(1-r_{2Y}^{2}\right)}.$$

Given that *L* < *U* by construction, application of this method rejects the null hypothesis of equality of correlations whenever *L* > 0 or *U* < 0.

## 3 CRITERIA FOR COMPARISON

**FIGURE 1:**
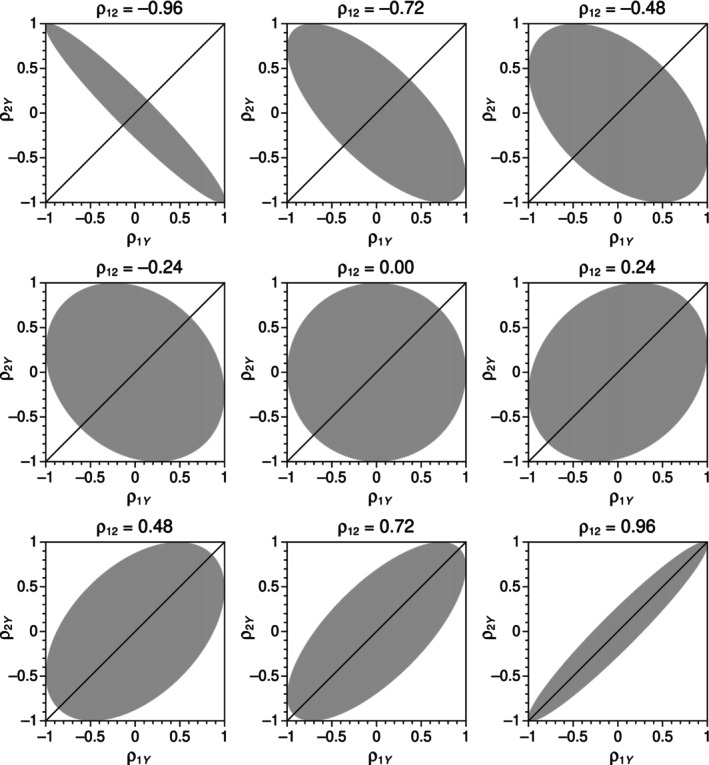
Region (shaded area) where pairs of values for *ρ*_1*Y*_ and *ρ*_2*Y*_ may exist in a trivariate normal distribution, as a function of the value taken on by *ρ*_12_ (panels). The diagonal in each panel is the identity line *ρ*_1*Y*_ = *ρ*_2*Y*_.

Before presenting the criteria for comparison of alternative tests for H_0_: *ρ*_1*Y*_ = *ρ*_2*Y*_, it should be noted that the identity stated in the null can only hold for a restricted range of values determined by the population correlation *ρ*_12_. The reason is that a non‐degenerate trivariate normal distribution can only exist if $\left|Ρ\right|=1-\rho_{1Y}^{2}-\rho_{2Y}^{2}-\rho_{12}^{2}+2\rho_{1Y}\rho_{2Y}\rho_{12}>0$. Algebraic manipulation of this inequality indicates that the possible range for *ρ*_2*Y*_ given arbitrary values for *ρ*_12_ and *ρ*_1*Y*_ is

(23) $$\rho_{12}\rho_{1Y}-\sqrt{\left(1-\rho_{12}^{2}\right)\left(1-\rho_{1Y}^{2}\right)}<\rho_{2Y}<\rho_{12}\rho_{1Y}+\sqrt{\left(1-\rho_{12}^{2}\right)\left(1-\rho_{1Y}^{2}\right)};$$

note that the range is not empty for any pair of values for *ρ*_12_ and *ρ*_1*Y*_. The shaded areas in Figure 1 show cross‐sectional slices of the region of feasibility of *ρ*_1*Y*_ and *ρ*_2*Y*_ at selected values for *ρ*_12_. The intersection of these regions with the diagonal identity line describes the range of values for which a trivariate normal distribution may exist where H_0_ is true. A comparison of the accuracy of tests is thus restricted to feasible true nulls (i.e., *ρ*_1*Y*_ = *ρ*_2*Y*_) for each value of *ρ*_12_, whereas a comparison of their power is restricted to feasible false nulls (i.e., *ρ*_1*Y*_ ≠ *ρ*_2*Y*_) for each value of *ρ*_12_.

The first criterion for comparison is the region in the three‐dimensional space of sample correlations *r*_1*Y*_, *r*_2*Y*_, and *r*_12_ for which a test statistic is real‐valued. Although the region of existence of a trivariate normal distribution in the analogous three‐dimensional space of population correlations is well defined and illustrated in Figure 1, it is not clear that all test statistics can accommodate any sample correlations in this region. Consider the Hotelling test statistic in Equation (3), where the radicand (Equation (4)) simply sets on sample correlations the same condition set on population correlations for the existence of a trivariate normal distribution, thus ensuring coverage of the sample space of trivariate correlations. In contrast, the standard Williams statistic in Equation (5) and the Hendrickson–Stanley–Hills statistic in Equation (7) both incorporate into the radicand additive and positive‐valued terms that broaden the applicability of these statistics. It is not immediately obvious how the relatively more complex radicands in the remaining test statistics affect their corresponding regions of applicability.

The second and third criteria for comparison involve the accuracy of the tests (i.e., the match between nominal and actual Type I error rates) and their power, both assessed when observations are drawn from trivariate normal distributions and from non‐normal distributions. Comparisons involving these criteria were carried out via simulations described in the next section.

## 4 SIMULATION METHOD

Empirical Type I error rates were determined for each test for values of *ρ*_12_ between −.9 and .9 in steps of .15 and, in each case, within the admissible range of values for *ρ*_1*Y*_ = *ρ*_2*Y*_ in steps of .02 (i.e., along the diagonal identity line in the panels of Figure 1 within the region indicated by the shaded area). For each combination of *ρ*_12_ and *ρ*_1*Y*_ = *ρ*_2*Y*_, we conducted *N* = 500,000 replications each consisting of *n* triplets of standard normal observations *X*_1_, *X*_2_, and *Y* drawn from the corresponding trivariate normal distribution. In separate simulation runs, sample sizes *n* were 20, 50, 100, and 200. The 10 test statistics were computed for each sample in each replication, and the empirical Type I error rate for each test was computed as the proportion of replications (out of *N*) in which the null was rejected in a two‐sided test at *α* = .05, keeping separate counts of rejection in each tail.

**FIGURE 2:**
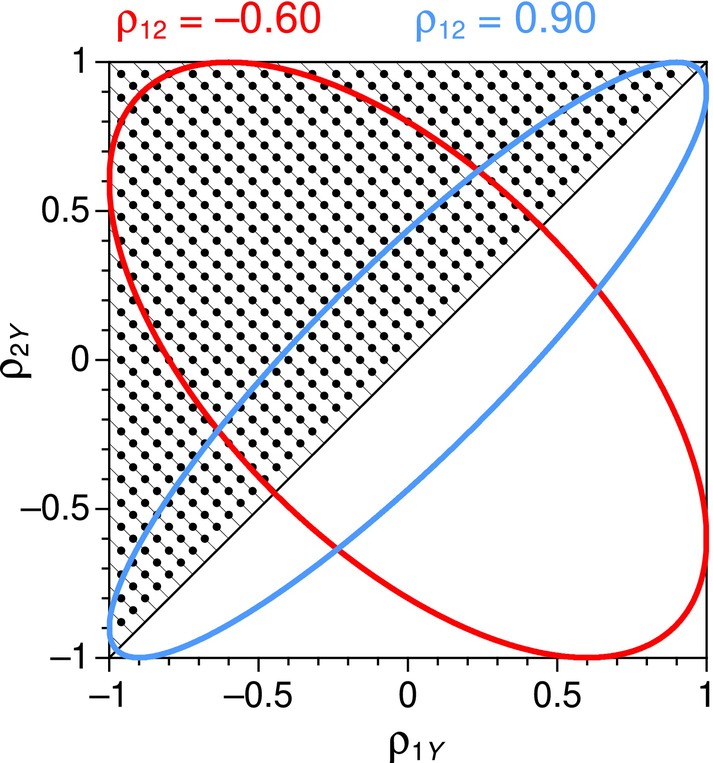
Lattice points (small circles) for the analysis of power. For each value of *ρ*_12_, only lattice points are used that fall within the region of existence, as illustrated by the red and blue ellipses (Colour is available online).

Power was determined for each test in a similar manner with 200,000 replications per condition and for the same set of values of *ρ*_12_, drawing triplets from trivariate normal distributions with *ρ*_1*Y*_ ≠ *ρ*_2*Y*_ in pairings described by the lattice points in Figure 2. Lattice points are located along lines perpendicular to the identity line and with respect to reference points on the diagonal ranging from *ρ*_1*Y*_ = *ρ*_2*Y*_ = −.96 to *ρ*_1*Y*_ = *ρ*_2*Y*_ = .96 in steps of .04. Successive lattice points along off‐diagonal directions are created by progressively subtracting .04 from the reference value of *ρ*_1*Y*_ and adding .04 to the reference value of *ρ*_2*Y*_. Power at a given lattice point was determined only if a trivariate normal distribution exists under the applicable value of *ρ*_12_, for example, for the lattice points within the region of existence illustrated in Figure 2 for *ρ*_12_ = −.60 (red ellipse) or for *ρ*_12_ = .90 (blue ellipse). No lattice points were defined below the diagonal in Figure 2 because of the symmetry of the tests with respect to the sign of the difference between *ρ*_1*Y*_ and *ρ*_2*Y*_. The off‐diagonal lines on which lattice points are placed allow assessment of whether power varies as a simple function of the absolute value of the difference *ρ*_1*Y*_ − *ρ*_2*Y*_ (which is constant for all lattice points falling on the same imaginary line parallel to the identity line) or differs also with the point on the diagonal relative to which |*ρ*_1*Y*_ − *ρ*_2*Y*_| is placed, that is, with the location of the intersection of the off‐diagonal line and the identity line. The location of this intersection is the point at which both *ρ*_1*Y*_ and *ρ*_2*Y*_ equal their average (*ρ*_1*Y*_ + *ρ*_2*Y*_)/2. In other words, different off‐diagonal lines define lattice points of increasing value of |*ρ*_1*Y*_ − *ρ*_2*Y*_| at fixed values of (*ρ*_1*Y*_ + *ρ*_2*Y*_)/2.

Triplets of observations drawn from non‐normal distributions were used to assess accuracy and power under violation of the assumption of trivariate normality. Many forms of non‐normality have been described in psychological data at both the univariate and multivariate levels (Cain et al., 2017). Hence, a thorough assessment of robustness to arbitrary violations of trivariate normality is impractical. Our goal here is thus limited to describing results for a number of plausible cases that nevertheless shed light on the robustness (or lack thereof) of the tests under analysis. This was done in three different ways. For the first, data from beta distributions with *a* = 1 and *b* = 1 (i.e., uniform) or *a* = 2 and *b* = 5 (positively skewed) were generated by first drawing triplets of observations in *Z*_1_, *Z*_2_, and *Z*_*y*_ from a trivariate standard normal distribution with correlation matrix **Ρ**. Sample values were then transformed via $X_{1}=F^{-1}\left(\Phi \left(Z_{1}\right);a_{1},b_{1}\right)$, $X_{2}=F^{-1}\left(\Phi \left(Z_{2}\right);a_{2},b_{2}\right)$, and $Y=F^{-1}\left(\Phi \left(Z_{y}\right);a_{y},b_{y}\right)$, where Φ is the standard normal distribution function and *F* ^−1^ is the beta quantile function with parameters *a* and *b*. Note that beta parameters are defined separately for each variable, thus allowing any combination of forms for the univariate distributions of *X*_1_, *X*_2_, and *Y*. For the second way, triplets *Z*_1_, *Z*_2_, and *Z*_*y*_ generated as described above were transformed via *X*_1_ = exp(*Z*_1_), *X*_2_ = exp(*Z*_2_) and *Y* = exp(*Z*_*y*_), which produces data with univariate Lognormal(0, 1) distributions. Finally, normal mixtures were generated whose first component (with probability .9) was the trivariate standard normal distribution with correlation matrix **Ρ** and whose second component (with probability .1) was a trivariate normal distribution with the same correlation matrix but larger variances (2, 4, and 10) for all variables.

Generation of non‐normal variables via transformation (or mixture) of normal variables ensured that the former matched the target correlations *ρ*_12_, *ρ*_1*Y*_, and *ρ*_2*Y*_ included in analogous simulations with normally distributed data. Specifically, for our mixtures of normal variables with the same correlation matrix and differing only in a common variance, the correlation matrix of the mixture is that of the original variables and, then, the components were generated with the applicable target correlations. When standard normal variables *X* and *Y* with correlation *ρ*_*xy*_ are exponentially transformed into lognormal variables $\tilde{X}$ and $\tilde{Y}$, the correlation between the latter is given by

(24) $$\rho_{\tilde{x}\tilde{y}}=\frac{\exp\left(\rho_{\mathrm{xy}}\right)-1}{\exp\left(1\right)-1}$$

(De Veaux, 1976). Then the correlation matrix for the originating normal variables was set by inverting this relation so that the resulting lognormal variables had the required target correlations. This, in turn, reduces the breadth of target correlations that can be explored because the lowest possible correlation between lognormal variables is –exp(−1) ≈ −.3679 (De Veaux, 1976). Finally, beta variables generated via the inverse transform method do not generally keep the correlation of the originating normal variables and there is no closed‐form expression to compensate for this fact in the correlation matrix of the generating normal variables. Yet, Appendix B shows that the original correlations are almost exactly preserved with the beta parameters of our choice. The skewness and kurtosis of each of the resulting non‐normal univariate distributions are listed in Table 1; note that heteroscedasticity generally holds for bivariate distributions with non‐normal marginals.

**TABLE 1:** Skewness and kurtosis of the non‐normal univariate distributions used in the simulations.

Distribution	Skewness	Kurtosis
Uniform	0.000	1.800
Beta (2,5)	0.596	2.880
Lognormal (0,1)	6.149	113.936
Mixture 0.9 N(0,1) + 0.1 N(0,2)	0.000	3.223
Mixture 0.9 N(0,1) + 0.1 N(0,4)	0.000	4.438
Mixture 0.9 N(0,1) + 0.1 N(0,10)	0.000	9.059

The study includes cases in which all variables had the same non‐normal univariate distribution and a number of other cases in which form of distribution varied across variables while keeping the same distribution for *X*_1_ and *X*_2_ to ensure that the null hypothesis remains true or false as needed. In all cases, data were generated under all of the applicable conditions described earlier for similar analyses under trivariate normality, except that we used *N* = 250,000 replications in studies of accuracy and *N* = 100,000 replications in studies of power.

Because of the extensive and time‐consuming computations required for completion of the study, Fortran code was written to carry out all the simulations.

## 5 RESULTS

### 5.1 Region of empirical applicability

**FIGURE 3:**
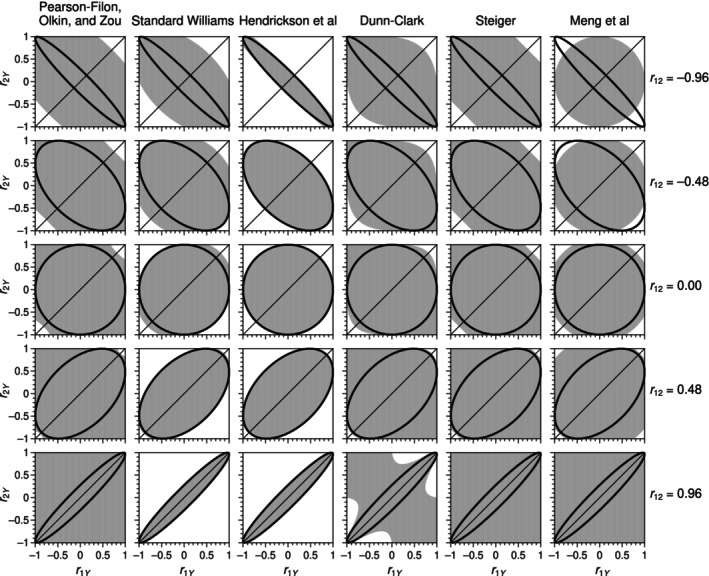
Region of applicability (shaded area) of tests (columns) at several sample values for *r*_12_ (rows). The elliptical region of existence of a trivariate normal distribution is also shown in each panel for reference.

Obtaining the region of empirical applicability amounts to finding out which triplets (*r*_1*Y*_, *r*_2*Y*_, *r*_12_) on the cube [−1, 1] × [−1, 1] × [−1, 1] result in a positive‐valued radicand on computation of each test statistic. A minimum requirement is that the statistic is real‐valued when sample correlations take any values compatible with a trivariate normal distribution, and we have already discussed that the Hotelling statistic in Equation (3) has this exact characteristic. The standard Williams and Hendrickson–Stanley–Hills statistics (both with *n* = 100; see the denominators in Equations (5) and (7)) are similar in this respect and their empirical region of applicability does not differ much from the theoretical region of existence of trivariate normal distributions (see the second and third columns from the left in Figure 3).

At the other end, the Hittner–May–Silver test is applicable for all triplets on the cube and it is thus not displayed in Figure 3. The remaining statistics vary in their region of applicability, as seen in Figure 3. All tests are applicable with any triplet (*r*_1*Y*_, *r*_2*Y*_, *r*_12_) compatible with a trivariate normal distribution, except the Meng–Rosenthal–Rubin test without the imposition *f* ≤ 1 discussed in Section 2.9 above. As seen in the rightmost column of Figure 3, not replacing an $f\geq 1/\bar{r^{2}}$ with *f* = 1 prevents computation of the statistic in some areas of the region of existence of trivariate normal distributions, although only when the sample value of *r*_12_ is negative. The consequences of setting versus not setting a bound on *f* will be described later.

### 5.2 Accuracy with trivariate normal observations

**FIGURE 4:**
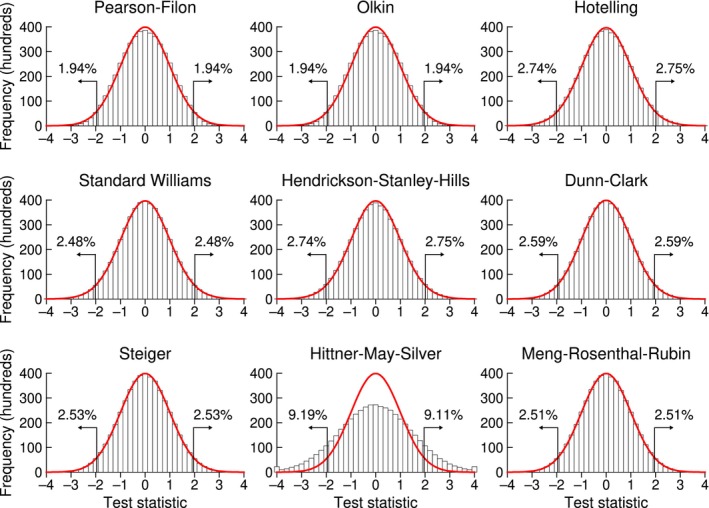
Sampling distribution (histogram) of each test statistic when *ρ*_1*Y*_ = *ρ*_2*Y*_ = .80, *ρ*_12_ = .75, and *n* = 50. The asymptotic distribution (red curve) is drawn to scale. Percentage of cases beyond the critical points for a two‐sided size‐.05 test are indicated at the tails (Colour is available online).

For a preliminary illustration, Figure 4 shows the empirical sampling distribution of each of the nine test statistics in the simulation condition with (*ρ*_1*Y*_, *ρ*_2*Y*_, *ρ*_12_) = (.80, .80, .75) and *n* = 50 (recall that the Zou test does not come in the form of a test statistic whose sampling distribution can be inspected). The continuous curve in each panel is the applicable asymptotic distribution (i.e., standard normal or Student's *t* with *n* − 3 degrees of freedom), to which empirical distributions generally match except for the Hittner–May–Silver test. Empirical Type I error rates at the nominal size−.05 test are also shown for each tail in the panels of Figure 4.

The similarity between empirical and nominal rejection rates at each tail indicates an acceptable accuracy for most tests, although some of them seem a little conservative (e.g., Pearson–Filon and Olkin) while others seem a little liberal (e.g., Hotelling and Hendrickson–Stanley–Hills), leaving aside the unacceptably liberal Hittner–May–Silver test. Empirical sampling distributions are also approximately symmetric in Figure 4, as was the case for the all other simulation conditions. For this reason, subsequent graphical results display Type I error rates aggregated over tails.

**FIGURE 5:**
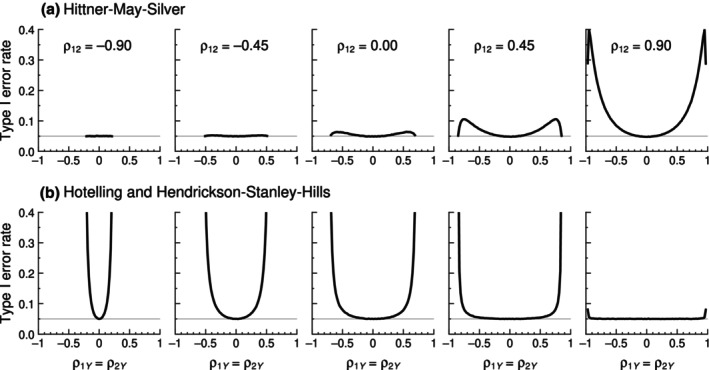
Type I error rates of inaccurate tests as a function the actual value of *ρ*_1*Y*_ = *ρ*_2*Y*_ (horizontal axis) for selected values of *ρ*_12_ (columns) when sample size is *n* = 200. The nominal *α* = .05 level is indicated by a thin horizontal line.

Figure 5 displays summary results (at *n* = 200) for the three tests whose accuracy proved unacceptable but whose Type I error rates turned out to be essentially invariant with sample size *n*. The Hittner–May–Silver test (Figure 5a) is accurate only when *ρ*_1*Y*_ = *ρ*_2*Y*_ is very near zero, something that is generally the case when *ρ*_12_ is negative (see the top row in Figure 1). Yet, as the positive value of *ρ*_12_ increases, the test becomes increasingly liberal as the common value of *ρ*_1*Y*_ and *ρ*_2*Y*_ (under the null) moves away from zero. On the other hand, the Hotelling and Hendrickson–Stanley–Hills tests (Figure 5b) are very (and identically) inaccurate when *ρ*_12_ is negative unless *ρ*_1*Y*_ = *ρ*_2*Y*_ is virtually zero, whereas their accuracy increases across the range of values that *ρ*_1*Y*_ = *ρ*_2*Y*_ can actually take as the positive value of *ρ*_12_ increases.

**FIGURE 6:**
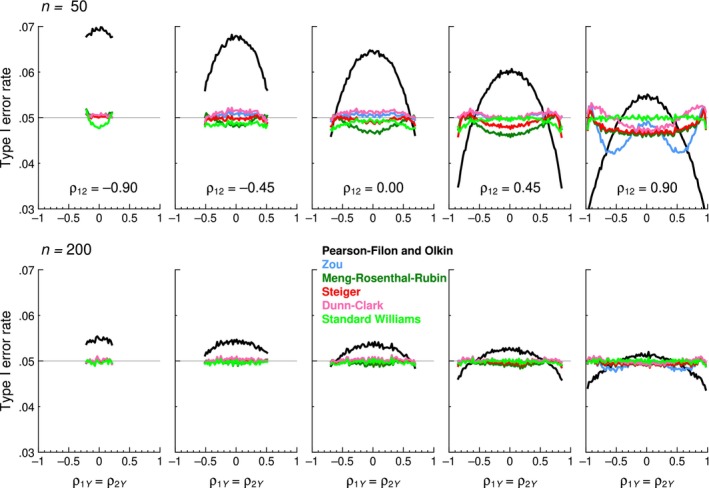
Type I error rates of seven tests (see legend) as a function the actual value of *ρ*_1*Y*_ = *ρ*_2*Y*_ (horizontal axis) for selected values of *ρ*_12_ (columns). Sample sizes are *n* = 50 (top row) and *n* = 200 (bottom row). The nominal *α* = .05 level is indicated by a thin horizontal line (Colour is available online).

Results for the seven other tests are displayed in Figure 6 in another format, where the vertical axis in each panel also spans a narrower range (from .03 to .07) than that in Figure 5. The Pearson–Filon and Olkin tests (black curves in Figure 6) have identical (in)accuracy that varies with sample size *n* also identically. Although these two tests are much less inaccurate than those for which results were displayed in Figure 5, they are both outperformed by the five other tests displayed in Figure 6 (coloured curves), whose accuracies are similar at large sample sizes (bottom row in Figure 6). Yet, with small sample sizes (top row in Figure 6) and positive values of *ρ*_12_, differences can be observed that allow the tests to be ranked in increasing order of accuracy down the list in the legend to Figure 6. At one extreme, the Zou test (blue curves) displays a wavy pattern that departs the most from the .05 line when *ρ*_12_ = .9; at the other extreme, the standard Williams test (light green curves) stays closest overall to the .05 line. Full graphical results for all tests at all sample sizes are presented in Section A of the Appendix S1.

**FIGURE 7:**
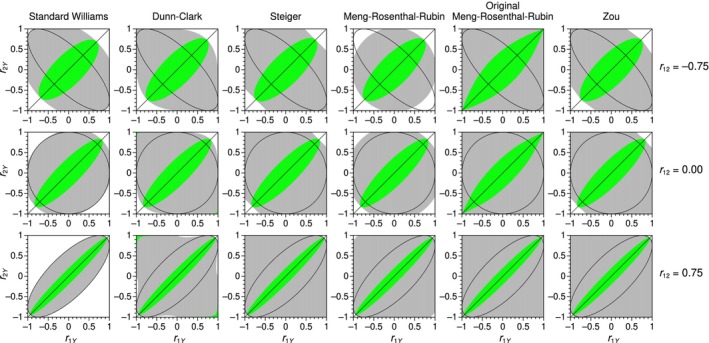
Region of non‐rejection (green area) in the two‐dimensional space with coordinates *r*_1*Y*_ and *r*_2*Y*_ for accurate tests (columns) at selected values for *r*_12_ (rows) and with *n* = 50. Each panel also shows the region of applicability of the test (grey area) and the region of existence of trivariate normal distributions (ellipse) (Colour is available online).

Based on these results on accuracy, only five tests are advisable (standard Williams, Dunn–Clark, Steiger, Meng–Rosenthal–Rubin, and Zou) although the latter is inferior with small samples and large *ρ*_12_. Yet, it is worth looking at the region within which sample values of *r*_1*Y*_ and *r*_2*Y*_ result in non‐rejection of the null by each of these five tests, a region that is in turn confined within the region where each test can actually be used by the requirement of a positive radicand. Figure 7 shows these regions (green areas) for the tests of concern at selected sample values of *r*_12_ (rows) when *n* = 50.

All tests appear virtually identical at any given *r*_12_ within the region of feasible trivariate distributions (ellipse in each panel). It is also worth pointing out that the Dunn–Clark test (second column from the left in Figure 7) displays an anomaly at the top‐left and bottom‐right corners of the panel (i.e., when *r*_1*Y*_ and *r*_2*Y*_ are both large in absolute value but have opposite signs), an anomaly that spreads out to a larger region as *r*_12_ increases and that progressively disappears as the sample size *n* increases. Admittedly, the applicable combinations of *r*_12_, *r*_1*Y*_, and *r*_2*Y*_ in this area are empirically impossible, but structural non‐rejection of the null in such cases defies logic.

This (inconsequential) anomaly of the Dunn–Clark test raises the question of whether some anomaly may also obtain when the Meng–Rosenthal–Rubin test is applied under the admonition to use *f* = 1 in Equation (15) whenever computation of *f* using sample statistics renders a value greater than unity. This replacement has two consequences. One is that it turns an otherwise undefined statistic into a real‐valued one whose magnitude might result in inadequate rejections or non‐rejections; the other is that it alters the value of a well‐defined statistic when $1<f<1/\bar{r^{2}}$.

Results for this (original) version of Meng–Rosenthal–Rubin test are displayed in the fifth column from the right of Figure 7, where it is immediately obvious that the test becomes computable at all points, thus (unnecessarily) extending its applicability. A second feature not apparent in Figure 7 is that the region of non‐rejection is minimally larger for the original form within the region of existence of trivariate normal distributions. This is more apparent with small samples and negative *ρ*_12_ and it is caused by making *f* = 1 when $1<f<1/\bar{r^{2}}$ and, thus, even when the test statistic is well defined. The desirability of an artificially larger non‐rejection area may be contentious, but we see little reason for the admonition to define *f* as the smaller of unity and its computed value via Equation (15).

In sum, as far as accuracy and applicability are concerned, the list of recommendable tests in order of preference are standard Williams, Dunn–Clark, Steiger, Meng–Rosenthal–Rubin (without setting a bound for *f* at unity), and Zou (see accuracies for these tests in Figure 6).

### 5.3 Power with trivariate normal observations

**FIGURE 8:**
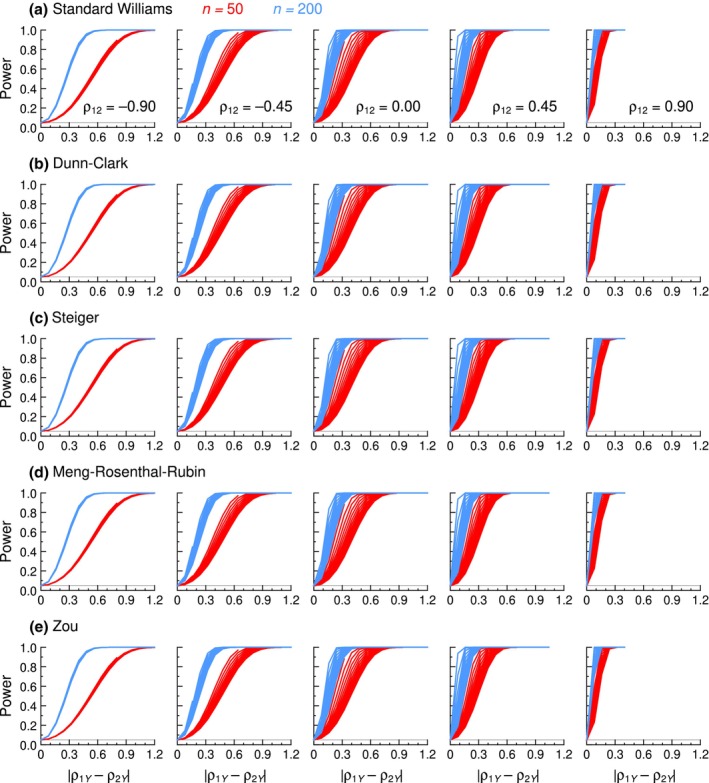
Power of five tests (rows) at selected values of *ρ*_12_ (columns) as a function of the absolute difference between *ρ*_1*Y*_ and *ρ*_2*Y*_ (horizontal axis) when *n* = 50 (red curves) and *n* = 200 (blue curves) (Colour is available online).

Power results are reported here for the five tests just mentioned, but full results for all tests are presented in Section B of the Appendix S1. For a quick glimpse, Figure 8 shows the power of each test (rows) as a function of the absolute difference |*ρ*_1*Y*_ − *ρ*_2*Y*_| at selected values of *ρ*_12_ (columns) along each of the applicable off‐diagonal lines in Figure 2 for *n* = 50 (red curves) and *n* = 200 (blue curves). The most apparent feature is that all tests appear identically powered, as there are no discernible differences across rows. Two other unsurprising characteristics are that power increases with sample size (blue versus red curves in the panels of Figure 8) and with the magnitude of the absolute difference between *ρ*_1*Y*_ and *ρ*_2*Y*_ (horizontal axis in the panels of Figure 8). Two further characteristics are also apparent. One is that the power to detect an absolute difference of some magnitude between *ρ*_1*Y*_ and *ρ*_2*Y*_ (i.e., at any arbitrary point along the horizontal axis) increases with increasing *ρ*_12_ (i.e., rightward each row in Figure 8). The other is that the power curves for different values of (*ρ*_1*Y*_ + *ρ*_2*Y*_)/2 (set of identically coloured curves in each panel) do not superimpose, the less so as *ρ*_12_ approaches zero from either side. Altogether, this means that power is not a simple function of |*ρ*_1*Y*_ − *ρ*_2*Y*_| but that it also depends heavily on (*ρ*_1*Y*_ + *ρ*_2*Y*_)/2 and *ρ*_12_.

**FIGURE 9:**
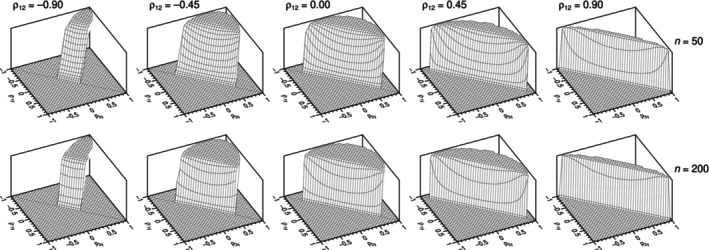
Perspective plots of the power curves plotted in Figure 8 for the standard Williams test when *n* = 50 (top row) and *n* = 200 (bottom row).

This dependence is best appreciated in perspective plots of the power surface defined on the two‐dimensional space of Figure 2 at different values of *ρ*_12_. Figure 9 displays these plots for the standard Williams test; plots for other tests were virtually identical. Note that the identity line in Figure 2 runs slightly tilted from left to right in each panel of Figure 9 and that the off‐diagonal lines in Figure 2, which represent locations with increasing value of |*ρ*_1*Y*_ − *ρ*_2*Y*_| at a fixed value for (*ρ*_1*Y*_ + *ρ*_2*Y*_)/2, run perpendicular to the identity line in the panels of Figure 9, whereas lines parallel to the identity line describe power at locations of constant |*ρ*_1*Y*_ − *ρ*_2*Y*_| for different values for (*ρ*_1*Y*_ + *ρ*_2*Y*_)/2. The U‐shaped form of the latter lines within the region where power increases from 0 to 1 indicates that, for any fixed value of |*ρ*_1*Y*_ − *ρ*_2*Y*_|, power increases as (*ρ*_1*Y*_ + *ρ*_2*Y*_)/2 moves away from 0.

### 5.4 Robustness

**FIGURE 10:**
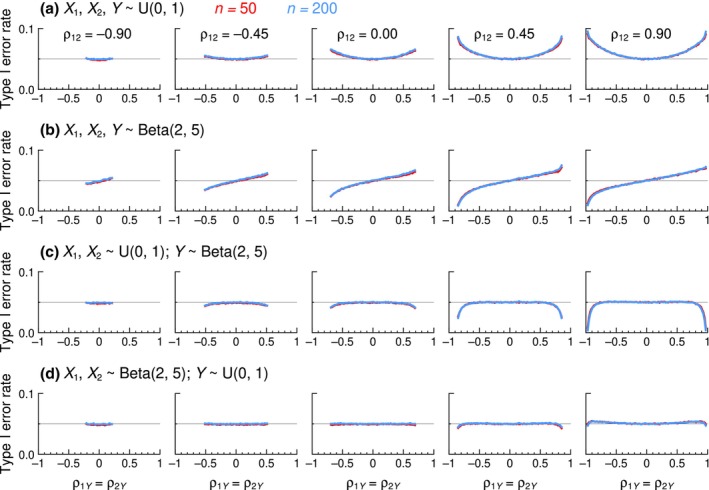
Type I error rates of the standard Williams test as a function of the actual value of *ρ*_1*Y*_ = *ρ*_2*Y*_ (horizontal axis) for selected values of *ρ*_12_ (columns) when (a) all variables have uniform distributions, (b) all variables have Beta(2, 5) distributions, (c) *X*_1_ and *X*_2_ have uniform distributions while *Y* has a Beta(2, 5) distribution, and (d) *X*_1_ and *X*_2_ have Beta(2, 5) distributions while *Y* has a uniform distribution. Sample sizes are *n* = 50 (red curves) and *n* = 200 (blue curves). The nominal *α* = .05 level is indicated by a thin horizontal line (Colour is available online).

Non‐normality deteriorated accuracy similarly for all tests, on top of the fact that tests already differed in accuracy under trivariate normality (see Figures 5 and 6). The magnitude and form of deterioration differed greatly across the cases under study. To illustrate the mildest deterioration, Figure 10 shows aggregated Type I error rates for the standard Williams test when all variables had uniform distributions (Figure 10a), Beta(2, 5) distributions (Figure 10b), or mixed uniform and Beta(2, 5) distributions (Figure 10c,d). In these cases, the deterioration was largely invariant with sample size (compare the red and blue curves in each panel of Figure 10) and was milder when the univariate distribution of *Y* differed from the common univariate distribution of *X*_1_ and *X*_2_ (Figure 10c,d) than it was when the three variables had the same univariate distribution (Figure 10a,b). In comparison to the corresponding plots in Figure 6 for accuracy of the same test (light green curves) under trivariate normality, uniform distributions for all variables (Figure 10a) do not affect accuracy when *ρ*_1*Y*_ = *ρ*_2*Y*_ are very close to zero but they make the test increasingly liberal as *ρ*_1*Y*_ = *ρ*_2*Y*_ move away from zero within their possible range of values (which broadens as *ρ*_12_ increases). Beta(2, 5) distributions for all variables (Figure 10b) also do not affect the accuracy of the test when *ρ*_1*Y*_ = *ρ*_2*Y*_ are virtually zero but, in contrast, they make the test increasingly more conservative (liberal) as *ρ*_1*Y*_ = *ρ*_2*Y*_ become increasingly negative (positive) within their possible range of values. Mixed forms of distribution across variables (Figure 10c,d) clearly reduced the deterioration, a result that is likely limited to the particular distributions considered here. Non‐normality also resulted in symmetric sampling distributions of test statistics that were also centred on zero (i.e., similar to what was shown in Figure 4 under normality) and, thus, Type I error rates in each of the tails were virtually identical. The remaining tests with acceptable accuracy under normality (see Figure 6) displayed a deterioration under non‐normality that was similar to that shown in Figure 10 for the standard Williams test.

**FIGURE 11:**
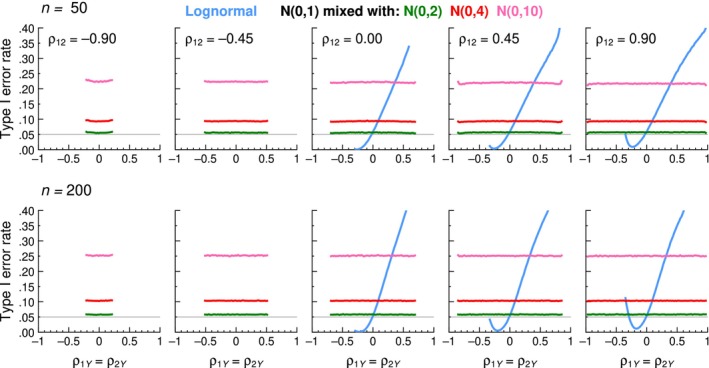
Type I error rates of the standard Williams test as a function of the actual value of *ρ*_1*Y*_ = *ρ*_2*Y*_ (horizontal axis) for selected values of *ρ*_12_ (columns) when all variables have lognormal distributions (blue curves), or follow normal mixtures as indicated in the legend at the top. The feasible range of values for *ρ*_12_, *ρ*_1*Y*_, and *ρ*_2*Y*_ with normal mixtures is the same as that for normal variables, whereas the feasible range for lognormal distributions is narrower and renders no possible triplets for the leftmost two panels in each row. Sample sizes are *n* = 50 (top row) and *n* = 200 (bottom row). The nominal *α* = .05 level is indicated by a thin horizontal line (Colour is available online).

The lack of generality of the robustness results just discussed is corroborated by the results obtained for the remaining forms of distribution listed in Table 1, which are also illustrated in Figure 11 in a different form and with a broader range of Type I error rates (between 0 and .4, compared to between 0 and .1 in Figure 10) for the standard Williams test. The lognormal distribution (blue curves in Figure 11) resulted in the most dramatic and nonlinear deterioration. On the other hand, normal mixtures (green, red, and pink curves in Figure 11) resulted in a deterioration whose magnitude was constant across the board but increased with the variance difference between the two components of the mixture. In addition, the magnitude of deterioration increased with sample size (top versus bottom rows in Figure 11). Full results for all tests are presented in Sections C, E, and G–J of the Appendix S1.

Given the preceding results, it comes as no surprise that power curves for the cases considered in Figure 10 had the same features displayed in Figures 8 and 9 for trivariate normal data. Power curves for the cases considered in Figure 11 were similarly sigmoidal, although they increased from a lower asymptote at the inappropriate Type I error rates depicted in Figure 11. Graphical presentation of these results is deferred to Sections D and F of the Appendix S1.

## 6 EMPIRICAL EXAMPLES

Correlational studies are the natural context of application of the tests considered in this paper, but tests are also needed in other types of study in which dependent correlations are compared for whatever reason (e.g., Blake & Palmisano, 2021; Ćoso et al., 2023). A research area in which these tests are frequently needed is the search for cognitive or behavioural correlates of the 2D–4D digit ratio (i.e., the ratio of the lengths of the index and ring fingers), and this is the area from which the coming examples were drawn. Another area of application is research on correlates of state versus trait anxiety (e.g., Guo et al., 2022; Menculini et al., 2023), but no examples will be given from this context. It should be noted that the following examples do not come from papers in which the core of the analysis was a comparison of correlations, and the only criterion guiding the choice of papers was availability of the value of *r*_12_ in the paper itself, a correlation that is rarely reported when the nominal correlations of concern are only *r*_1*Y*_ and *r*_2*Y*_.

Since the 2D–4D digit ratio can be measured on each of the hands, a common question of interest is whether the target variable *Y* correlates equally with the right‐hand ratio (*X*_1_) and the left‐hand ratio (*X*_2_). For instance, Brosnan et al. (2011) reported that the correlation between right‐hand digit ratio and Java grade in a sample of 73 first‐year students taking a course in Java programming reached statistical significance (*r*_1*Y*_ = −.22; *p* = .03) but not so for the left‐hand ratio (*r*_2*Y*_ = −.13; n.s.), whereas the left‐ and right‐hand ratios were significantly correlated (*r*_12_ = .46; *p* < .01). Use of the standard Williams test yields *t*_W_ = −0.7420 with *p* = .461, so that there is no basis for supporting the notion that digit ratios in the right versus the left hand are differently correlated to Java grades, despite the anecdotal result that one of the correlations is significantly different from zero and the other is not.

In another paper from the same research field, Uzun and Tok (2023) reported correlational analyses of right‐hand and left‐hand digit ratios (variables *X*_1_ and *X*_2_) with attention gathering skill (variable *Y*) in 112 children aged 60–72 months. The correlation was significant for the right hand (*r*_1*Y*_ = .209; *p* < .05) but not for the left hand (*r*_2*Y*_ = .086; n.s.), while the left‐ and right‐hand ratios correlated *r*_12_ = .114 (which is not significantly different from zero). Use of the standard Williams test yields *t*_W_ = 0.9846 with *p* = .327, so that there is no basis for supporting the notion that digit ratios in the right versus the left hand are differently correlated to attention‐gathering skill, despite the anecdotal result that only one of the correlations is significantly different from zero (Incidentally, table 7 in Uzun and Tok (2023) gives *r*_2*Y*_ = −.086; if this were the actual value, the standard Williams test would yield *t*_W_ = 2.3795 with *p* = .019, rejecting the null that attention‐gathering skill holds the same relation to the right‐ and left‐hand digit ratio).

Also of interest in this area is the search for associations between digit ratio in one of the hands and personality factors, cognitive performance in various tasks, or behavioural or physiological measures. In such cases, digit ratio plays the role of variable *Y* whereas the other measures define a set of *J* variables *X*_*j*_ (with 1 ≤ *j* ≤ *J* and *J* > 2). At first sight, this situation may seem to imply the null H_0_: *ρ*_1*Y*_ = *ρ*_2*Y*_ = … = *ρ*_*JY*_, for which statistical tests have been developed (see, for example, Choi, 1977; Cohen, 1989; Olkin & Finn, 1990). Yet, the context for such a null typically implies that the *X*_*j*_ are measures of the same variable at different time points in a longitudinal study and the goal is to check out whether the correlation between *X* and *Y* across the *J* occasions remains stable. In the context of our examples here, there is no theoretical expectation that digit ratio should hold the same relation with, say, all dimensions of personality. The actual research question is instead whether digit ratio is more strongly associated with some personality dimension than it is with others (see, for example, Fink et al., 2004) and, thus, it requires pairwise analyses with multiple tests of the simple null H_0_: *ρ*_*jY*_ = *ρ*_*kY*_ for all *j* ≠ *k*. Although each of these multiple tests leads to an individual claim (and, hence, there is no risk of inflation of Type I error rates that might demand alpha adjustments; see García‐Pérez, 2023; Rubin, 2021, 2024), a researcher may still want to set the alpha level different from the usual .05 for other reasons (Maier & Lakens, 2022).

The handling of comparisons of correlations in a case like this one is illustrated here with data from Del Giudice and Angeleri (2016), a study in which 135 boys and 150 girls contributed measures of right‐hand digit ratio (variable *Y*) and three measures of attachment styles: avoidance (variable *X*_1_), preoccupation (variable *X*_2_), and felt security (variable *X*_3_). Intercorrelations among these variables were reported in their Table 1 separately for the samples of boys and girls. Results of our pairwise analyses of equality of correlations with the standard Williams test in the sample of girls are presented in Table 2, with the outcome that equality of correlation with digit ratio is not rejected for any pair of attachment styles. It should be stressed that an analogous and independent set of tests for the sample of boys could be conducted but, depending on the goal of the analysis, it might be more fitting to use the ANOVA‐like strategy developed by Bilker et al. (2004) for that purpose.

**TABLE 2:** Pairwise standard Williams tests of equality of dependent correlations for data from the sample of girls in the study of Del Giudice and Angeleri (2016).

Pairing	*r*_*jY*_	*r*_*kY*_	*r*_*jk*_	*t*_W_	*p*
*j* = 1, *k* = 2	−.11	.10	−.45	−1.5067	.134
*j* = 1, *k* = 3	−.11	−.03	−.33	−0.5979	.551
*j* = 2, *k* = 3	.10	−.03	−.30	0.9817	.328

## 7 DISCUSSION

### 7.1 Summary of results

Our comparative analysis of the performance of the 10 tests can be summarized as follows. When variables have a trivariate normal distribution:

1. all tests are applicable under any possible combination of the values that sample correlations may take. This holds for the Meng–Rosenthal–Rubin test only when administered with the original admonition to replace any occurrence of *f* > 1 in Equation (15) with *f* = 1.
2. Type I error rates stay at their nominal level across all possible combinations of population correlations for only five of the tests (in order of preference, standard Williams, Dunn–Clark, Steiger, Meng–Rosenthal–Rubin, and Zou), whose Type I error rates are also barely affected by sample size.
3. power curves are similar for the five tests just mentioned and, across the board, power increases with sample size and with effect size defined as the absolute value of the difference between *ρ*_1*Y*_ and *ρ*_2*Y*_. Two additional factors that modulate power are the average of the two population correlations *ρ*_1*Y*_ and *ρ*_2*Y*_ under comparison (with power increasing faster with effect size as this average correlation moves away from zero) and the value of the population correlation *ρ*_12_ (with power also increasing faster with effect size as *ρ*_12_ increases). The former feature was anecdotally noted by May and Hittner (1997b). The presence of these modulating factors raises a warning flag on the results of power analyses that only consider effect size defined as a difference in correlation (see Faul et al., 2009).

When variables are instead non‐normally distributed in the limited set of forms explored here, accuracy of the five tests deteriorates to varying extents from negligibly in some cases (see Figure 10) to unacceptably in others (see Figure 11). Further comments on non‐normal distributions are deferred to the next subsection.

The results just summarized modulate those reported in previous papers that addressed the analysis for a smaller number of test statistics, under a narrower set of population correlations, with smaller samples, and with fewer replications to estimate Type I error rates and power. By exploring combinations of population correlations more thoroughly than was done in previous studies and by using substantially more replications to estimate Type I error rates and power, this study has identified inadequate performance of some tests whose deficiencies only show within certain ranges of the parameter space (see, for example, accuracy results in Figure 5). Analogously, a presumed inaccuracy of the standard Williams test with trivariate normal observations when *ρ*_1*Y*_ = *ρ*_2*Y*_ = .7 and *ρ*_12_ = .1 for *n* ∈ {20, 50, 100, 300} and Type I error rates estimated with 2000 replications (see, for example, May & Hittner, 1997a; see also Hittner et al., 2003) was not replicated in our results, which do not show any sign of a singularity at or near this region (see the light green curves in Figure 6).

### 7.2 Non‐normal distributions

The non‐normal distributions included in our study offer a suitable account of the diverse performance of the tests under plausible scenarios, but no study can include all of the non‐normal distributions that can be found in empirical data (see Cain et al., 2017). For instance, some variables are subject to order restrictions such that *X*_*i*_ < *Y*_*i*_ for every paired observation *i* in the sample (e.g., years of marriage and age), a feature that incorporates irrelevant structural components into the magnitude of sample correlations (see García‐Pérez & Núñez‐Antón, 2013). Our results show that non‐normality per se is not necessarily a threat to the accuracy of tests for equality of dependent correlations with overlapping variables (compare Figures 10 and 11), although it seems impossible to determine in advance whether the tests will be robust to the particular (and unknown) form of non‐normality of the distributions of any data on hand.

One might think of checking for form of distribution to decide whether any of these tests is safe to use under the circumstances, perhaps leading to preliminary simulations of accuracy under the particular forms of distribution that have been identified. It should nevertheless be noted that such an identification is impossible in strict sense, as one can only test (and then simply reject or fail to reject) a null hypothesis about some particular form of distribution. For instance, with typical small samples, normality may often be incorrectly not rejected when the data actually come from contaminated normal distributions such as those considered here (see Table 1). And this preliminary testing for normality also brings its own inflation of Type I error rates due to the conditional approach (see García‐Pérez, 2012).

One could also think of using alternative tests that are robust to violations of normality, none of which have been explored in this paper for reasons discussed next. Wilcox and Tian (2008) considered six such alternative tests in a comparison of accuracy and power with those of the standard Williams test under normality and non‐normality (uniform or exponential distributions for all variables). They discarded four of the tests (designated B1, B2, B3, and B4) for various reasons, and their simulations (which were somewhat limited in scope) showed that the two other tests (designated D1 and D2) slightly outperformed the standard Williams test when data were drawn from non‐normal distributions, but only by the criterion that Type I error rates do not exceed the nominal *α* level. Yet, neither of these two tests fared well in comparison to the standard Williams test when data were instead drawn from normal distributions. Given the difficulty of ascertaining whether the data on hand come or do not come from normal distributions, using these alternative tests cannot be recommended in general. Alternative tests do not seem to be available that are robust to violations of normality and remain accurate under normality.

Another option is to address the hypothesis of equality of dependent correlations with overlapping variables via replacement of Pearson's correlation with an alternative correlation coefficient for which tests have been developed that are robust to violation of normality. Some such tests have been developed with mixed results in a narrow set of simulation conditions (see, for example, Wilcox, 2016). Nevertheless, it should be noted that the statistical analysis of data must be guided by the research question that a study addresses; replacing Pearson's correlation with an alternative coefficient may not then be appropriate to address the research question.

We must also stress that our study only included data generated to have specific univariate distributions whose higher‐order moments are naturally predetermined. Alternatively, non‐normal data can be generated to have predefined higher‐order moments (skewness and kurtosis) without specification of the form of the distribution. This may seem to capture more realistically the features of psychologically relevant data distributions (see, for example, Cain et al., 2017), which immensely broadens the set of scenarios for an analysis of the robustness of the statistical tests of equality of dependent correlations. In addition, Fairchild et al. (2024) have shown that different algorithms that nominally generate non‐normal data with identical levels of skewness and kurtosis produce distributions with distinctly different univariate shapes, with potential implications for the resulting robustness of the tests under study. In consequence, the generalizability of the results of any study on robustness to violations of normality is always uncertain.

Another aspect that must be kept in mind when using these tests in practical applications with (potentially) non‐normally distributed variables is that the mere possibility that the null is true is subject to serious structural threats. Consider the hypothetical case in which *X*_1_ has a Lognormal(0, 1) distribution, *X*_2_ has a Beta(2, 5) distribution, and *Y* has a chi‐square distribution with 10 degrees of freedom. In these circumstances, Xiang (2019) showed that the ranges of possible correlations vary greatly across pairs of variables: *ρ*_1*Y*_ ⊂ (−.640, .862), *ρ*_2*Y*_ ⊂ (−.928, .996), and *ρ*_12_ ⊂ (−.646, .815). Thus, structurally, there is a very limited range of true nulls on consideration that positive‐definiteness is additionally imposed on the triplets of intercorrelations. There may actually be occasions when *ρ*_1*Y*_ and *ρ*_2*Y*_ will both be near their maximum (or minimum) values and, yet, their magnitude will differ. General recommendations may not be given for handling these situations in practice although, in a sense, the utility of a correlation lies in its practical implications and not so much in the extent to which it is free of a structural consequence of the forms of distribution of the variables of concern.

### 7.3 Testing for equality of strength of correlation, regardless of sign

**FIGURE 12:**
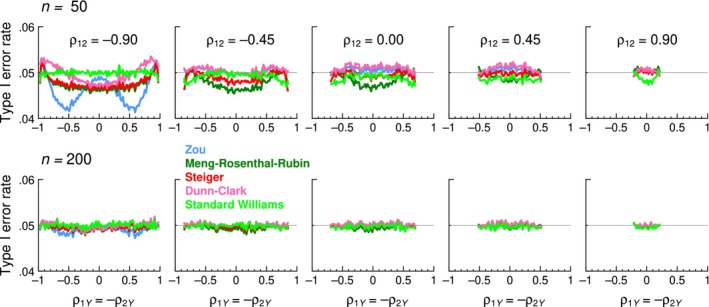
Type I error rates of accurate tests (see legend) as a function the actual value of *ρ*_1*Y*_ = −*ρ*_2*Y*_ (horizontal axis) for selected values of *ρ*_12_ (columns). Sample sizes are *n* = 50 (top row) and *n* = 200 (bottom row). The nominal *α* = .05 level is indicated by a thin horizontal line (Colour is available online).

What matters in a number of practical situations is whether the *unsigned strength* of the association of two alternative variables with a target variable differs. For instance, for predictive purposes, the sign of correlation is irrelevant and the relative merits of two candidate predictors depend on whether the absolute value of their correlations with the target variable differ. This entails the null hypothesis H_0_: |*ρ*_1*Y*_| = |*ρ*_2*Y*_|, for which a test does not seem to have been developed. Yet, this null hypothesis branches out into two tractable nulls, namely, H_0_: *ρ*_1*Y*_ = *ρ*_2*Y*_ (for testing that both correlations are equal in value and sign, which is the case considered thus far in this paper) and H_0_: *ρ*_1*Y*_ = −*ρ*_2*Y*_ (for testing that both correlations differ only in sign). As discussed by Boyer et al. (1983) in cases in which *r*_1*Y*_ and *r*_2*Y*_ differ in sign, this difference in sign can be circumvented by testing instead the null H_0_: *ρ*_1*Y*_ = *ρ*_−2*Y*_, where *ρ*_−2*Y*_ stands for the correlation between –*X*_2_ and *Y*. Thus, this test of equality of unsigned strength of correlation is applied identically to the previous one but using instead the correlation between *X*_1_ and –*X*_2_ and the correlation between –*X*_2_ and *Y*. An analysis of Type I error rates and power analogous to that presented above was conducted for the same 10 tests considered in this paper and the results were identical. For illustration, Figure 12 shows accuracy results under trivariate normality, which are analogous to those in Figure 6. The only natural difference is that results for negative and positive values of *ρ*_12_ are swapped because true nulls now lie on the off‐diagonal in the panels of Figure 1, where *ρ*_1*Y*_ = −*ρ*_2*Y*_.

In the absence of a proper test of the null H_0_: |*ρ*_1*Y*_| = |*ρ*_2*Y*_|, it should be stressed that the choice between testing H_0_: *ρ*_1*Y*_ = *ρ*_2*Y*_ and testing H_0_: *ρ*_1*Y*_ = −*ρ*_2*Y*_ must be based (and justified) on a priori theoretical or practical considerations. The result of using one or the other will generally differ, a natural consequence of the fact that each of the nulls captures a different reality. For instance, consider a situation in which *r*_1*Y*_ = −.22, *r*_2*Y*_ = .17, and *r*_12_ = −.35 with a sample size of 100. Use of the standard Williams test for H_0_: *ρ*_1*Y*_ = *ρ*_2*Y*_ yields *t*_W_ = −2.4077 with *p* = .018; in apparent contrast, use of the same test for H_0_: *ρ*_1*Y*_ = −*ρ*_2*Y*_ yields *t*_W_ = 0.4437 with *p* = .658. Although the test comes out significant in the former case and not in the latter, one has to realize that the first test rejects the null that the two correlations have the same magnitude and sign whereas the second test does not reject the null that the two correlations have the same magnitude regardless of sign. It is incumbent on the researcher to decide which of the two tests captures the statistical aspects of the research question that motivated the comparison of correlations. Nevertheless, a pattern of sample correlations similar to that in the preceding example (namely, *r*_1*Y*_ and *r*_2*Y*_ with different signs along with negative and relatively large *r*_12_) is suggestive of a negative relation between *X*_1_ and *X*_2_ that naturally results in correlations between *X*_1_ and *Y* and between *X*_2_ and *Y* that differ in sign. In contrast, a different pattern in which *r*_12_ is relatively large and positive will naturally result in correlations between *X*_1_ and *Y* and between *X*_2_ and *Y* that have the same sign.

### 7.4 Extensions

A natural extension of the study reported in this paper consists of conducting analogous analyses under the remaining scenarios described in the Introduction, namely, equality of independent correlations and equality of dependent correlations with non‐overlapping variables. Indeed, there are a number of alternative tests for each of these scenarios (see Diedenhofen & Musch, 2015) but a study along those lines is beyond the scope of the present paper.

### 7.5 Practical recommendations

Our results advise against the use of Pearson–Filon, Olkin, Hotelling, Hendrickson–Stanley–Hills, and Hittner–May–Silver tests of equality of dependent correlations with overlapping variables. The five remaining tests (standard Williams, Dunn–Clark, Steiger, Meng–Rosenthal–Rubin, and Zou) are all dependable and virtually identical as regards their accuracy, power, and applicability under normality, but they are robust to violation of the normality assumption only under some forms of non‐normality that are impossible to identify for actual data.

Needless to say, the core recommendation is to compare dependent correlations with overlapping variables by using one of these five dependable tests. Given their uncertain robustness, use of these tests in practical applications should perhaps be accompanied by tests of normality for each of the variables of concern, not with the purpose of validating the tests of equality of correlations themselves but with the purpose of modulating their interpretation. Specifically, the statistical validity of the results of one of the above‐mentioned tests of equality of dependent correlations with overlapping variables is less questionable if compatibility with normal distributions is not rejected by normality tests within their own limitations of accuracy and power.

The simple practice of comparing correlations by eye or via the *p*‐values of isolated tests of significance with respect to zero should also be avoided. The prevalence of such practices is hard to investigate because of the absence of an identifiable characteristic of the papers reporting them that could lead to database searches. Yet, reporting practices involving proper use of tests is easier to investigate. The Introduction mentioned a search for papers that cited *cocor* (Diedenhofen & Musch, 2015) and reported the use of tests of equality of dependent correlations, with the results listed in Table A1 in Appendix A. Note that 10 of the papers did not report which test had been used, one reported that all tests gave the same result, and seven reported using the defective Pearson–Filon or Hittner–May–Silver tests. Only nine papers reported using the dependable Dunn–Clark, Steiger, or Meng–Rosenthal–Rubin tests. In the interest of transparency and best reporting practices, the particular test that was chosen in a given study should be reported and the choice should be among the tests that have proven dependable under normality, with a preference for the standard Williams test over all others.

## AUTHOR CONTRIBUTIONS

**Miguel A. García‐Pérez:** Conceptualization; methodology; software; data curation; investigation; validation; formal analysis; supervision; resources; project administration; visualization; writing – review and editing; funding acquisition; writing – original draft.

## 8 DISCLOSURE OF ARTIFICIAL INTELLIGENCE‐GENERATED CONTENT (AIGC) TOOLS

The author did not use generative AI or any other similar tools at any step of this research or for any purpose.

## 9 CONFLICTS OF INTEREST STATEMENT

The author declares no conflicts of interest.

## Supporting information


Appendix S1.

## Figures and Tables

**FIGURE B1:**
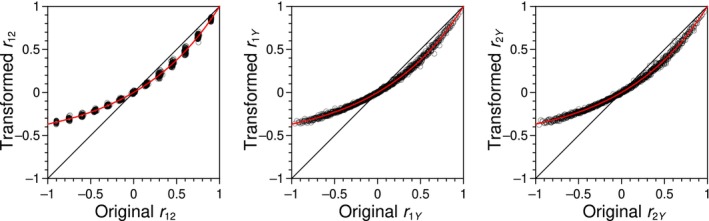
Relation between original correlations of normally distributed observations (abscissa) and correlations for their exponentially transformed counterparts (ordinate). Each symbol comes from a different sample of 10,000 triplets with original intercorrelations over the full range of conditions involved in our simulation studies. The red curve in each panel is the theoretical relation in Equation (B2). A diagonal identity line is displayed for reference. (Colour is available online).

**FIGURE B2:**
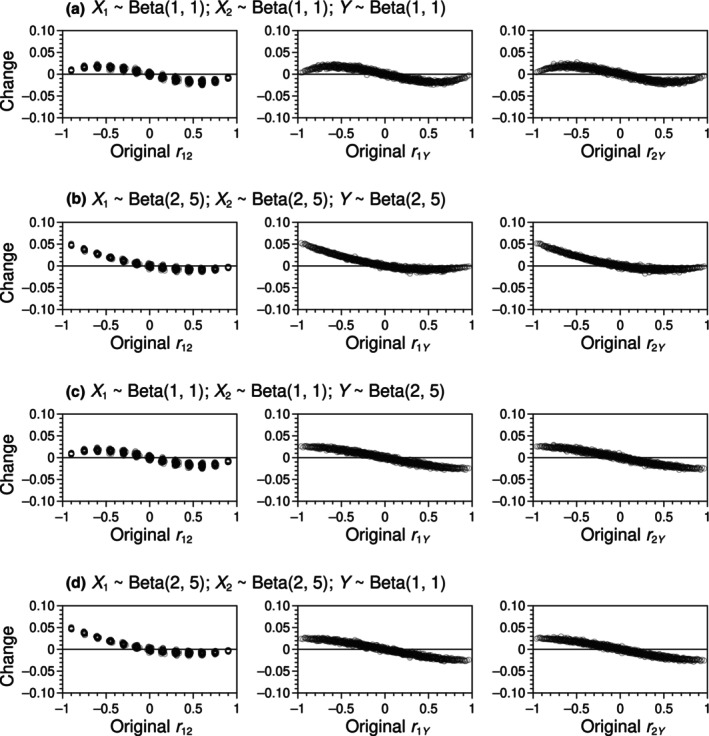
Signed change in correlation (ordinate in each panel) produced when trivariate normal variables are differently transformed (rows) to produce variables with univariate beta distributions, as a function of the original value of the correlations (abscissa in each panel). Each symbol comes from a different sample of 10,000 triplets with original intercorrelations over the full range of conditions involved in our simulation studies. A horizontal line at an ordinate of zero is displayed for reference.

## Data Availability

FORTRAN source code and output files for the accuracy and power studies reported in this paper are available at https://osf.io/4pw7r/. The source code calls NAG Library Mark 19 subroutines and it will not run if compiled without access to them.

## APPENDIX A Statistical test reportedly used in papers citing Diedenhofen and Musch (2015)

**TABLE A1:** Statistical tests used in papers that cite *cocor* (Diedenhofen & Musch, 2015) as the tool to test for equality of dependent correlations with overlapping variables.

Paper doi	Test used
https://doi.org/10.1027/1015‐5759/a000682	Not reported
https://doi.org/10.1002/jclp.23624	Not reported
https://doi.org/10.1080/1359432X.2023.2193692	Not reported
https://doi.org/10.1037/aca0000539	Not reported
https://doi.org/10.1177/01461672231210465	Not reported
https://doi.org/10.1007/s00426‐022‐01779‐4	Not reported
https://doi.org/10.1037/pas0001249	Not reported
https://doi.org/10.1016/j.jenvp.2022.101919	Not reported
https://doi.org/10.1007/s12144‐021‐01722‐7	Not reported
https://doi.org/10.1186/s40359‐023‐01111‐8	Not reported
https://doi.org/10.1037/emo0001013	All tests
https://doi.org/10.1177/09567976231185127	Pearson–Filon
https://doi.org/10.1027/1618‐3169/a000586	Pearson–Filon
https://doi.org/10.1002/ejsp.3019	Hittner–May–Silver
https://doi.org/10.1027/2698‐1866/a000037	Hittner–May–Silver
https://doi.org/10.1027/1015‐5759/a000717	Hittner–May–Silver
https://doi.org/10.1002/casp.2695	Hittner–May–Silver
https://doi.org/10.1525/collabra.88330	Hittner–May–Silver
https://doi.org/10.1016/j.cogpsych.2023.101550	Dunn–Clark
https://doi.org/10.1037/emo0001229	Steiger
https://doi.org/10.1007/s10869‐023‐09893‐9	Steiger
https://doi.org/10.1007/s12144‐023‐05183‐y	Steiger
https://doi.org/10.3389/fpsyg.2023.1165911	Steiger
https://doi.org/10.1037/aca0000573	Steiger
https://doi.org/10.1080/08870446.2023.2196994	Meng–Rosenthal–Rubin
https://doi.org/10.1186/s40359‐023‐01463‐1	Meng–Rosenthal–Rubin
https://doi.org/10.1080/02134748.2023.2171560	Meng–Rosenthal–Rubin

## APPENDIX B Effects of nonlinear transformations on intercorrelations

### B.1

This appendix analyses the consequences of nonlinear transformations for the intercorrelations among variables that originally have a trivariate standard normal distribution with correlation matrix

$$Ρ=\left(\begin{array}{ccc} 1 & \rho_{X_{1}X_{2}} & \rho_{X_{1}X_{3}}\\ \rho_{X_{1}X_{2}} & 1 & \rho_{X_{2}X_{3}}\\ \rho_{X_{1}X_{3}} & \rho_{X_{2}X_{3}} & 1 \end{array}\right).$$

Let

(B1)
$${\tilde{X}}_{i}=f_{i}\left(X_{i}\right),\;\text{for}\;i\in \left\{1,2,3\right\},$$

where *f*_*i*_ is an arbitrary nonlinear function such that the distribution of ${\tilde{X}}_{i}$ becomes non‐normal. For notational convenience, we refer to the third variable here as *X*_3_ instead of *Y* on the understanding that $X_{3}\;\;:\;=Y$. We are interested in finding out what the correlation matrix

$$\tilde{Ρ}=\left(\begin{array}{ccc} 1 & \rho_{{\tilde{X}}_{1}{\tilde{X}}_{2}} & \rho_{{\tilde{X}}_{1}{\tilde{X}}_{3}}\\ \rho_{{\tilde{X}}_{1}{\tilde{X}}_{2}} & 1 & \rho_{{\tilde{X}}_{2}{\tilde{X}}_{3}}\\ \rho_{{\tilde{X}}_{1}{\tilde{X}}_{3}} & \rho_{{\tilde{X}}_{2}{\tilde{X}}_{3}} & 1 \end{array}\right)$$

is for the nonlinearly transformed variables. A general answer to this question does not exist, but some cases have been analysed that lend themselves to finding out what is the minimum correlation attainable for non‐normal variables with arbitrary univariate distributions (e.g., Dukic & Marić, 2013; see also Xiang, 2019). For the purpose of this paper, it is useful to start dissecting a sample case that can be easily worked out analytically and where the consequences of nonlinear transformations are readily apparent. Let ${\tilde{X}}_{i}=f_{i}\left(X_{i}\right)=\exp\left(X_{i}\right)$ in Equation (B1) so that each of the original normal variables is transformed into one that has a lognormal distribution. This transformation is mathematically tractable and allows us to obtain.

(B2)
$$\rho_{{\tilde{X}}_{i}{\tilde{X}}_{j}}=\frac{\exp\left(\rho_{X_{i}X_{j}}\right)-1}{\exp\left(1\right)-1},\;\text{for}\;i,j\in \left\{1,2,3\right\}$$

(De Veaux, 1976). The red curve in the panels of Figure B1 displays this relation, where a nonlinear transformation of correlations is immediately apparent along with the fact that the minimum correlation between exponentially transformed normal variables is –exp(−1) ≈ −.3679 (De Veaux, 1976). To further illustrate this effect on correlations, we generated a sample of *n* = 10,000 triplets (*X*_1_, *X*_2_, *X*_3_) from a trivariate normal distribution with the applicable correlation matrix under each of the conditions involved in the accuracy study reported in this paper, transformed each triplet (*X*_1_, *X*_2_, *X*_3_) into $\left({\tilde{X}}_{1},{\tilde{X}}_{2},{\tilde{X}}_{3}\right)=\left(\exp\left(X_{1}\right),,,\exp\left(X_{2}\right),,,\exp\left(X_{3}\right)\right)$, and computed the sample intercorrelations among the original and among the transformed variables. The sample pairwise correlations obtained for the transformed variables are plotted as the ordinate of symbols in the panels of Figure B1, each at the abscissa pertaining to the corresponding pairwise correlation between the originating normal variables. The symbols naturally pack along the theoretical relation in Equation (B2) to within sampling error.

The consequence on estimates of, for example, empirical Type I error rates under violation of trivariate normality when non‐normal data are generated in this way is immediately apparent. Results obtained from trivariate normal data in the condition, say, *ρ*_1*Y*_ = *ρ*_2*Y*_ = .8 with *ρ*_12_ = .75 belong, by Equation (B2), to the condition *ρ*_1*Y*_ = *ρ*_2*Y*_ = .71 with *ρ*_12_ = .65 when the original data are transformed by Equation (B1) to have a lognormal distribution. There is nothing essentially incorrect in this approach, but interpretation of the results must keep in mind that conditions involving different forms of distribution may also bring differences in the values of population correlations to which they apply. Yet, this was only an illustrative example and we are actually interested in the consequences of the transformations used in this study to generate beta distributions.

A closed‐form expression analogous to Equation (B2) does not exist to relate the intercorrelations among trivariate normal variables to those of variables transformed to have beta distributions. Yet, the simulation approach illustrated in Figure B1 can be used to investigate the shape of this relation for all of the cases involved in our simulations, which cover different combinations of beta variables. Plots of the results in the form of Figure B1 showed that symbols fall virtually on the diagonal identity line, which hides the minor effects that these transformations have on the resultant intercorrelations. For this reason, the ordinate of the panels in Figure B2 displays the signed difference between transformed and original correlations so that the horizontal line at an ordinate of zero indicates no change in correlation after the transformations. Although all combinations of transformations alter the original correlations, the change is certainly very small. In addition, *r*_1*Y*_ and *r*_2*Y*_ remain identical (to within sampling error) as long as *X*_1_ and *X*_2_ are identically transformed. This warrants a direct comparison of the performance of the tests with and without violation of normality under otherwise nearly identical conditions.
