# Supplementary material for: Are alternative variables in a set differently associated with a target variable? Statistical tests and practical advice for dealing with dependent correlations
Source: Br J Math Stat Psychol. 2024 Jun 24;78(1):112–40. doi: 10.1111/bmsp.12354 (PMC11701387; doi:10.1111/bmsp.12354)

Are alternative variables in a set differently associated with a target variable? Statistical tests and practical advice for dealing with dependent correlations

British Journal of Mathematical and Statistical Psychology
https://doi.org/10.1111/bmsp.12354

Miguel A. García-Pérez

Supplemental Material

Section A: Type-I error rate of each test with normal data
Section B: Power of each test with normal data

Section C: Type-I error rate of each test with uniform data
Section D: Power of each test with uniform data

Section E: Type-I error rate of each test with Beta(2, 5) data
Section F: Power of each test with Beta(2, 5) data

Section G: Type-I error rate of each test with Lognormal(0, 1) data
Section H: Type-I error rate of each test with mixture 0.9 N(0, 1) + 0.1 N(0, 2) data
Section I: Type-I error rate of each test with mixture 0.9 N(0, 1) + 0.1 N(0, 4) data
Section J: Type-I error rate of each test with mixture 0.9 N(0, 1) + 0.1 N(0, 10) data

Section A: Type-I error rates of each test with normal data (sample size top to bottom: 20, 50, 100, 200)

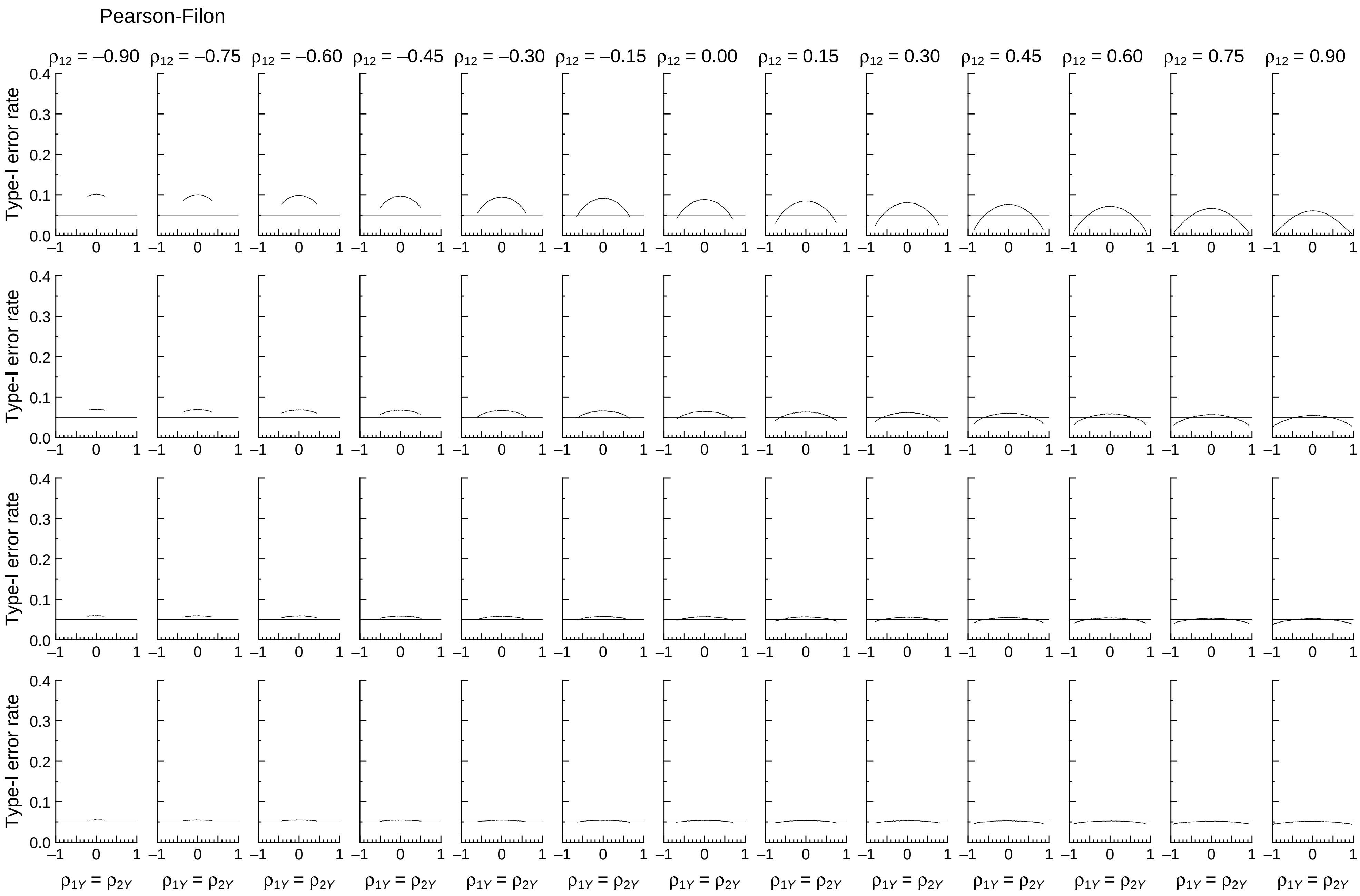

Section A: Type-I error rates of each test with normal data (sample size top to bottom: 20, 50, 100, 200)

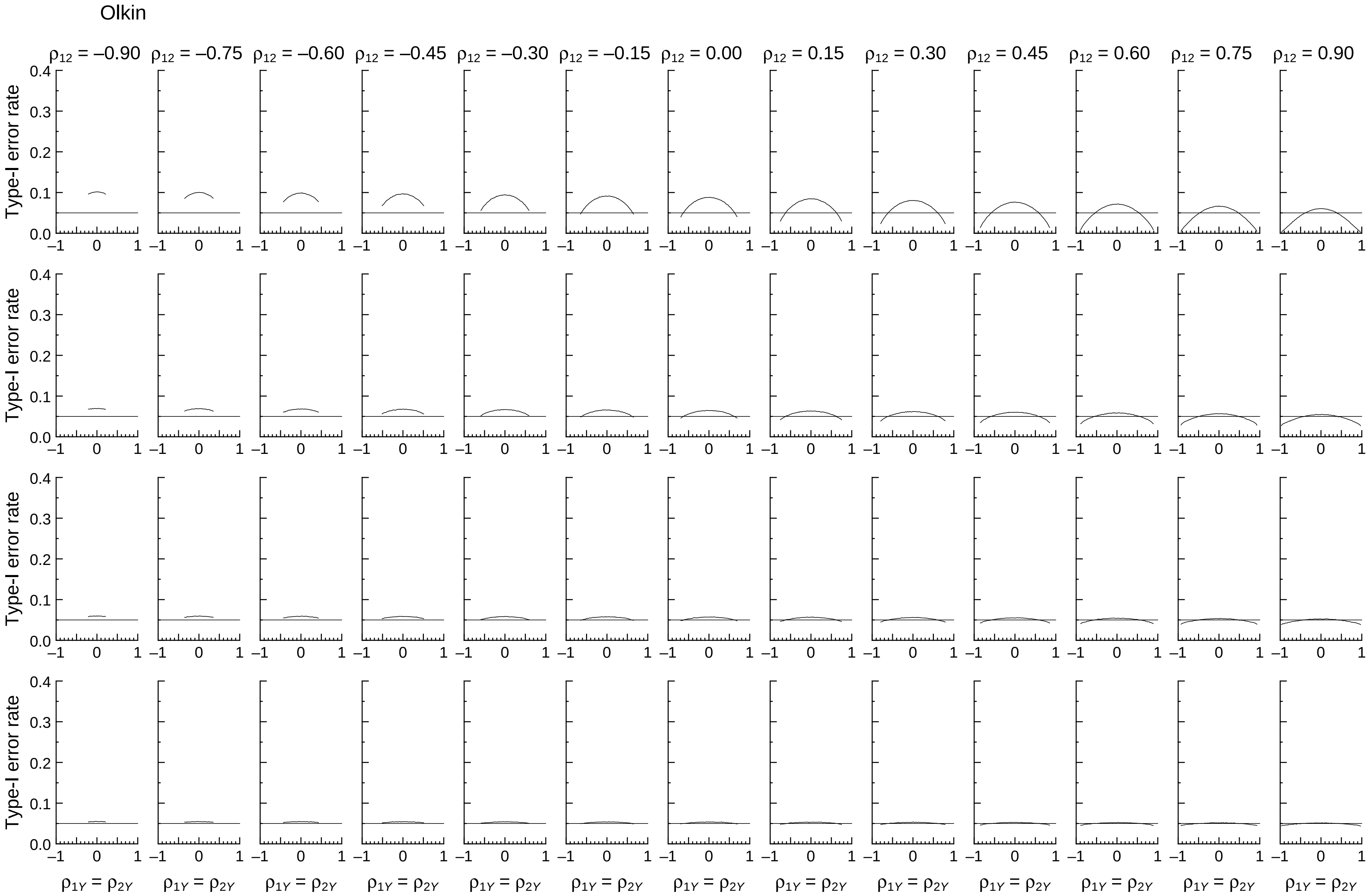

Section A: Type-I error rates of each test with normal data (sample size top to bottom: 20, 50, 100, 200)

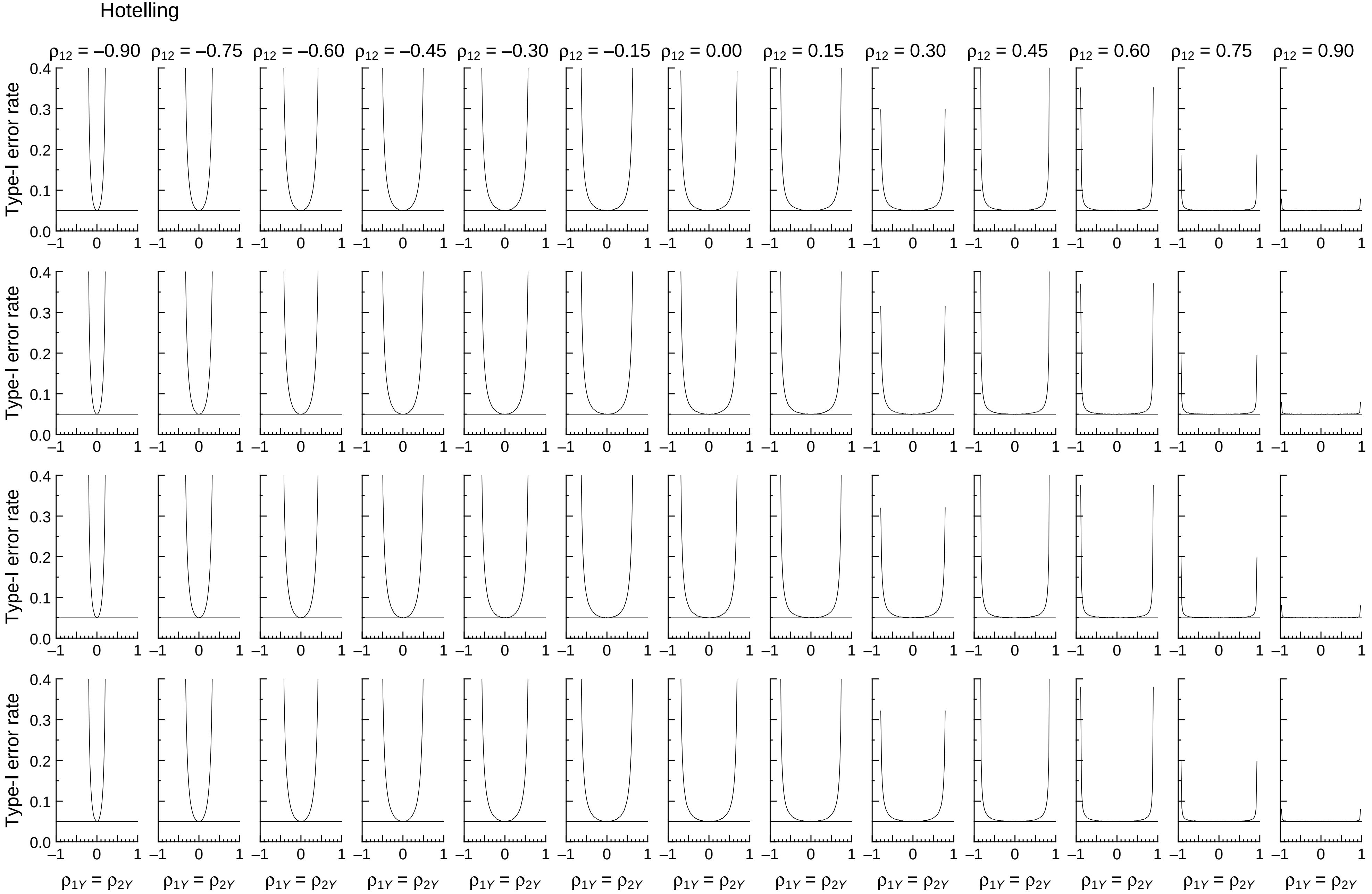

Section A: Type-I error rates of each test with normal data (sample size top to bottom: 20, 50, 100, 200)

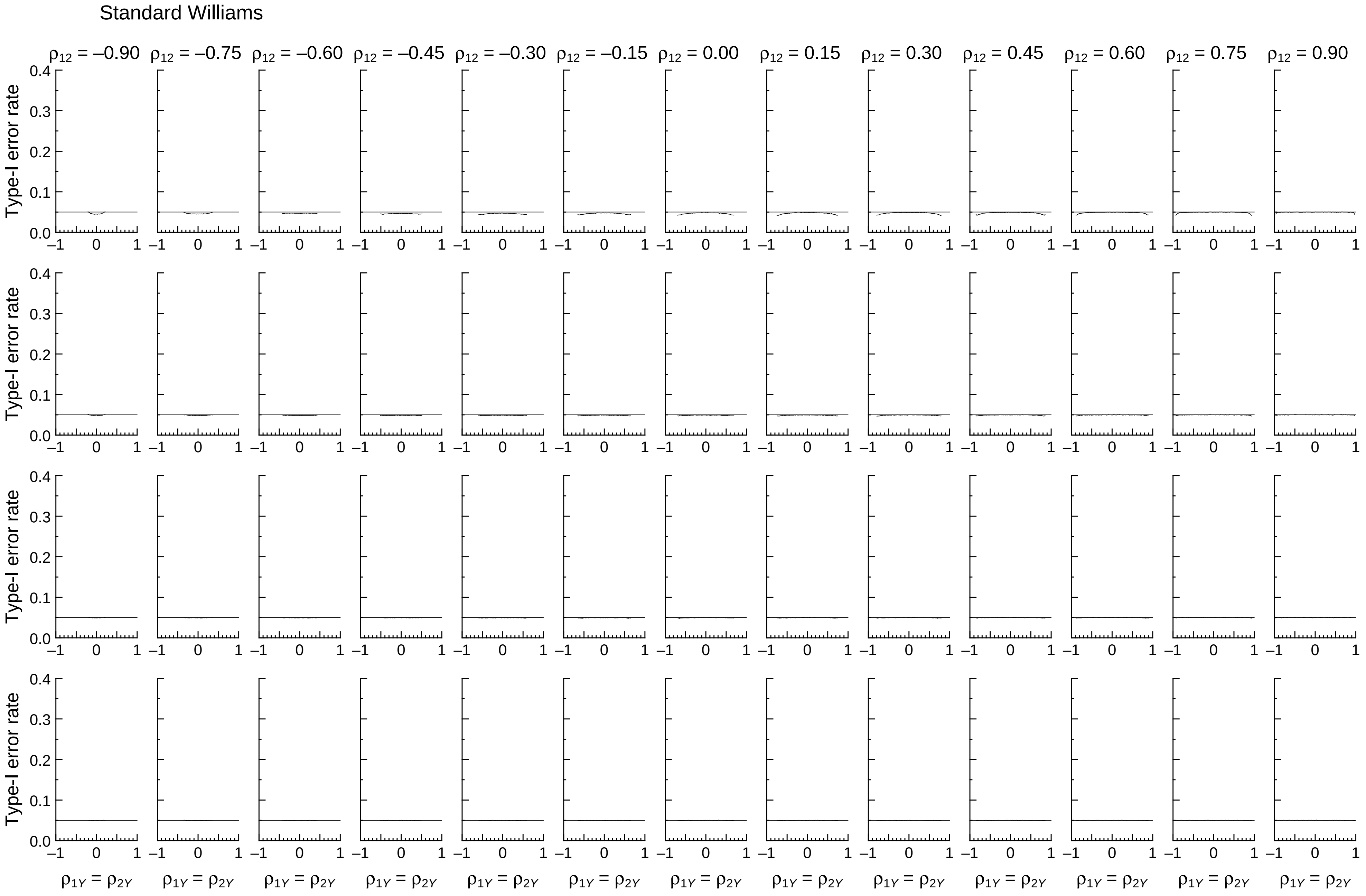

Section A: Type-I error rates of each test with normal data (sample size top to bottom: 20, 50, 100, 200)

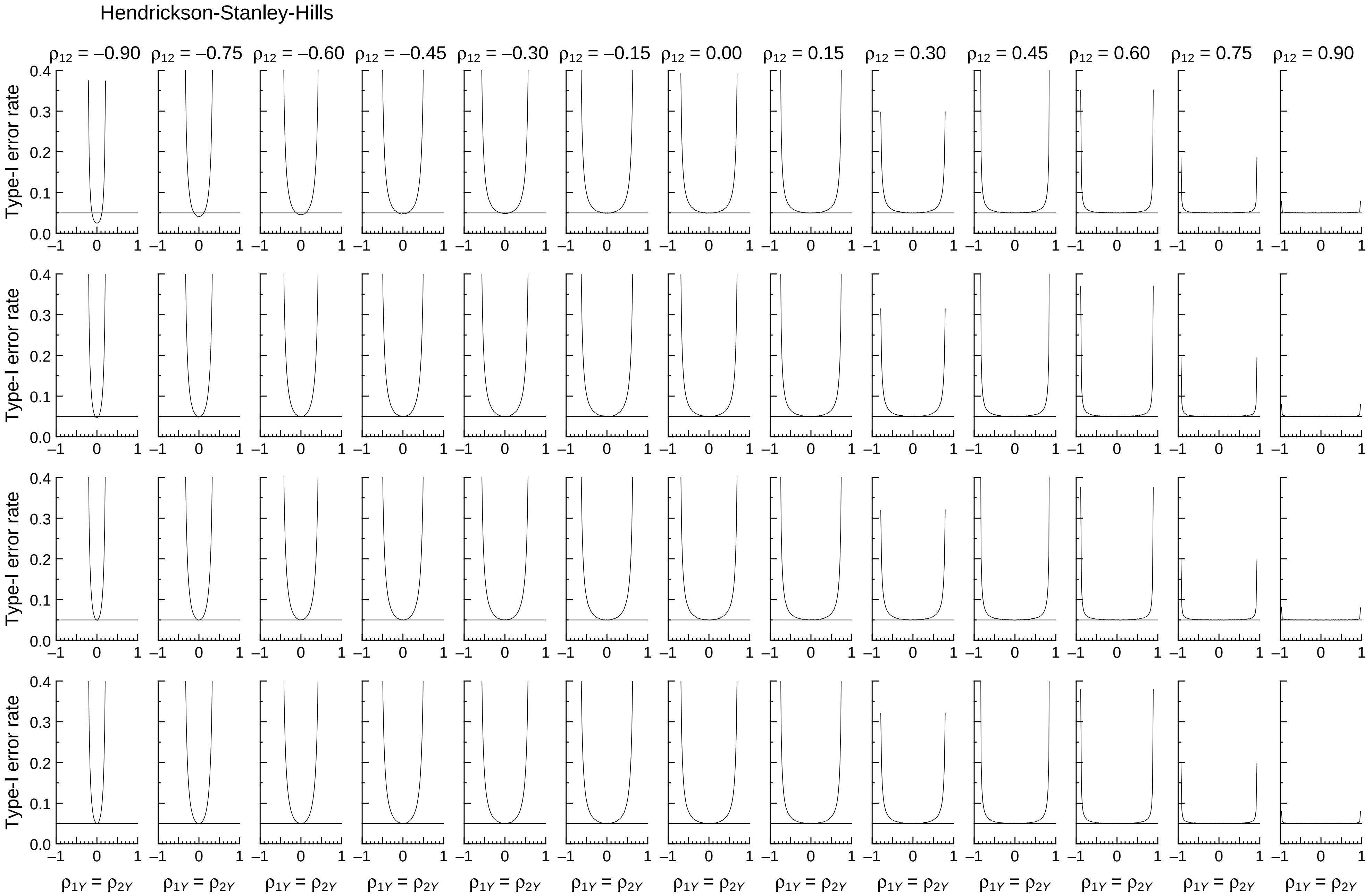

Section A: Type-I error rates of each test with normal data (sample size top to bottom: 20, 50, 100, 200)

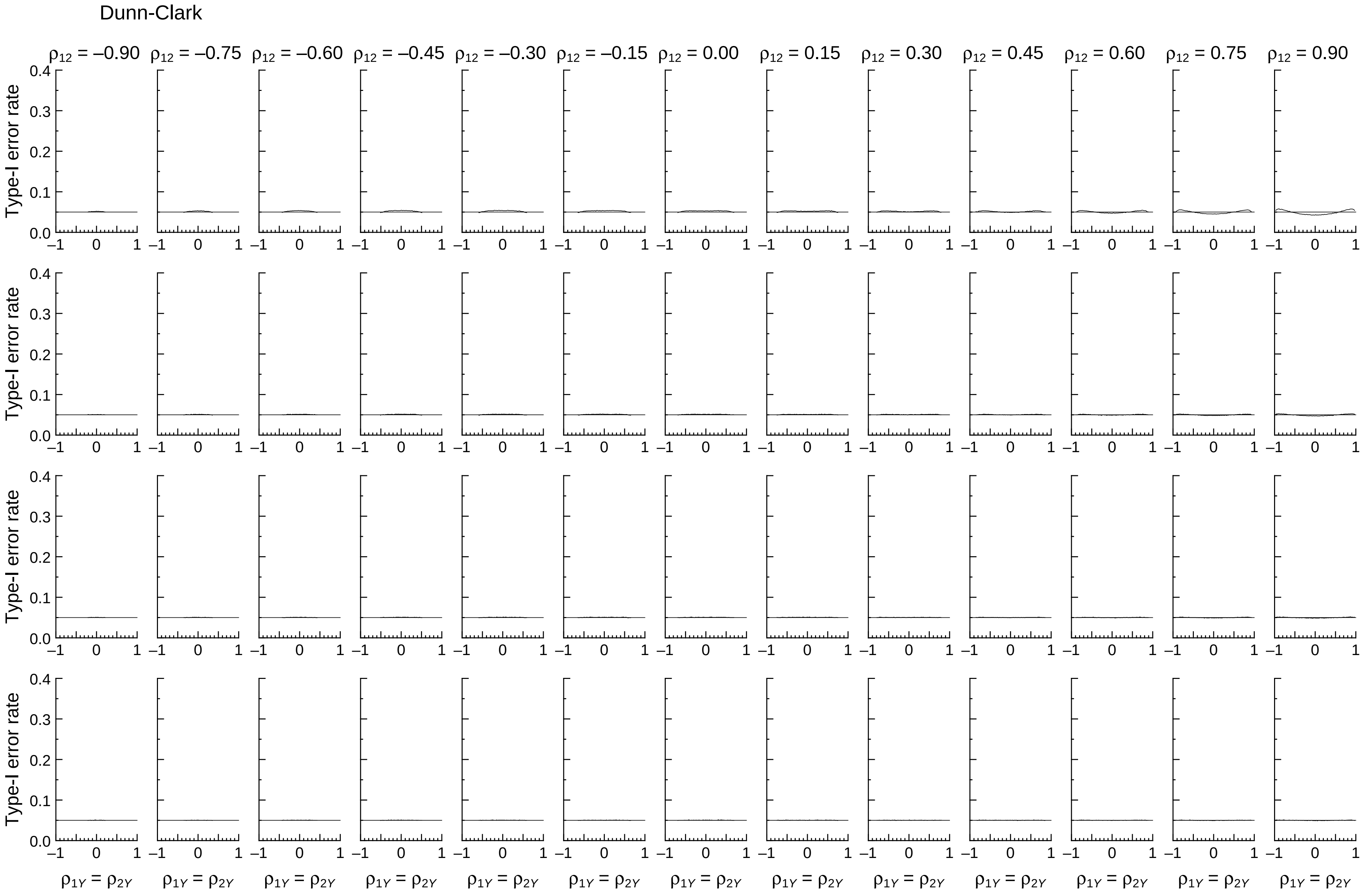

Section A: Type-I error rates of each test with normal data (sample size top to bottom: 20, 50, 100, 200)

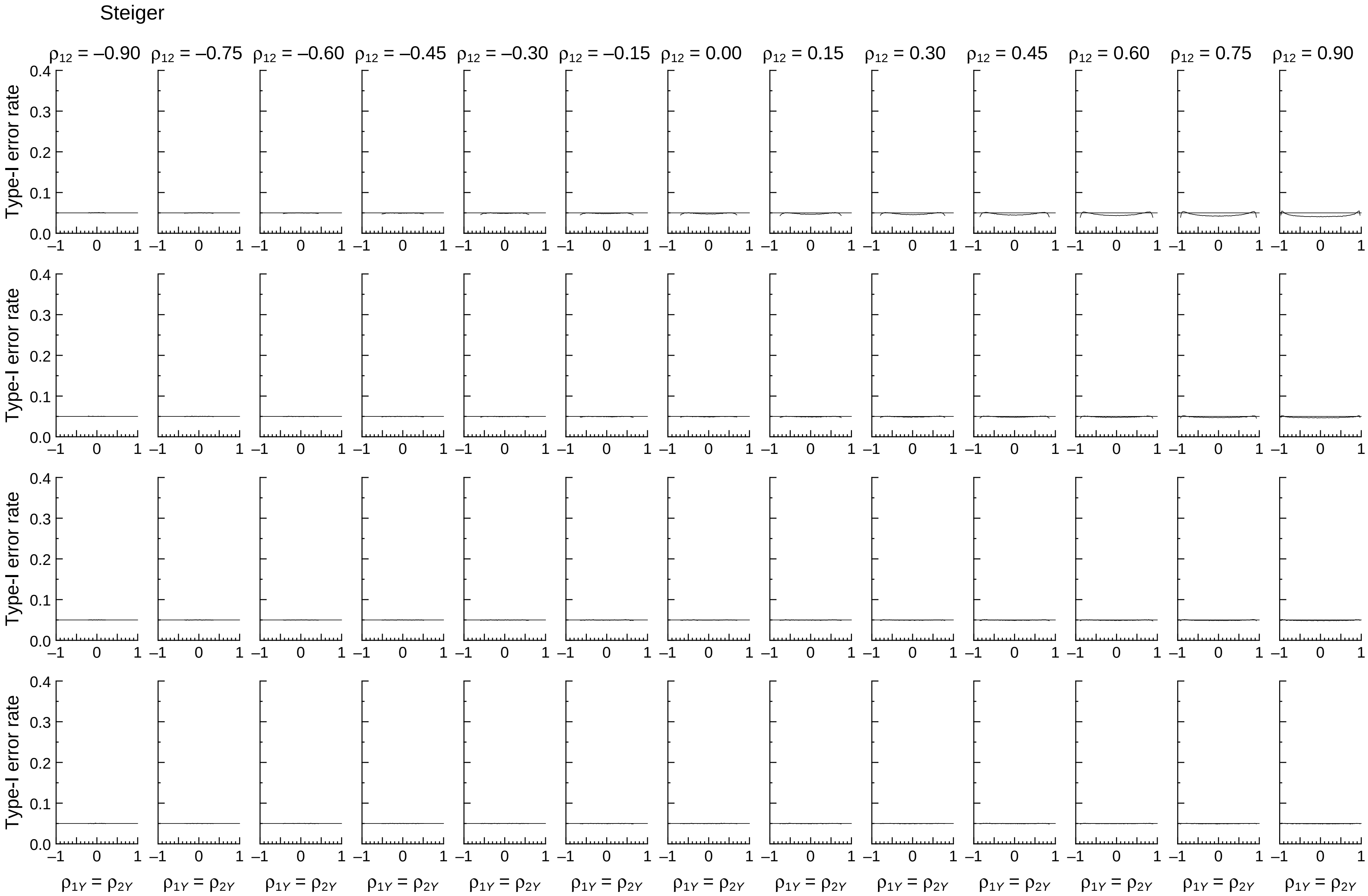

Section A: Type-I error rates of each test with normal data (sample size top to bottom: 20, 50, 100, 200)

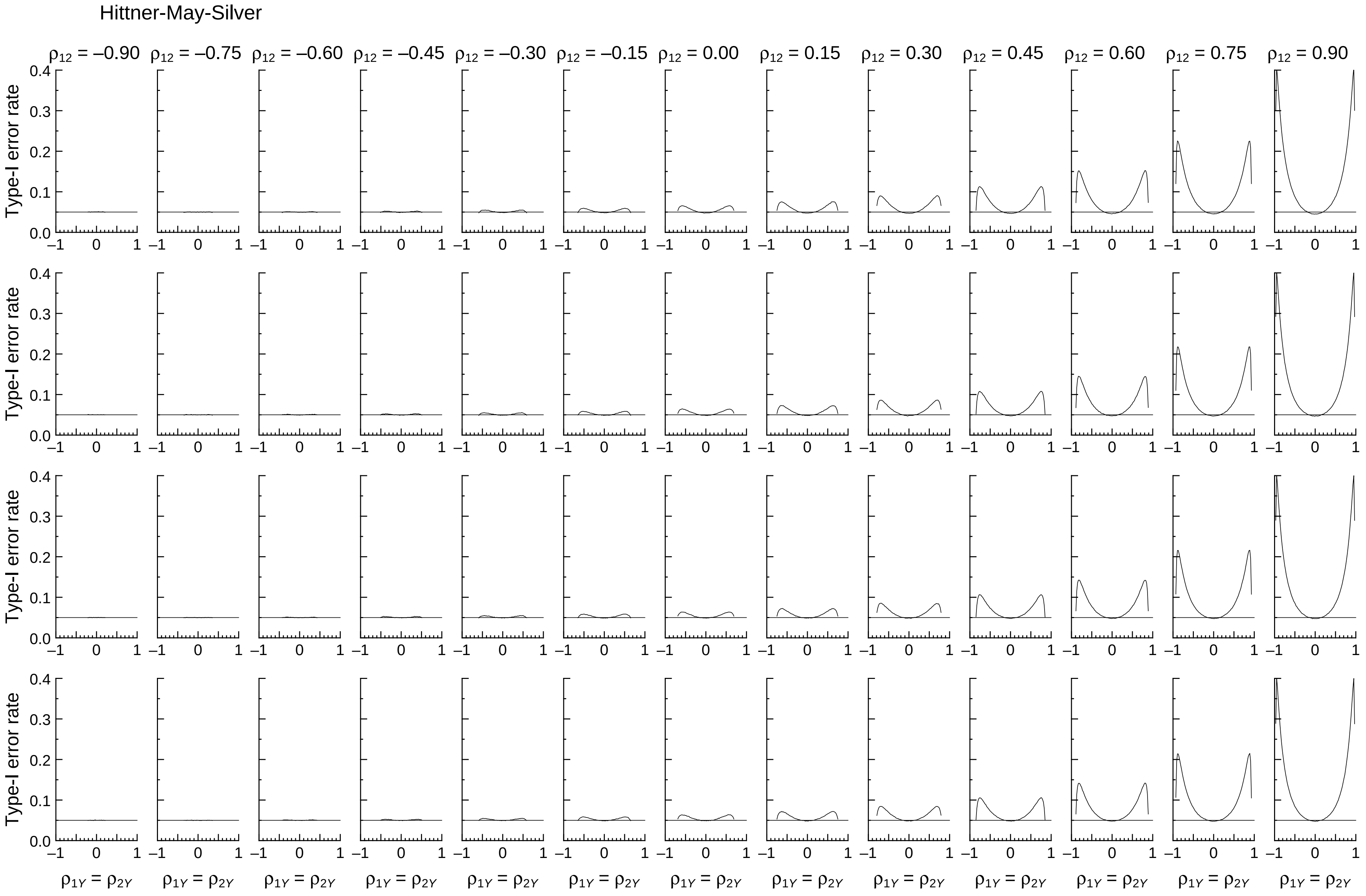

Section A: Type-I error rates of each test with normal data (sample size top to bottom: 20, 50, 100, 200)

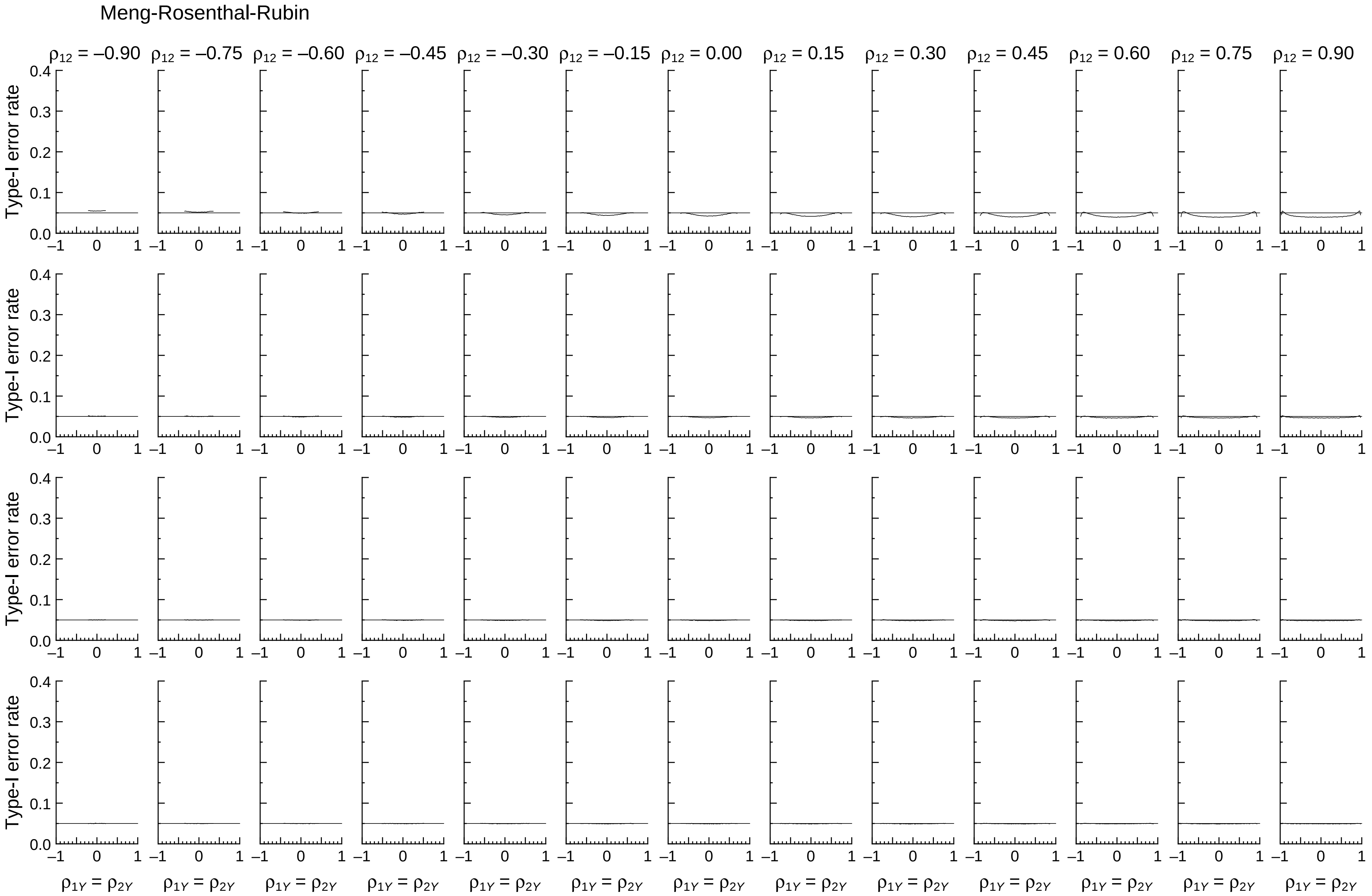

Section A: Type-I error rates of each test with normal data (sample size top to bottom: 20, 50, 100, 200)

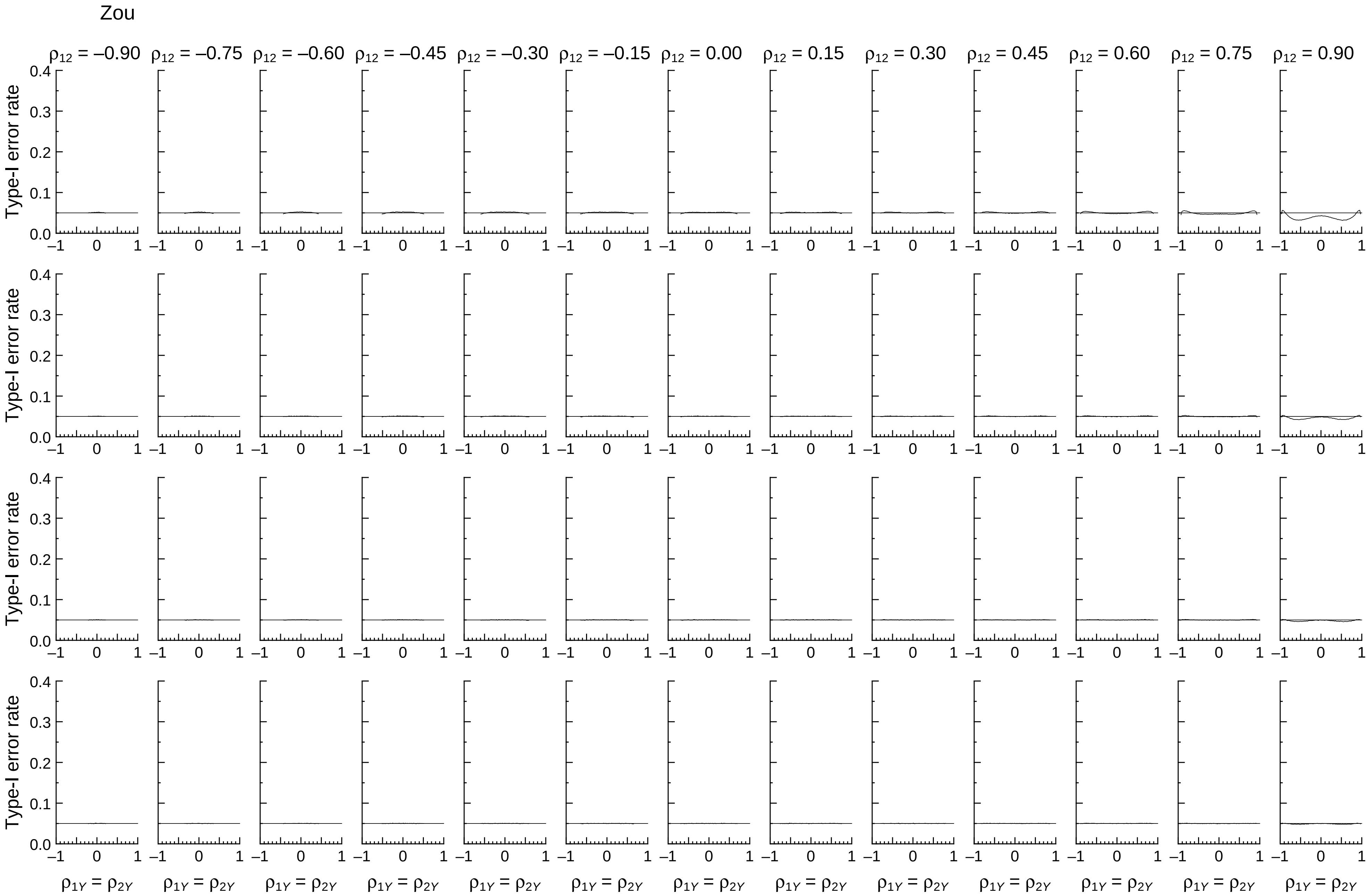

Section B: Power of each test with normal data (sample size top to bottom: 20, 50, 100, 200)

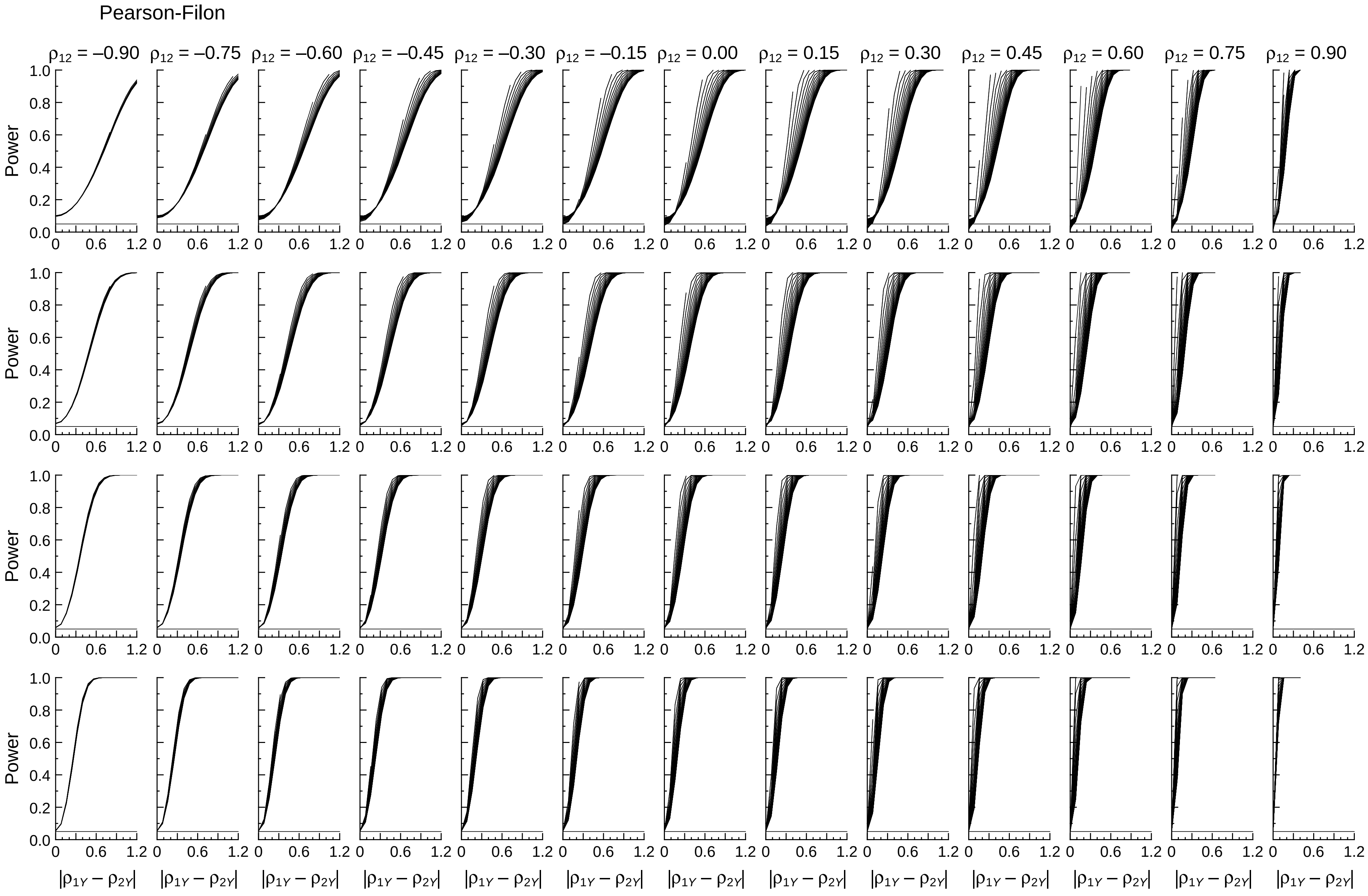

Section B: Power of each test with normal data (sample size top to bottom: 20, 50, 100, 200)

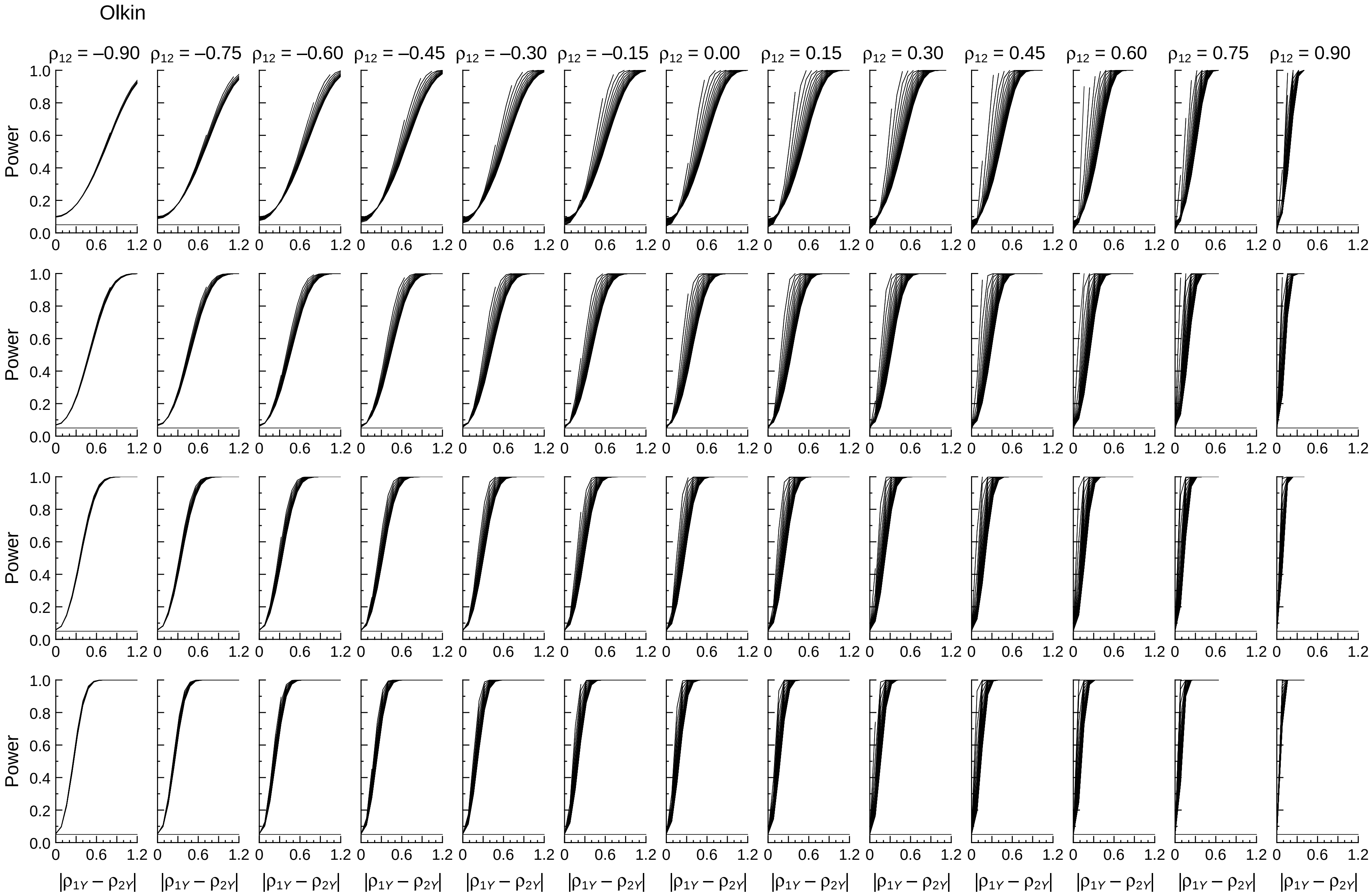

Section B: Power of each test with normal data (sample size top to bottom: 20, 50, 100, 200)

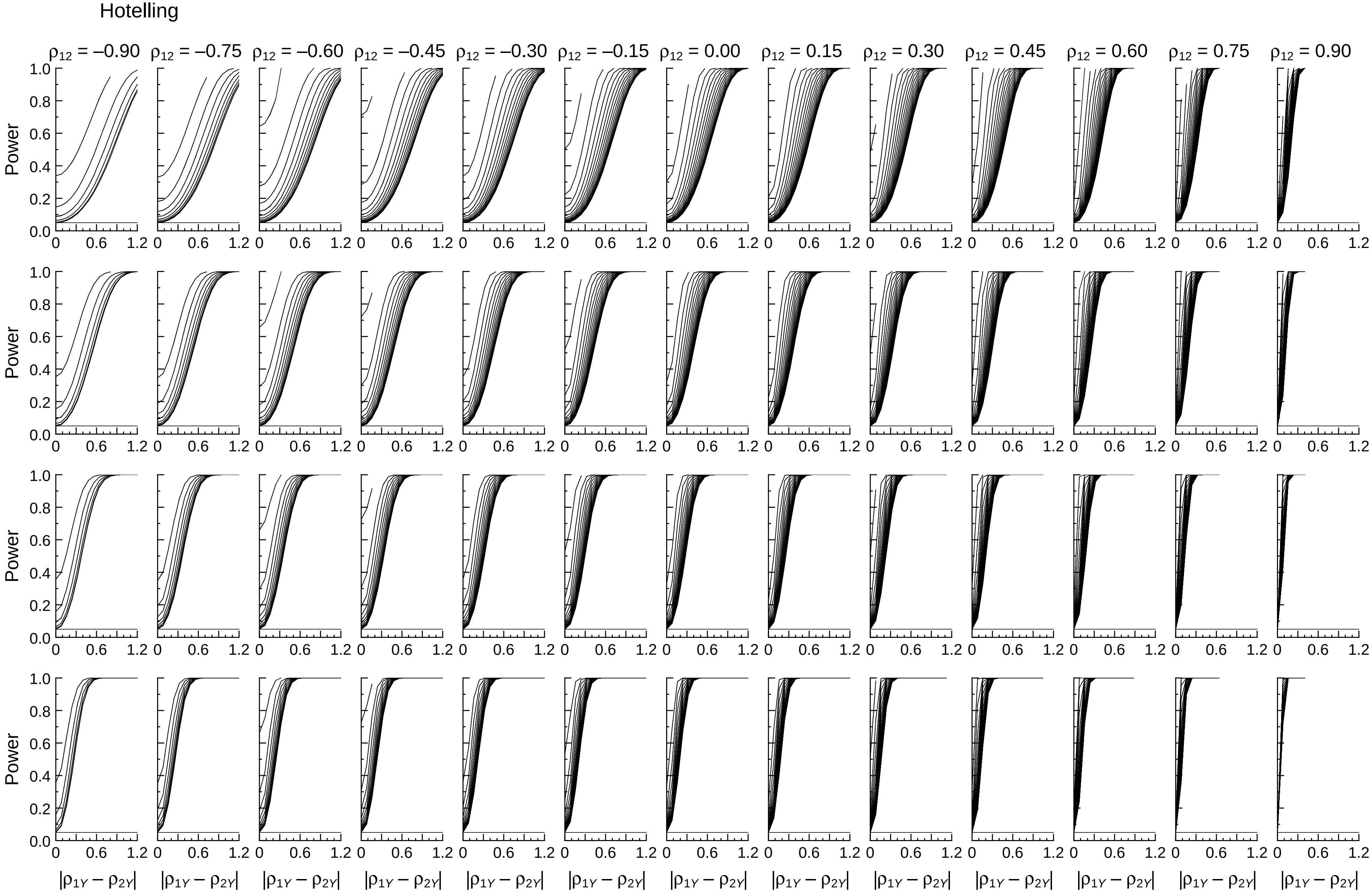

Section B: Power of each test with normal data (sample size top to bottom: 20, 50, 100, 200)

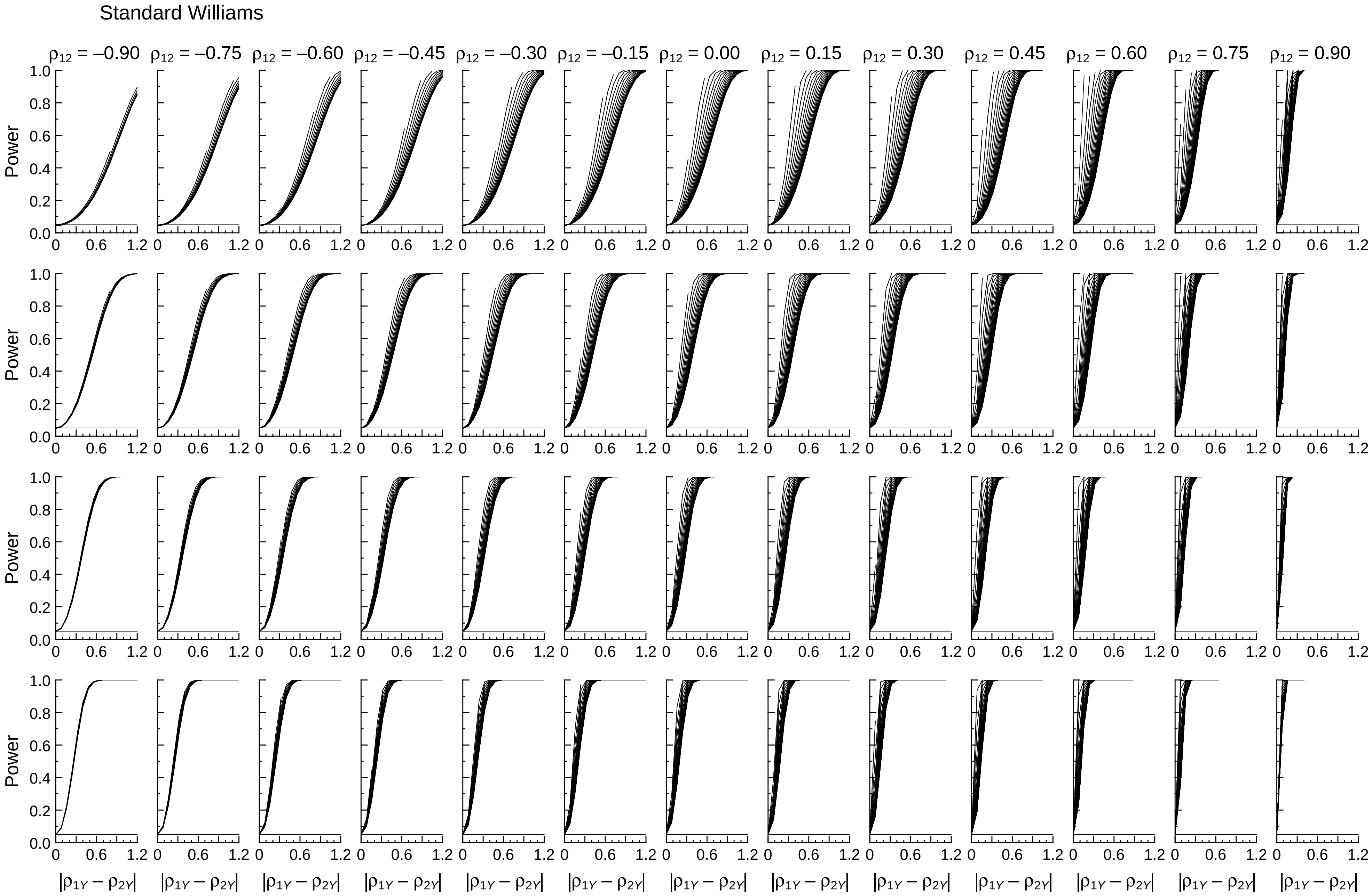

Section B: Power of each test with normal data (sample size top to bottom: 20, 50, 100, 200)

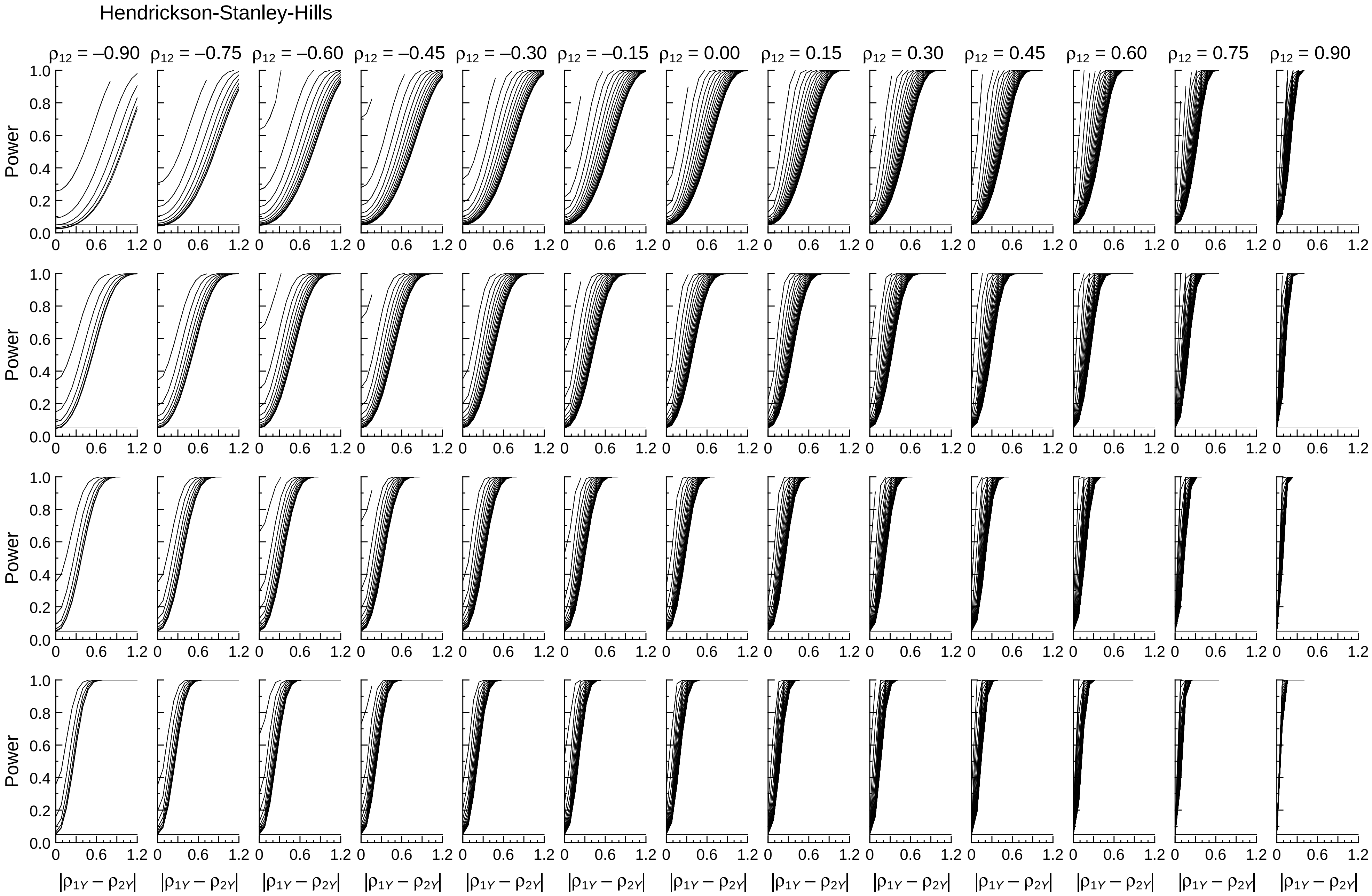

Section B: Power of each test with normal data (sample size top to bottom: 20, 50, 100, 200)

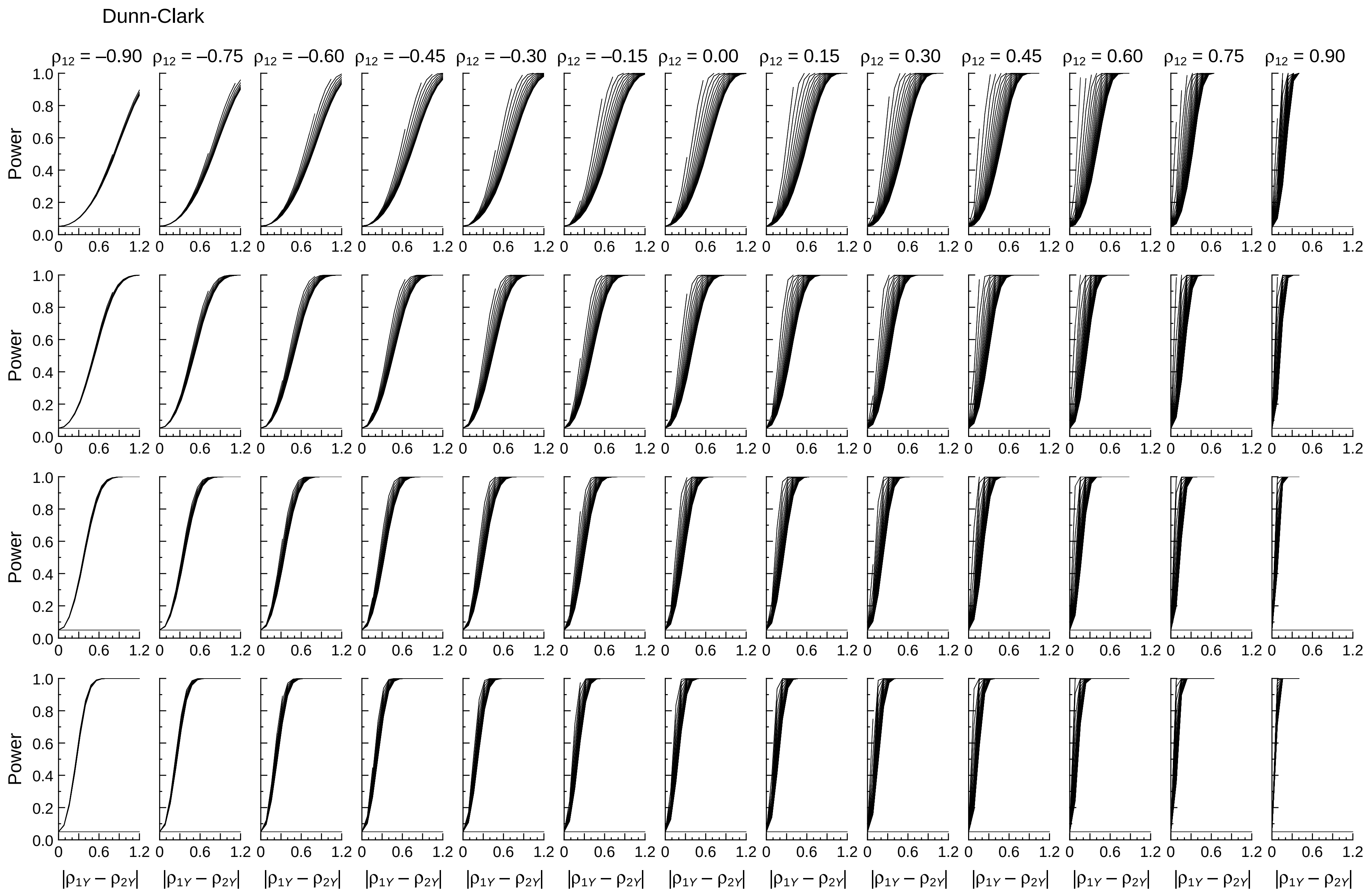

Section B: Power of each test with normal data (sample size top to bottom: 20, 50, 100, 200)

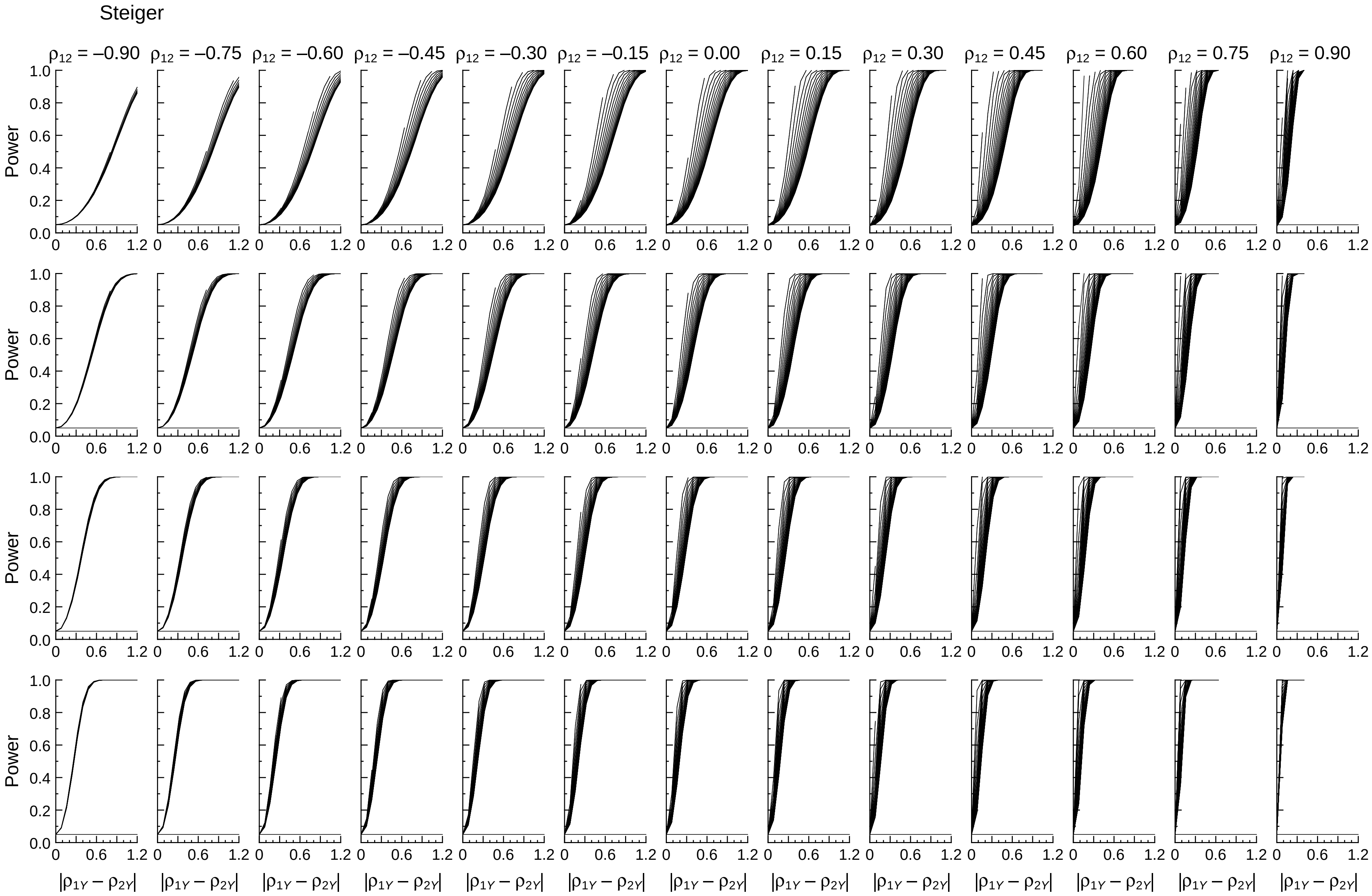

Section B: Power of each test with normal data (sample size top to bottom: 20, 50, 100, 200)

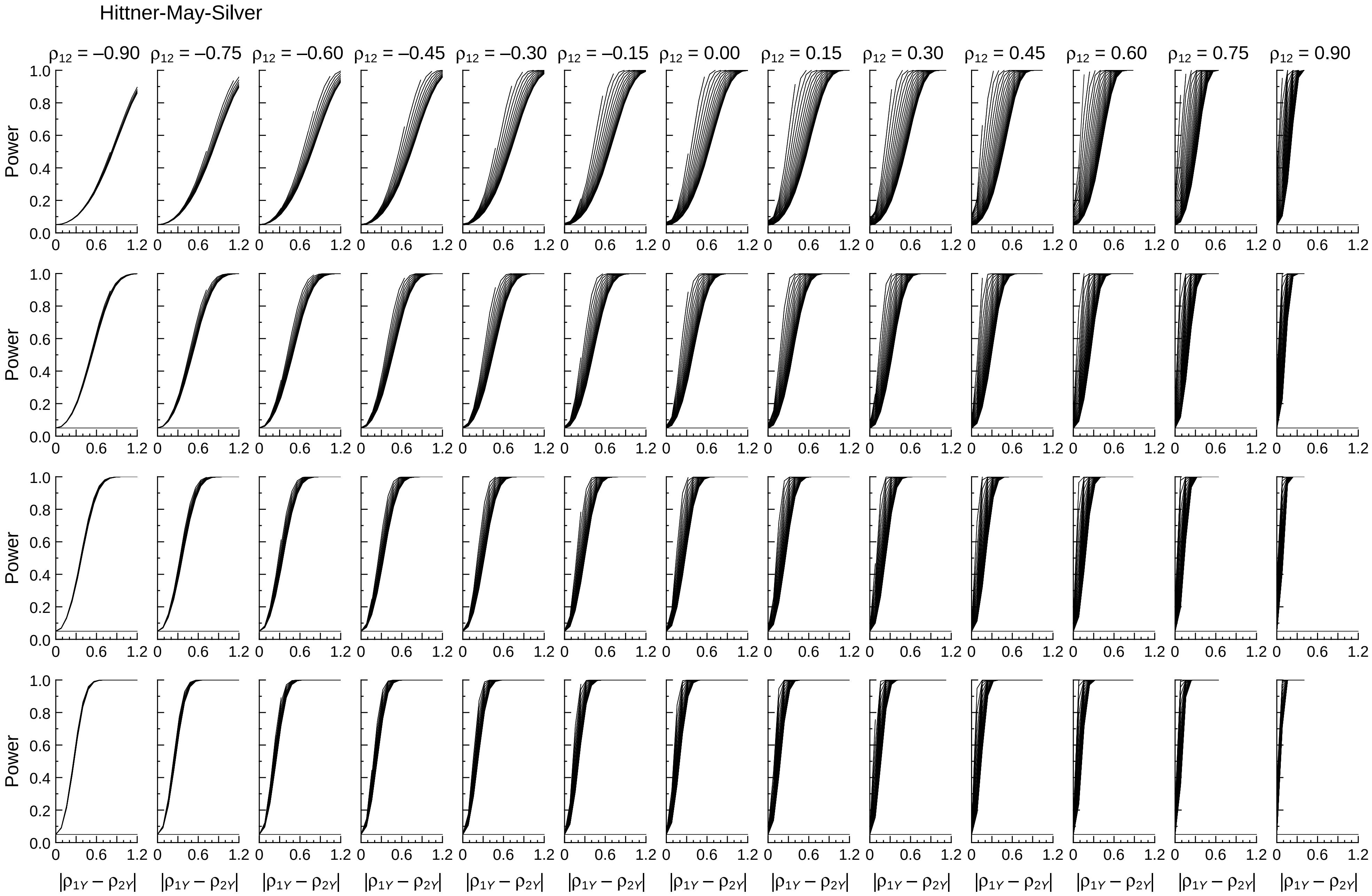

Section B: Power of each test with normal data (sample size top to bottom: 20, 50, 100, 200)

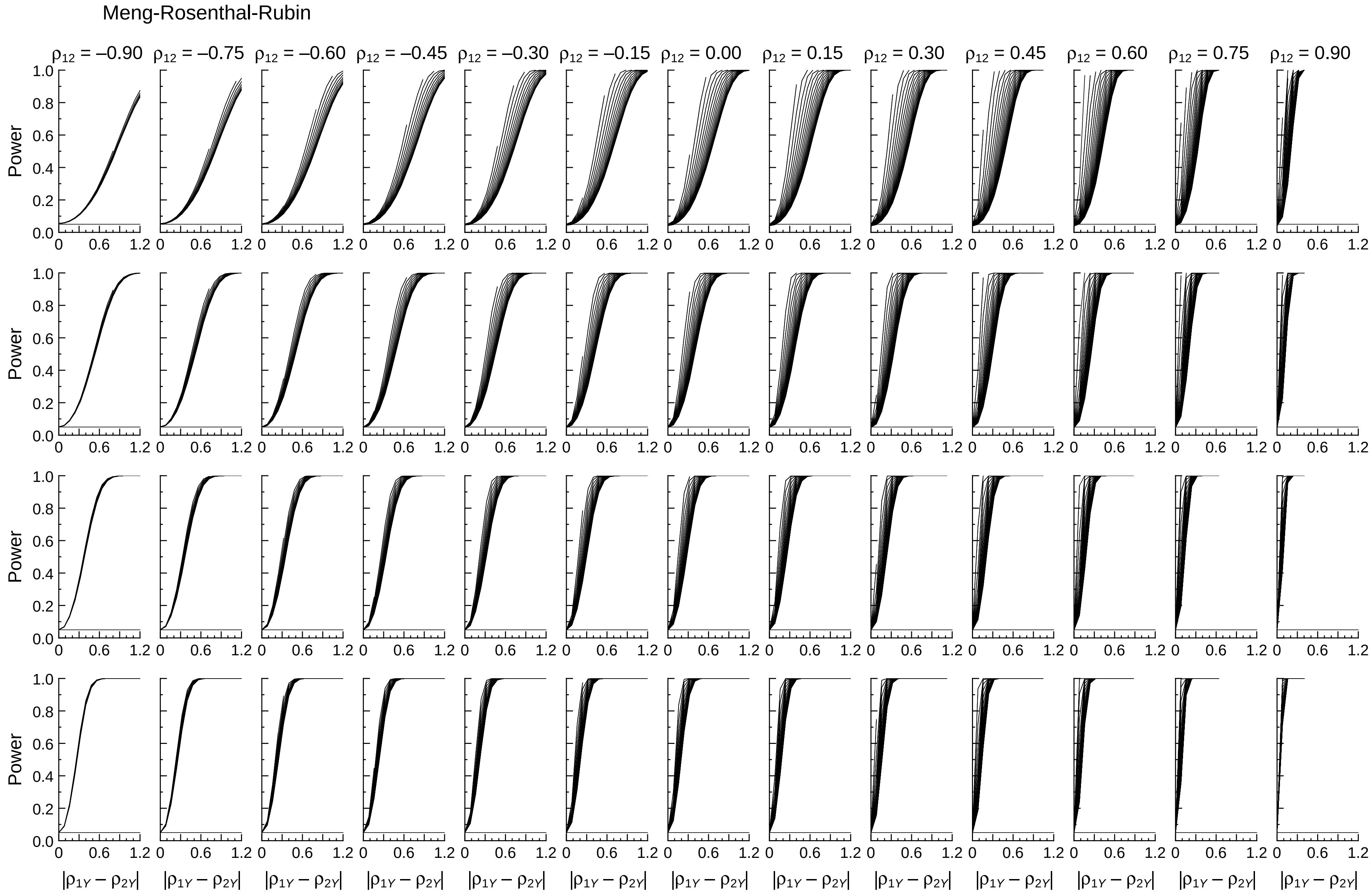

Section B: Power of each test with normal data (sample size top to bottom: 20, 50, 100, 200)

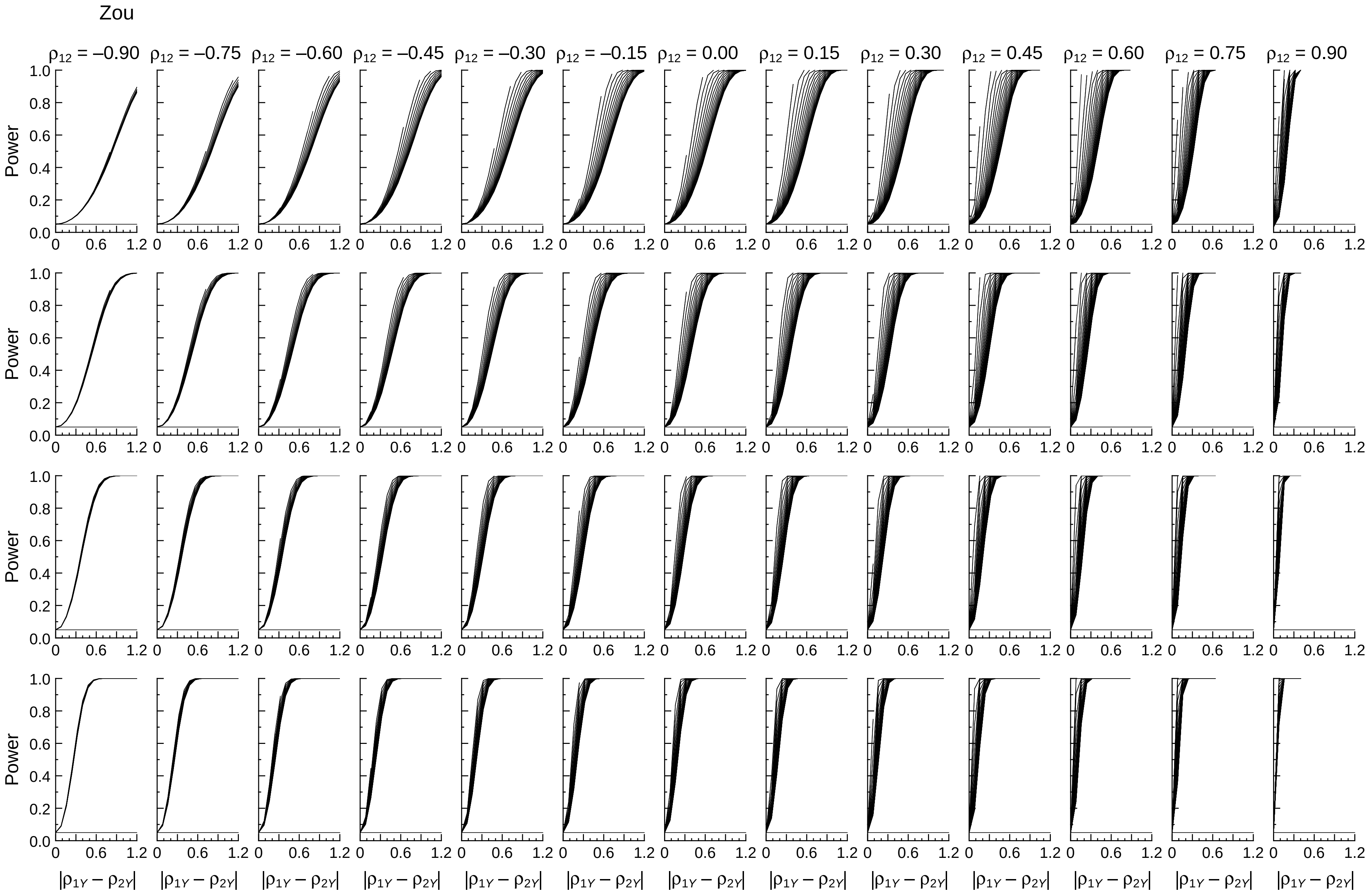

Section C: Type-I error rates of each test with uniform data (sample size top to bottom: 20, 50, 100, 200)

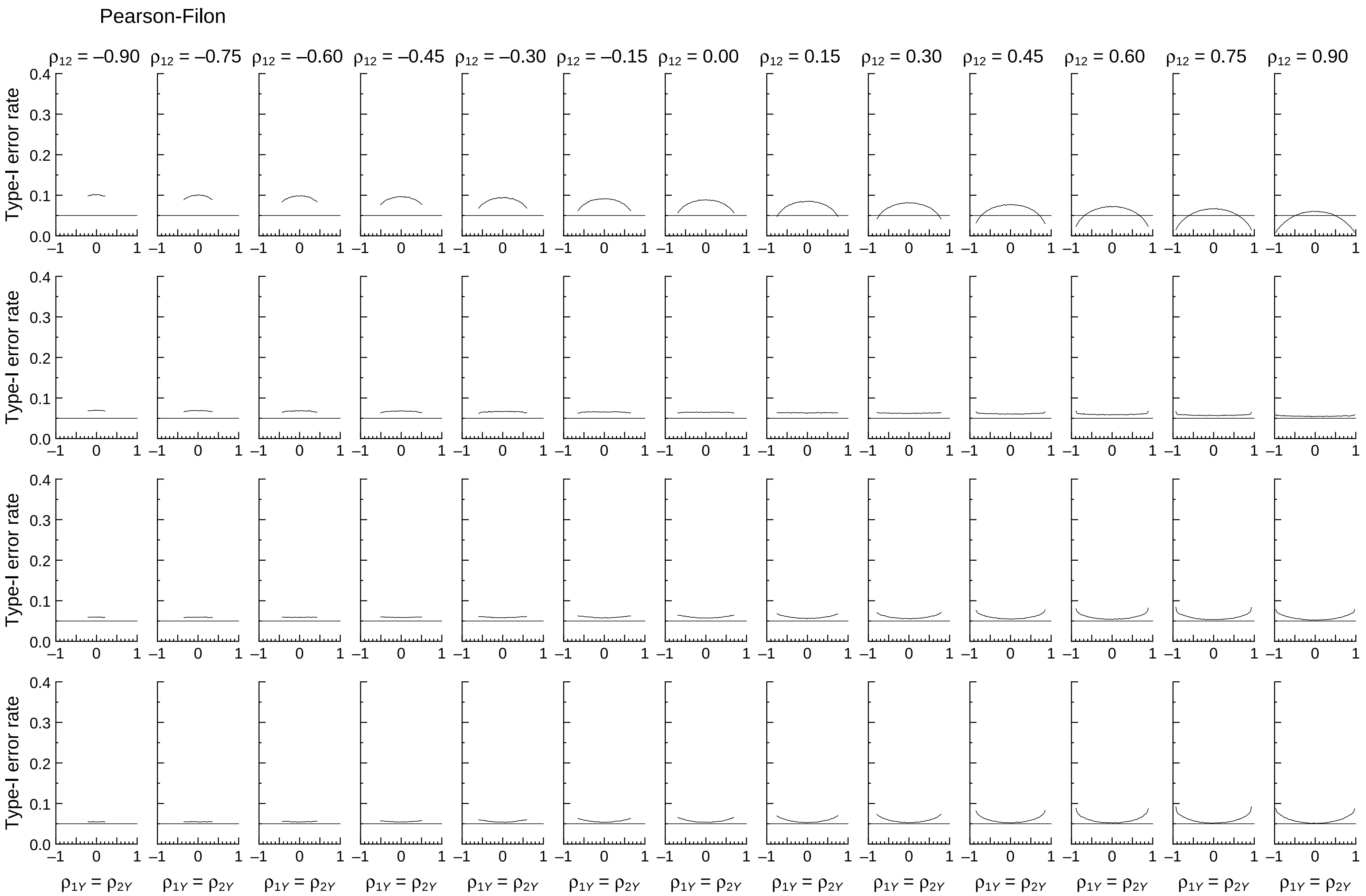

Section C: Type-I error rates of each test with uniform data (sample size top to bottom: 20, 50, 100, 200)

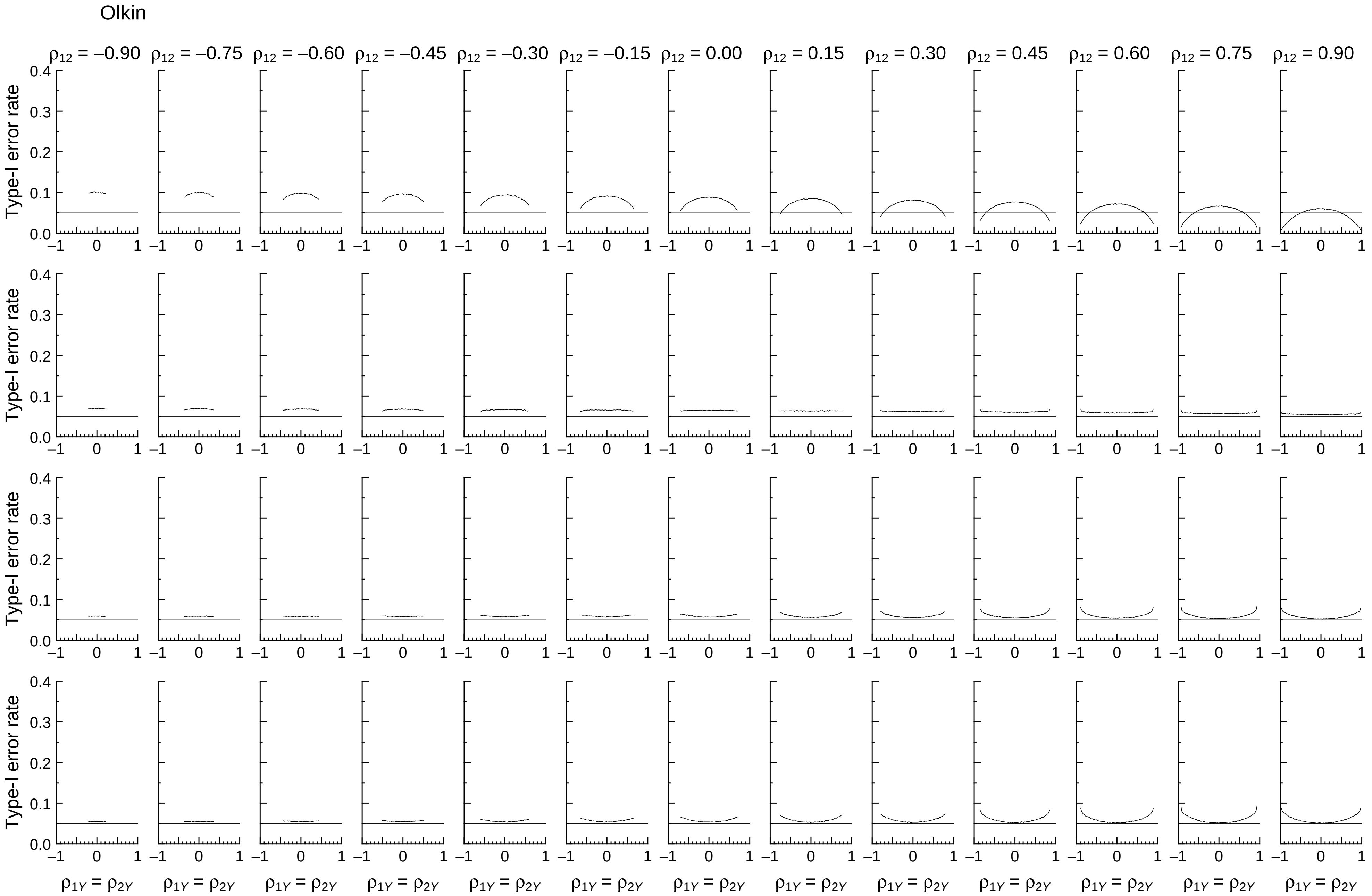

Section C: Type-I error rates of each test with uniform data (sample size top to bottom: 20, 50, 100, 200)

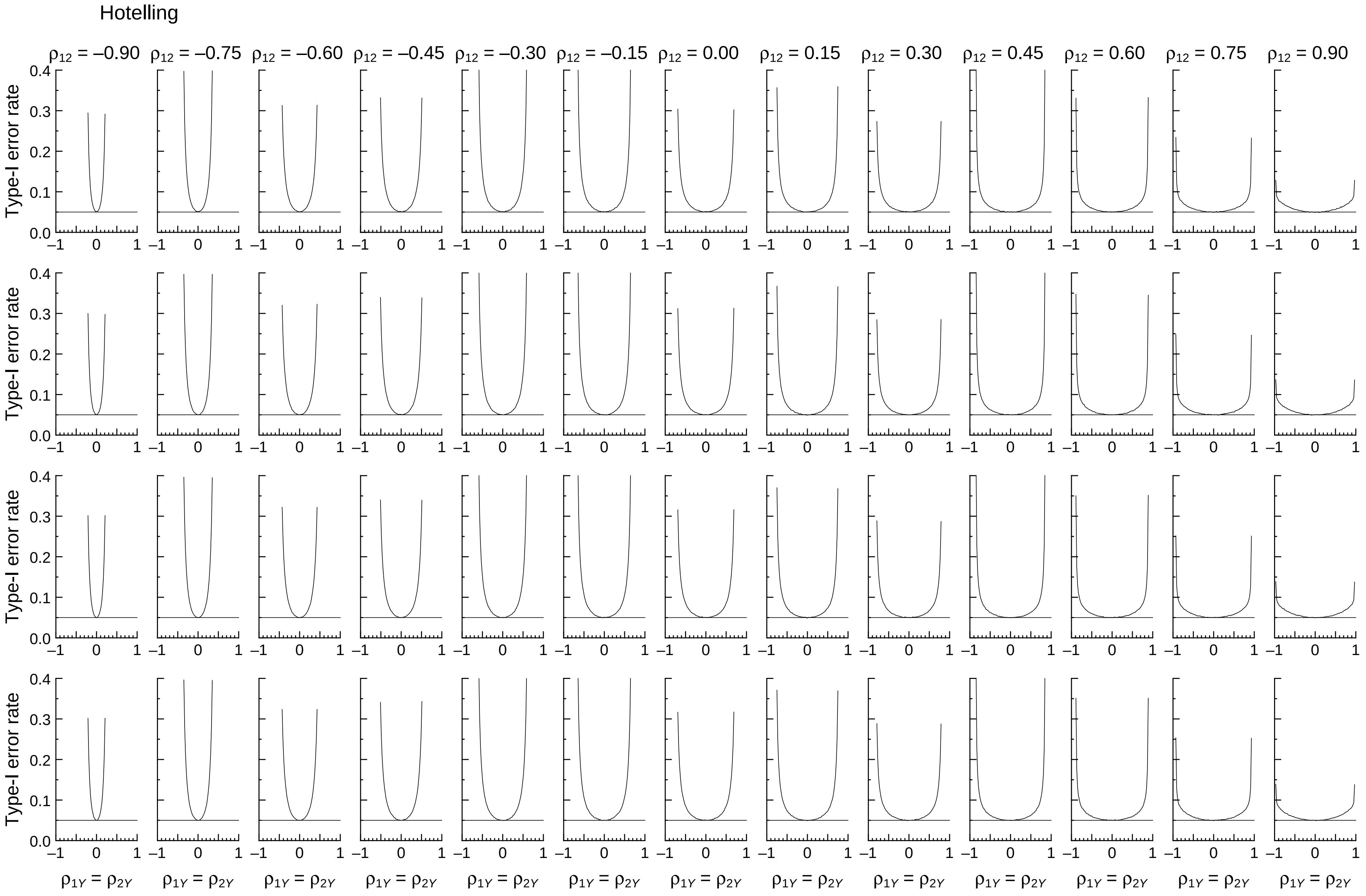

Section C: Type-I error rates of each test with uniform data (sample size top to bottom: 20, 50, 100, 200)

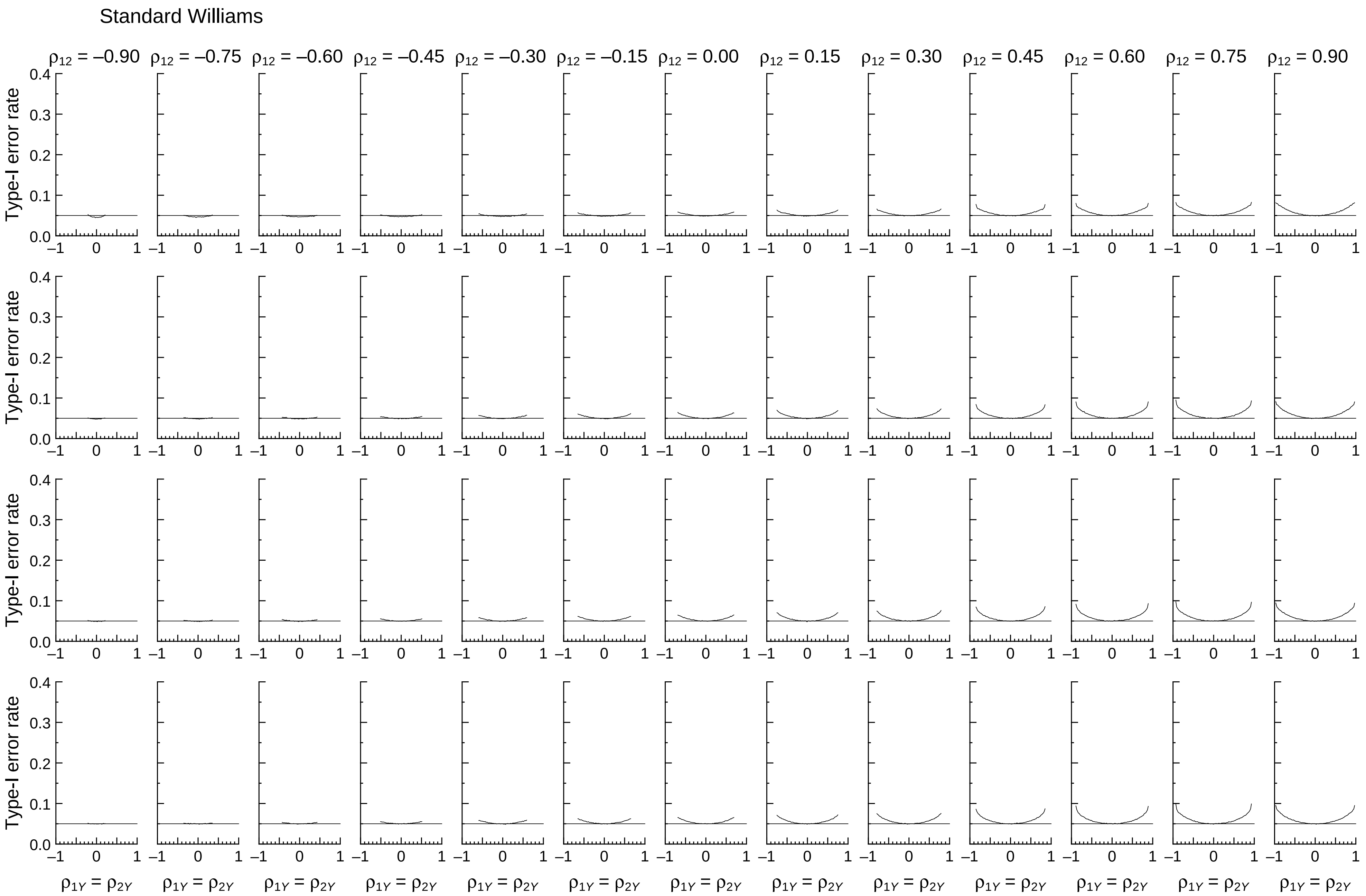

Section C: Type-I error rates of each test with uniform data (sample size top to bottom: 20, 50, 100, 200)

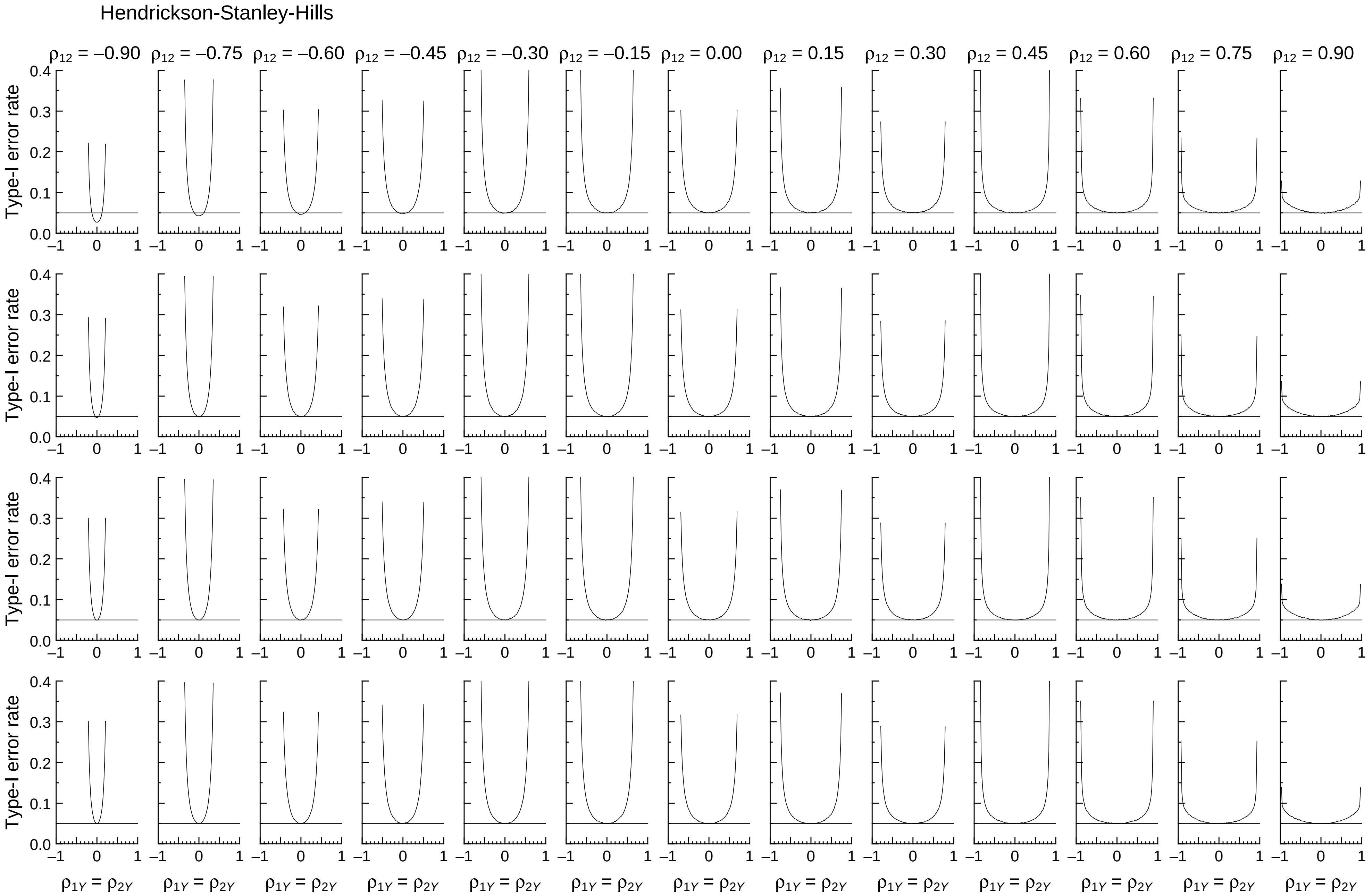

Section C: Type-I error rates of each test with uniform data (sample size top to bottom: 20, 50, 100, 200)

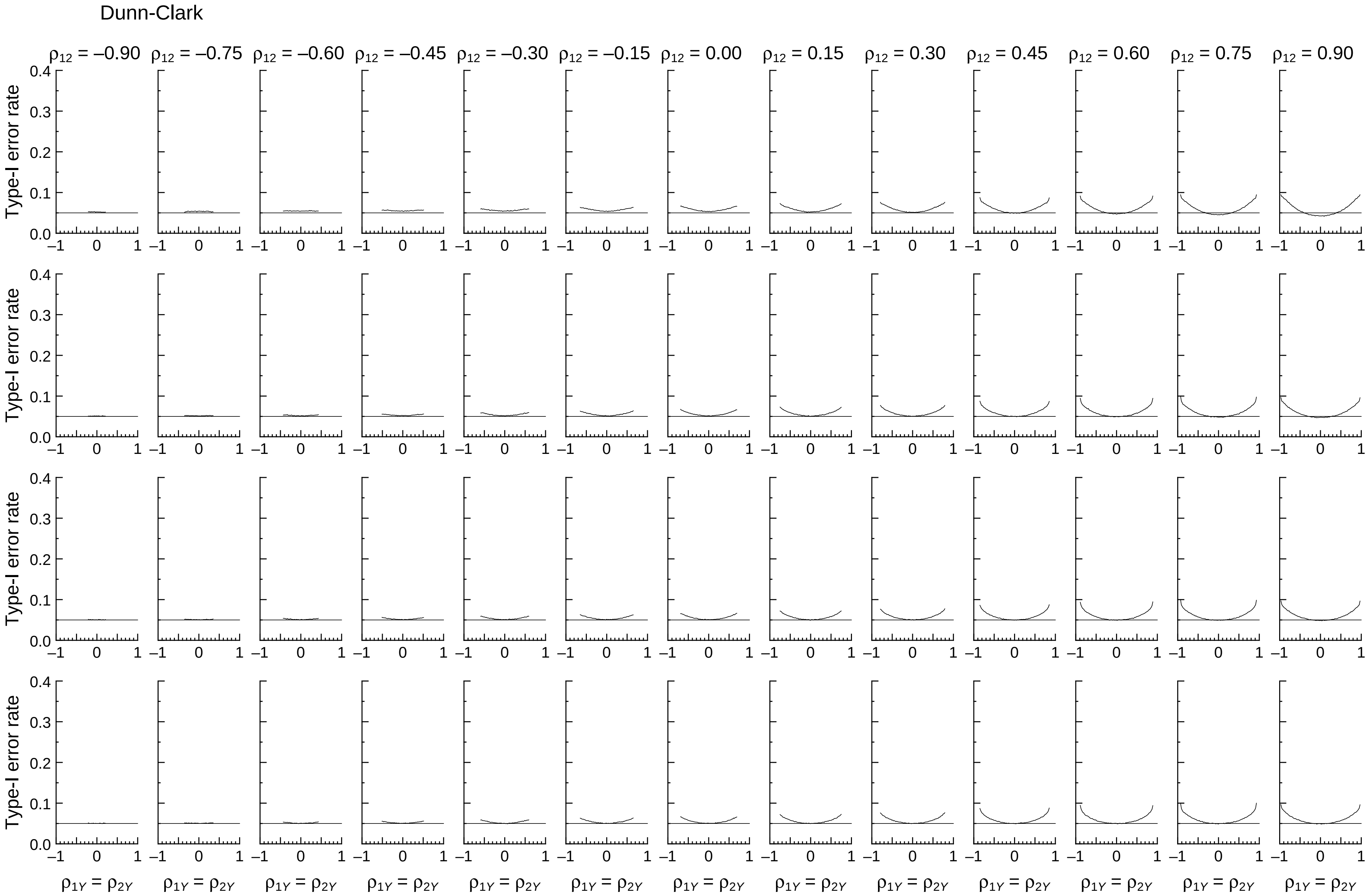

Section C: Type-I error rates of each test with uniform data (sample size top to bottom: 20, 50, 100, 200)

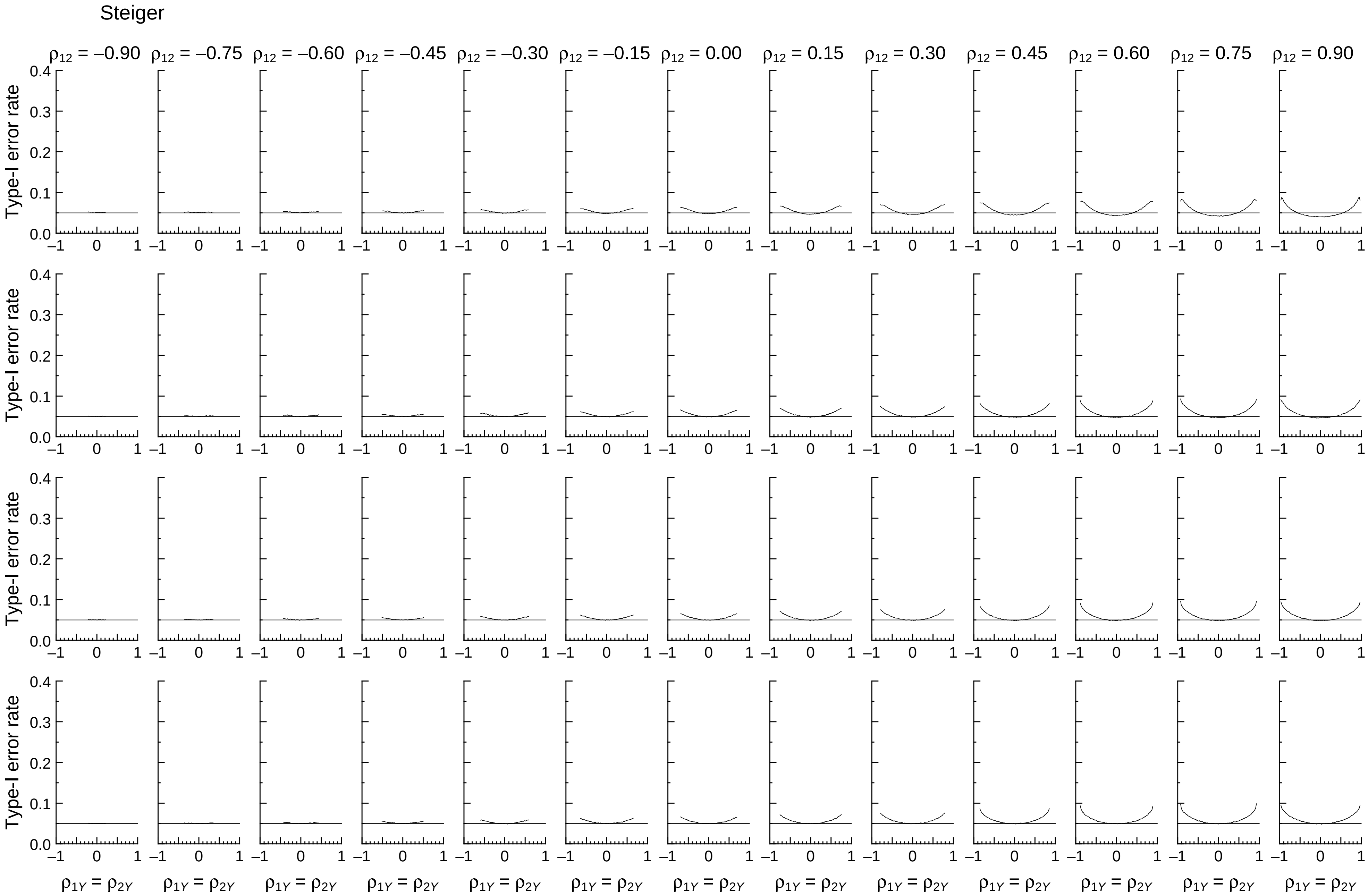

Section C: Type-I error rates of each test with uniform data (sample size top to bottom: 20, 50, 100, 200)

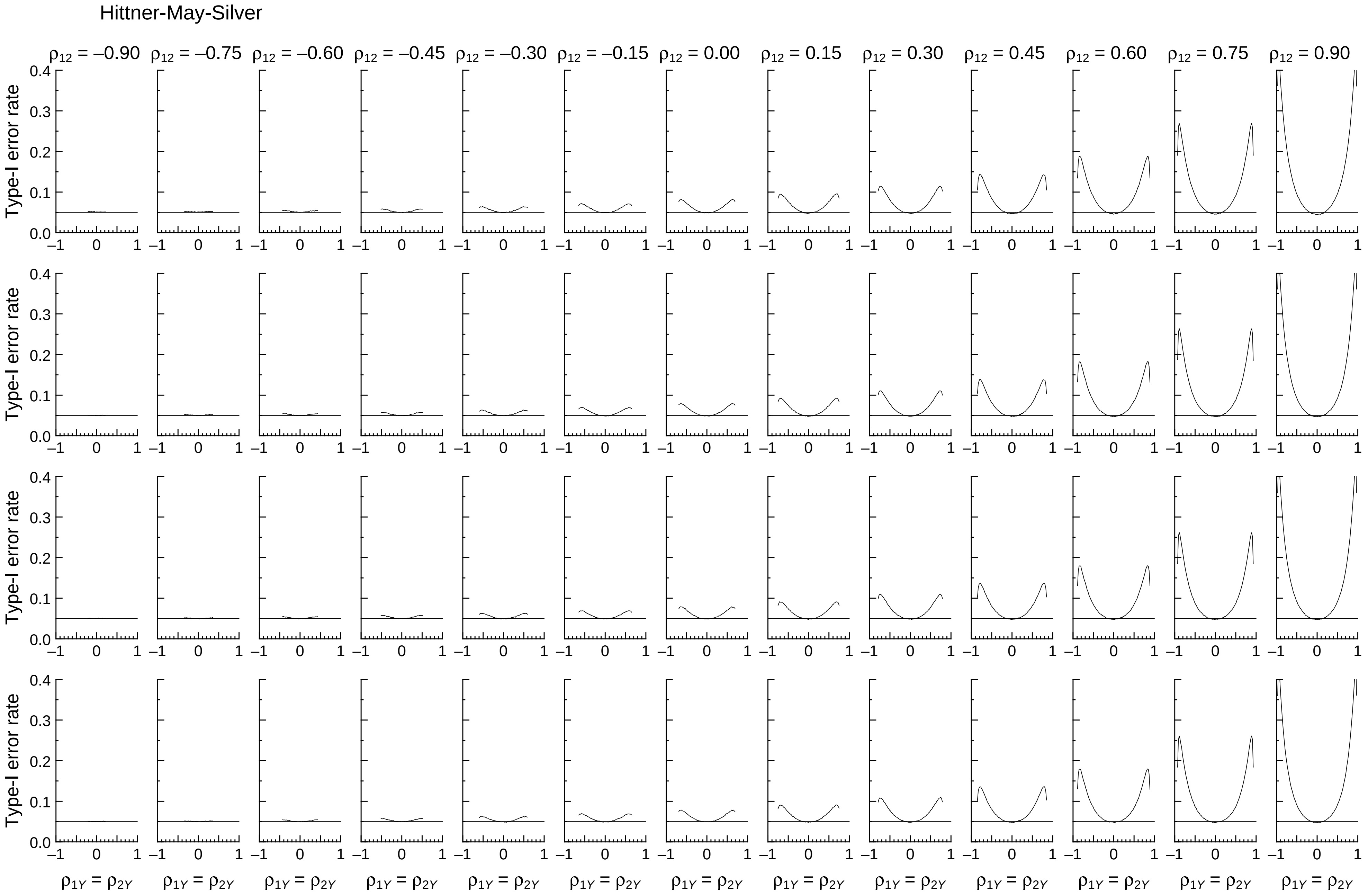

Section C: Type-I error rates of each test with uniform data (sample size top to bottom: 20, 50, 100, 200)

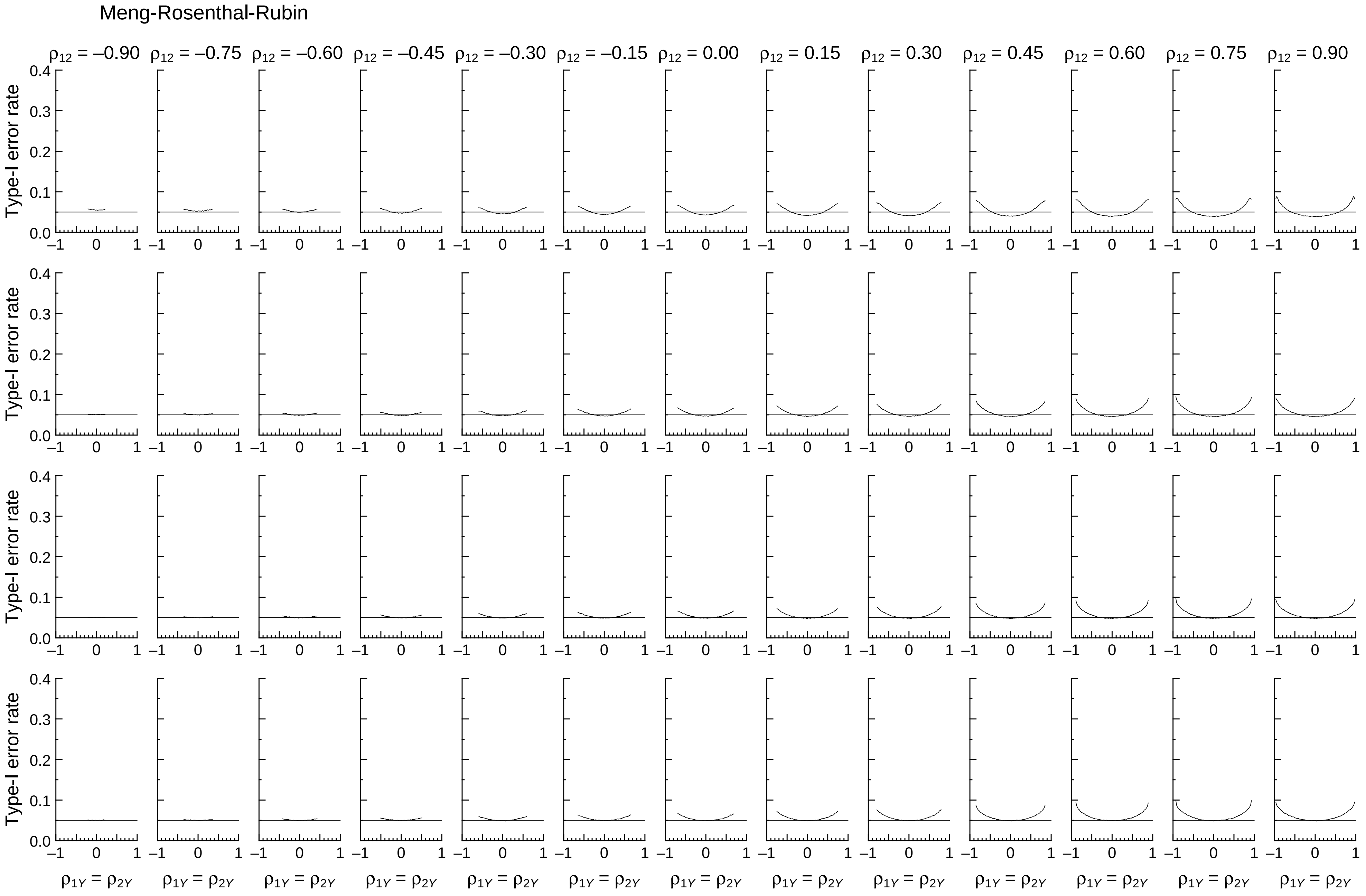

Section C: Type-I error rates of each test with uniform data (sample size top to bottom: 20, 50, 100, 200)

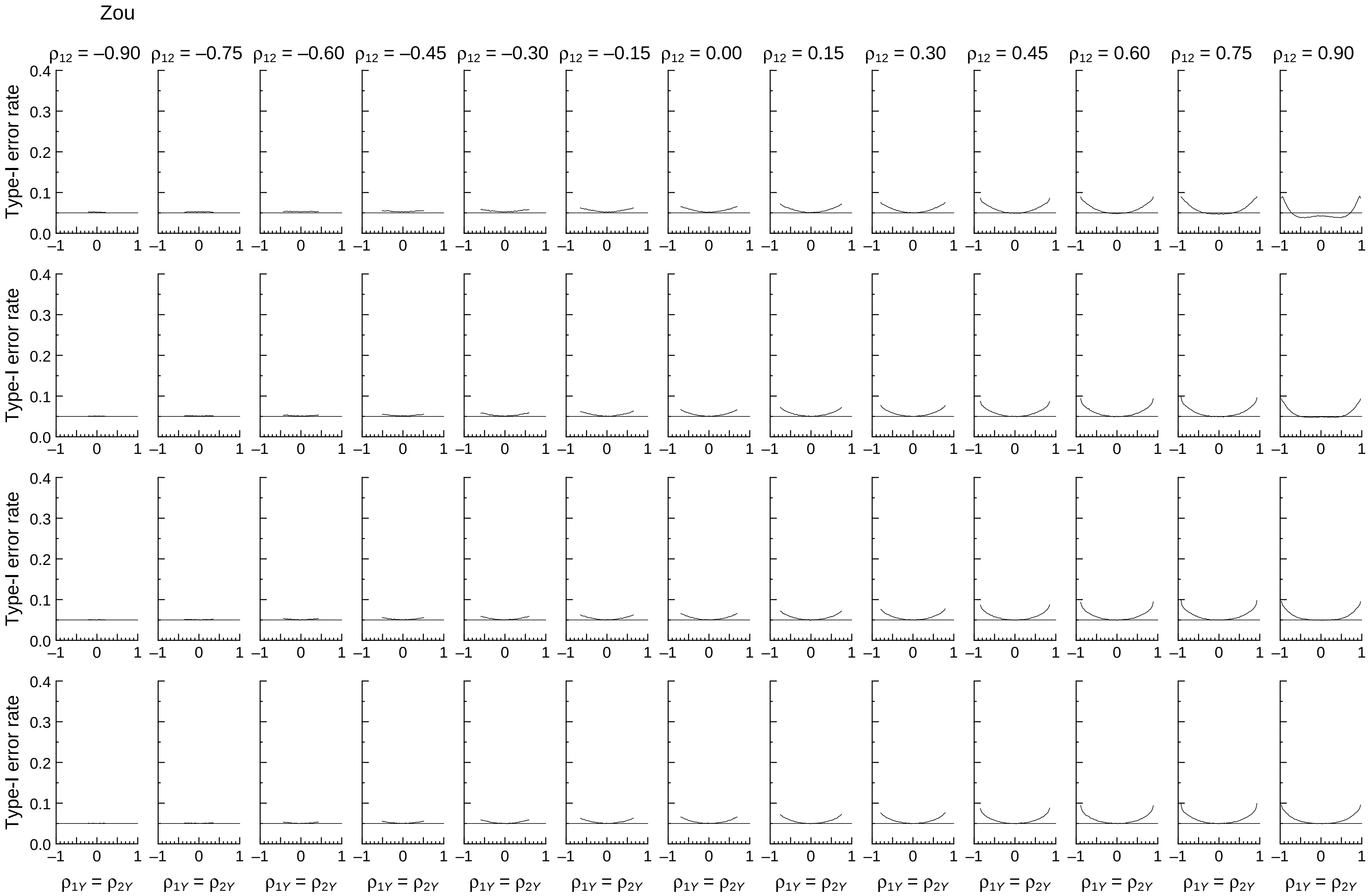

Section D: Power of each test with uniform data (sample size top to bottom: 20, 50, 100, 200)

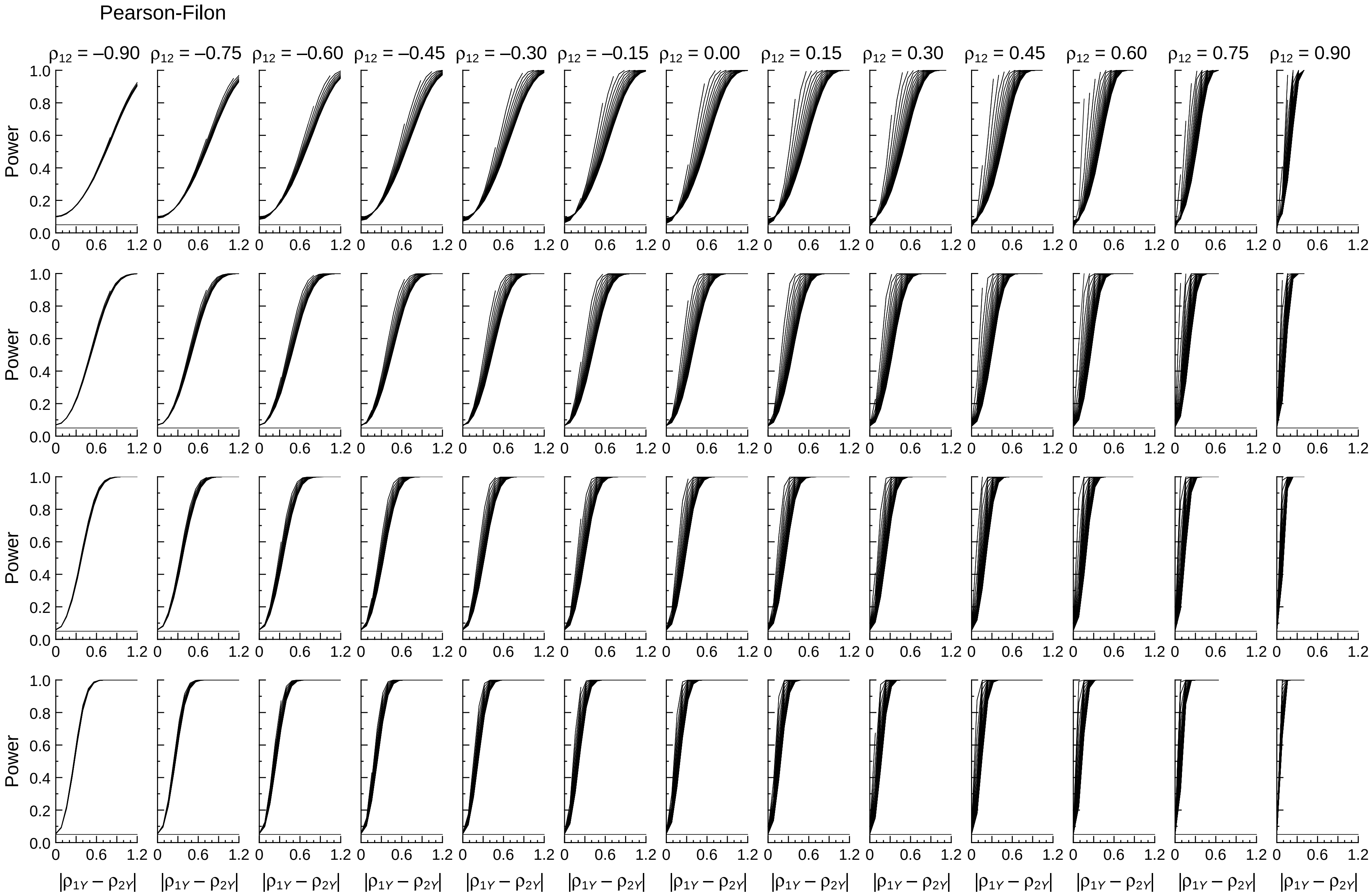

Section D: Power of each test with uniform data (sample size top to bottom: 20, 50, 100, 200)

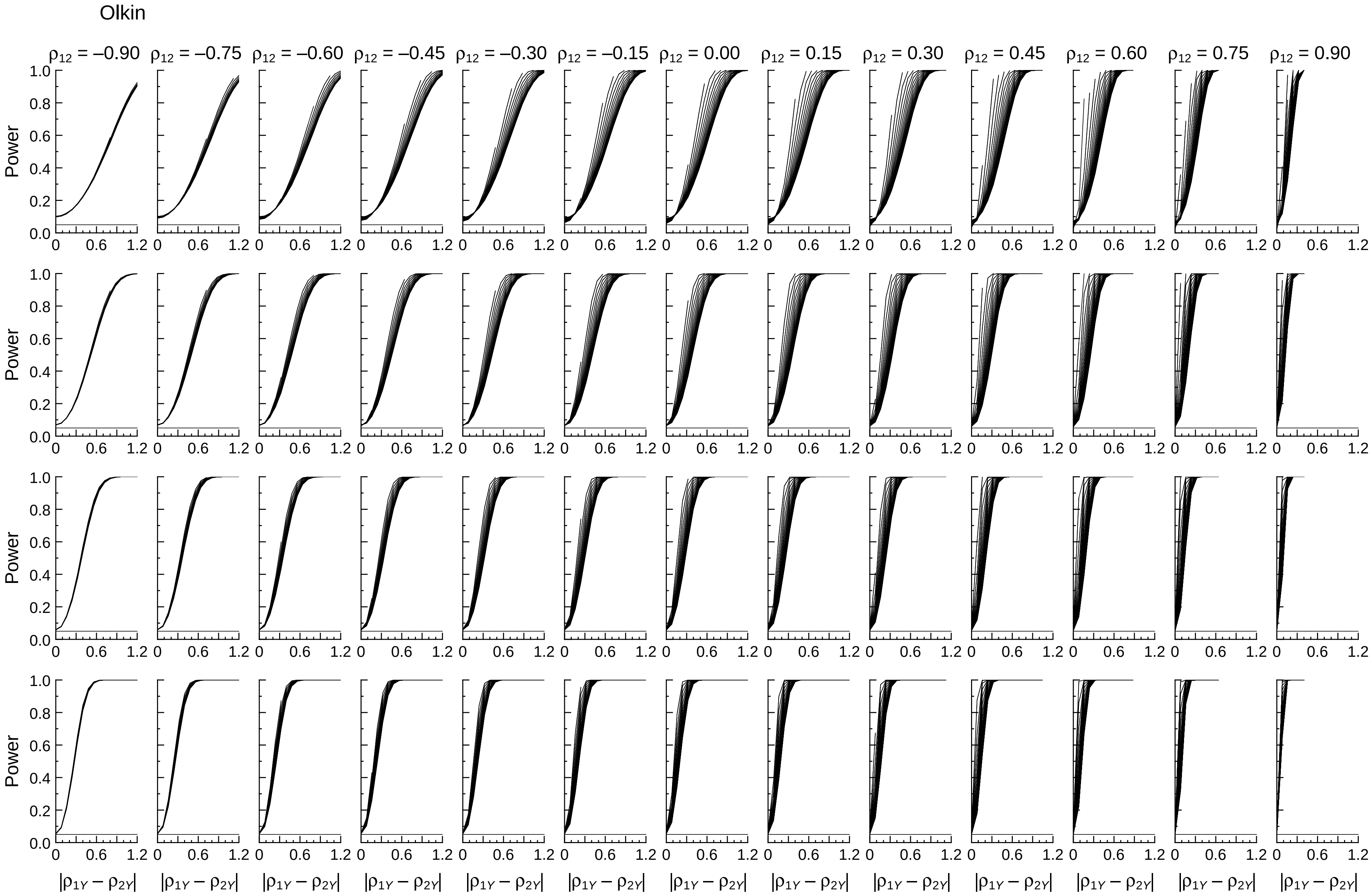

Section D: Power of each test with uniform data (sample size top to bottom: 20, 50, 100, 200)

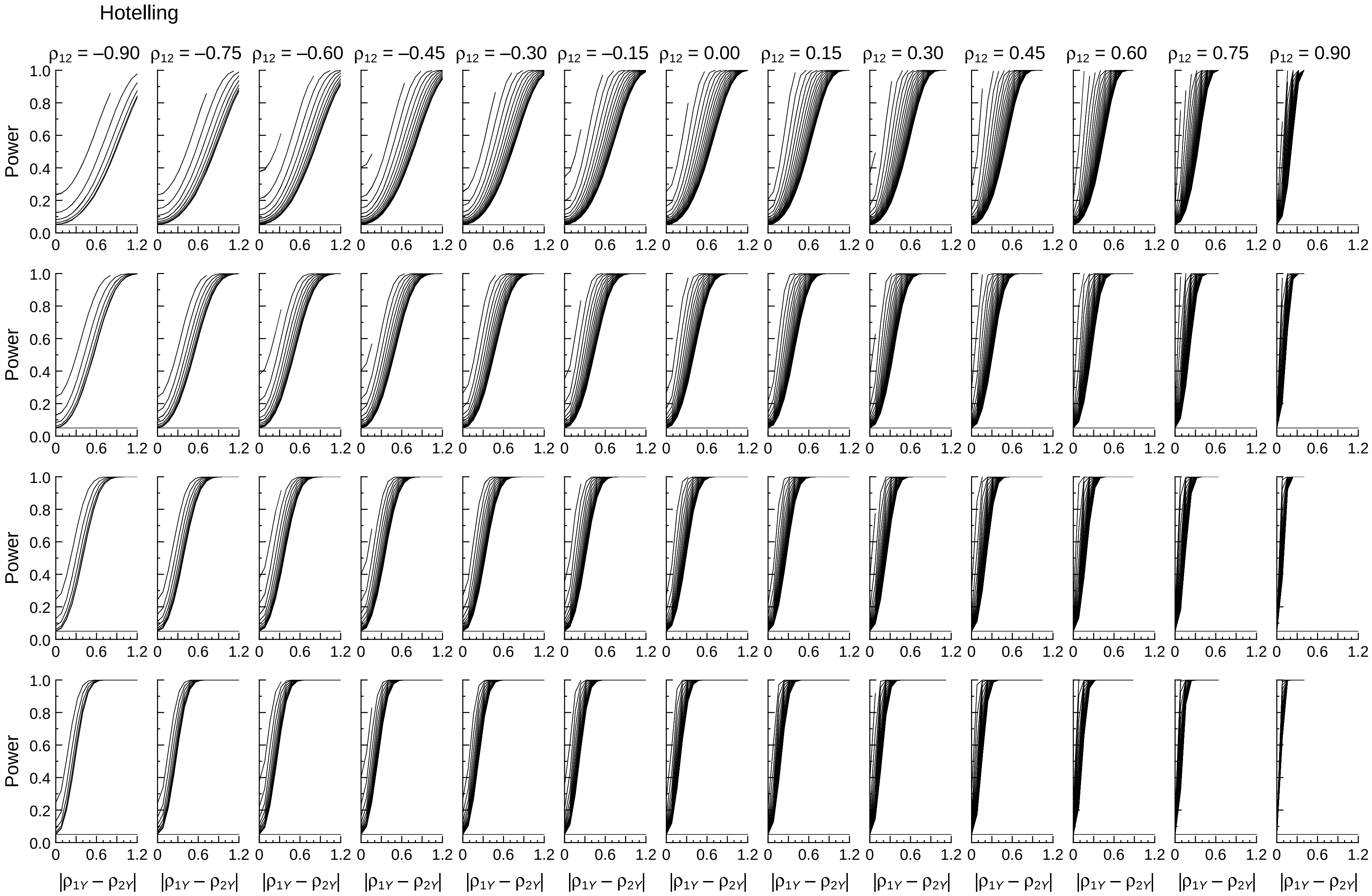

Section D: Power of each test with uniform data (sample size top to bottom: 20, 50, 100, 200)

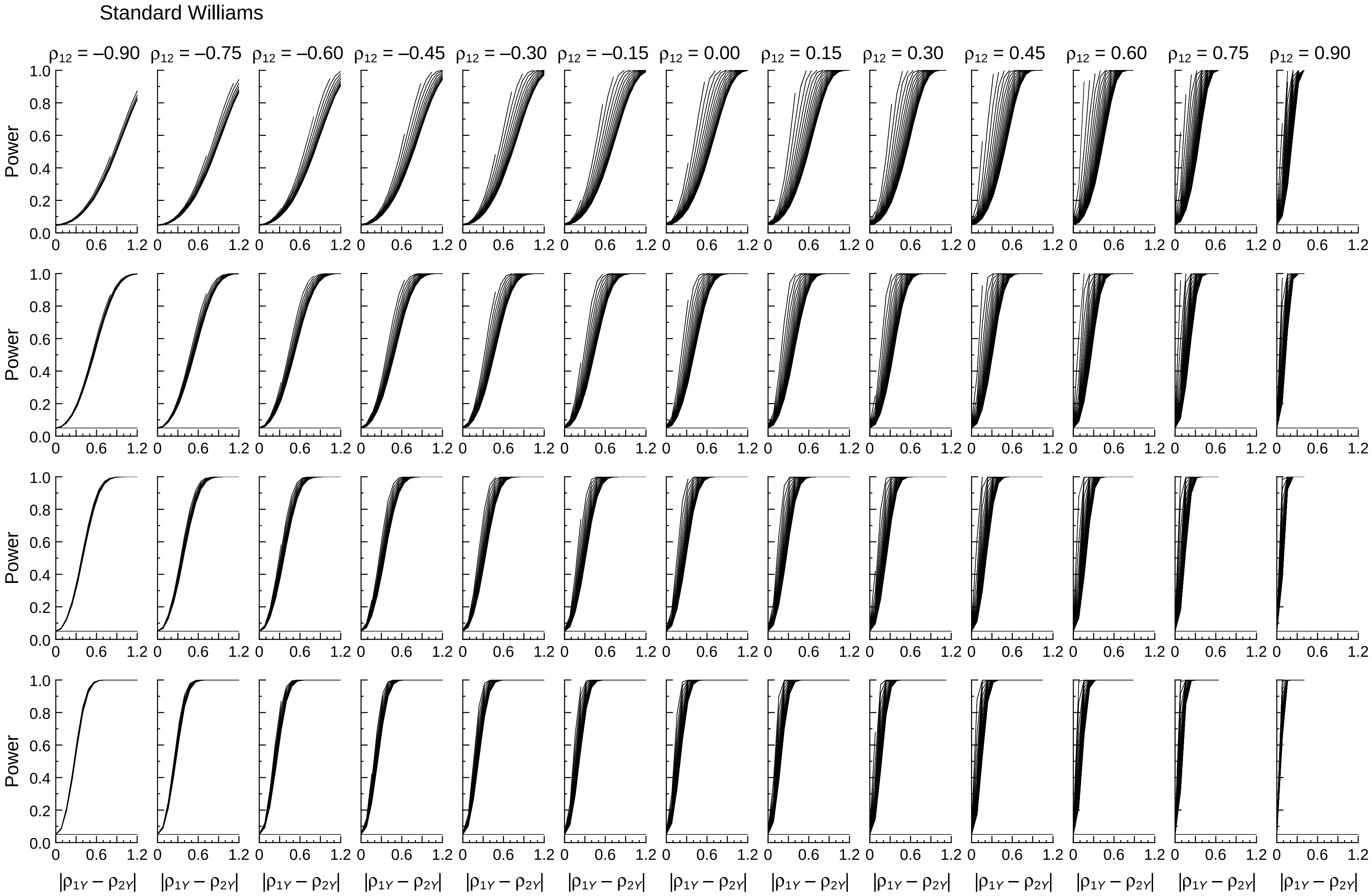

Section D: Power of each test with uniform data (sample size top to bottom: 20, 50, 100, 200)

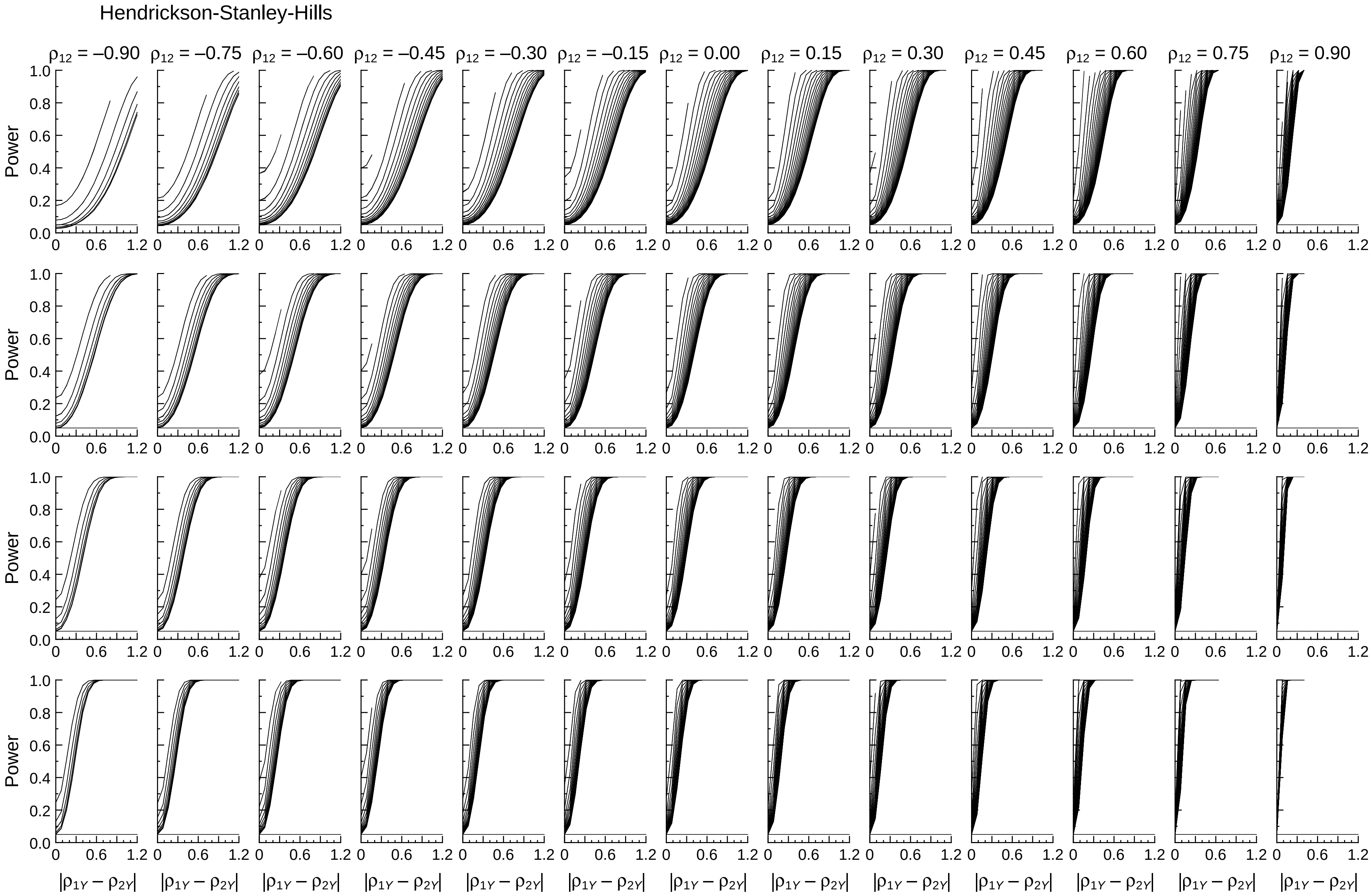

Section D: Power of each test with uniform data (sample size top to bottom: 20, 50, 100, 200)

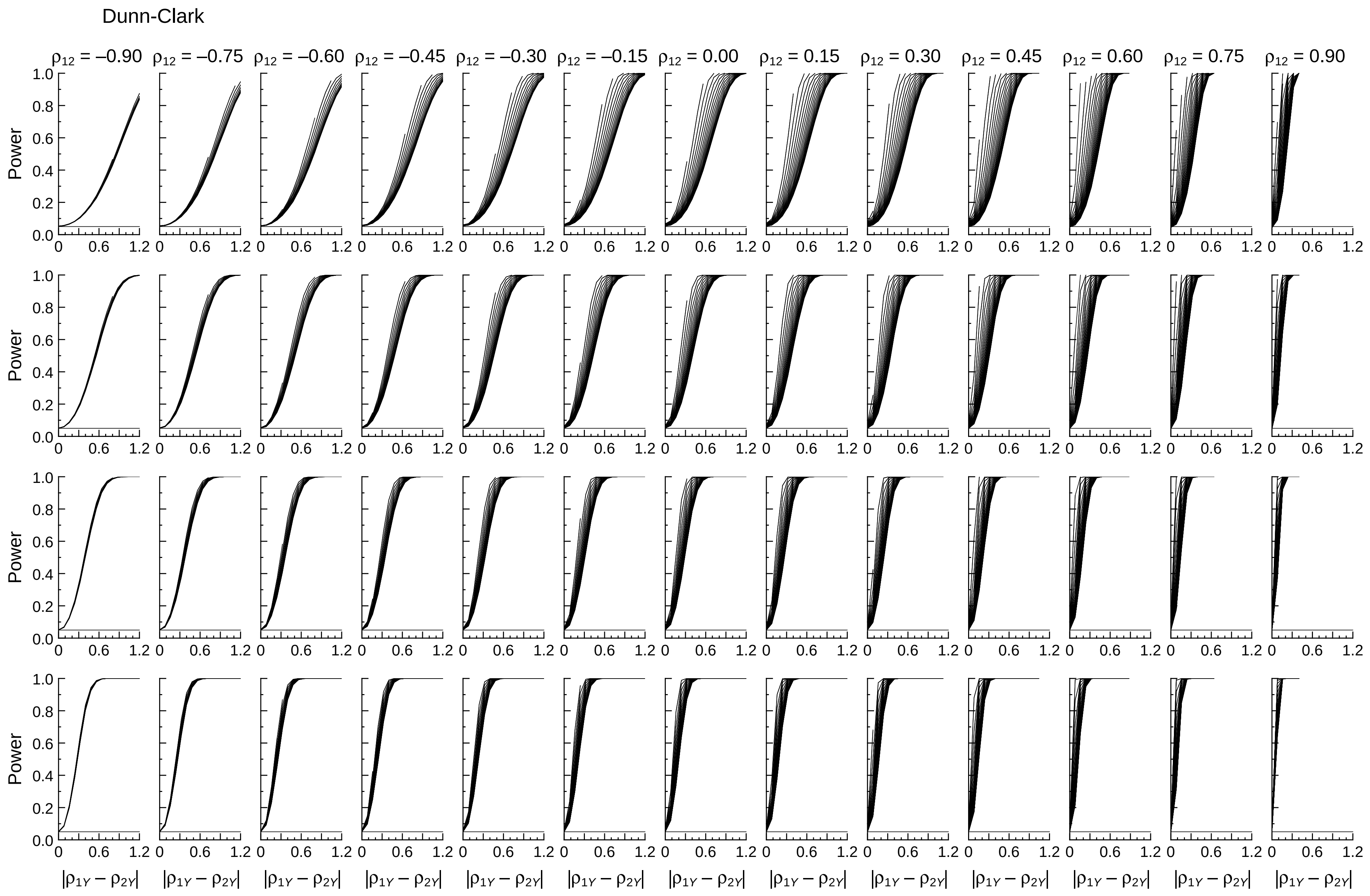

Section D: Power of each test with uniform data (sample size top to bottom: 20, 50, 100, 200)

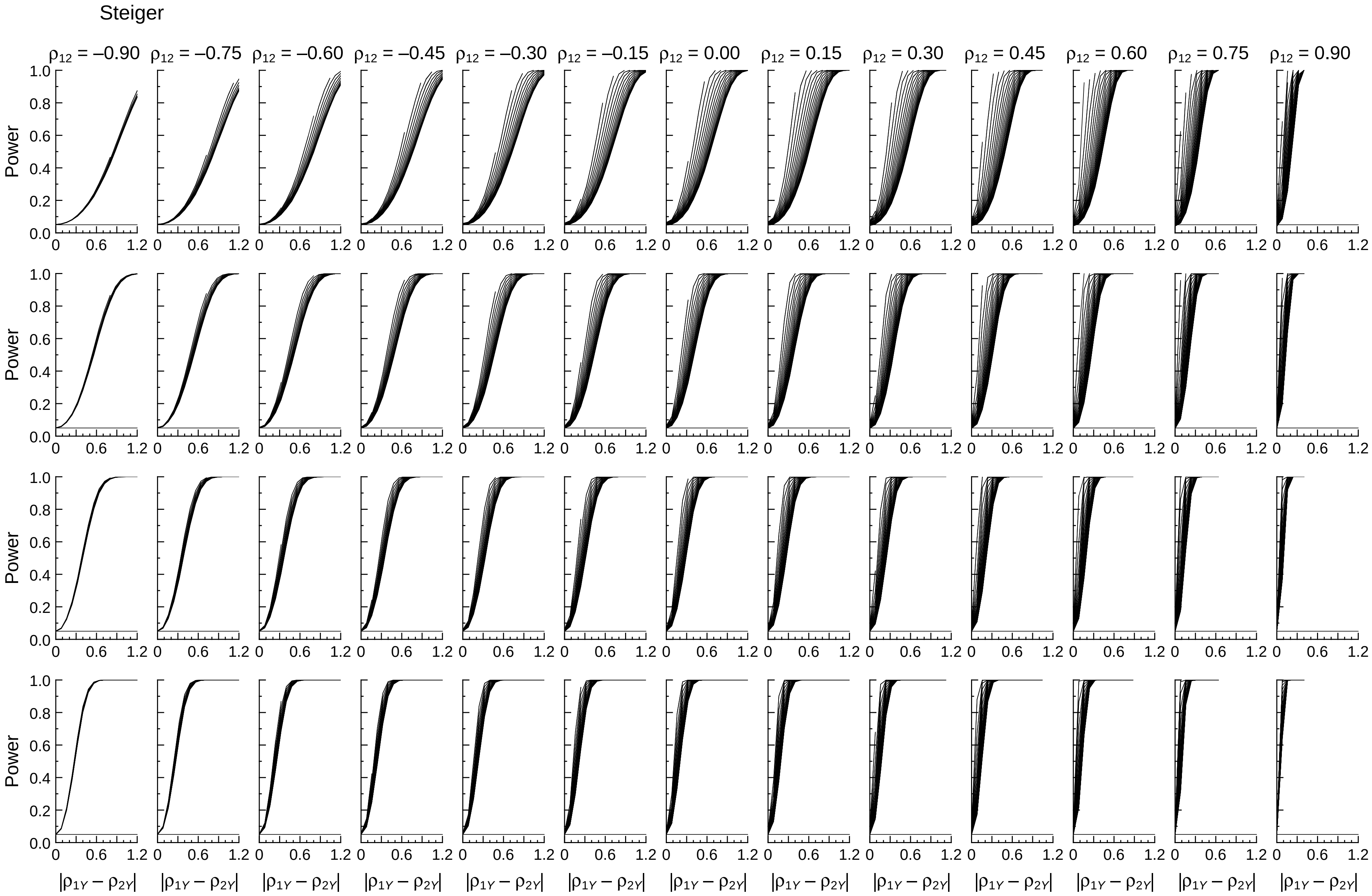

Section D: Power of each test with uniform data (sample size top to bottom: 20, 50, 100, 200)

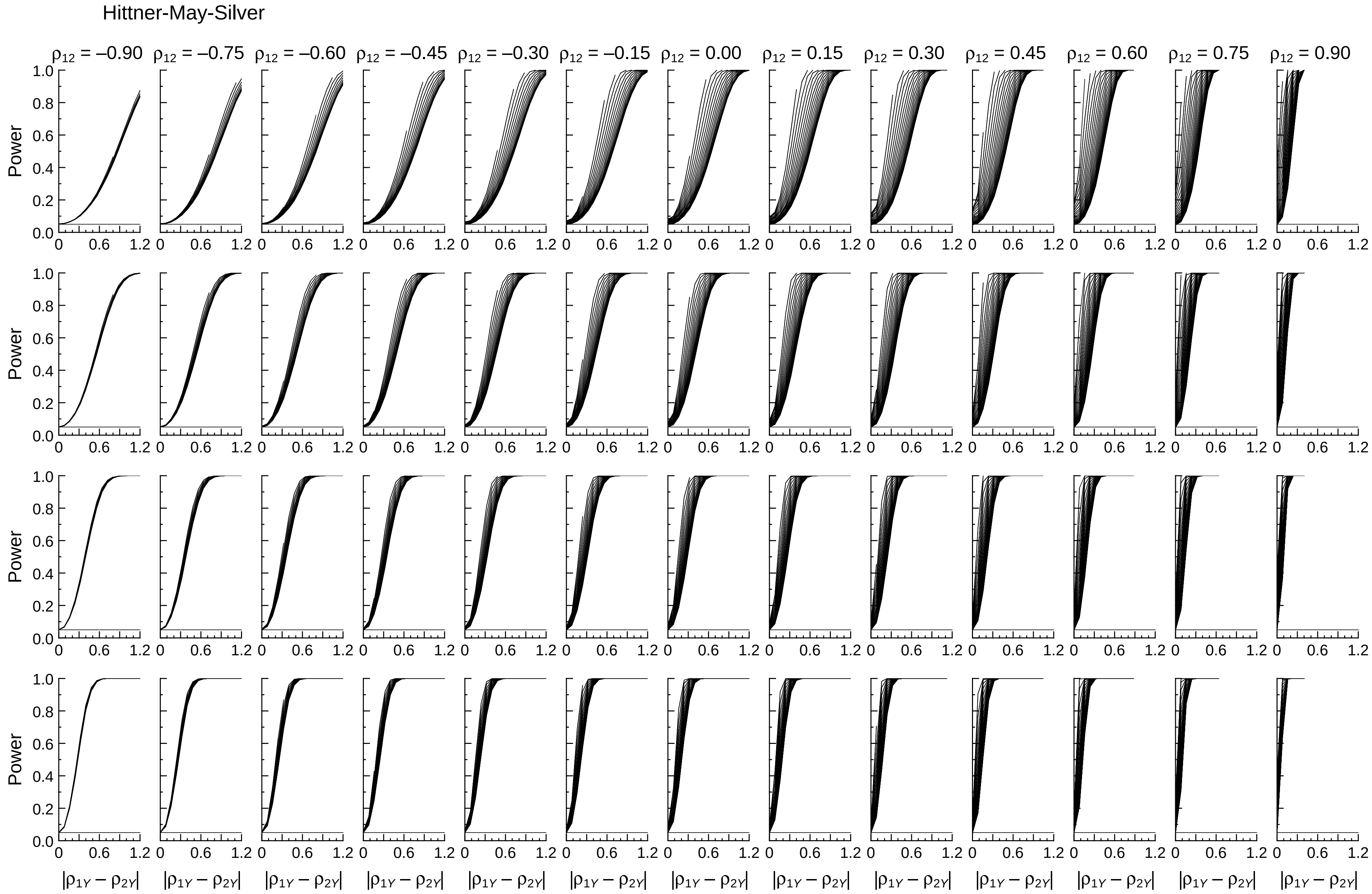

Section D: Power of each test with uniform data (sample size top to bottom: 20, 50, 100, 200)

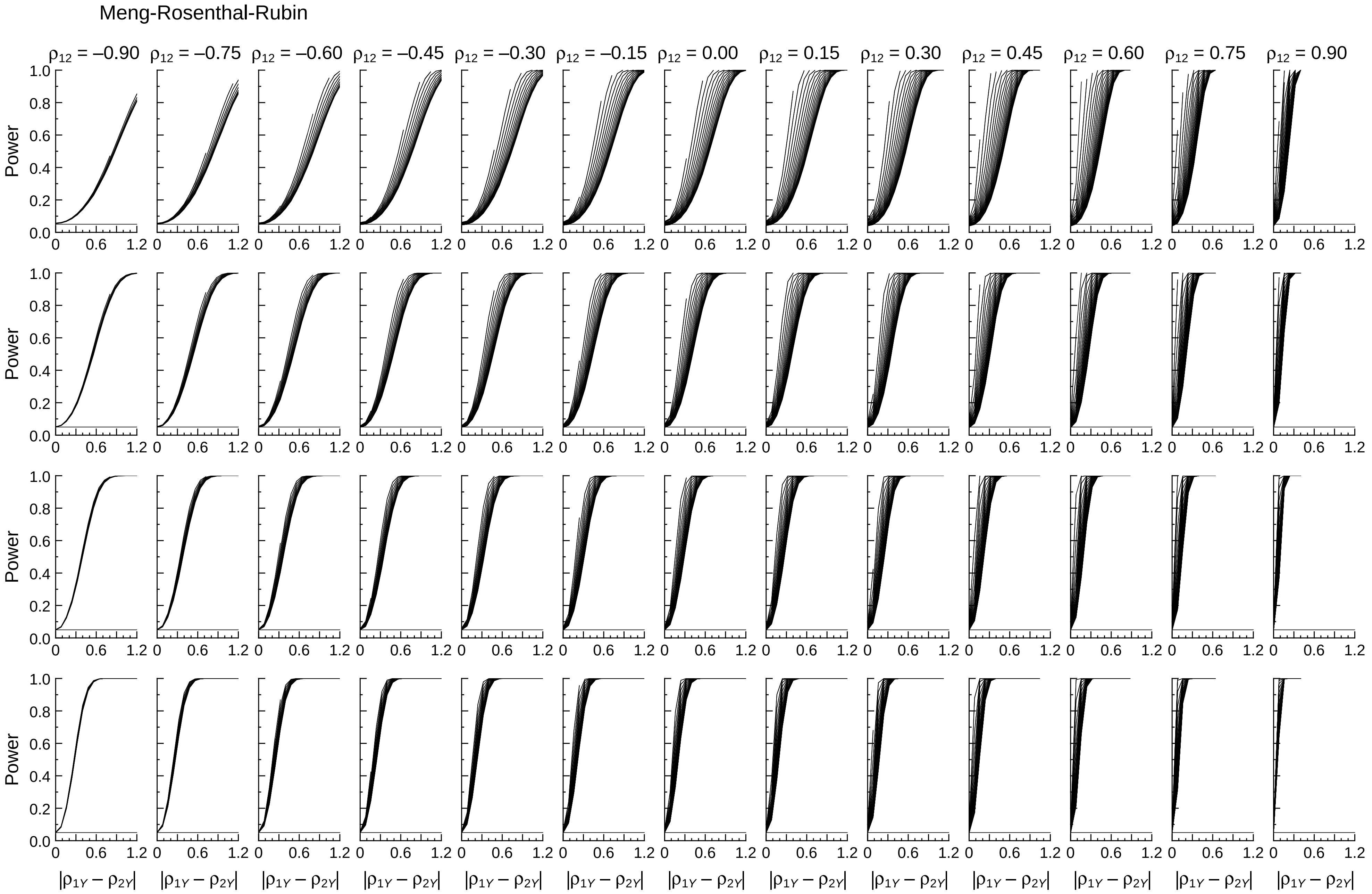

Section D: Power of each test with uniform data (sample size top to bottom: 20, 50, 100, 200)

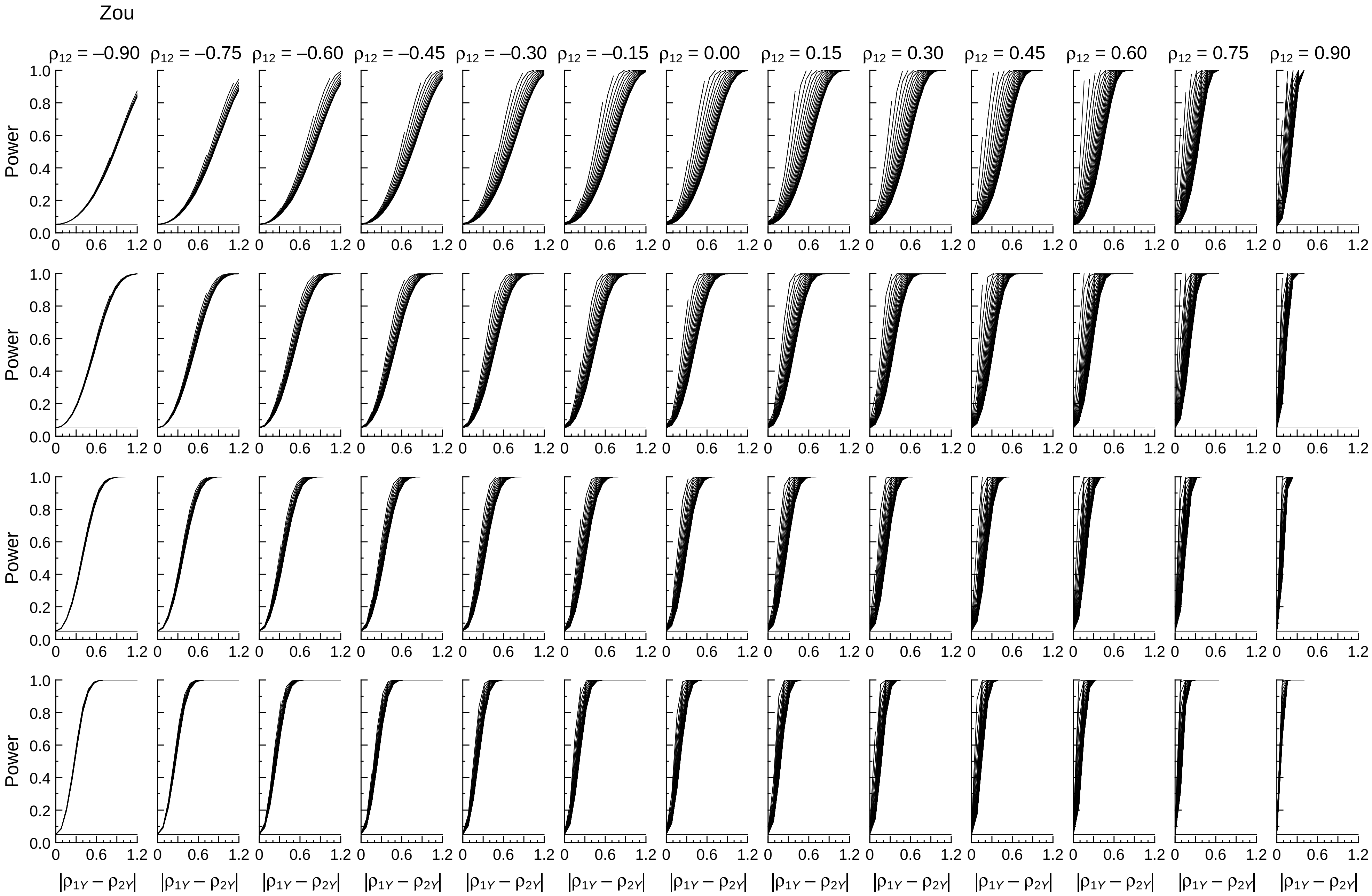

Section E: Type-I error rates of each test with Beta(2, 5) data (sample size top to bottom: 20, 50, 100, 200)

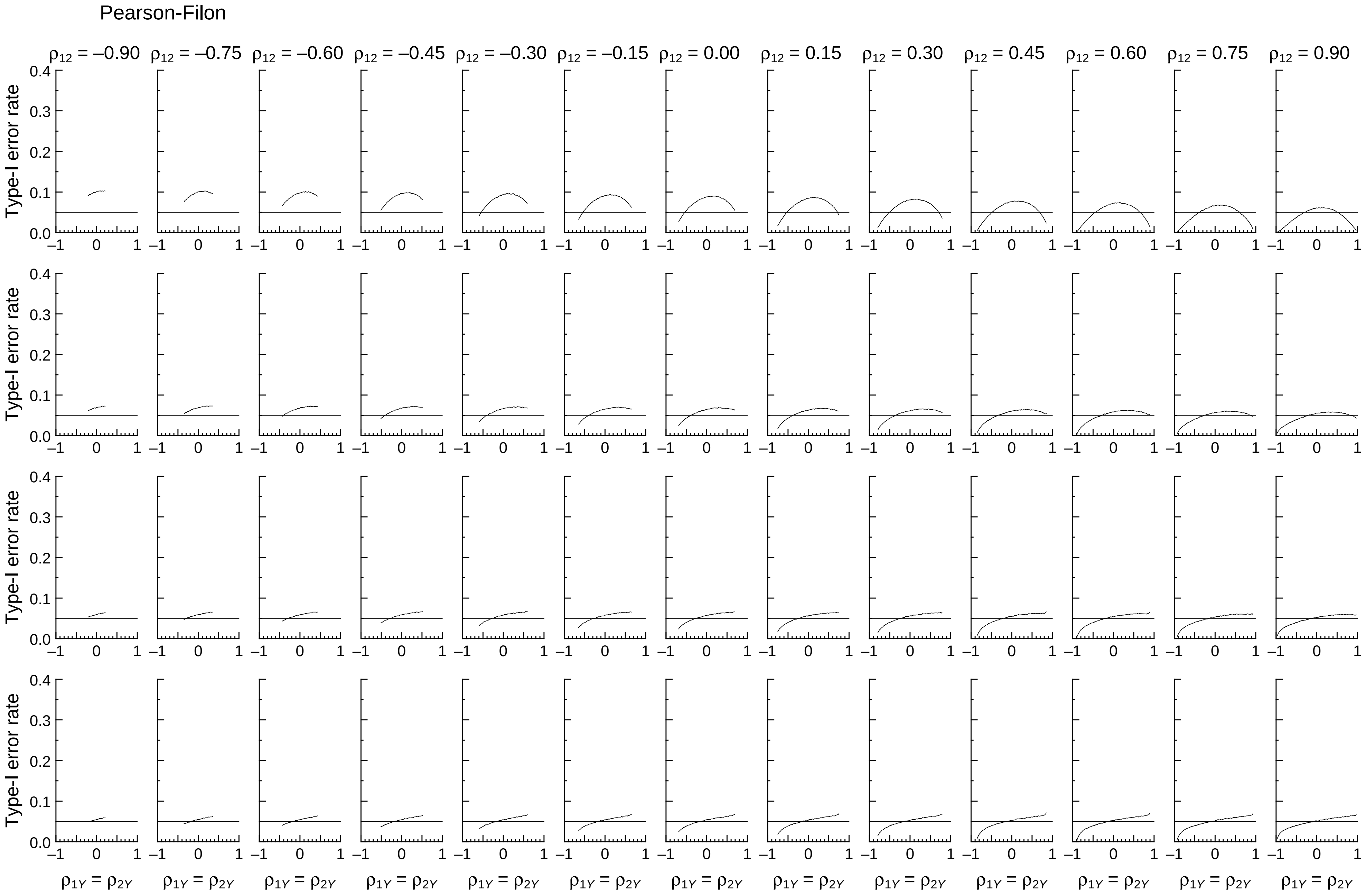

Section E: Type-I error rates of each test with Beta(2, 5) data (sample size top to bottom: 20, 50, 100, 200)

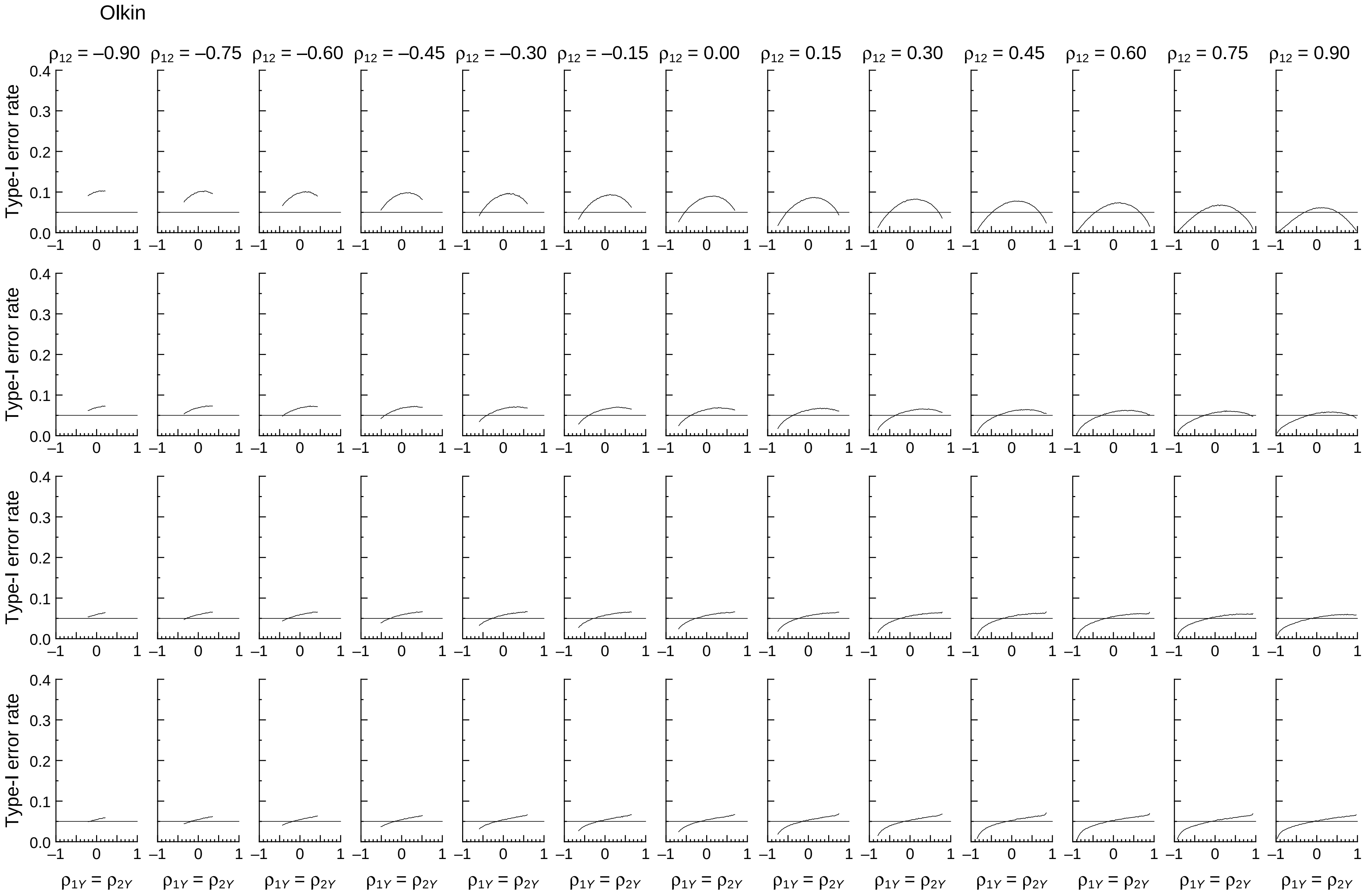

Section E: Type-I error rates of each test with Beta(2, 5) data (sample size top to bottom: 20, 50, 100, 200)

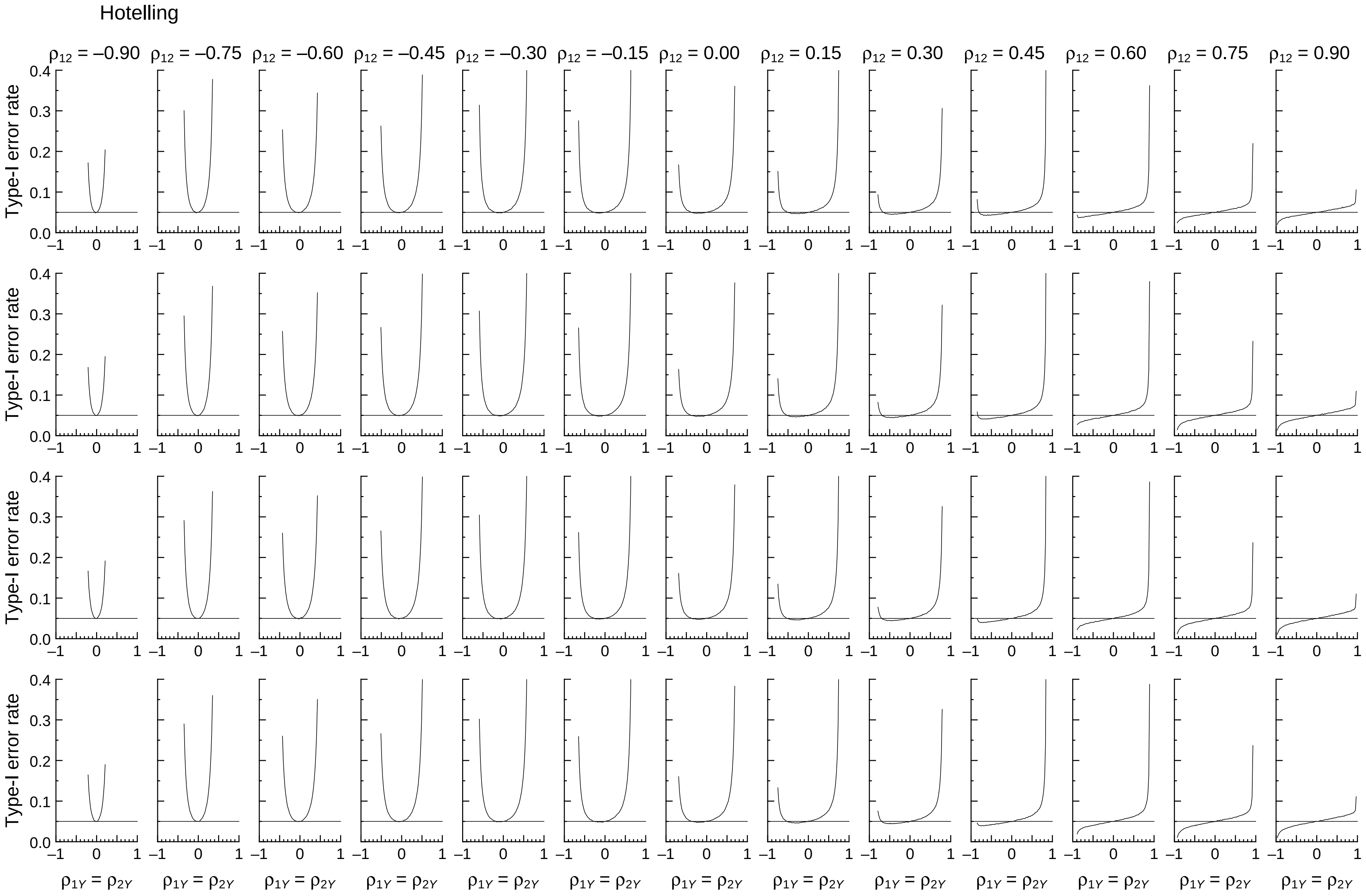

Section E: Type-I error rates of each test with Beta(2, 5) data (sample size top to bottom: 20, 50, 100, 200)

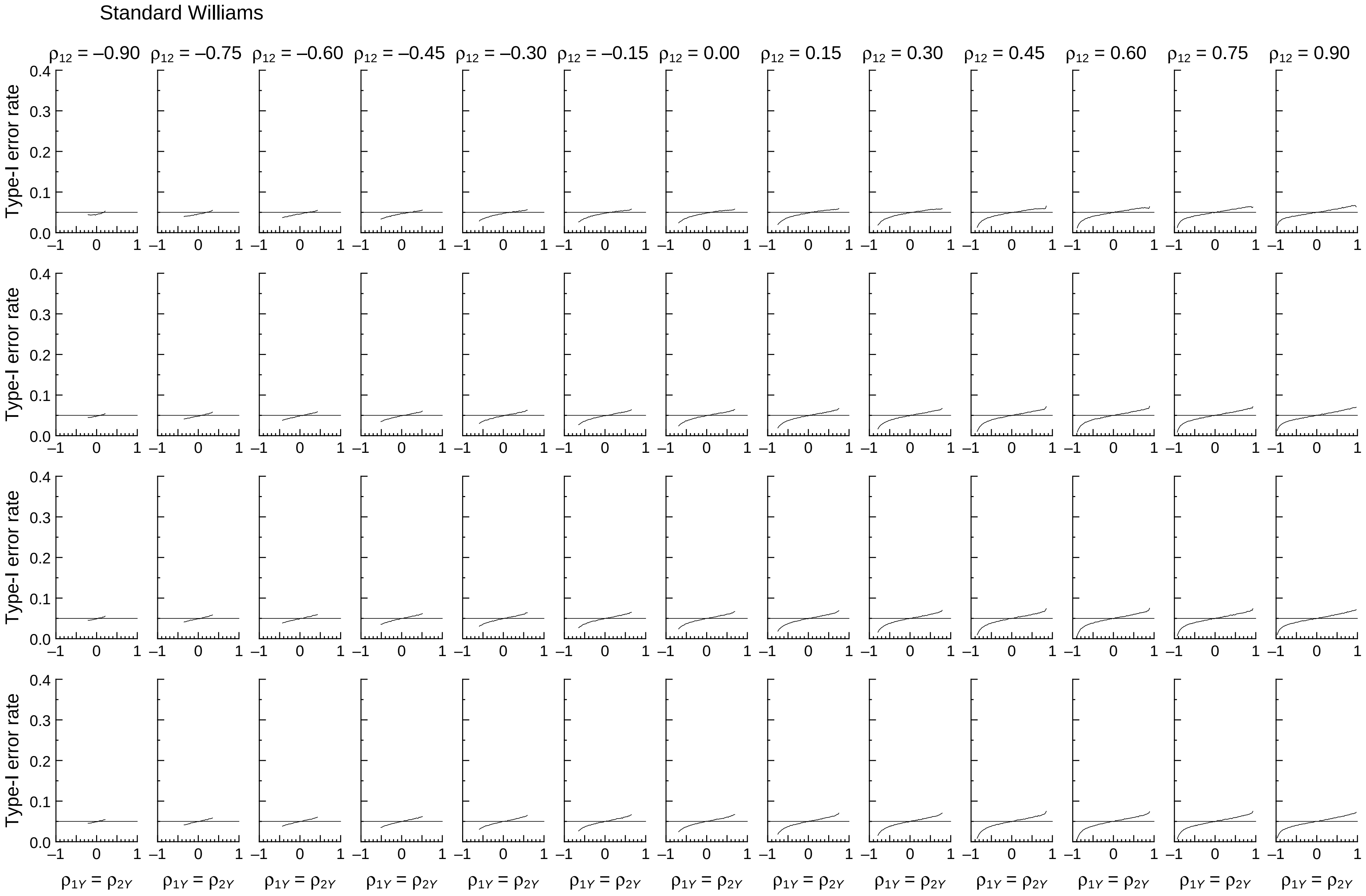

Section E: Type-I error rates of each test with Beta(2, 5) data (sample size top to bottom: 20, 50, 100, 200)

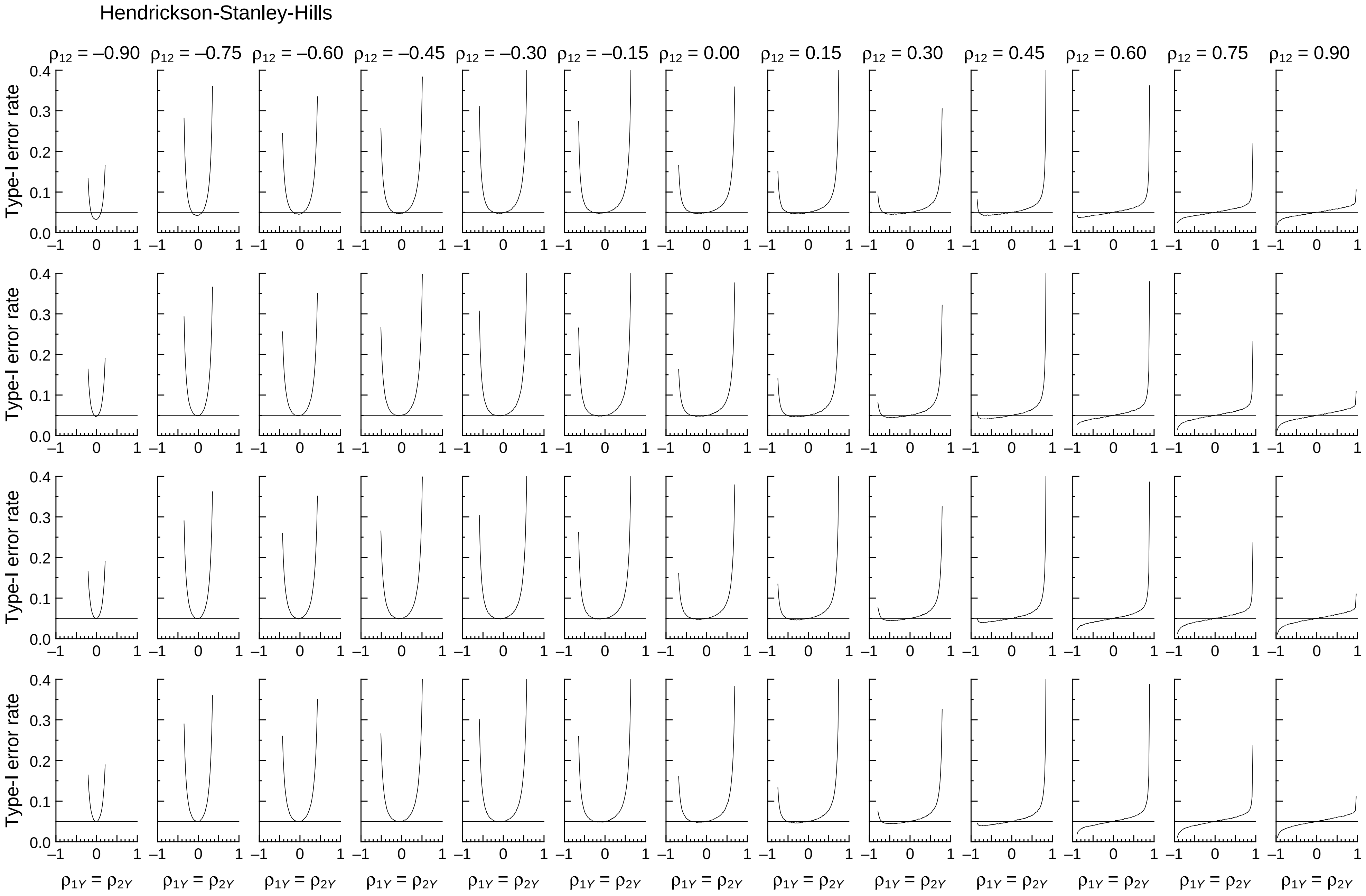

Section E: Type-I error rates of each test with Beta(2, 5) data (sample size top to bottom: 20, 50, 100, 200)

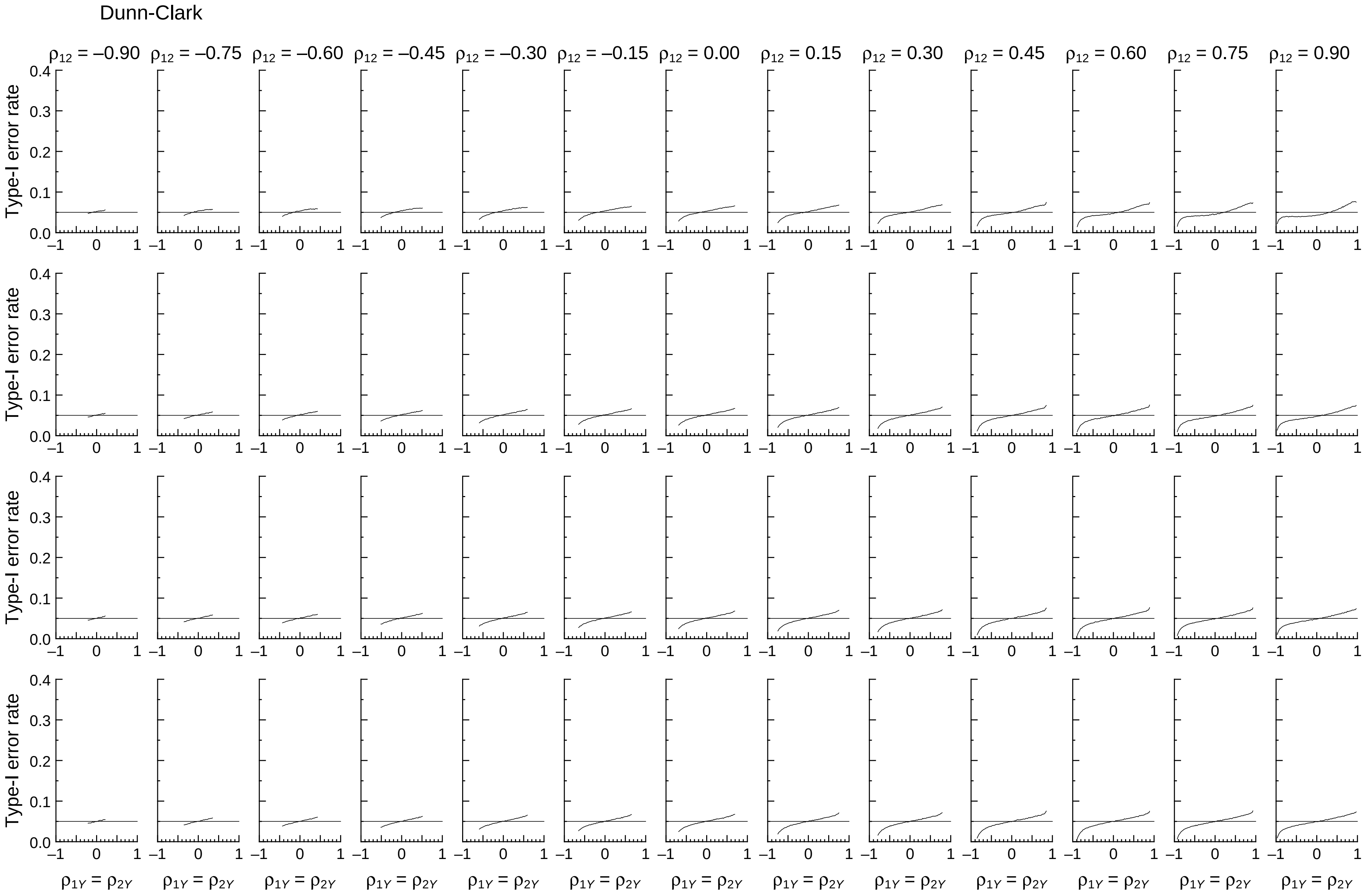

Section E: Type-I error rates of each test with Beta(2, 5) data (sample size top to bottom: 20, 50, 100, 200)

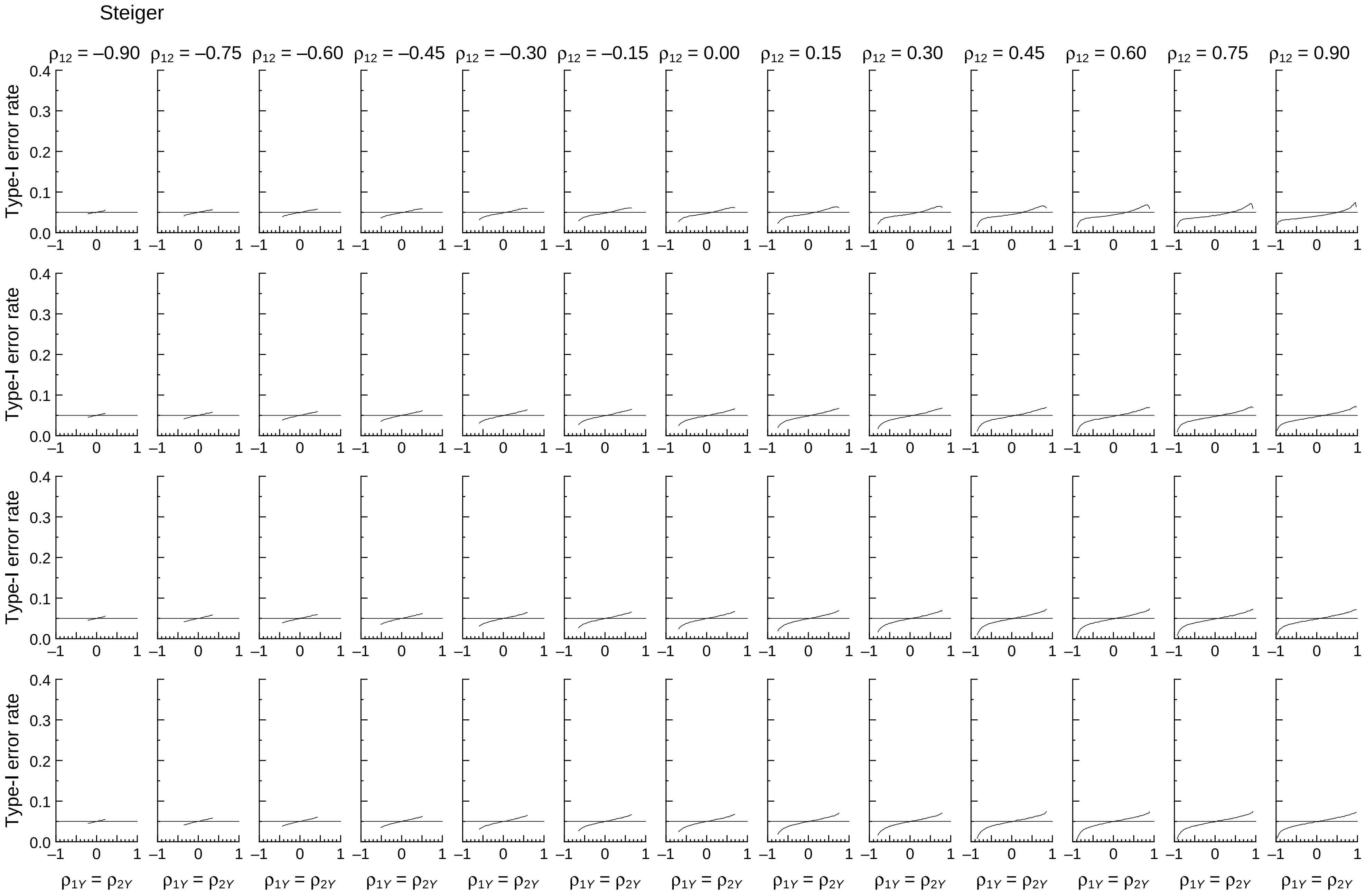

Section E: Type-I error rates of each test with Beta(2, 5) data (sample size top to bottom: 20, 50, 100, 200)

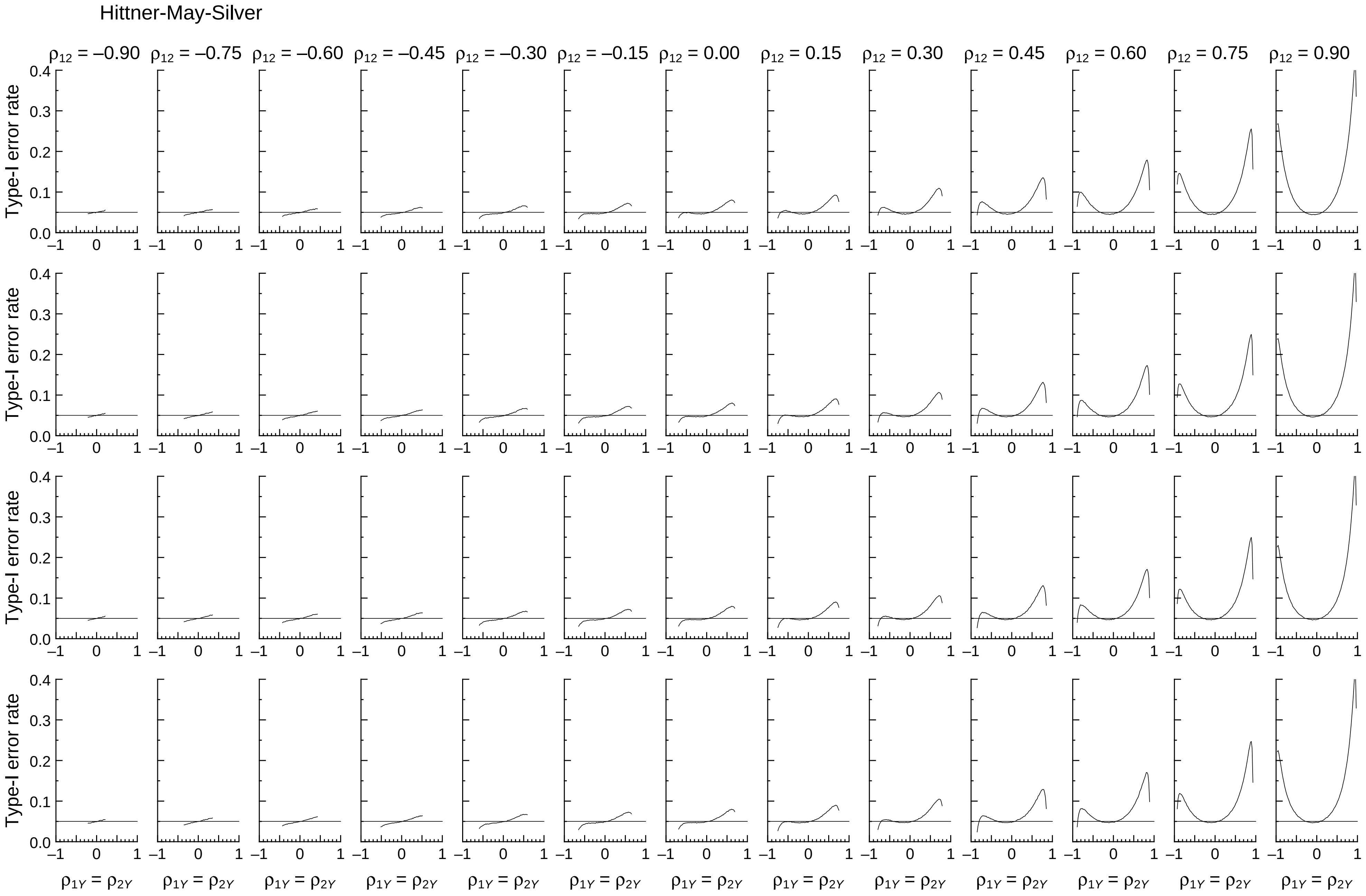

Section E: Type-I error rates of each test with Beta(2, 5) data (sample size top to bottom: 20, 50, 100, 200)

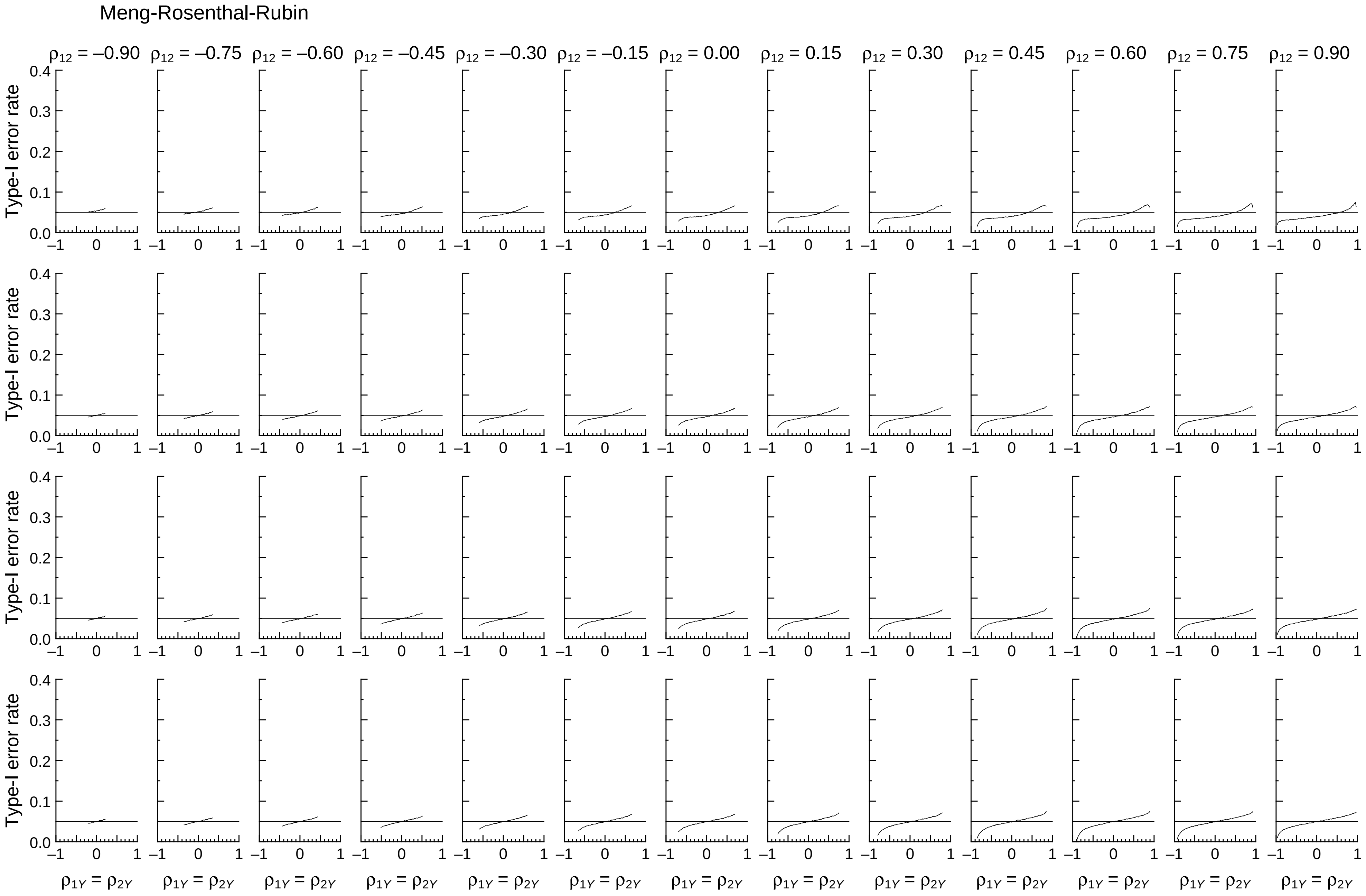

Section E: Type-I error rates of each test with Beta(2, 5) data (sample size top to bottom: 20, 50, 100, 200)

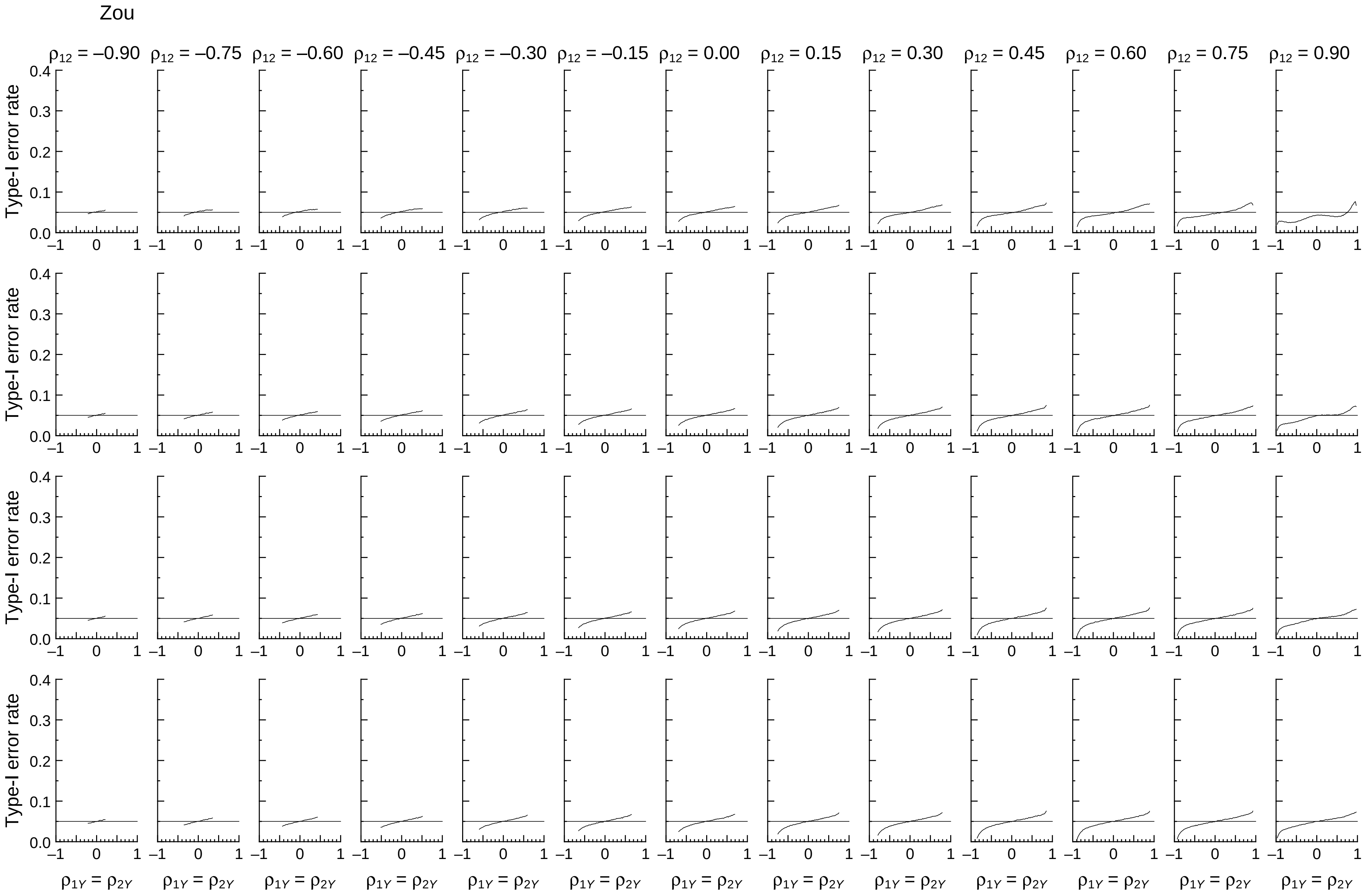

Section F: Power of each test with Beta(2, 5) data (sample size top to bottom: 20, 50, 100, 200)

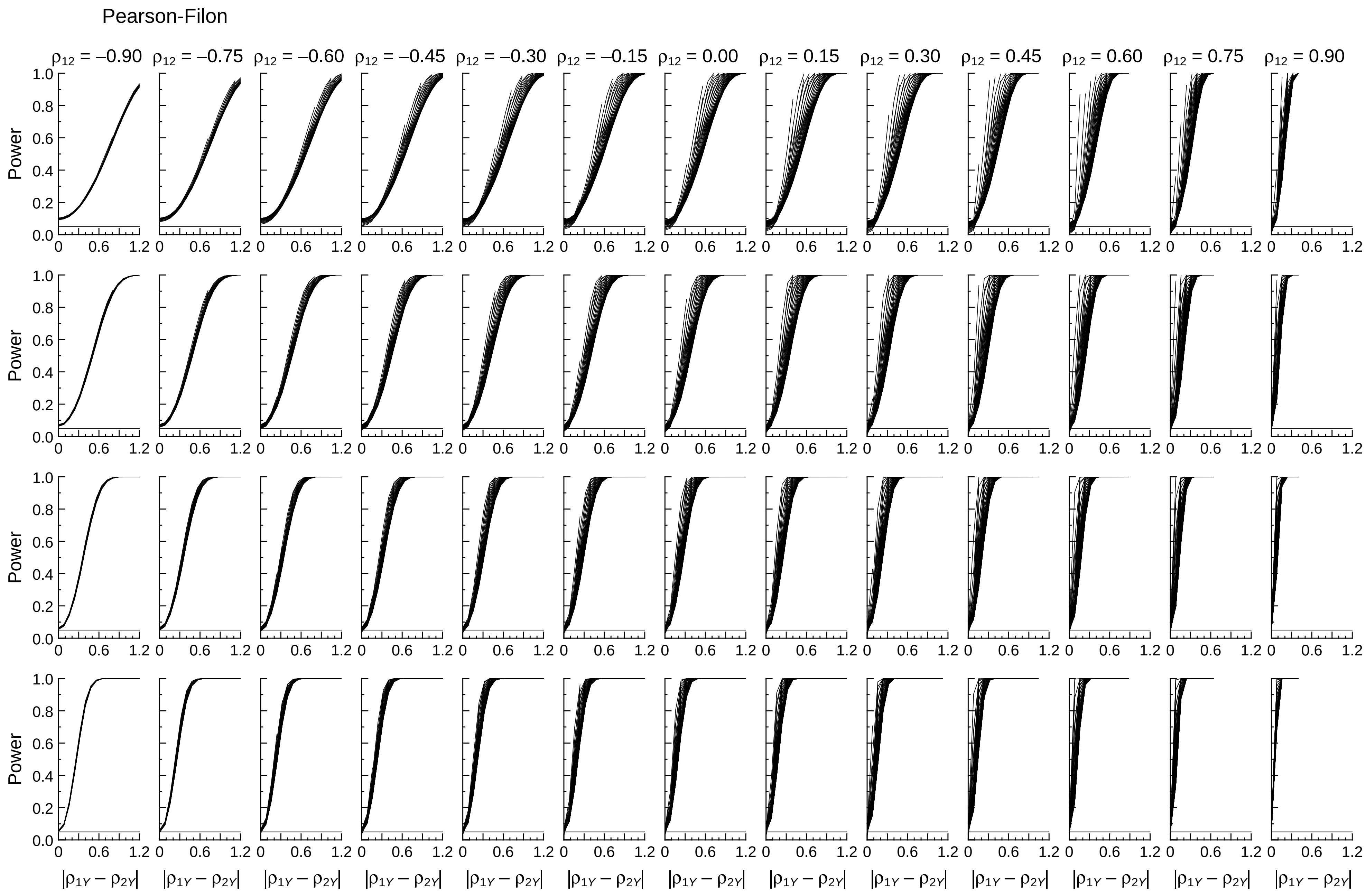

Section F: Power of each test with Beta(2, 5) data (sample size top to bottom: 20, 50, 100, 200)

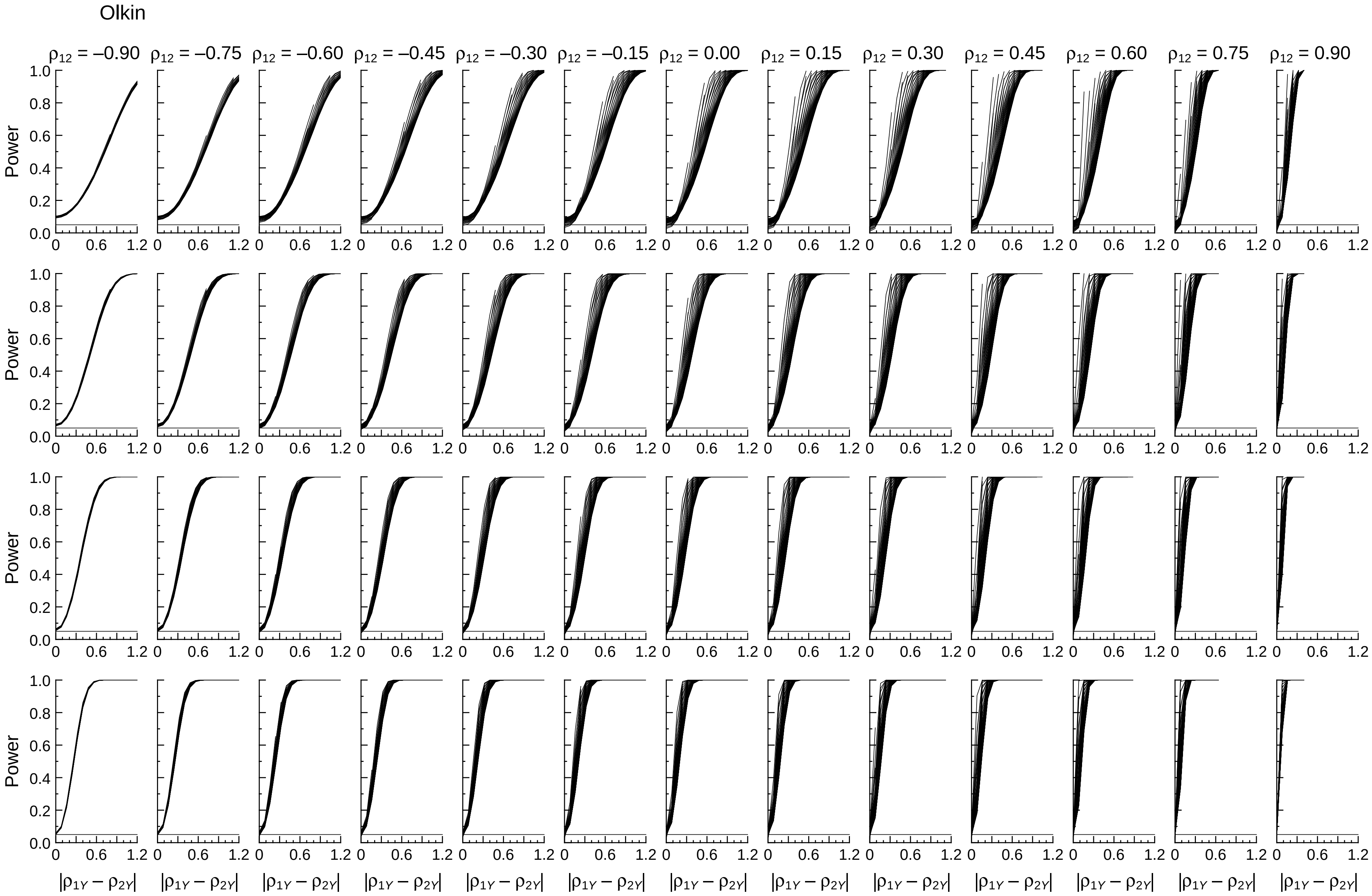

Section F: Power of each test with Beta(2, 5) data (sample size top to bottom: 20, 50, 100, 200)

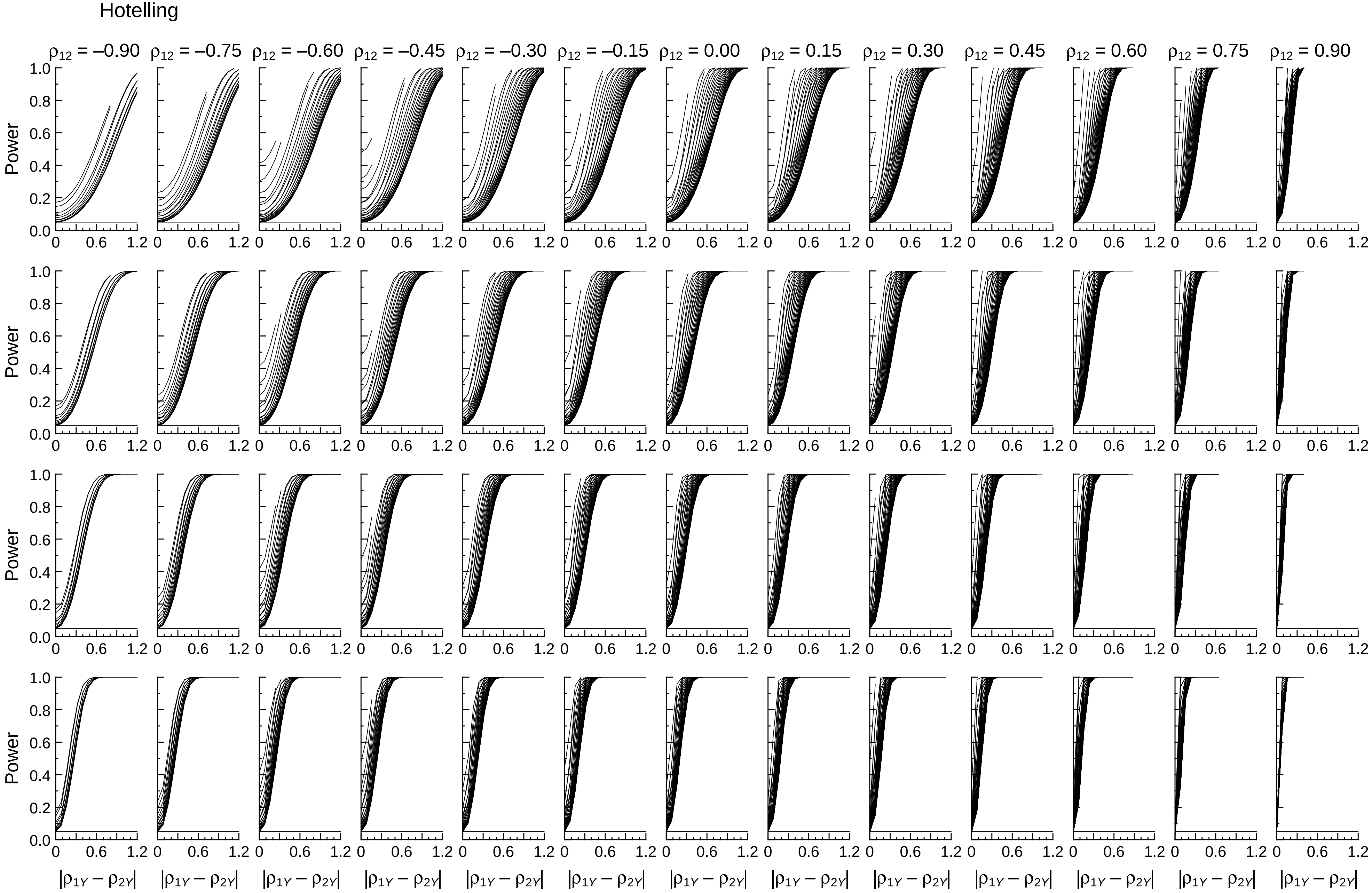

Section F: Power of each test with Beta(2, 5) data (sample size top to bottom: 20, 50, 100, 200)

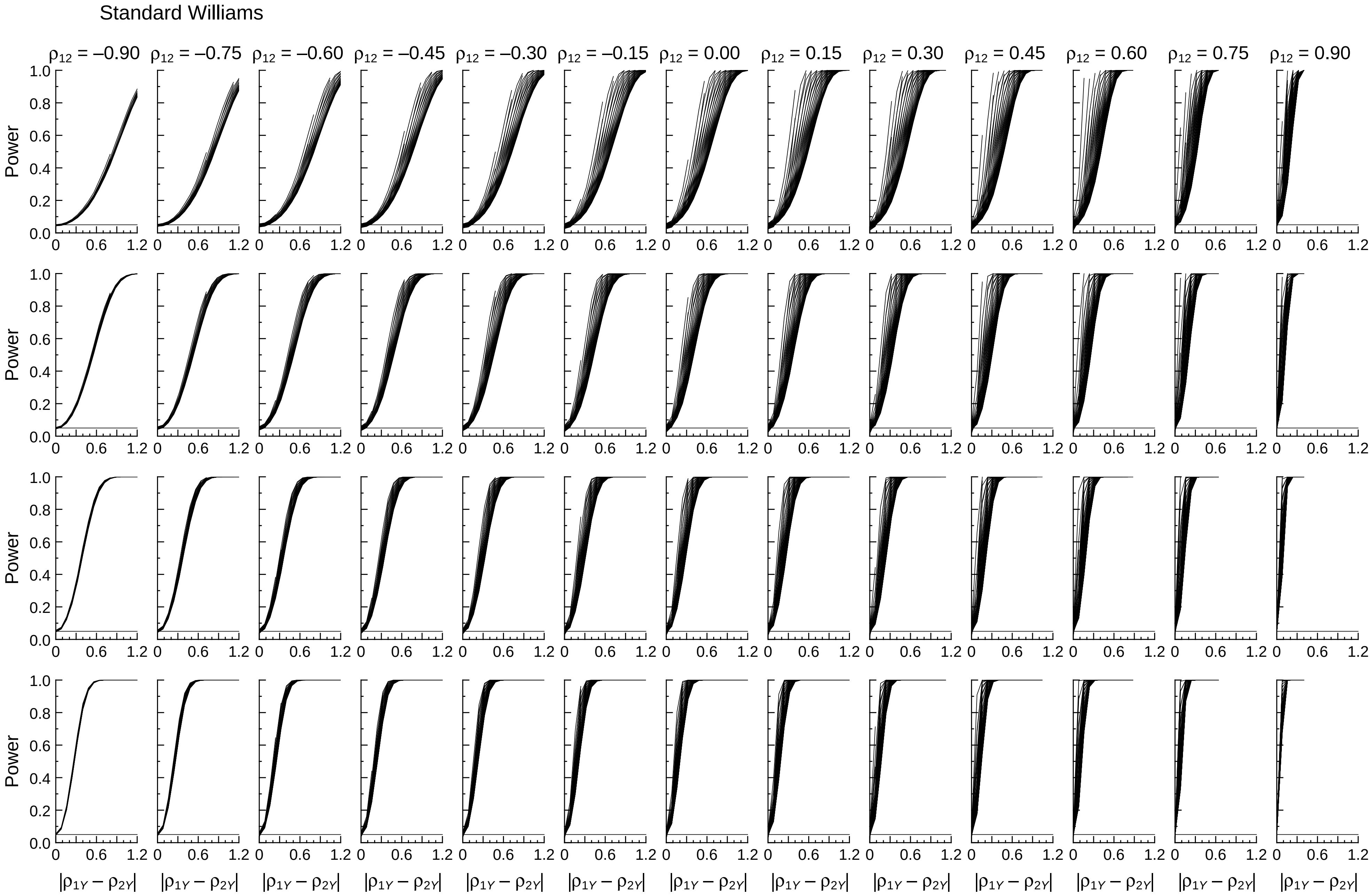

Section F: Power of each test with Beta(2, 5) data (sample size top to bottom: 20, 50, 100, 200)

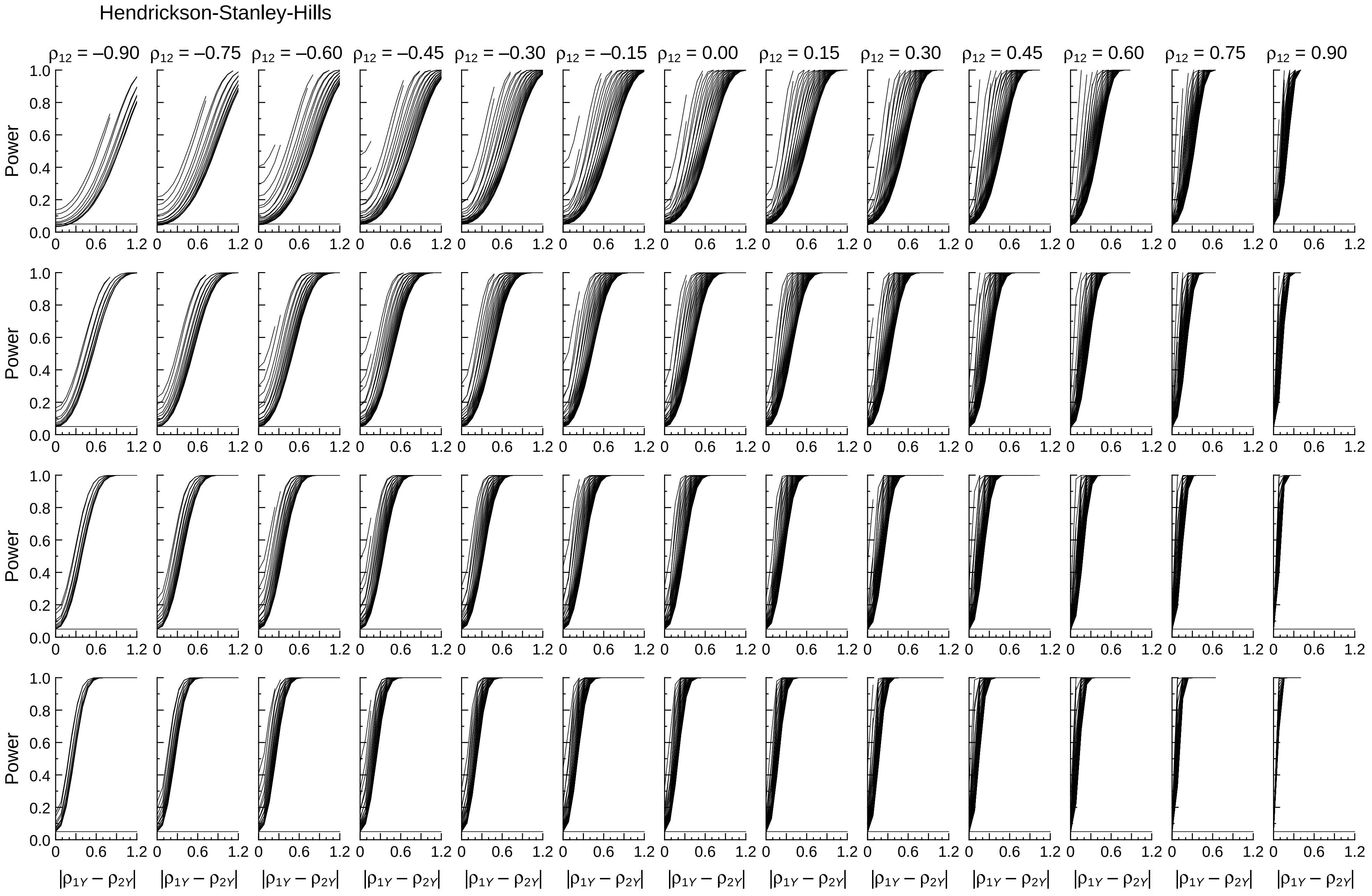

Section F: Power of each test with Beta(2, 5) data (sample size top to bottom: 20, 50, 100, 200)

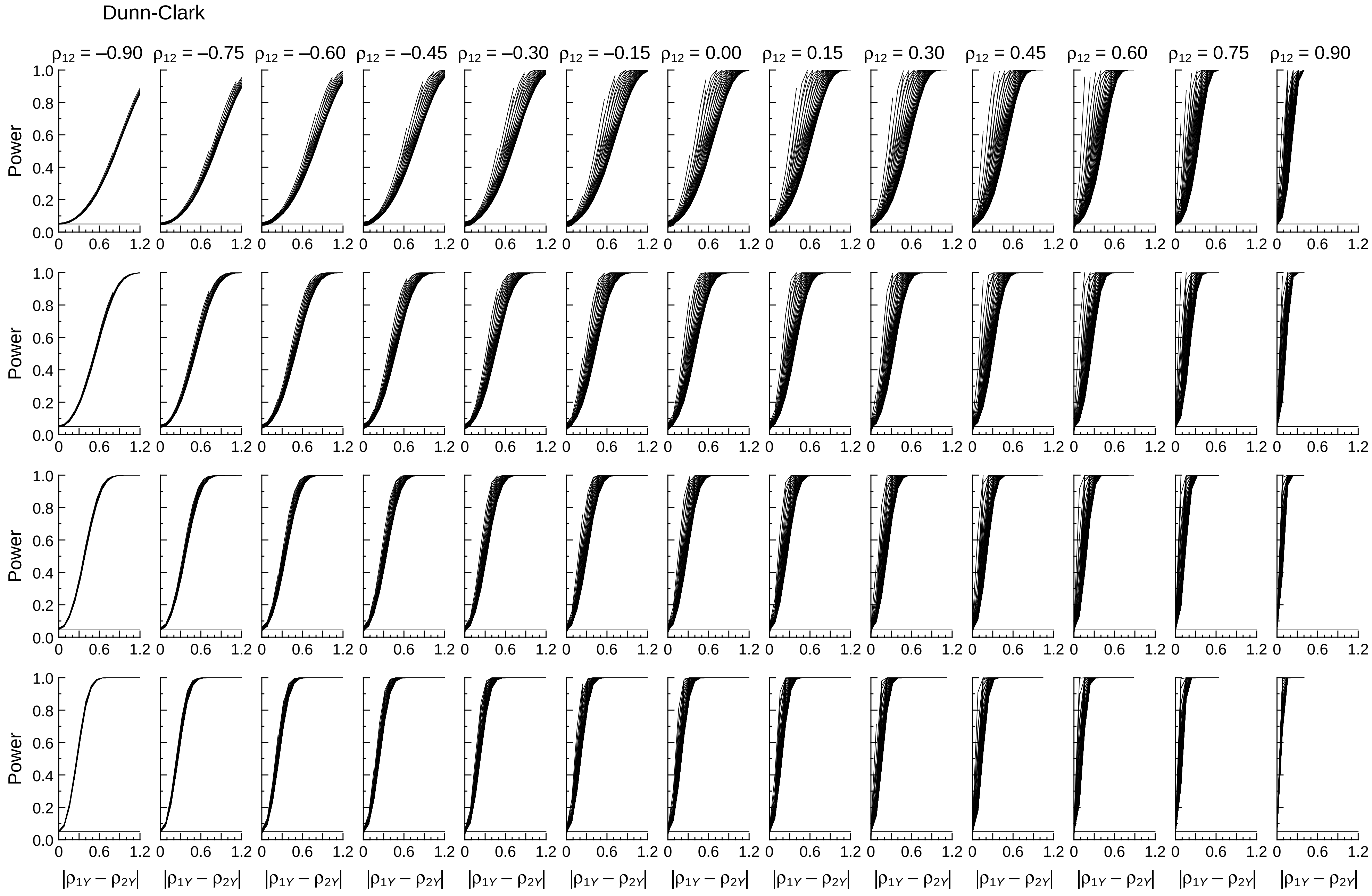

Section F: Power of each test with Beta(2, 5) data (sample size top to bottom: 20, 50, 100, 200)

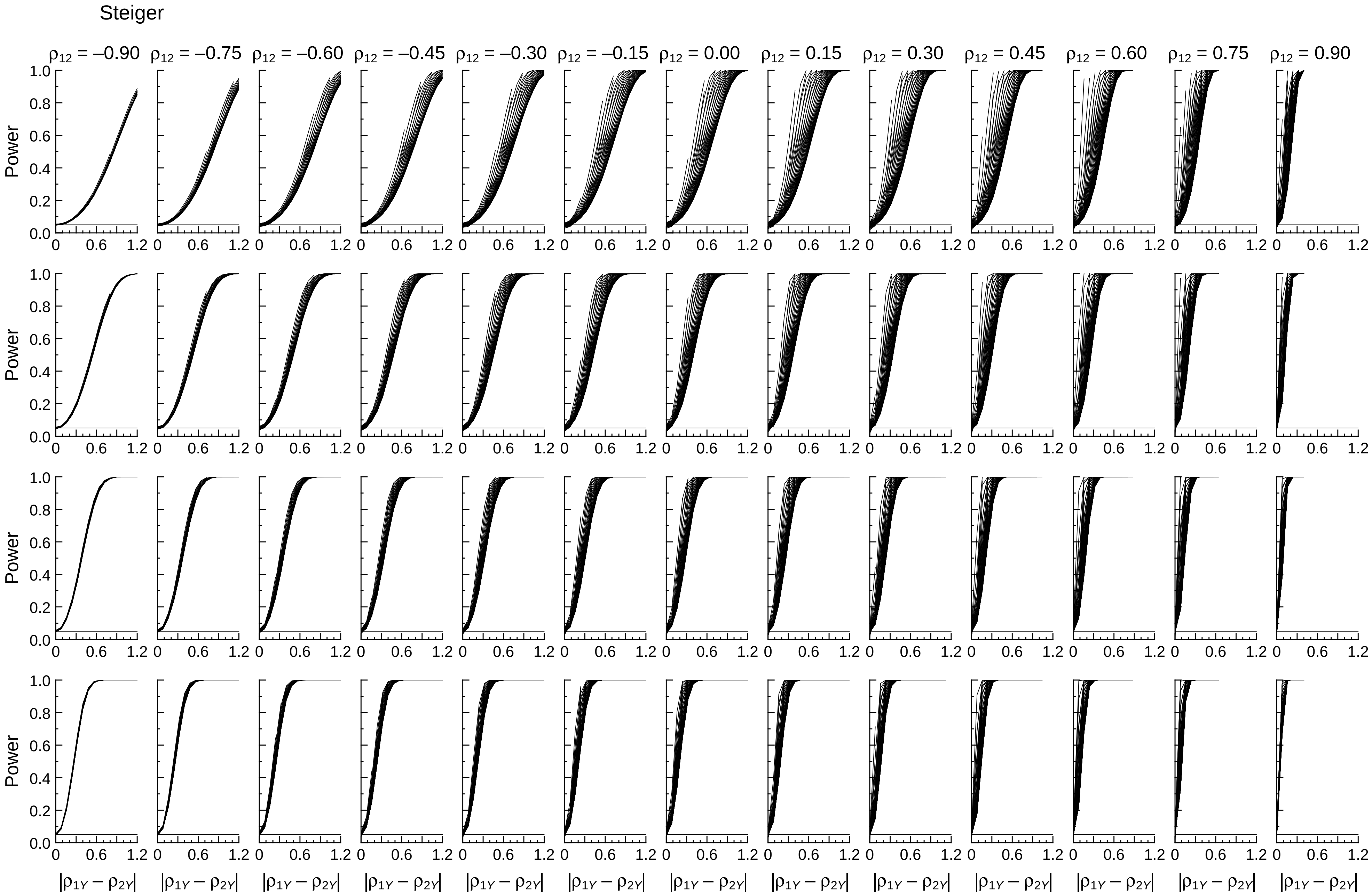

Section F: Power of each test with Beta(2, 5) data (sample size top to bottom: 20, 50, 100, 200)

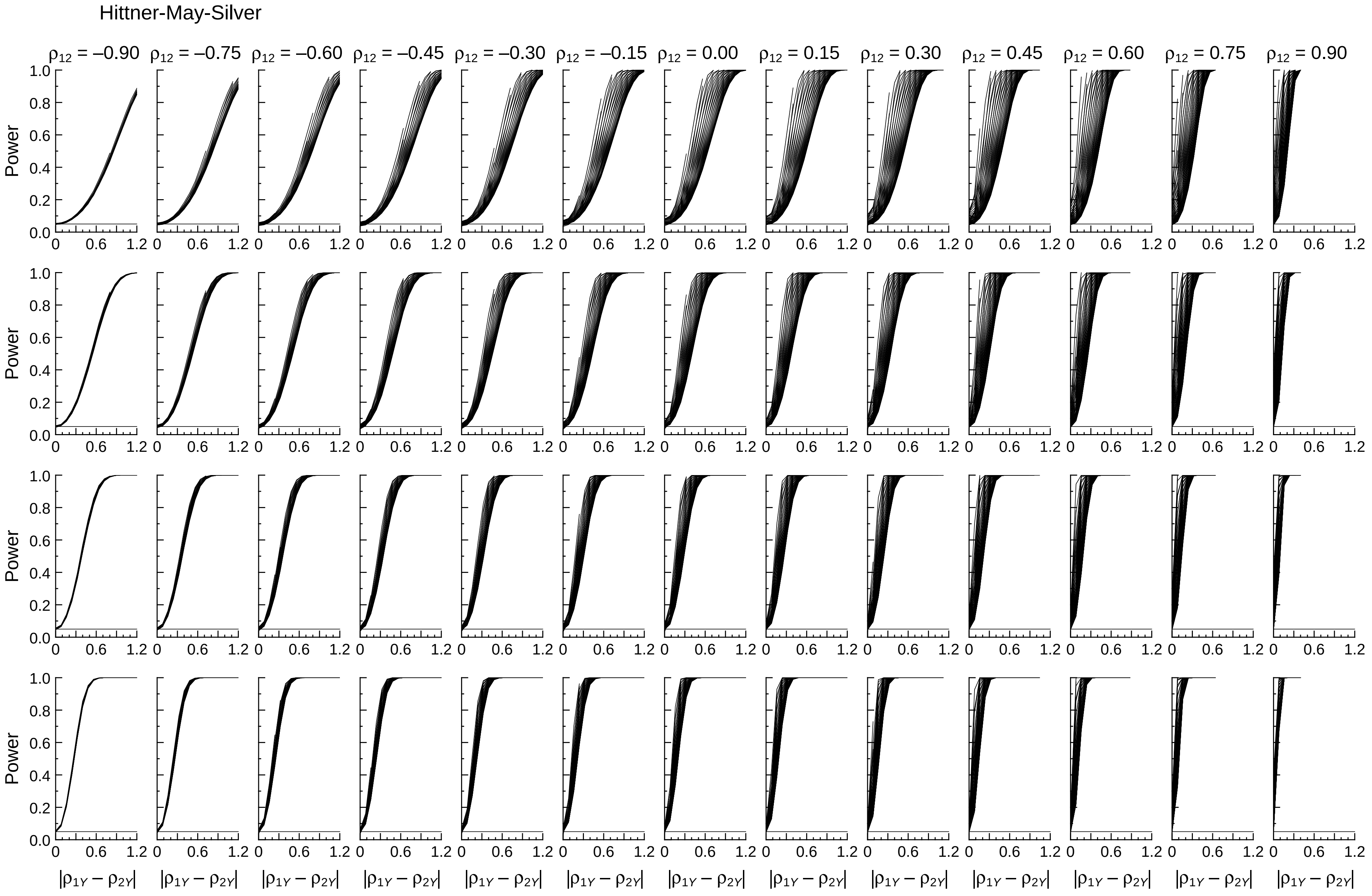

Section F: Power of each test with Beta(2, 5) data (sample size top to bottom: 20, 50, 100, 200)

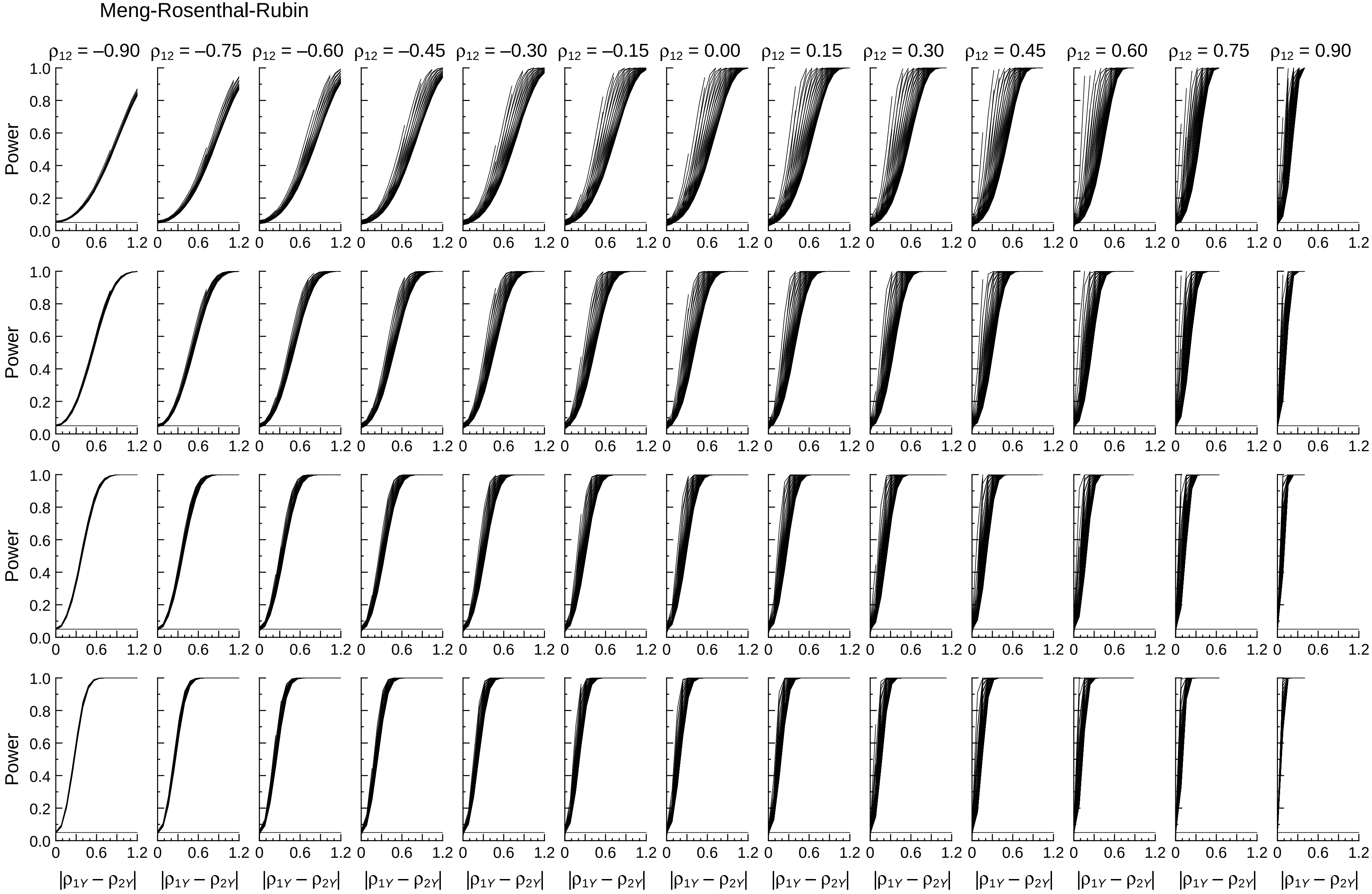

Section F: Power of each test with Beta(2, 5) data (sample size top to bottom: 20, 50, 100, 200)

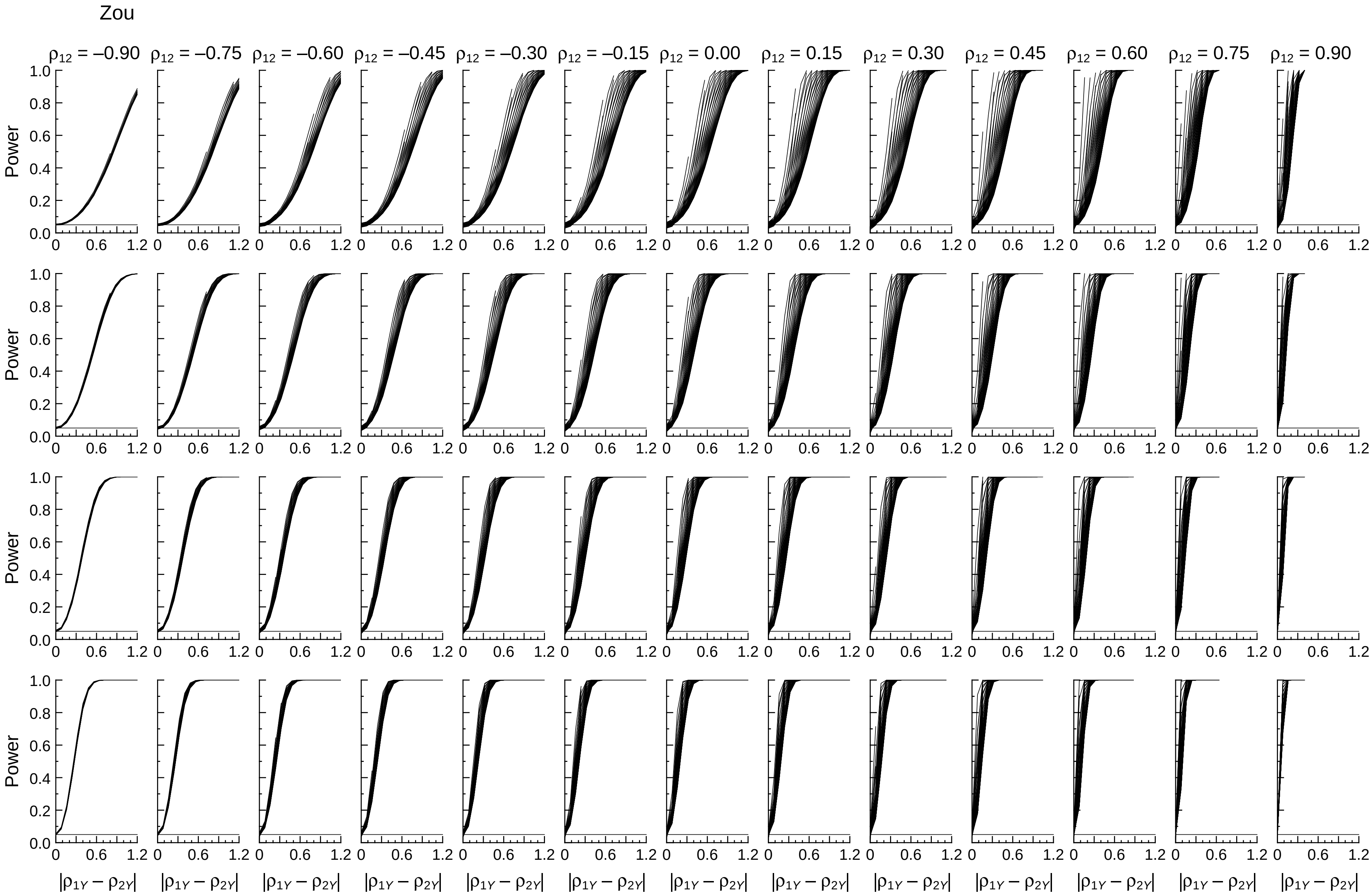

Section G: Type-I error rates of each test with Lognormal(0, 1) data (sample size top to bottom: 20, 50, 100, 200)

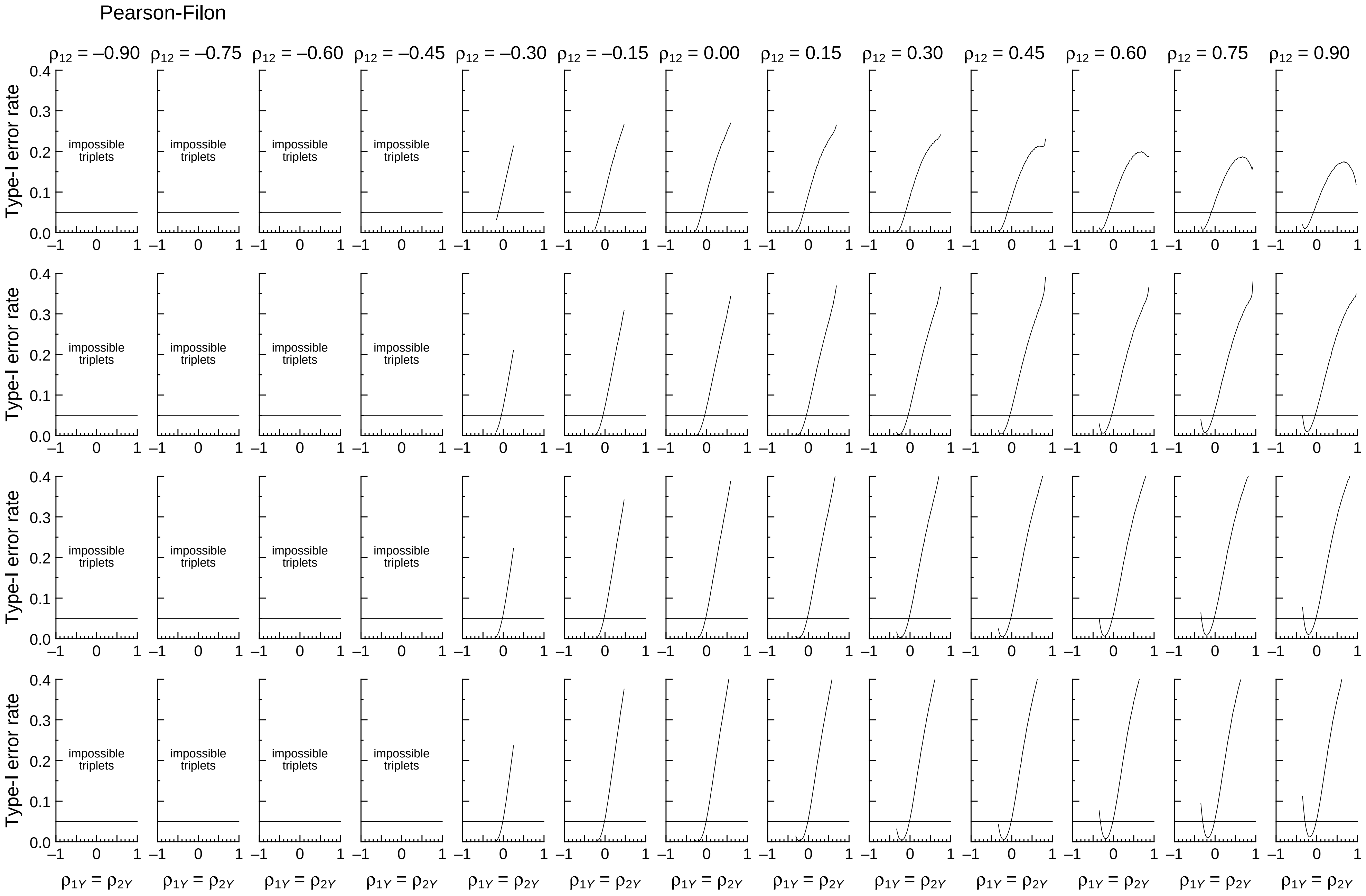

Section G: Type-I error rates of each test with Lognormal(0, 1) data (sample size top to bottom: 20, 50, 100, 200)

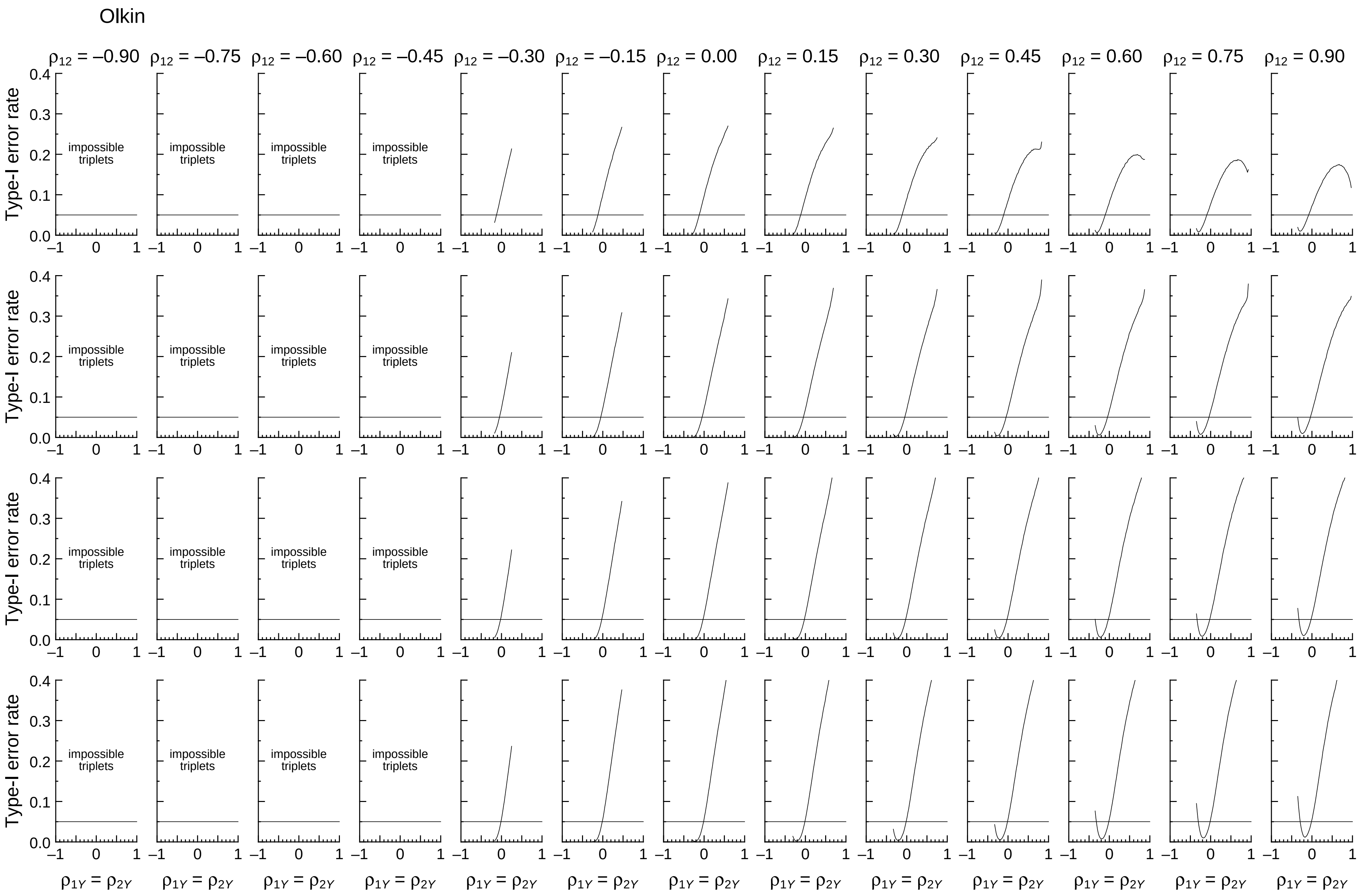

Section G: Type-I error rates of each test with Lognormal(0, 1) data (sample size top to bottom: 20, 50, 100, 200)

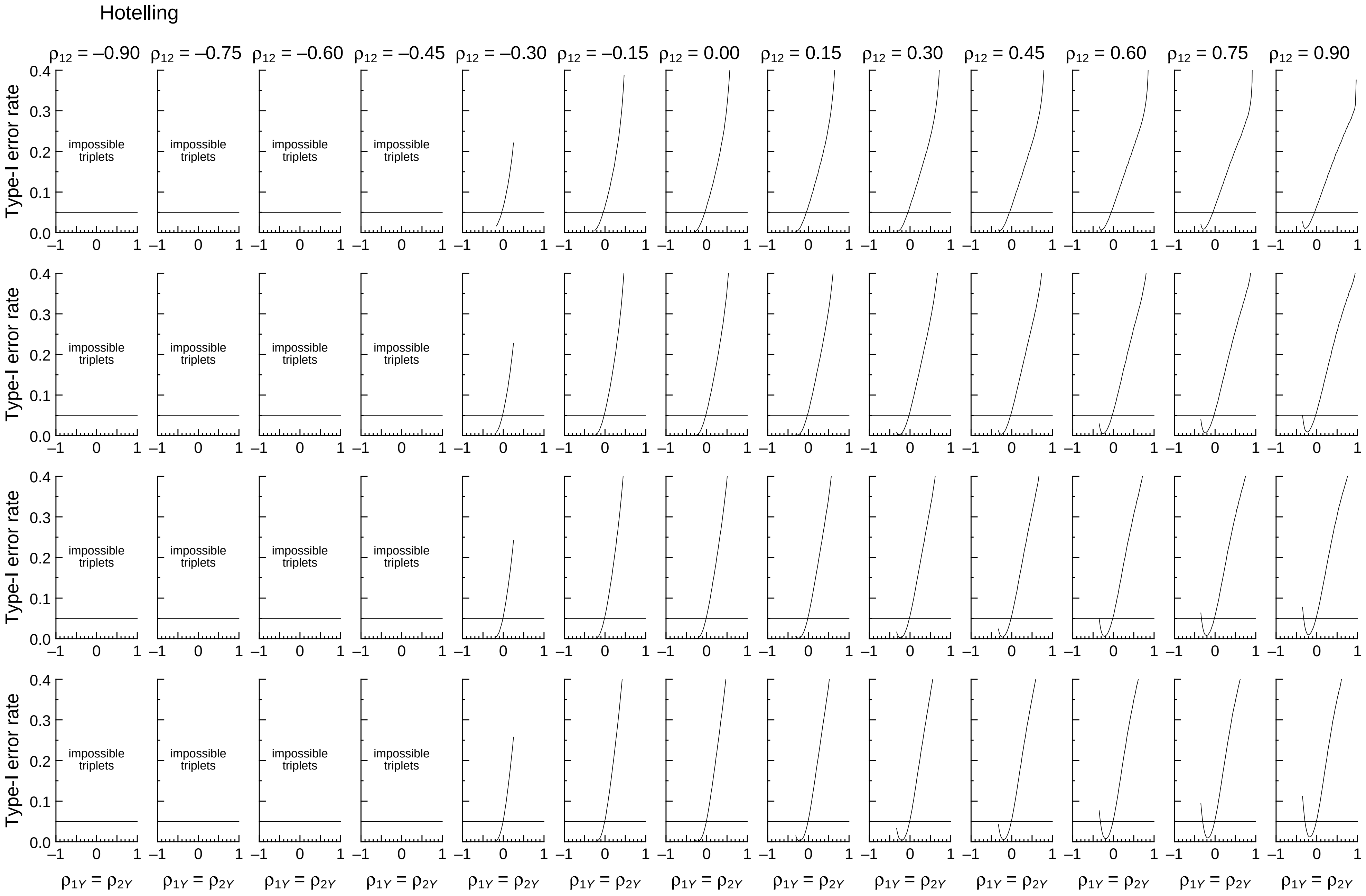

Section G: Type-I error rates of each test with Lognormal(0, 1) data (sample size top to bottom: 20, 50, 100, 200)

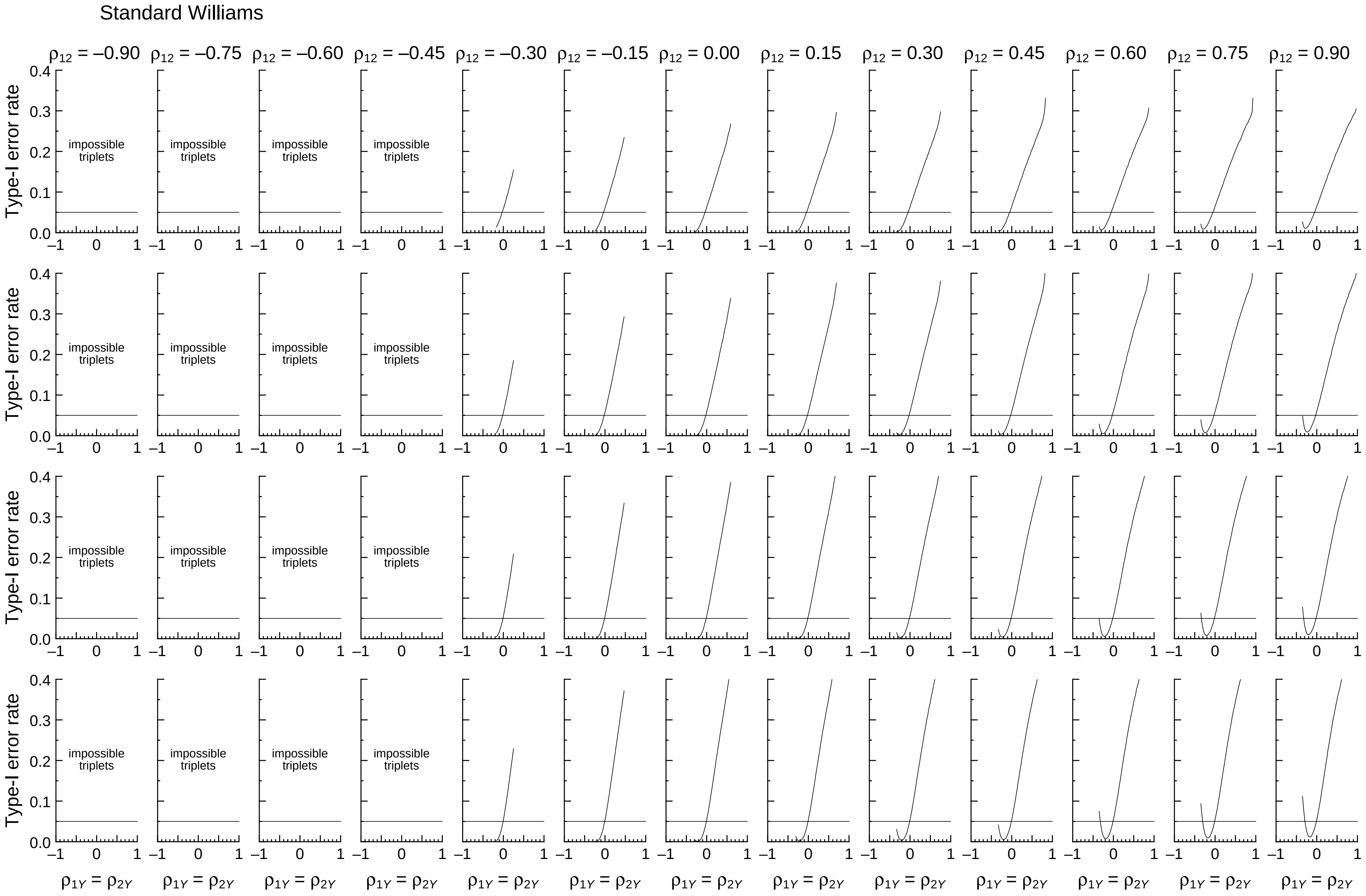

Section G: Type-I error rates of each test with Lognormal(0, 1) data (sample size top to bottom: 20, 50, 100, 200)

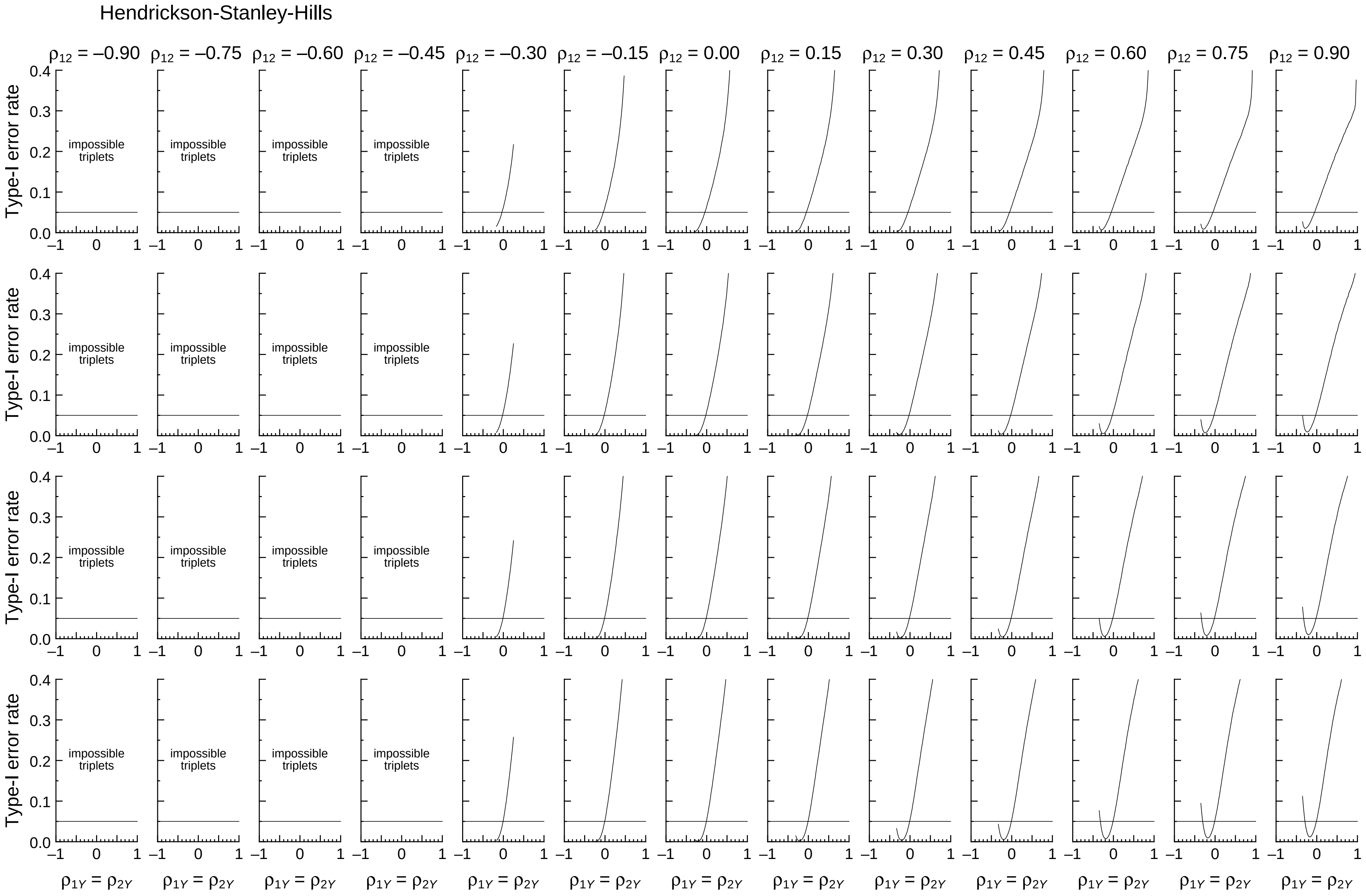

Section G: Type-I error rates of each test with Lognormal(0, 1) data (sample size top to bottom: 20, 50, 100, 200)

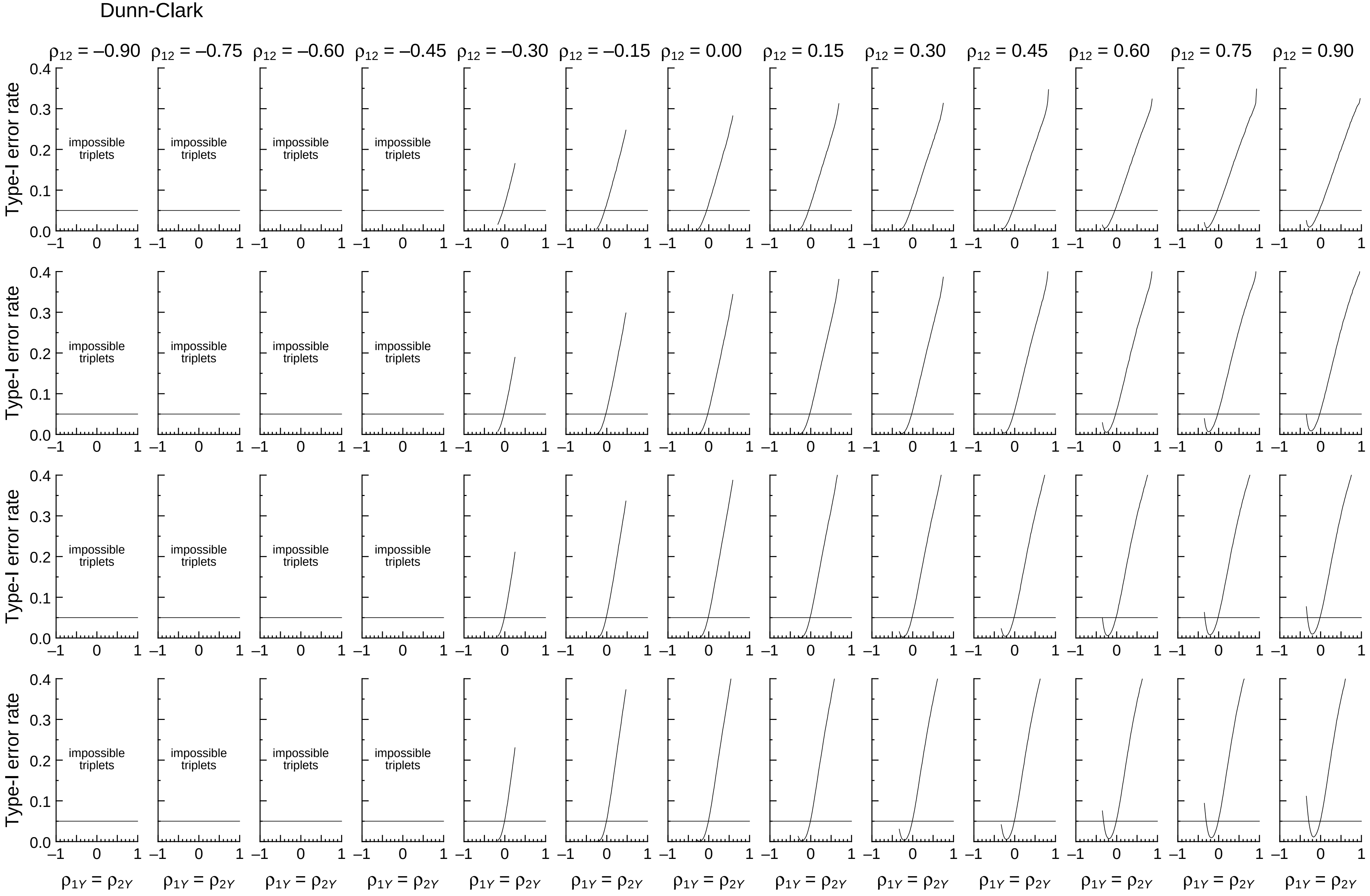

Section G: Type-I error rates of each test with Lognormal(0, 1) data (sample size top to bottom: 20, 50, 100, 200)

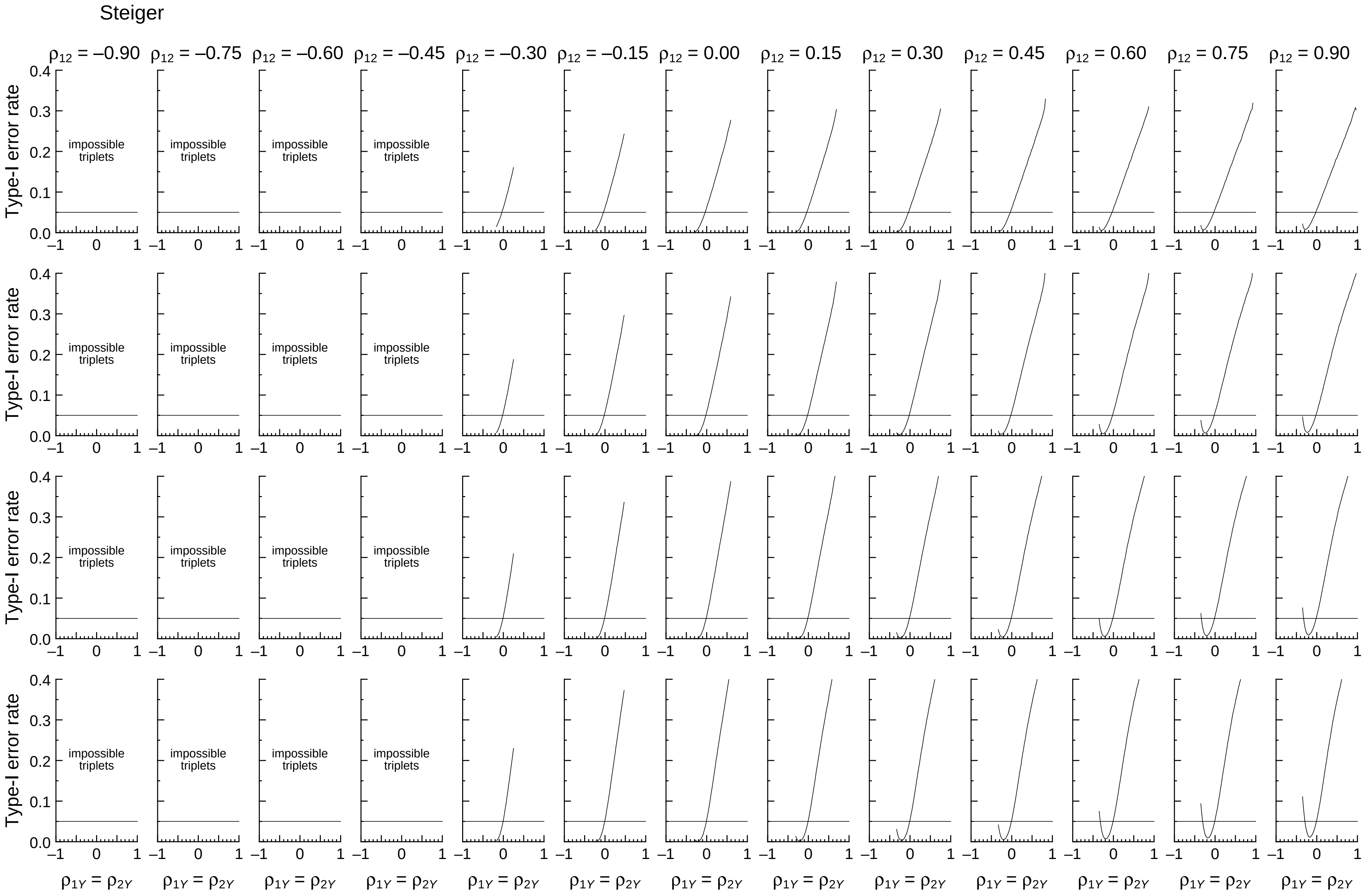

Section G: Type-I error rates of each test with Lognormal(0, 1) data (sample size top to bottom: 20, 50, 100, 200)

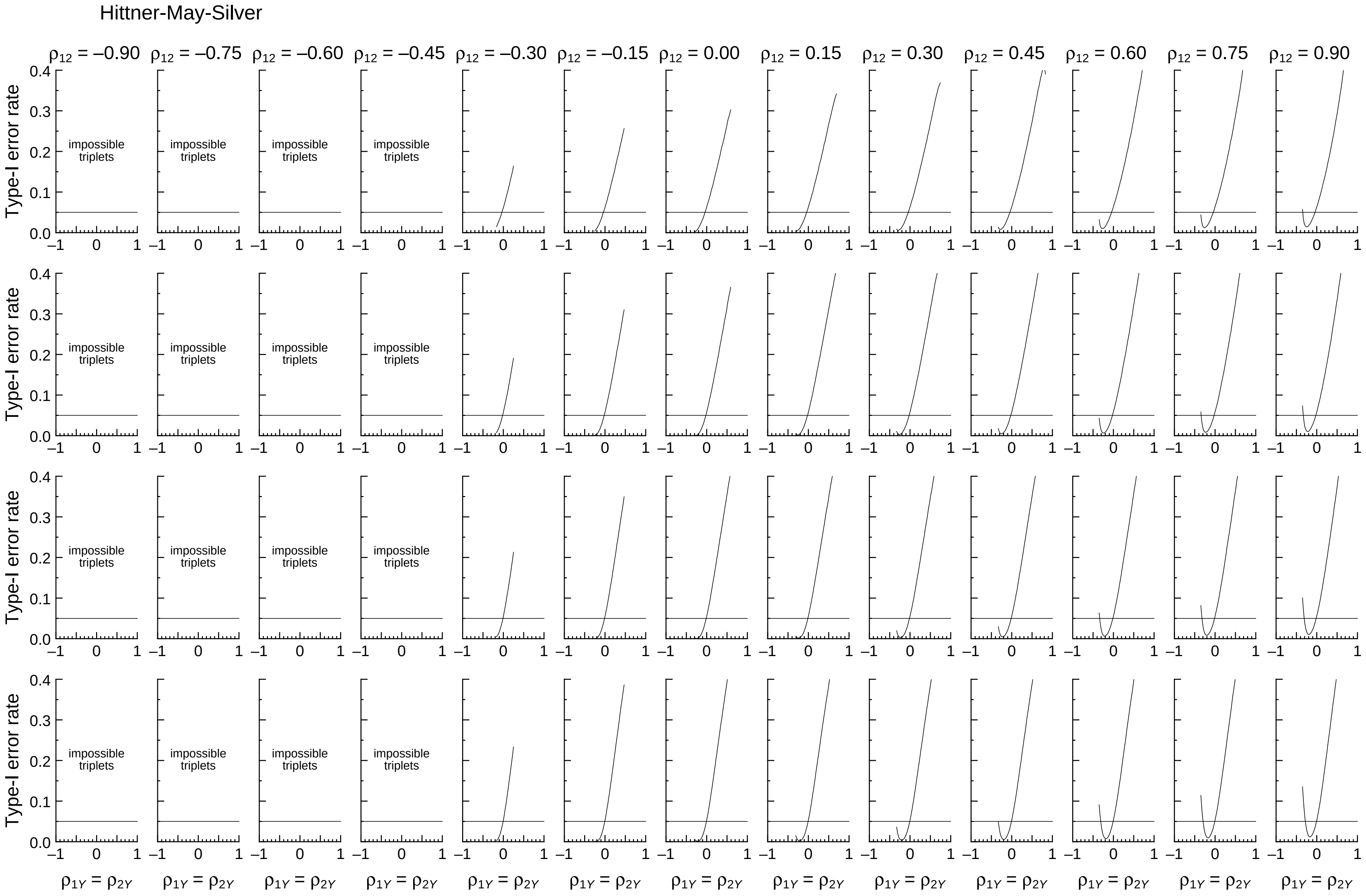

Section G: Type-I error rates of each test with Lognormal(0, 1) data (sample size top to bottom: 20, 50, 100, 200)

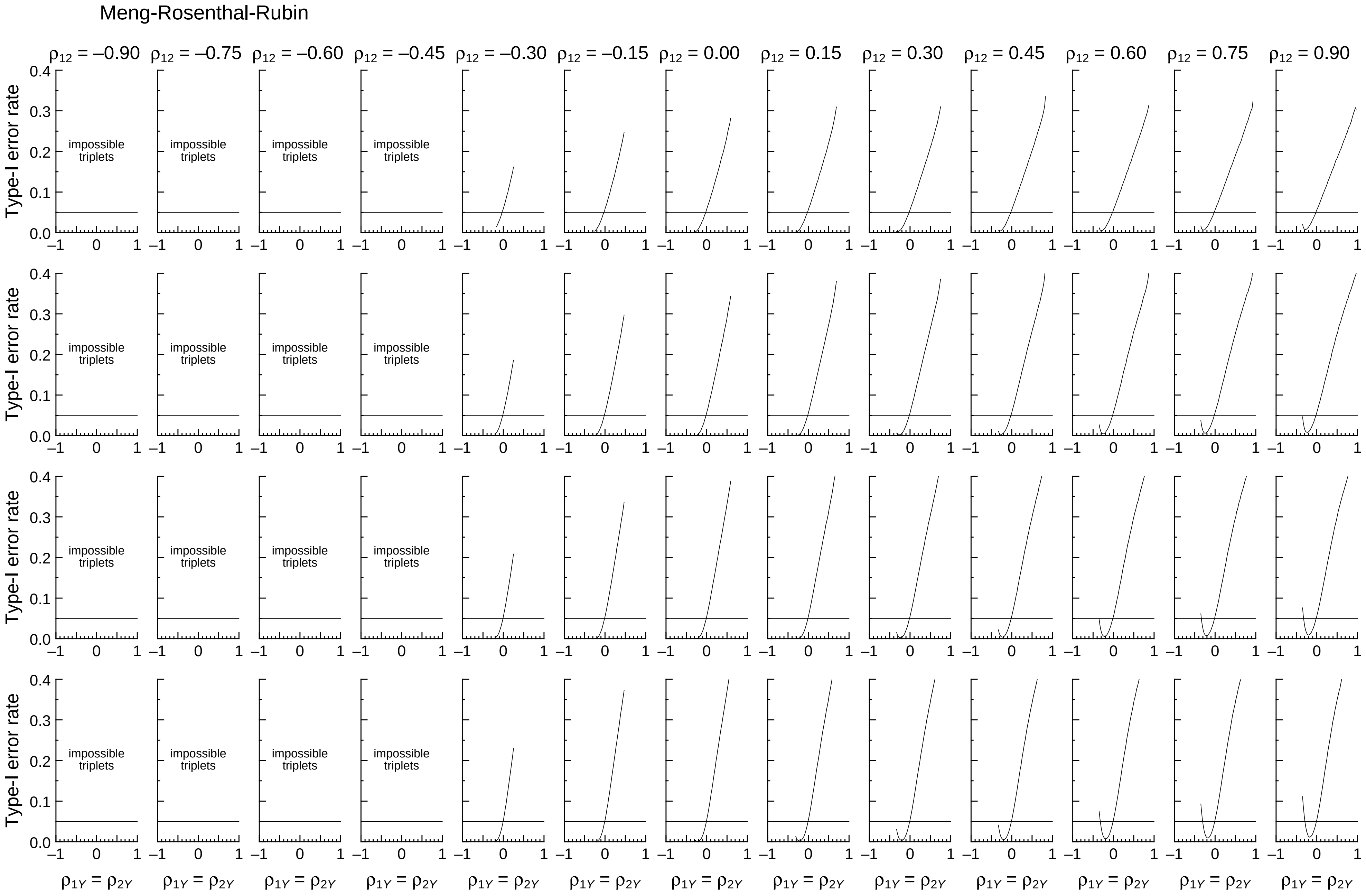

Section G: Type-I error rates of each test with Lognormal(0, 1) data (sample size top to bottom: 20, 50, 100, 200)

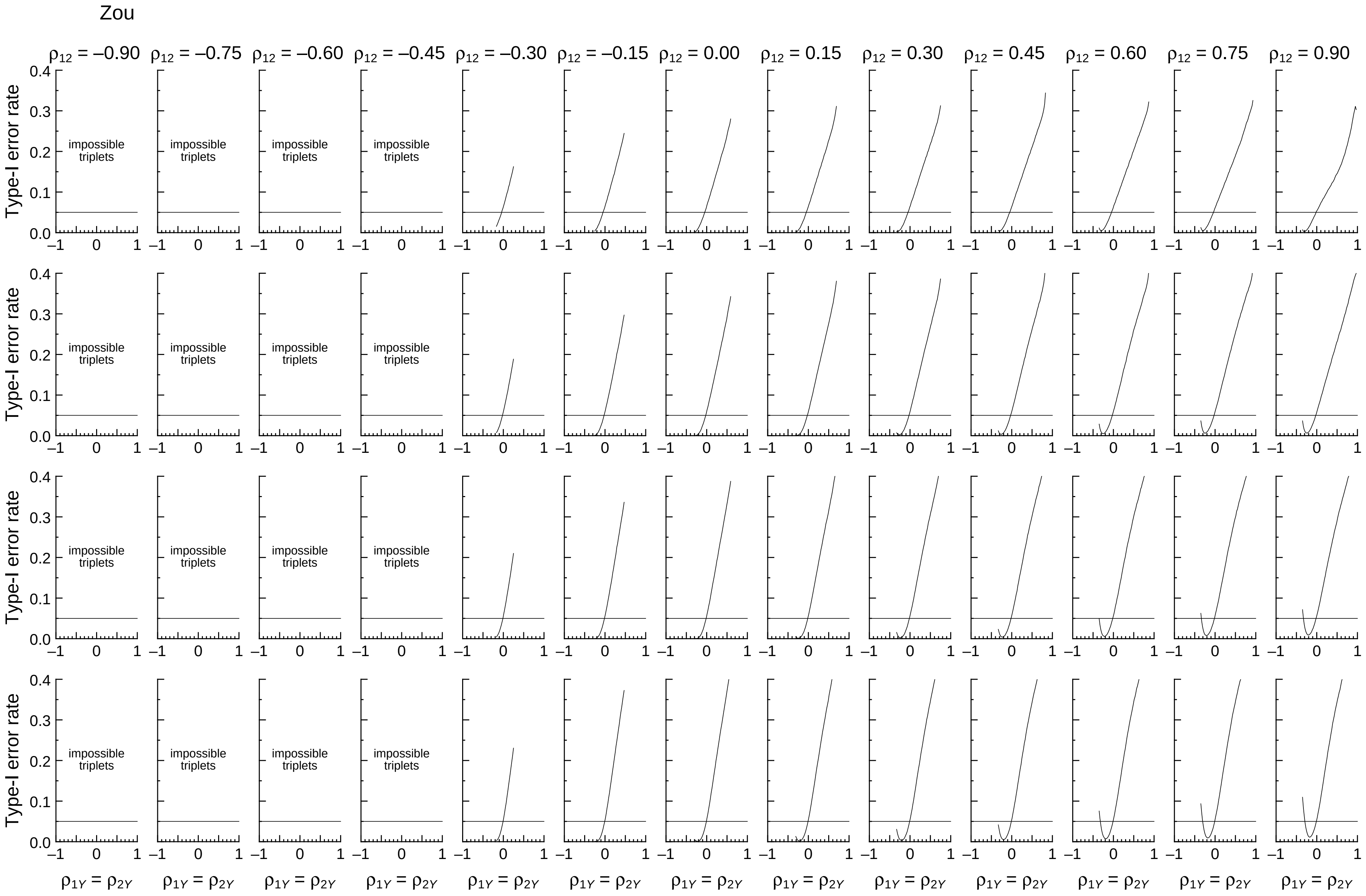

Section H: Type-I error rates of each test with mixture 0.9 N(0, 1) + 0.1 N(0, 2) data (sample size top to bottom: 20, 50, 100, 200)

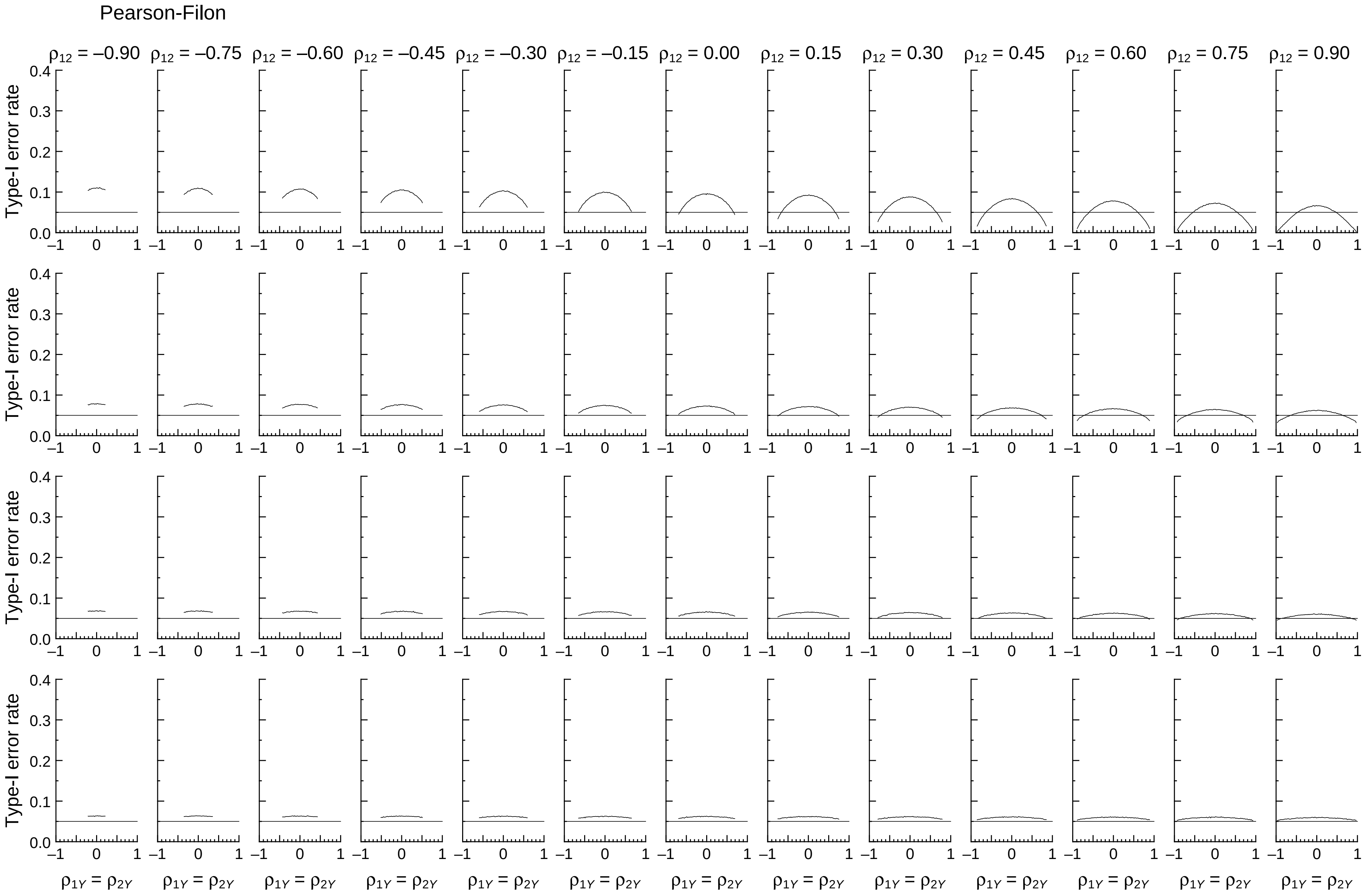

Section H: Type-I error rates of each test with mixture 0.9 N(0, 1) + 0.1 N(0, 2) data (sample size top to bottom: 20, 50, 100, 200)

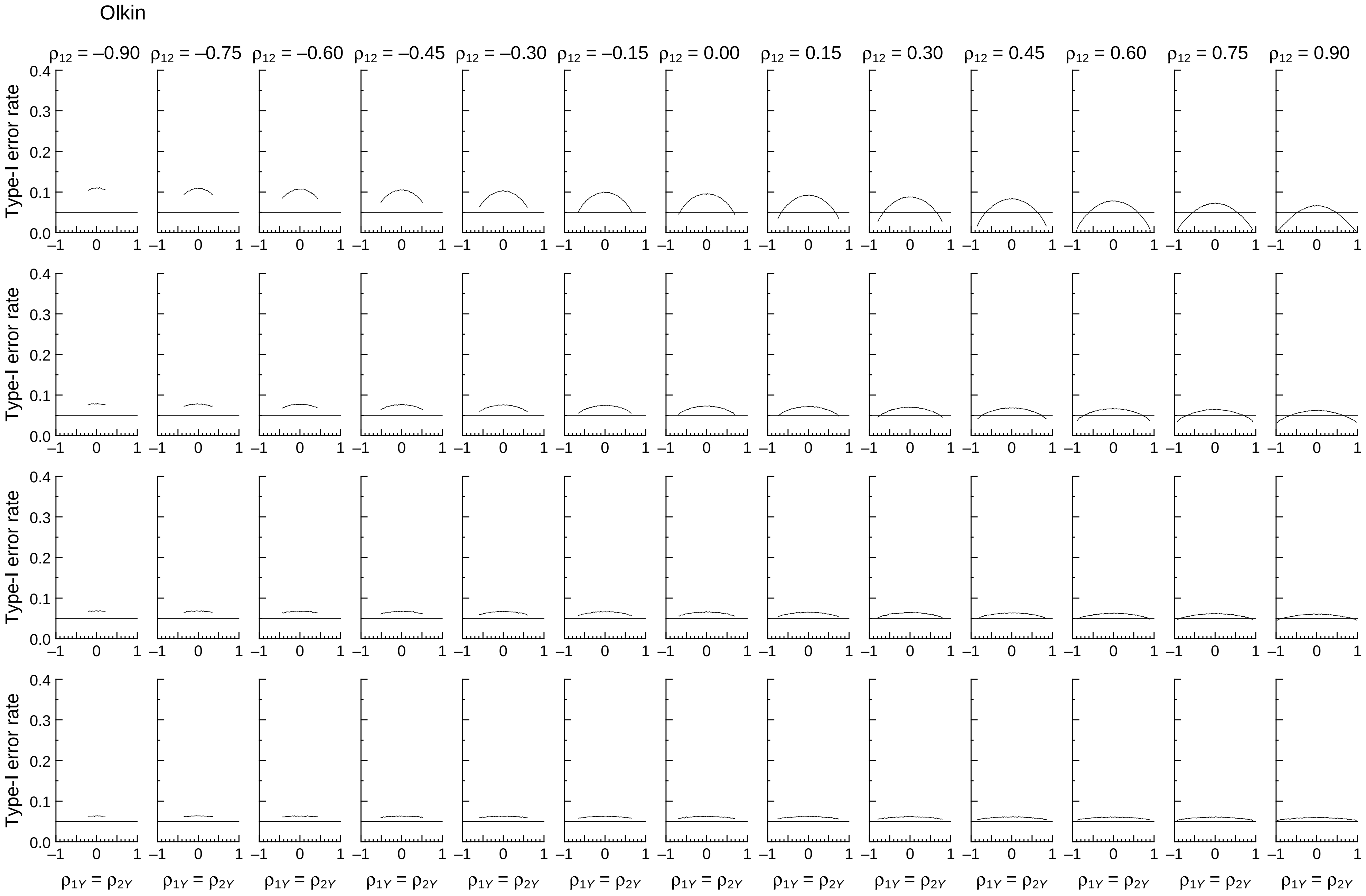

Section H: Type-I error rates of each test with mixture 0.9 N(0, 1) + 0.1 N(0, 2) data (sample size top to bottom: 20, 50, 100, 200)

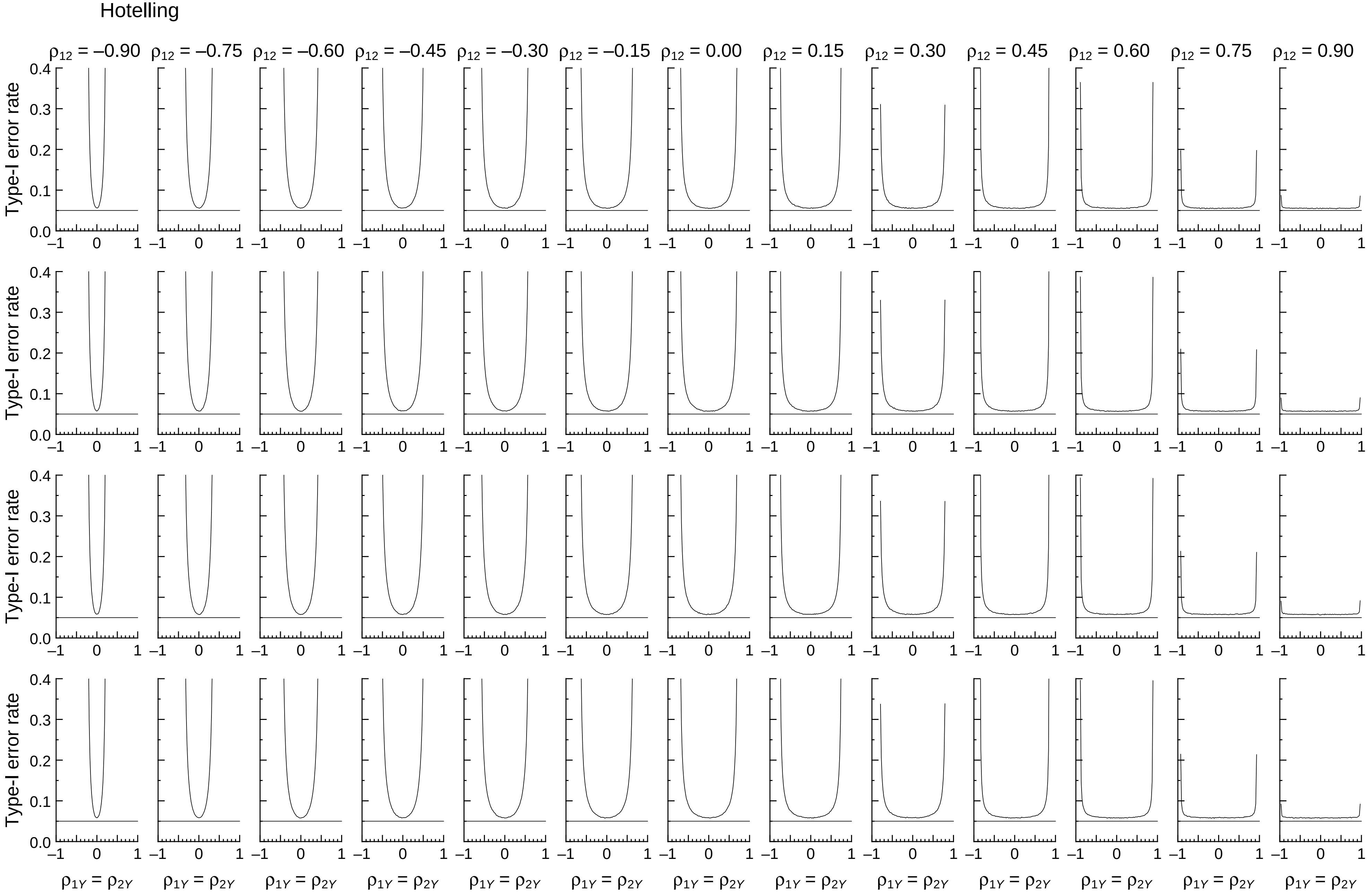

Section H: Type-I error rates of each test with mixture 0.9 N(0, 1) + 0.1 N(0, 2) data (sample size top to bottom: 20, 50, 100, 200)

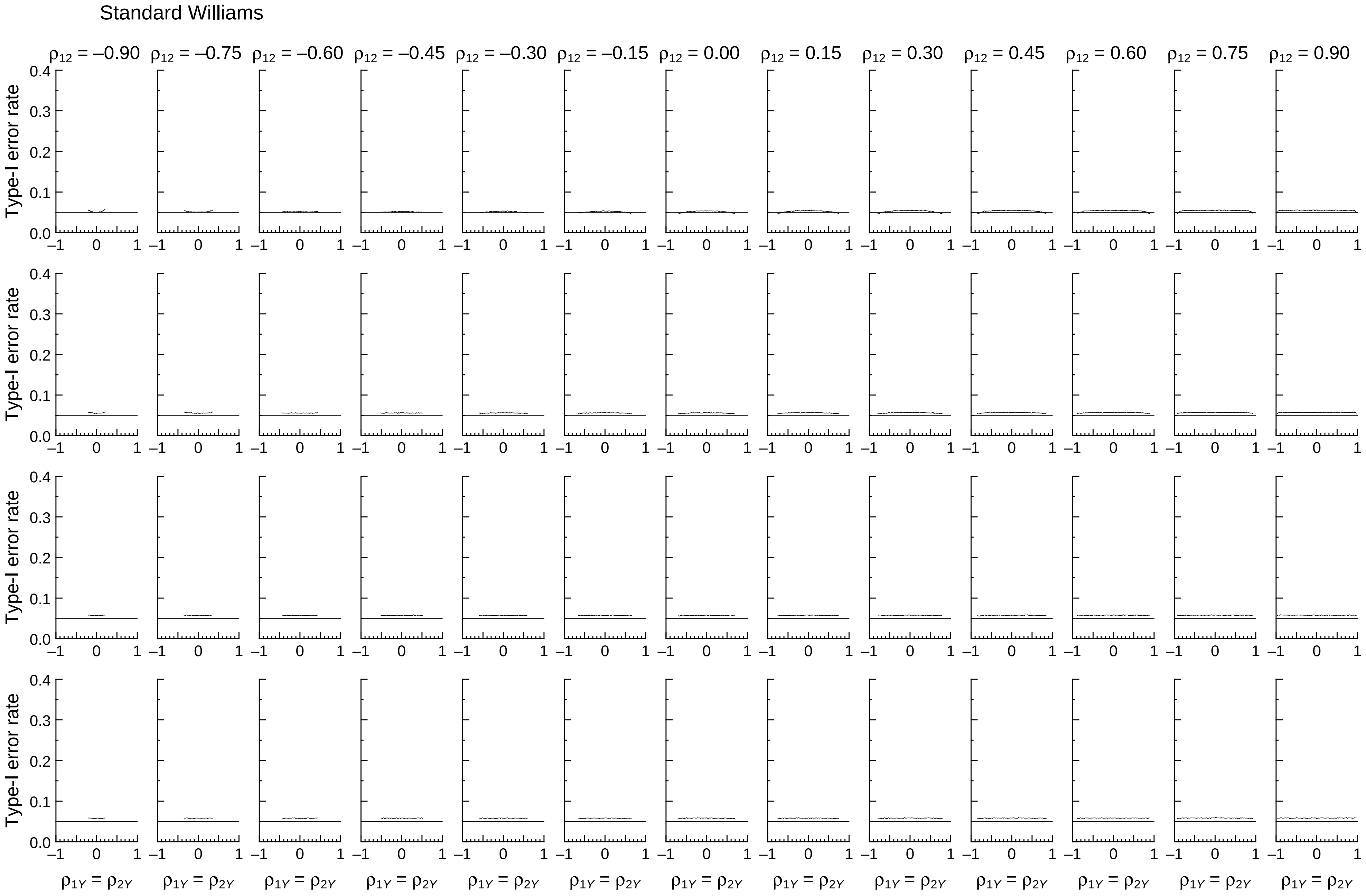

Section H: Type-I error rates of each test with mixture 0.9 N(0, 1) + 0.1 N(0, 2) data (sample size top to bottom: 20, 50, 100, 200)

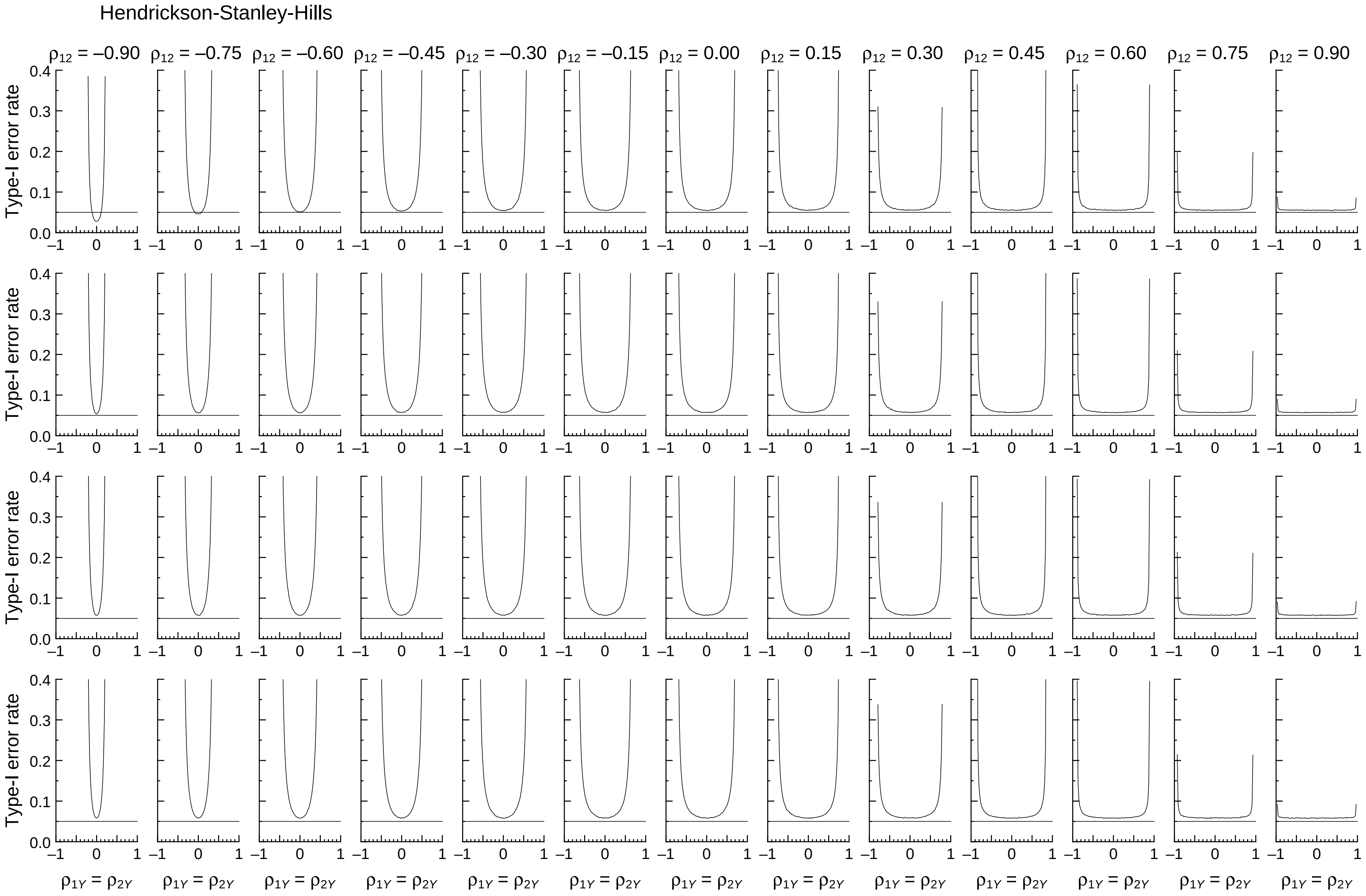

Section H: Type-I error rates of each test with mixture 0.9 N(0, 1) + 0.1 N(0, 2) data (sample size top to bottom: 20, 50, 100, 200)

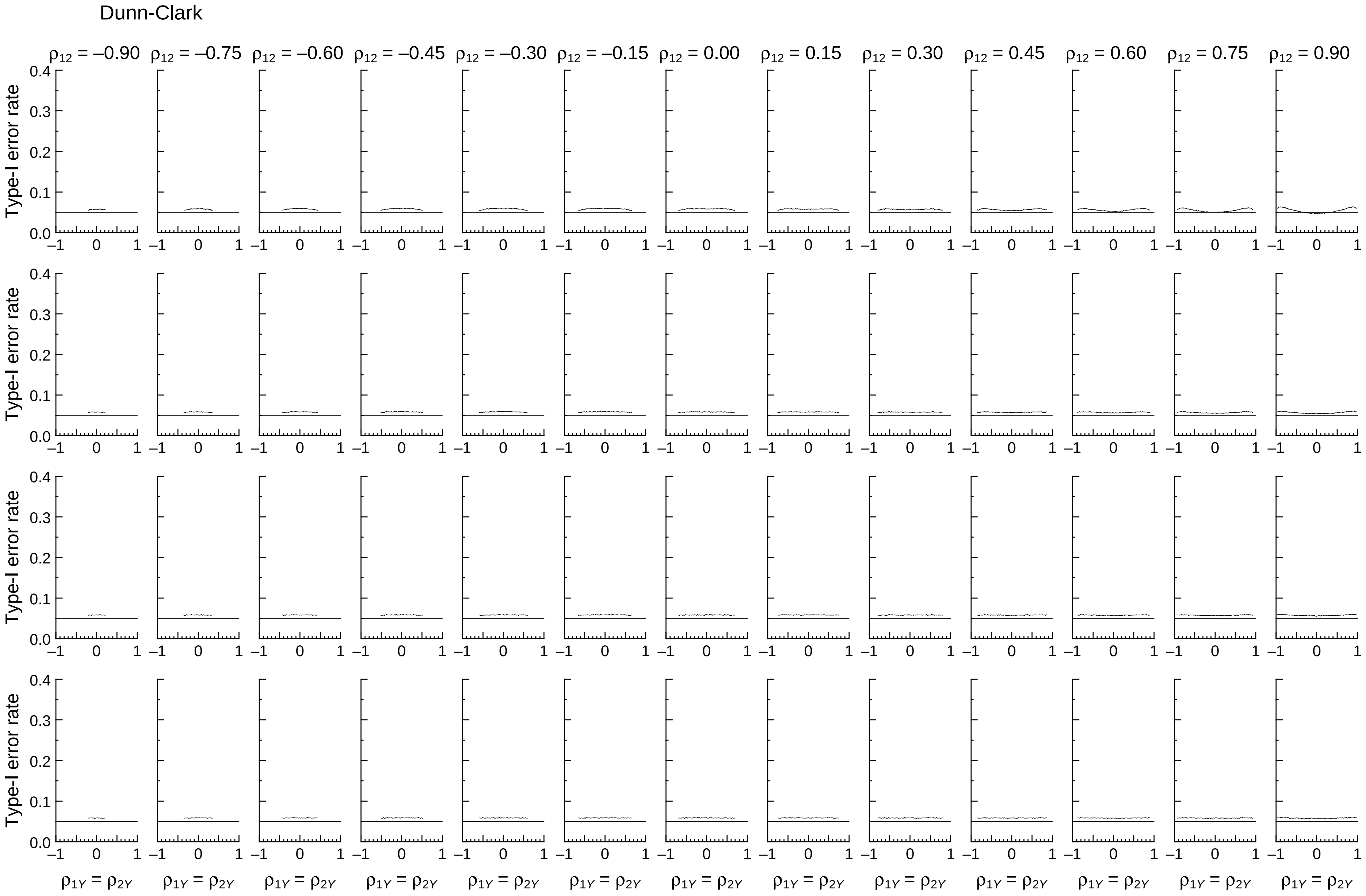

Section H: Type-I error rates of each test with mixture 0.9 N(0, 1) + 0.1 N(0, 2) data (sample size top to bottom: 20, 50, 100, 200)

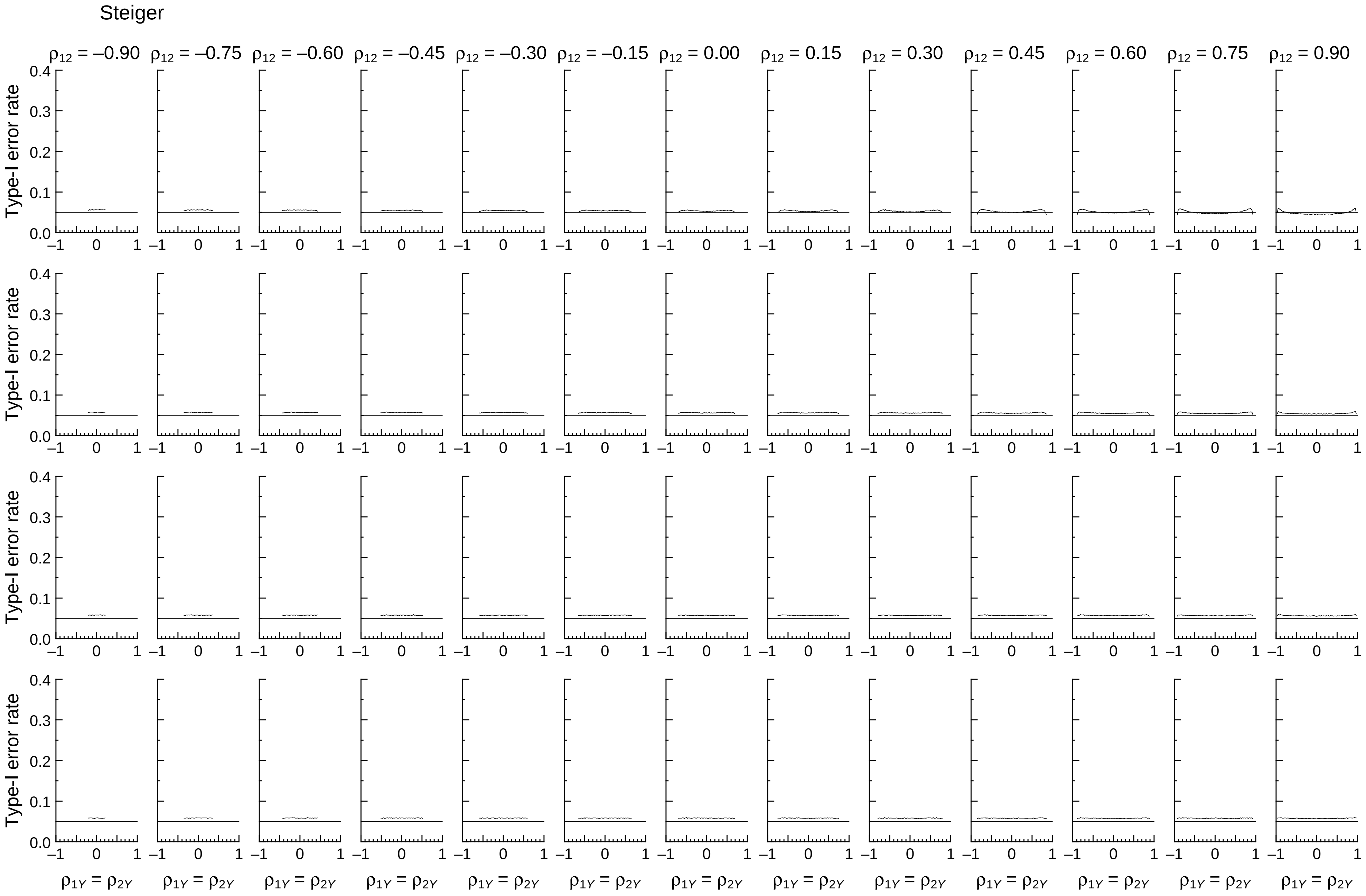

Section H: Type-I error rates of each test with mixture 0.9 N(0, 1) + 0.1 N(0, 2) data (sample size top to bottom: 20, 50, 100, 200)

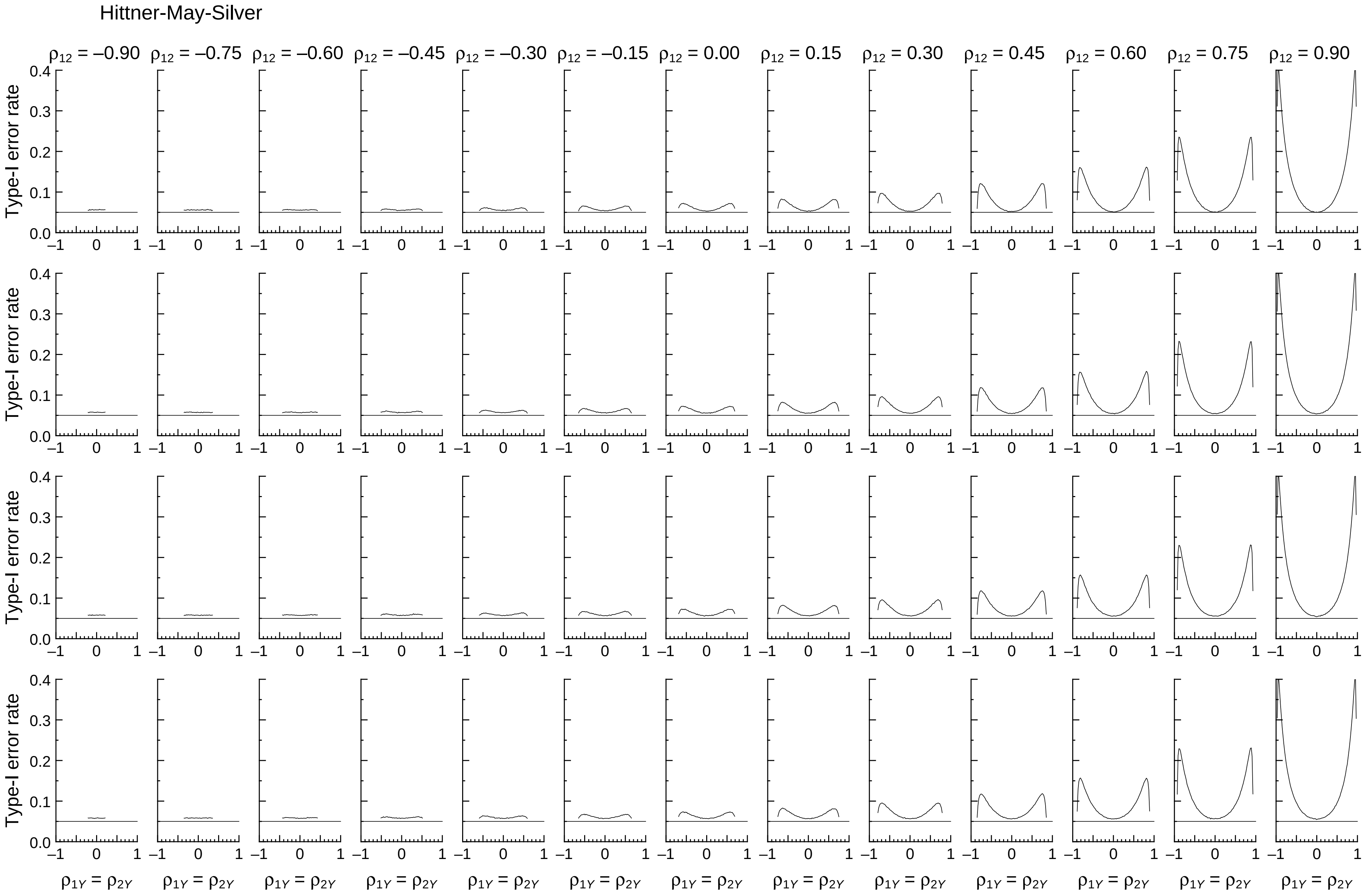

Section H: Type-I error rates of each test with mixture 0.9 N(0, 1) + 0.1 N(0, 2) data (sample size top to bottom: 20, 50, 100, 200)

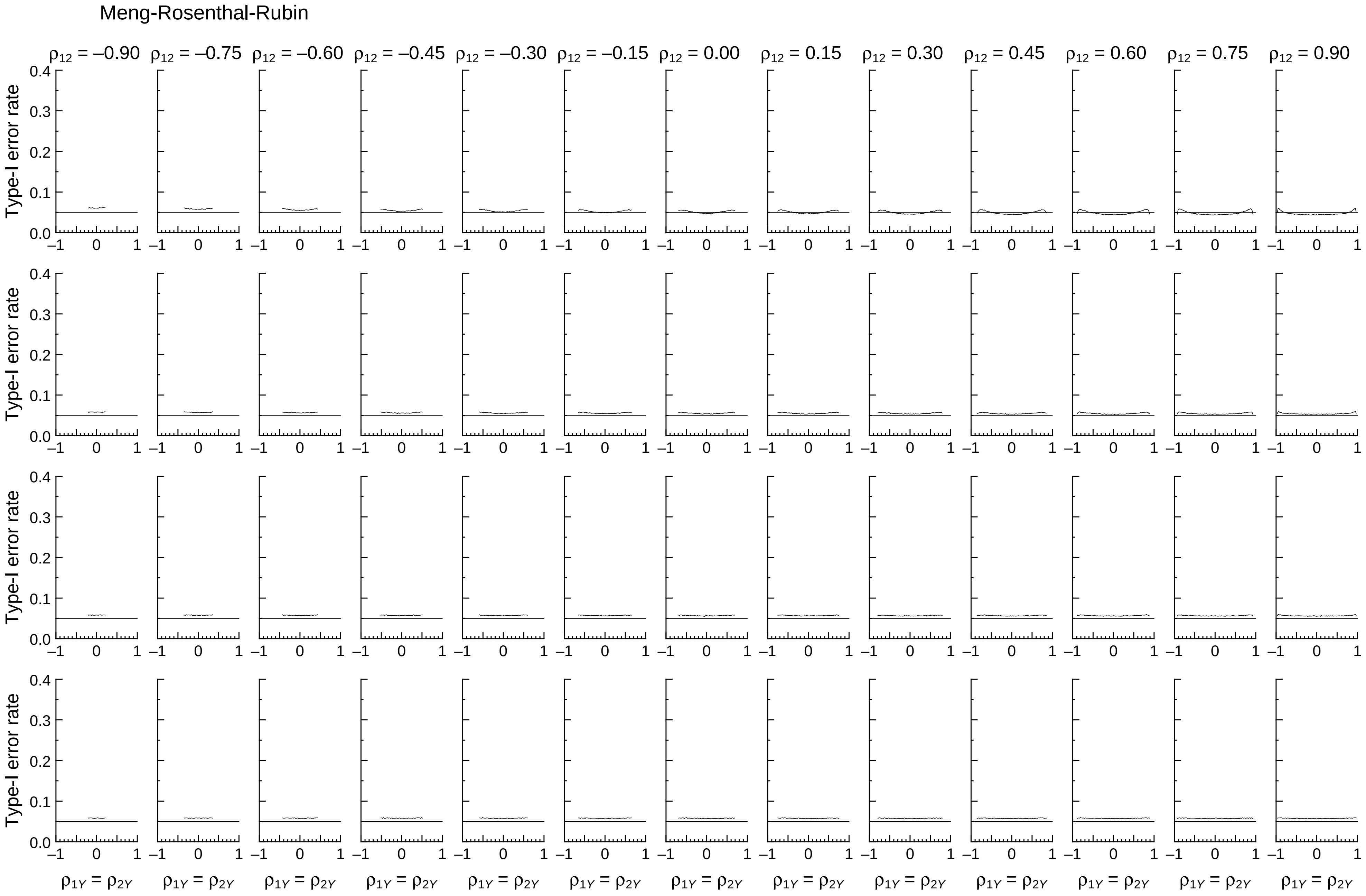

Section H: Type-I error rates of each test with mixture 0.9 N(0, 1) + 0.1 N(0, 2) data (sample size top to bottom: 20, 50, 100, 200)

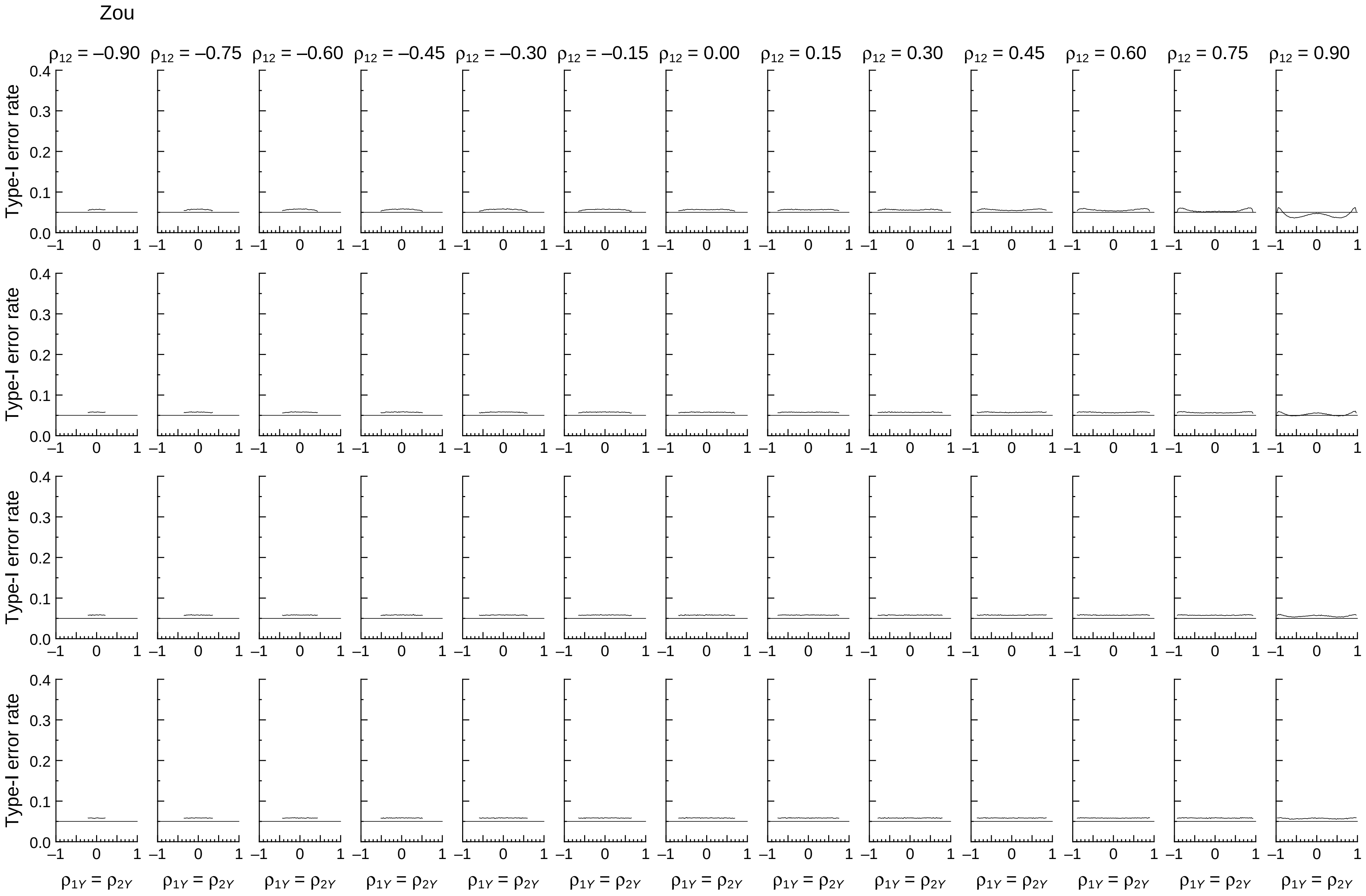

Section I: Type-I error rates of each test with mixture 0.9 N(0, 1) + 0.1 N(0, 4) data (sample size top to bottom: 20, 50, 100, 200)

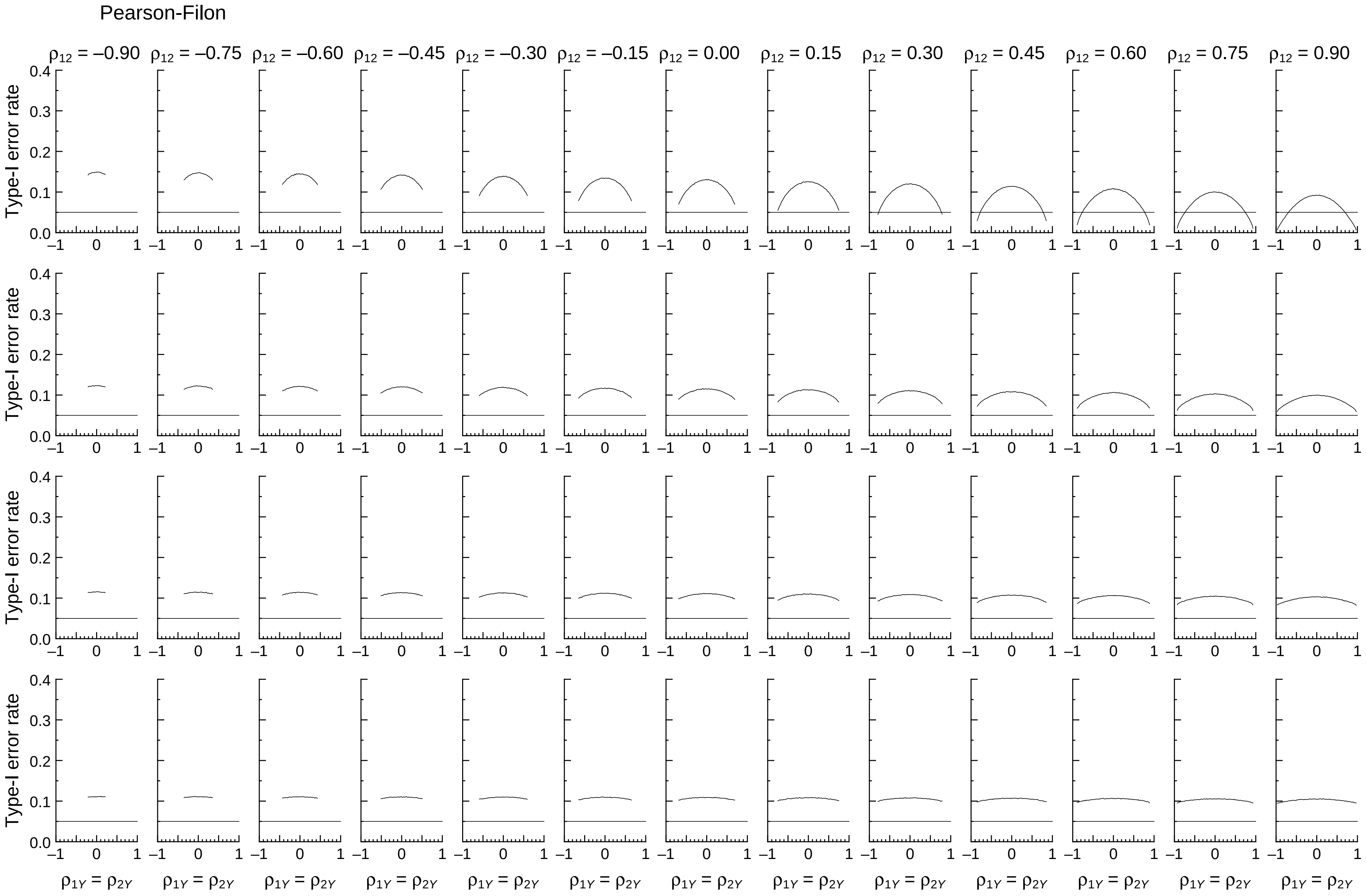

Section I: Type-I error rates of each test with mixture 0.9 N(0, 1) + 0.1 N(0, 4) data (sample size top to bottom: 20, 50, 100, 200)

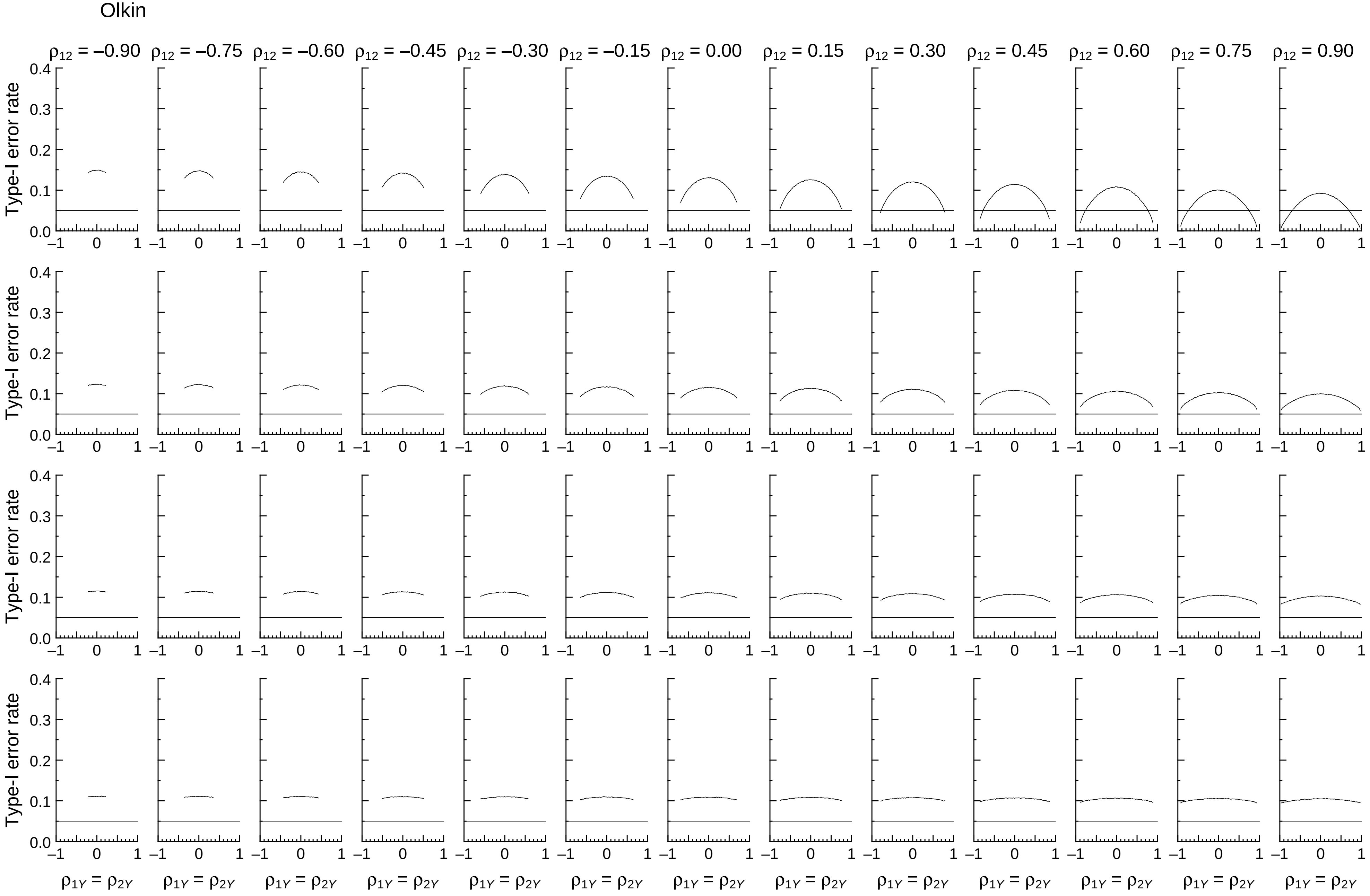

Section I: Type-I error rates of each test with mixture 0.9 N(0, 1) + 0.1 N(0, 4) data (sample size top to bottom: 20, 50, 100, 200)

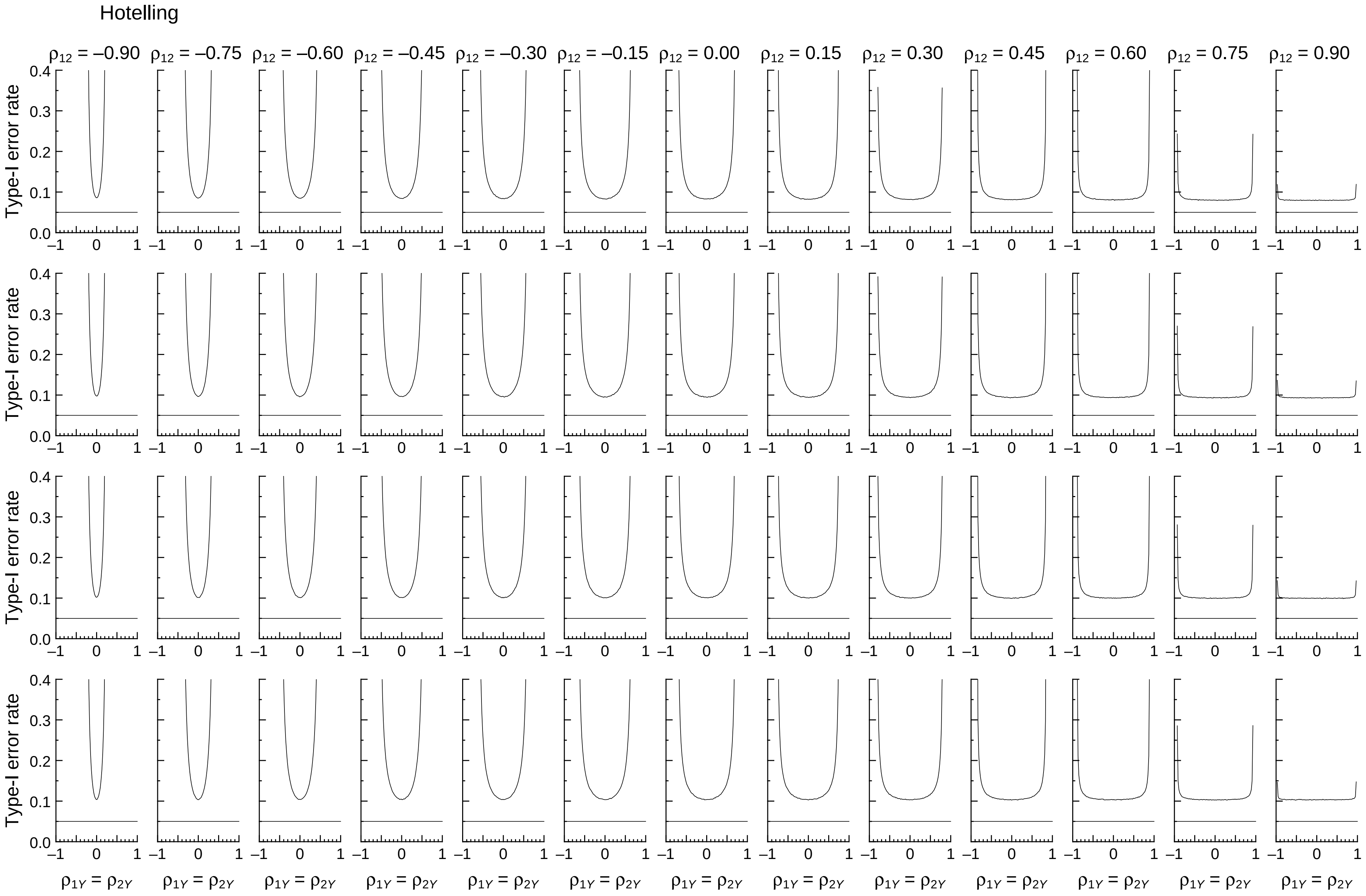

Section I: Type-I error rates of each test with mixture 0.9 N(0, 1) + 0.1 N(0, 4) data (sample size top to bottom: 20, 50, 100, 200)

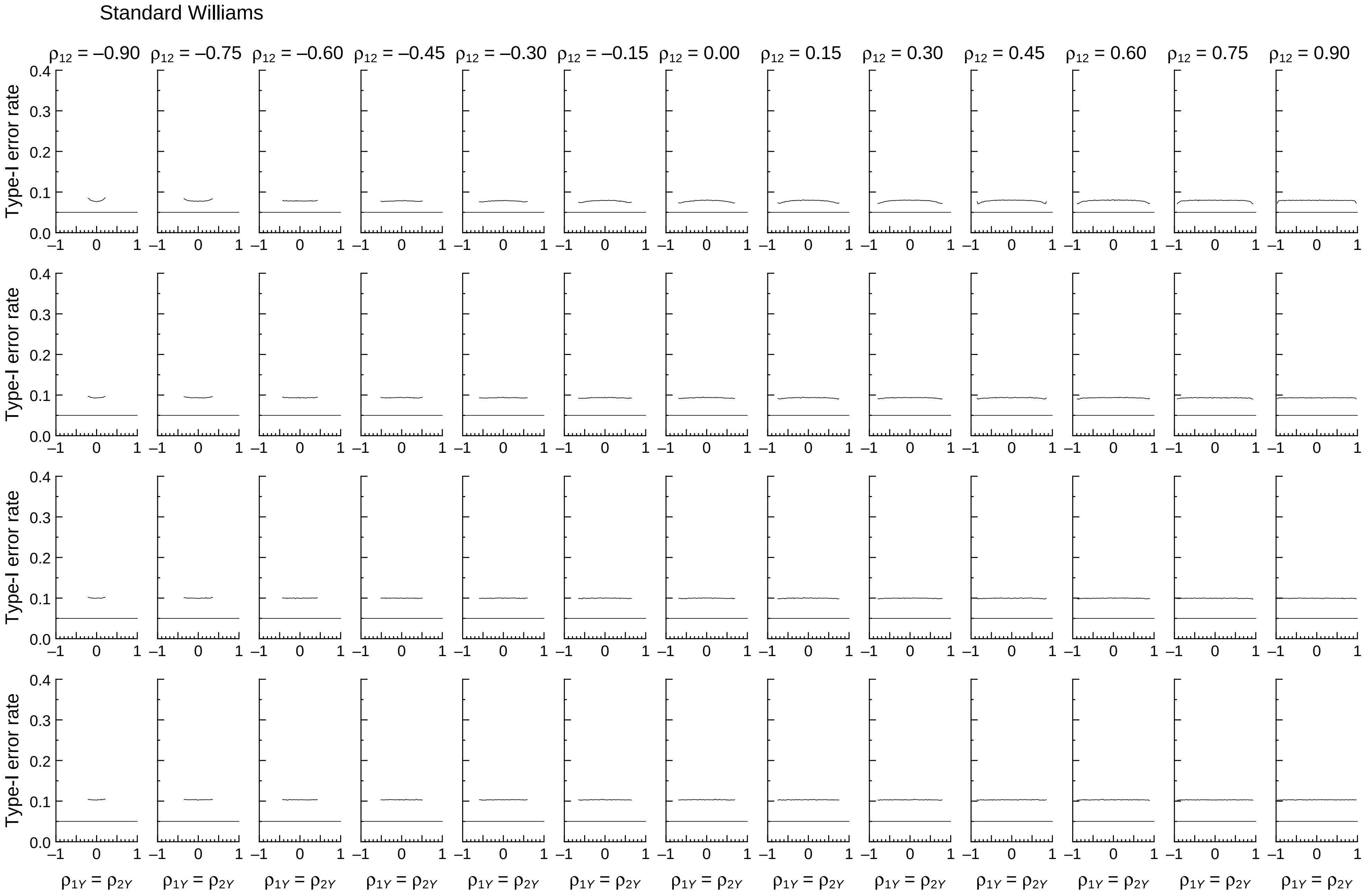

Section I: Type-I error rates of each test with mixture 0.9 N(0, 1) + 0.1 N(0, 4) data (sample size top to bottom: 20, 50, 100, 200)

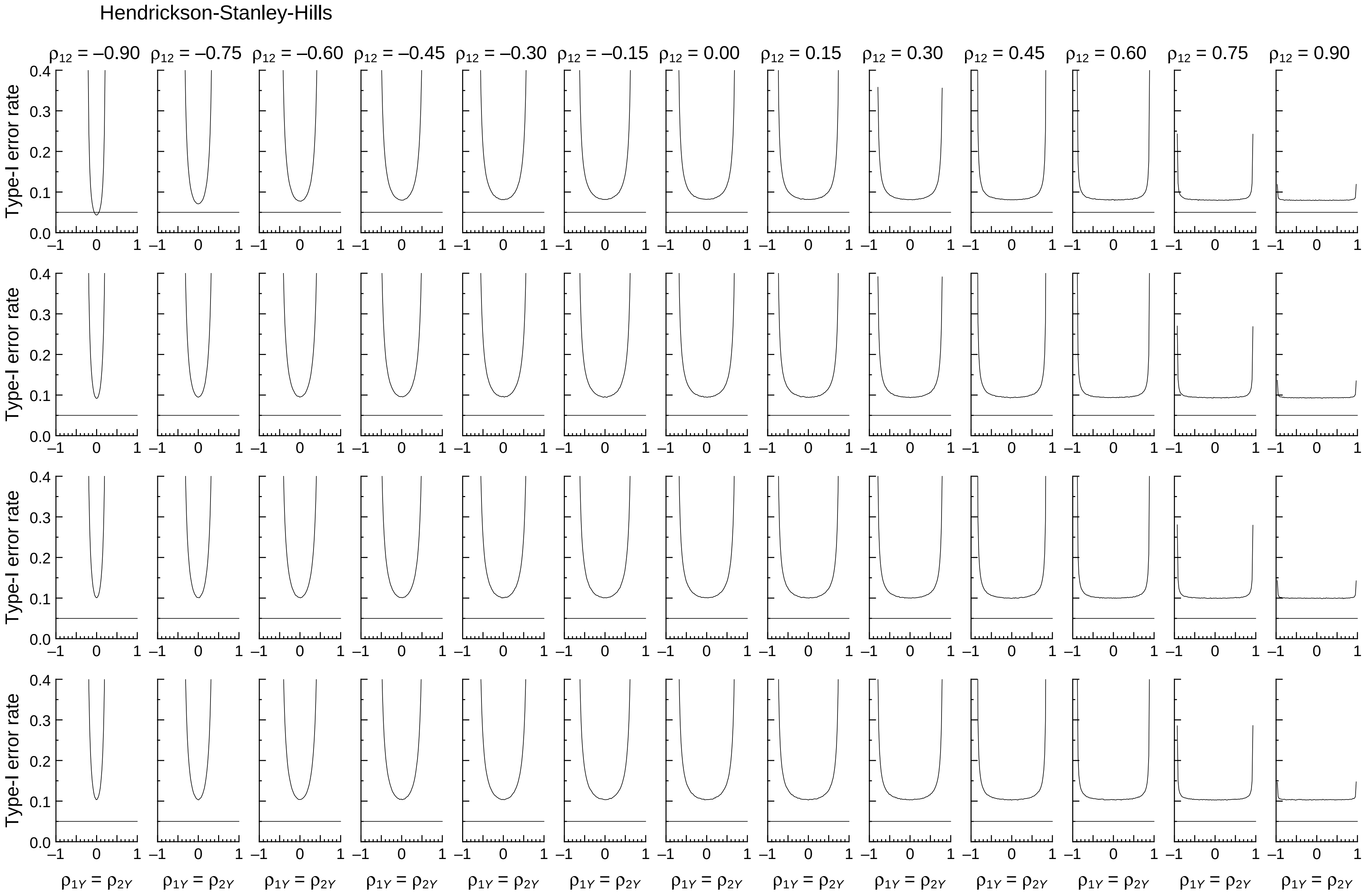

Section I: Type-I error rates of each test with mixture 0.9 N(0, 1) + 0.1 N(0, 4) data (sample size top to bottom: 20, 50, 100, 200)

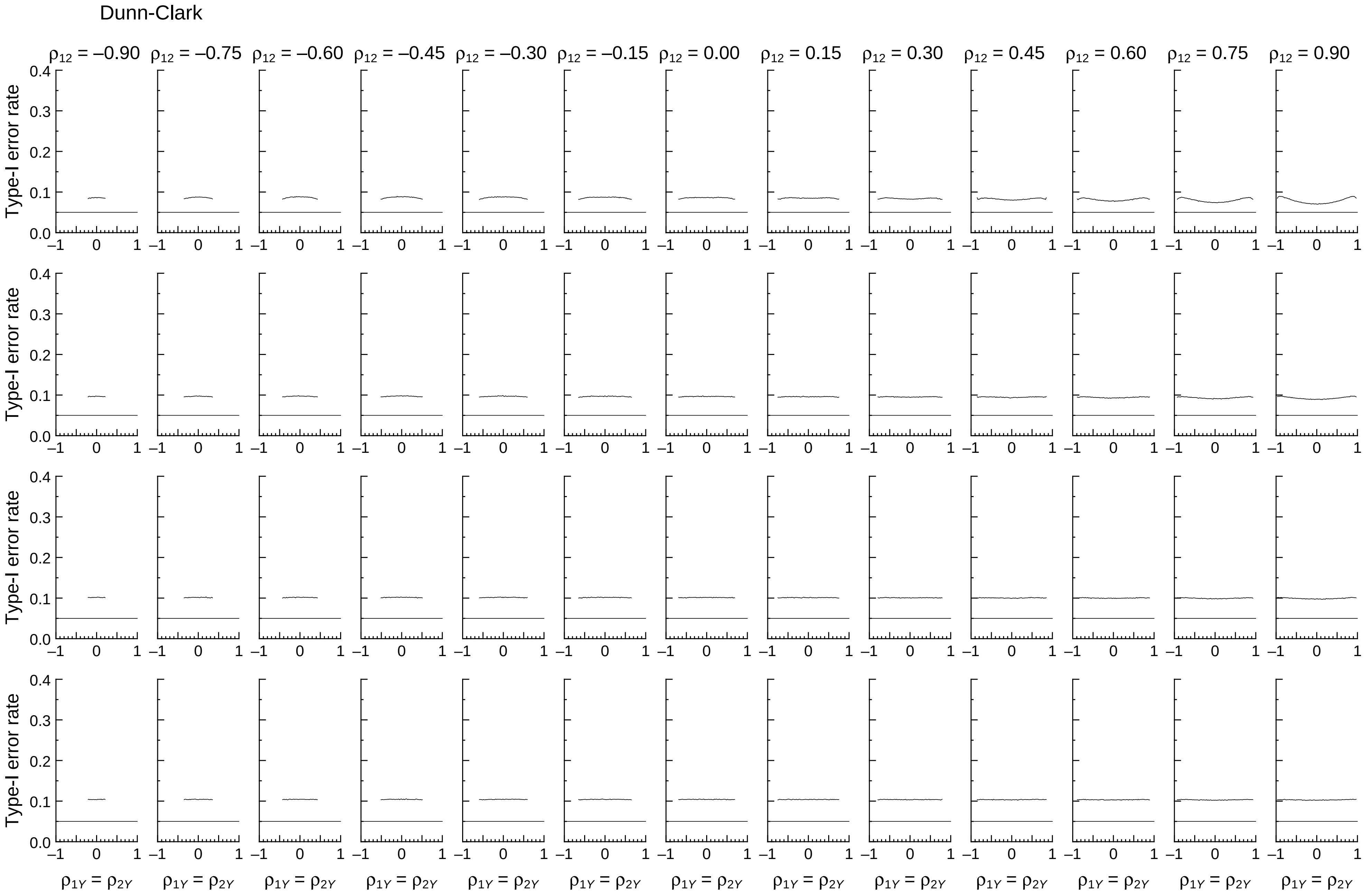

Section I: Type-I error rates of each test with mixture 0.9 N(0, 1) + 0.1 N(0, 4) data (sample size top to bottom: 20, 50, 100, 200)

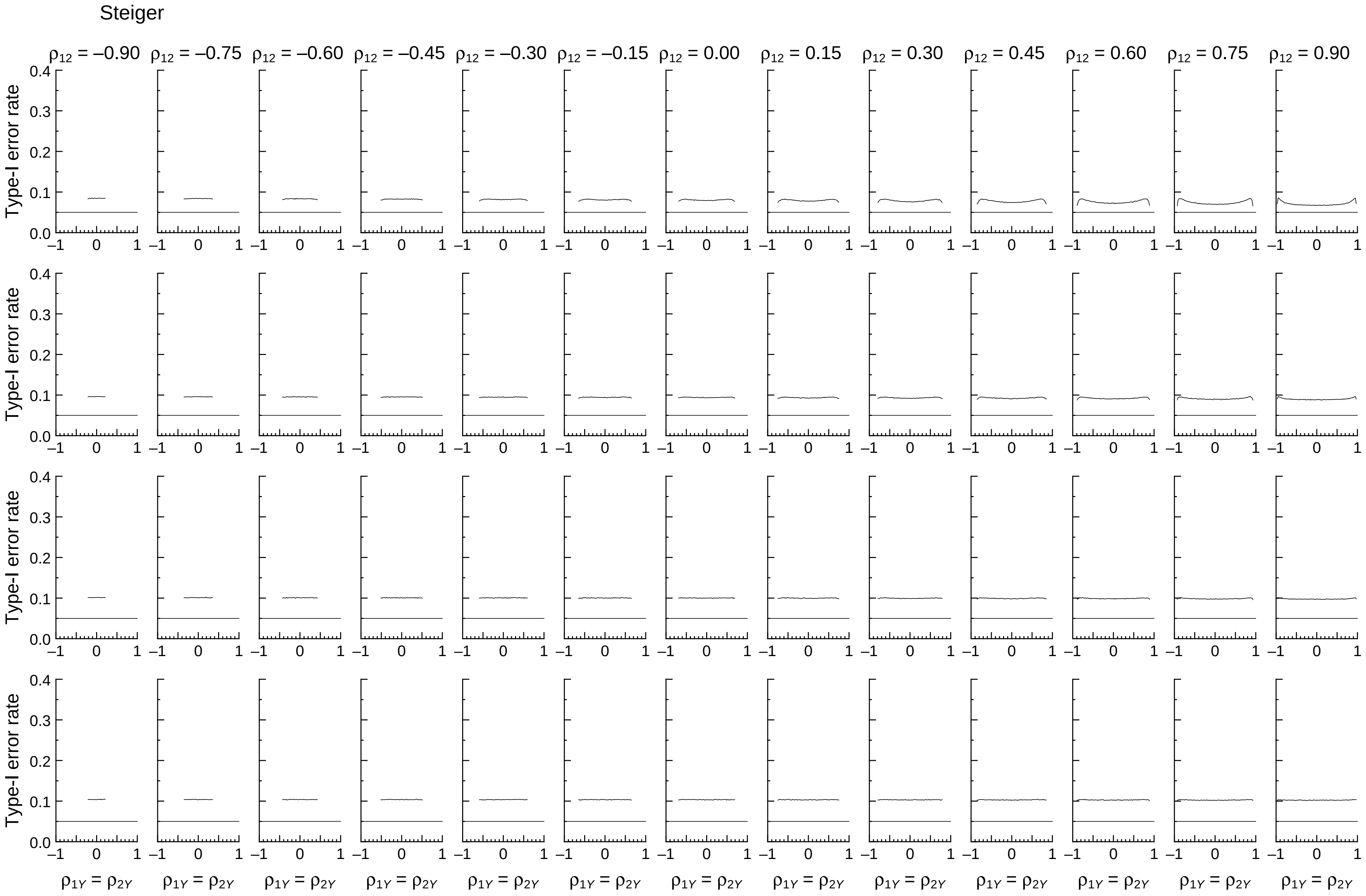

Section I: Type-I error rates of each test with mixture 0.9 N(0, 1) + 0.1 N(0, 4) data (sample size top to bottom: 20, 50, 100, 200)

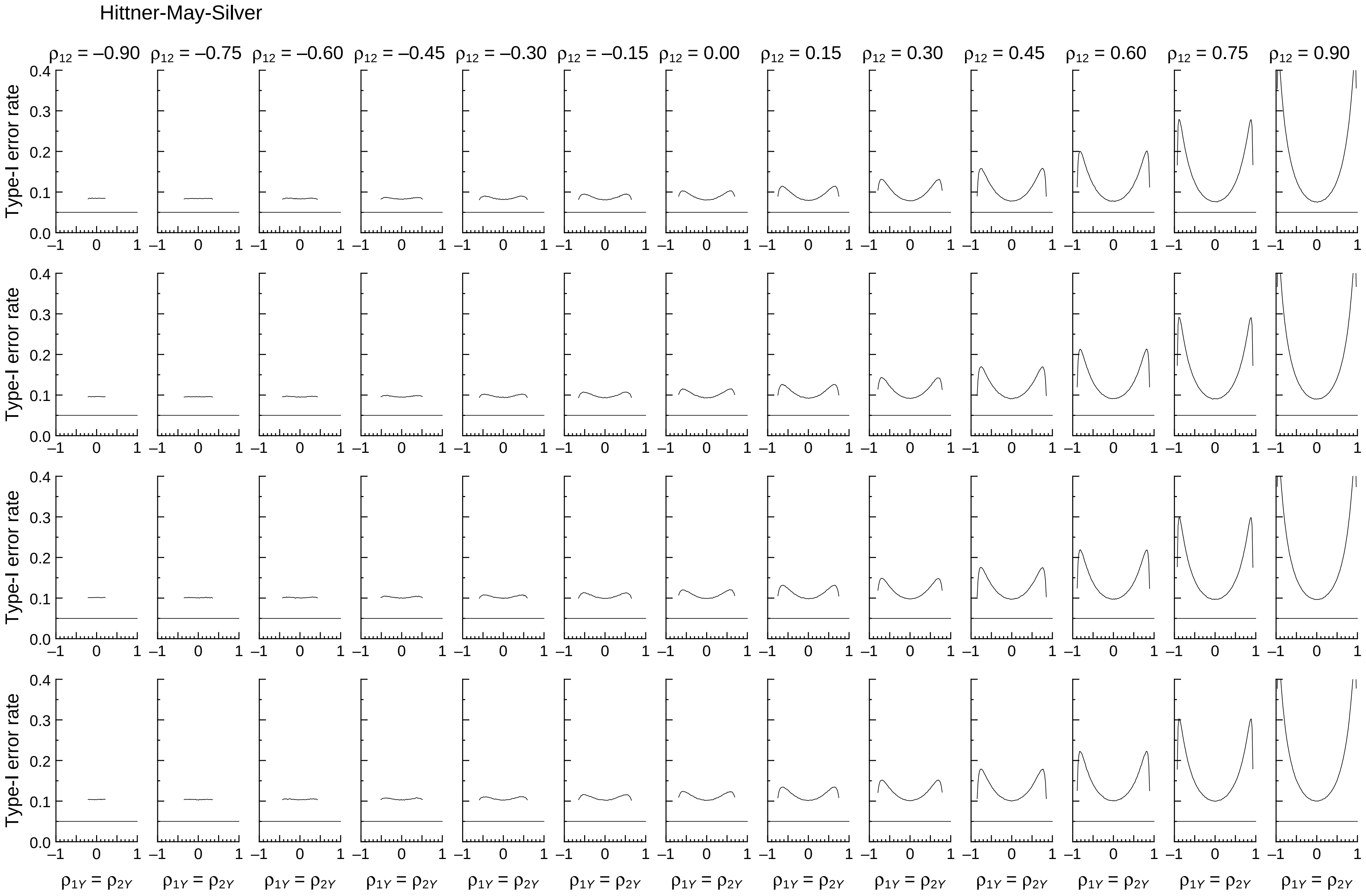

Section I: Type-I error rates of each test with mixture 0.9 N(0, 1) + 0.1 N(0, 4) data (sample size top to bottom: 20, 50, 100, 200)

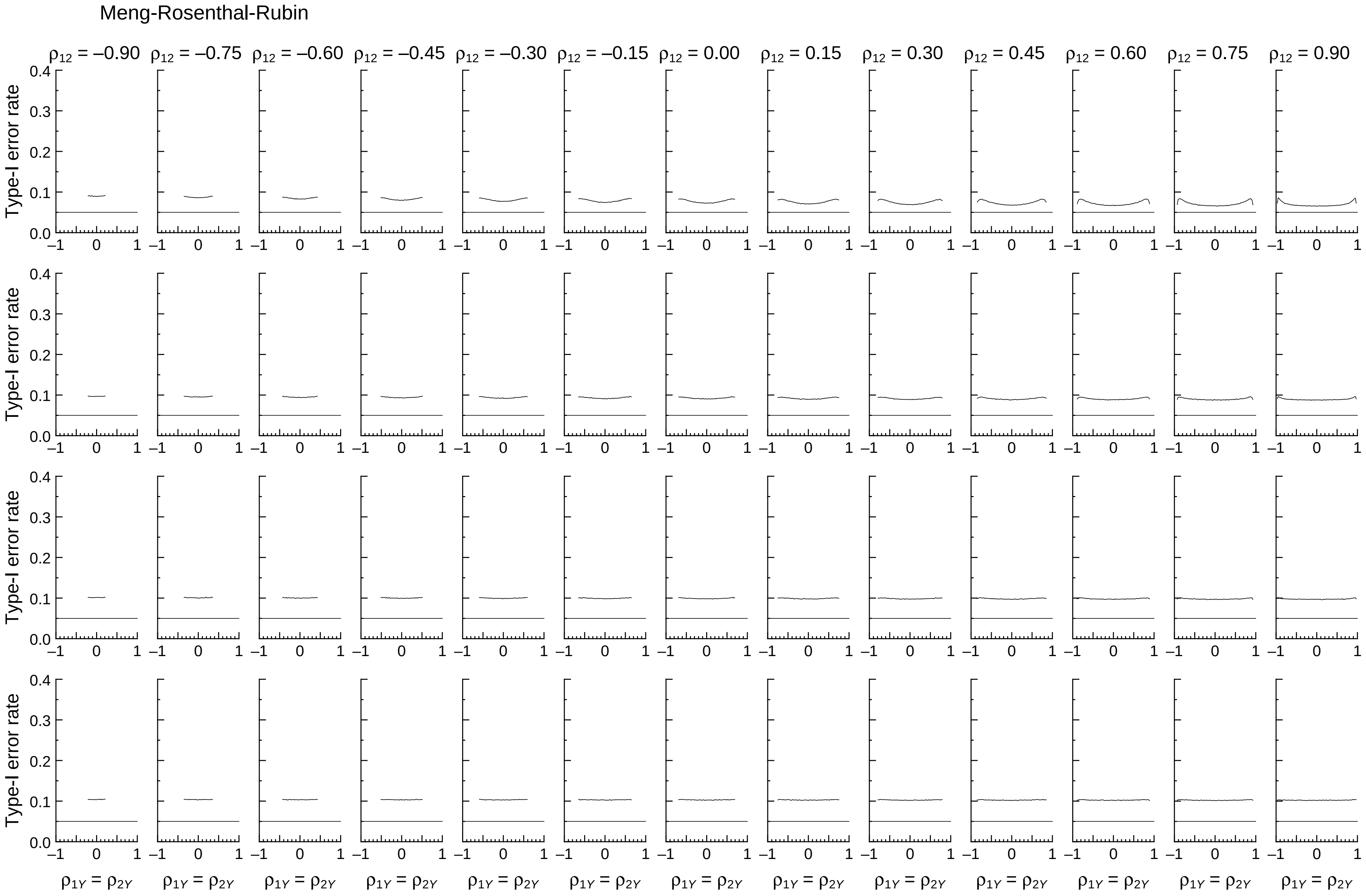

Section I: Type-I error rates of each test with mixture 0.9 N(0, 1) + 0.1 N(0, 4) data (sample size top to bottom: 20, 50, 100, 200)

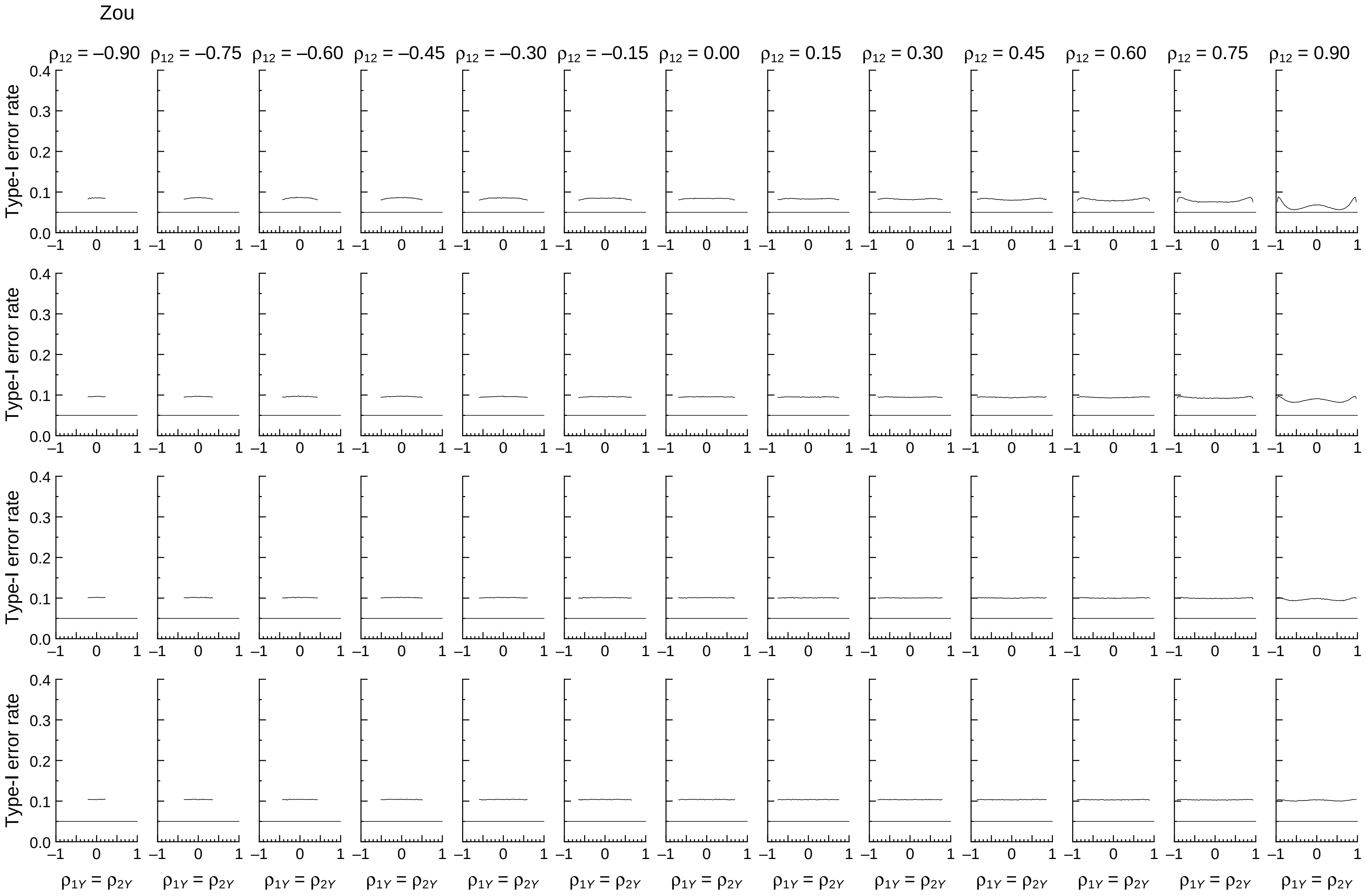

Section J: Type-I error rates of each test with mixture 0.9 N(0, 1) + 0.1 N(0, 10) data (sample size top to bottom: 20, 50, 100, 200)

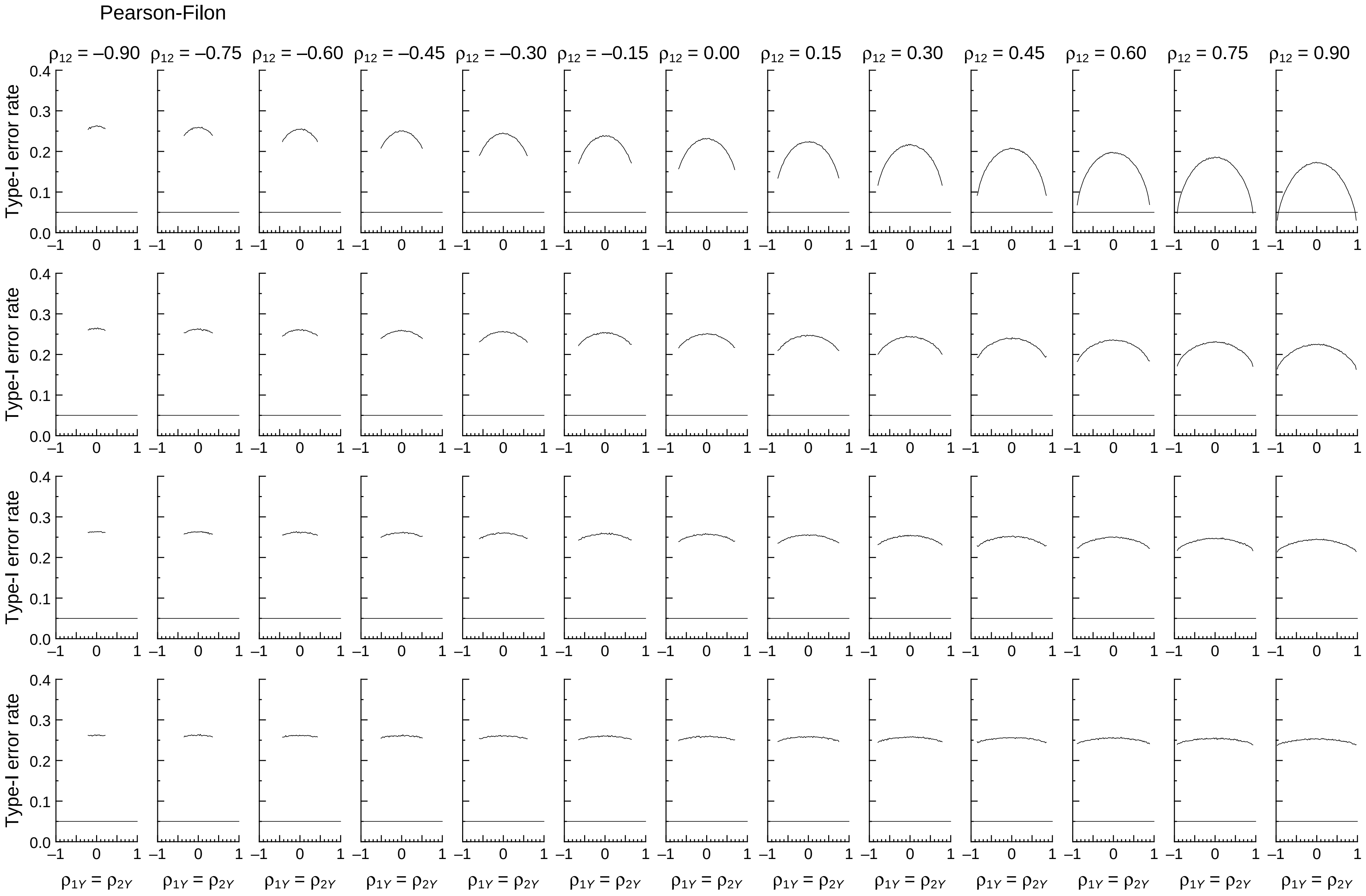

Section J: Type-I error rates of each test with mixture 0.9 N(0, 1) + 0.1 N(0, 10) data (sample size top to bottom: 20, 50, 100, 200)

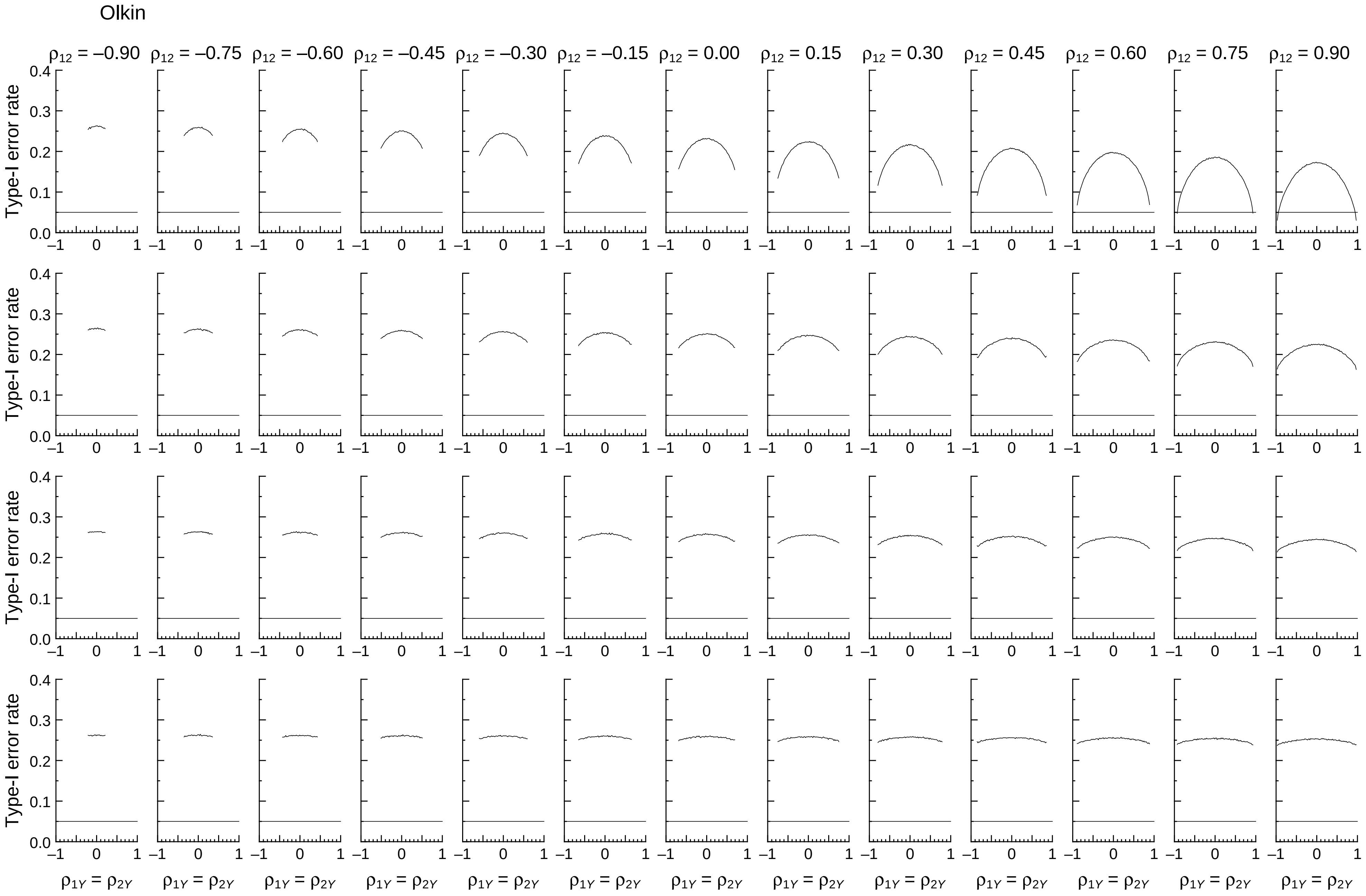

Section J: Type-I error rates of each test with mixture 0.9 N(0, 1) + 0.1 N(0, 10) data (sample size top to bottom: 20, 50, 100, 200)

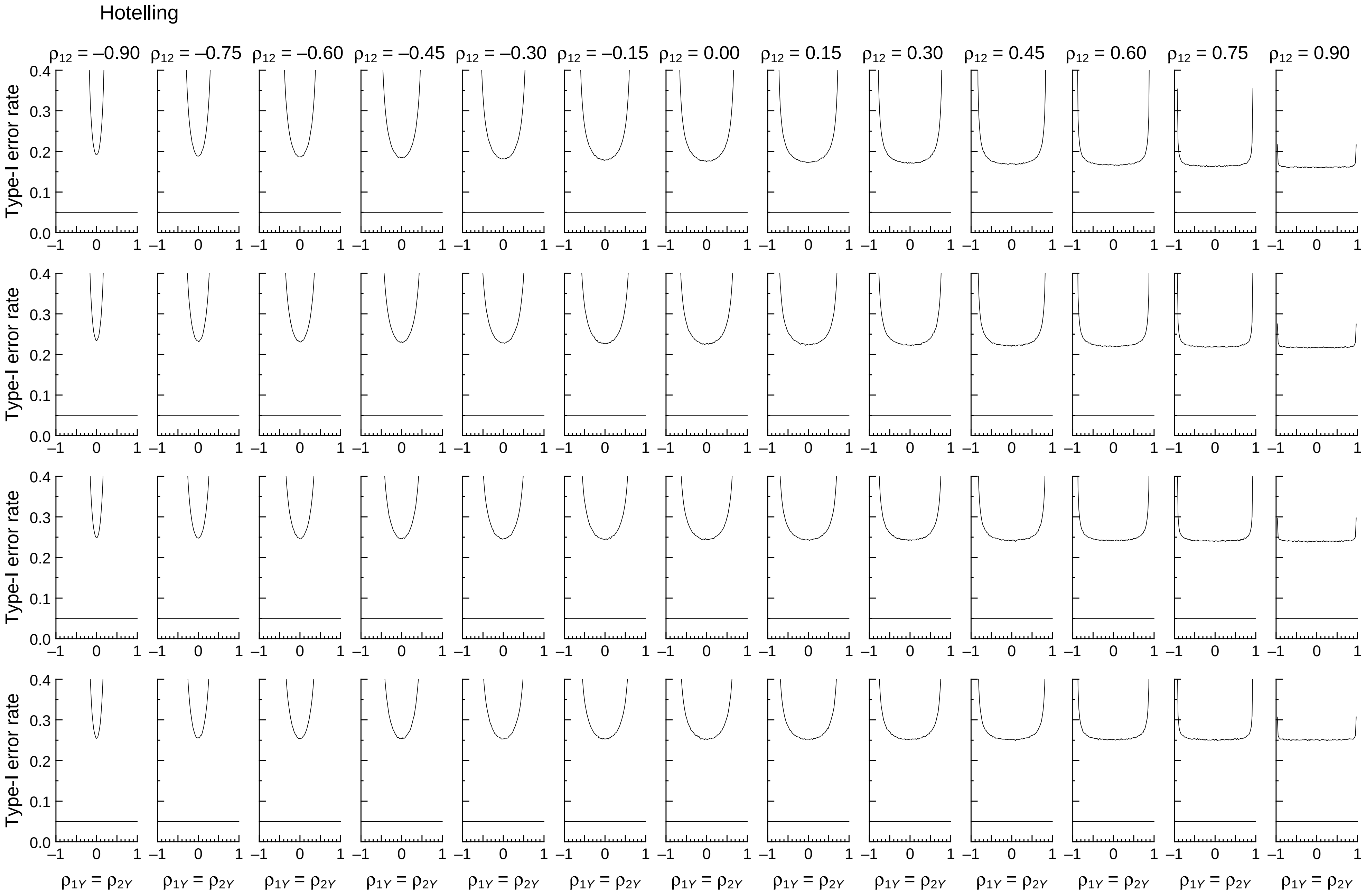

Section J: Type-I error rates of each test with mixture 0.9 N(0, 1) + 0.1 N(0, 10) data (sample size top to bottom: 20, 50, 100, 200)

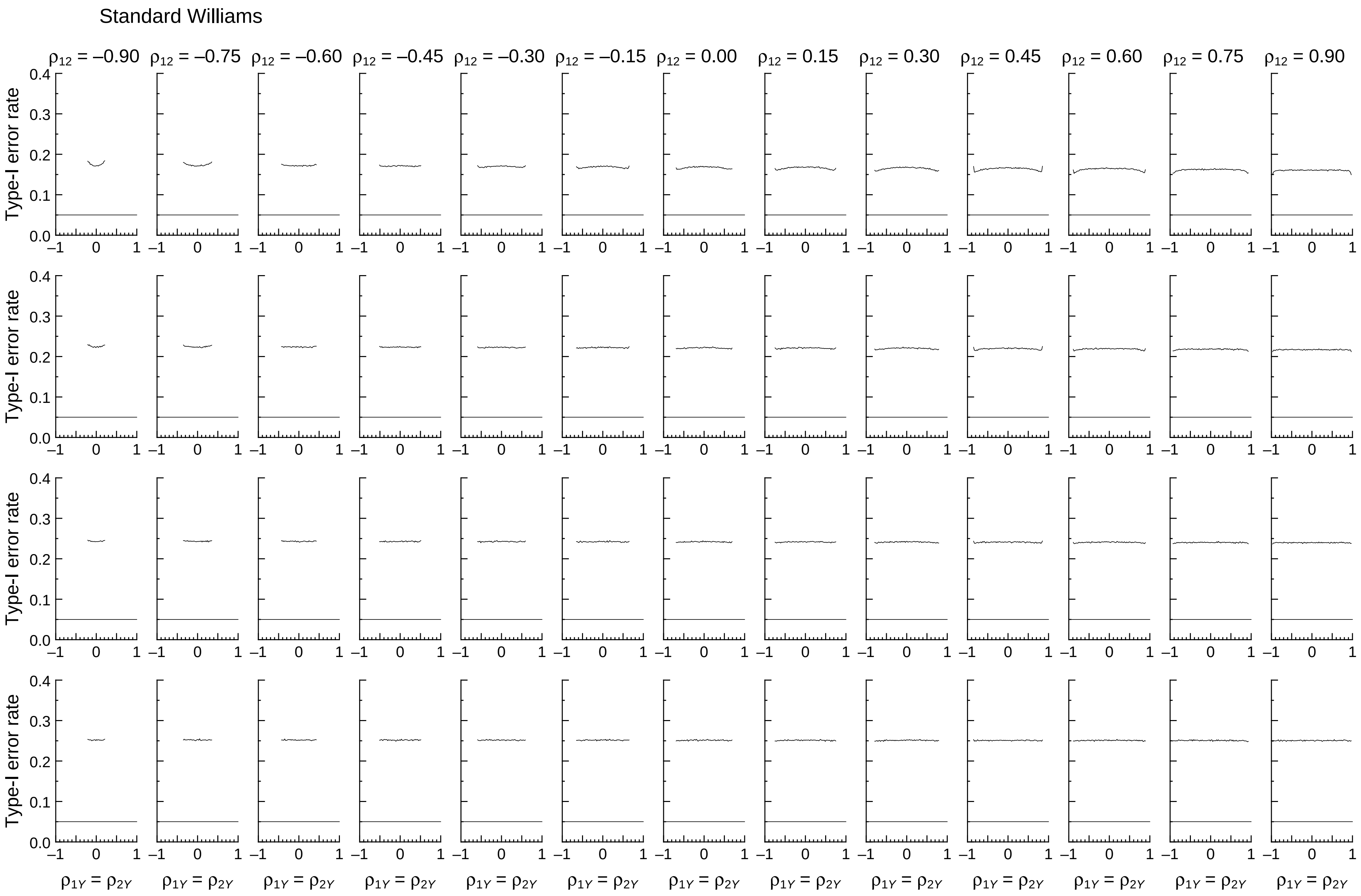

Section J: Type-I error rates of each test with mixture 0.9 N(0, 1) + 0.1 N(0, 10) data (sample size top to bottom: 20, 50, 100, 200)

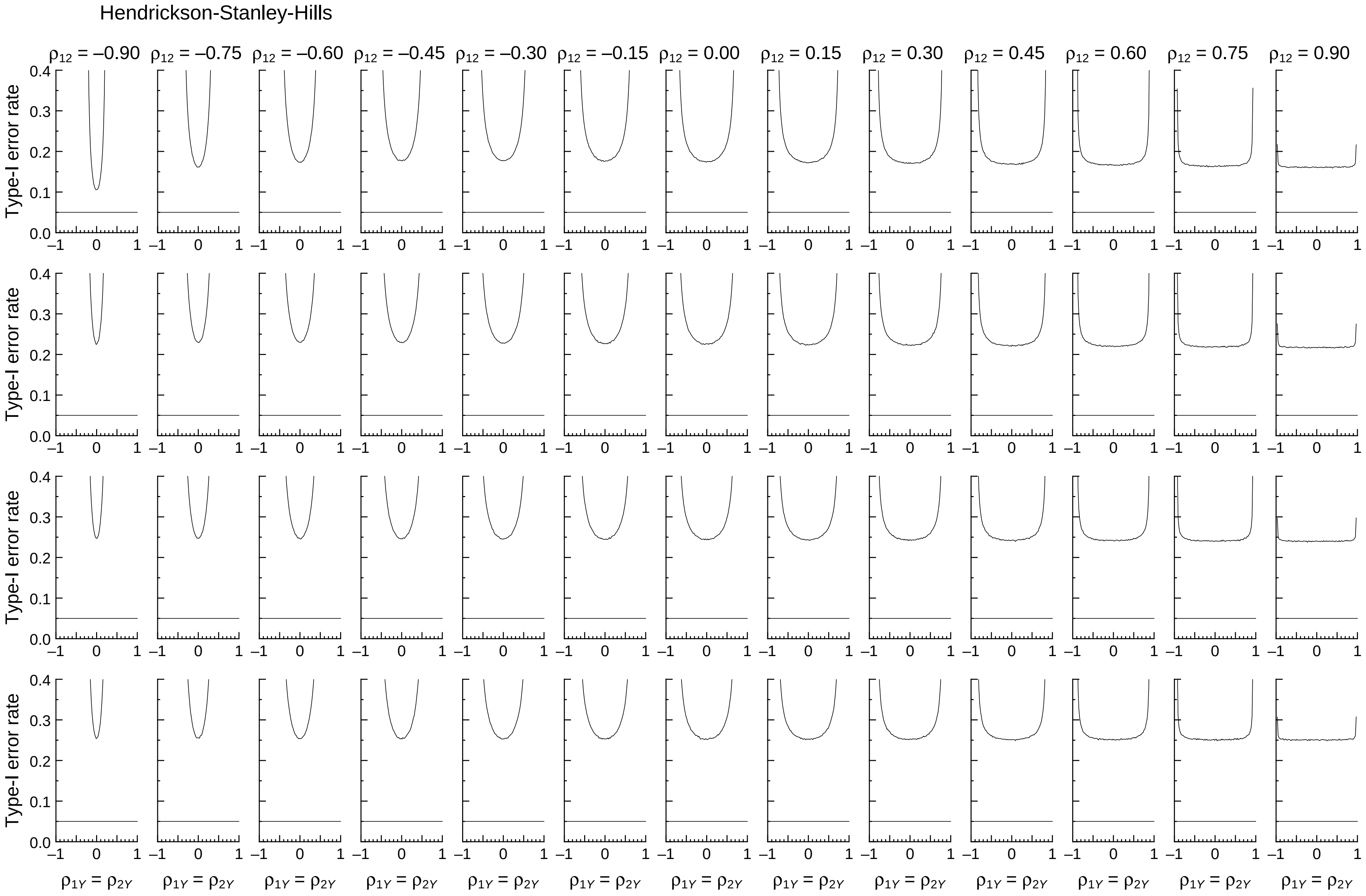

Section J: Type-I error rates of each test with mixture 0.9 N(0, 1) + 0.1 N(0, 10) data (sample size top to bottom: 20, 50, 100, 200)

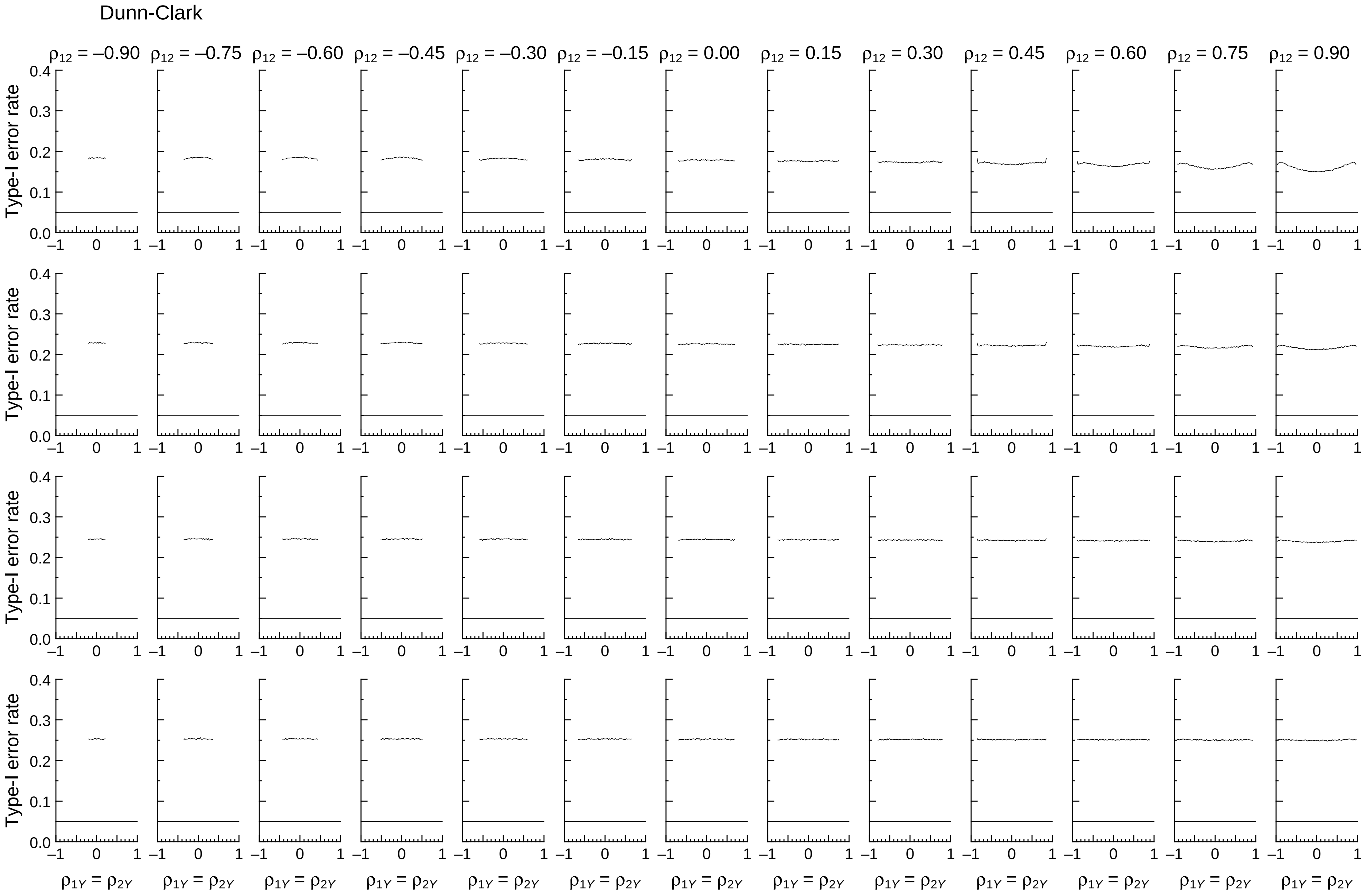

Section J: Type-I error rates of each test with mixture 0.9 N(0, 1) + 0.1 N(0, 10) data (sample size top to bottom: 20, 50, 100, 200)

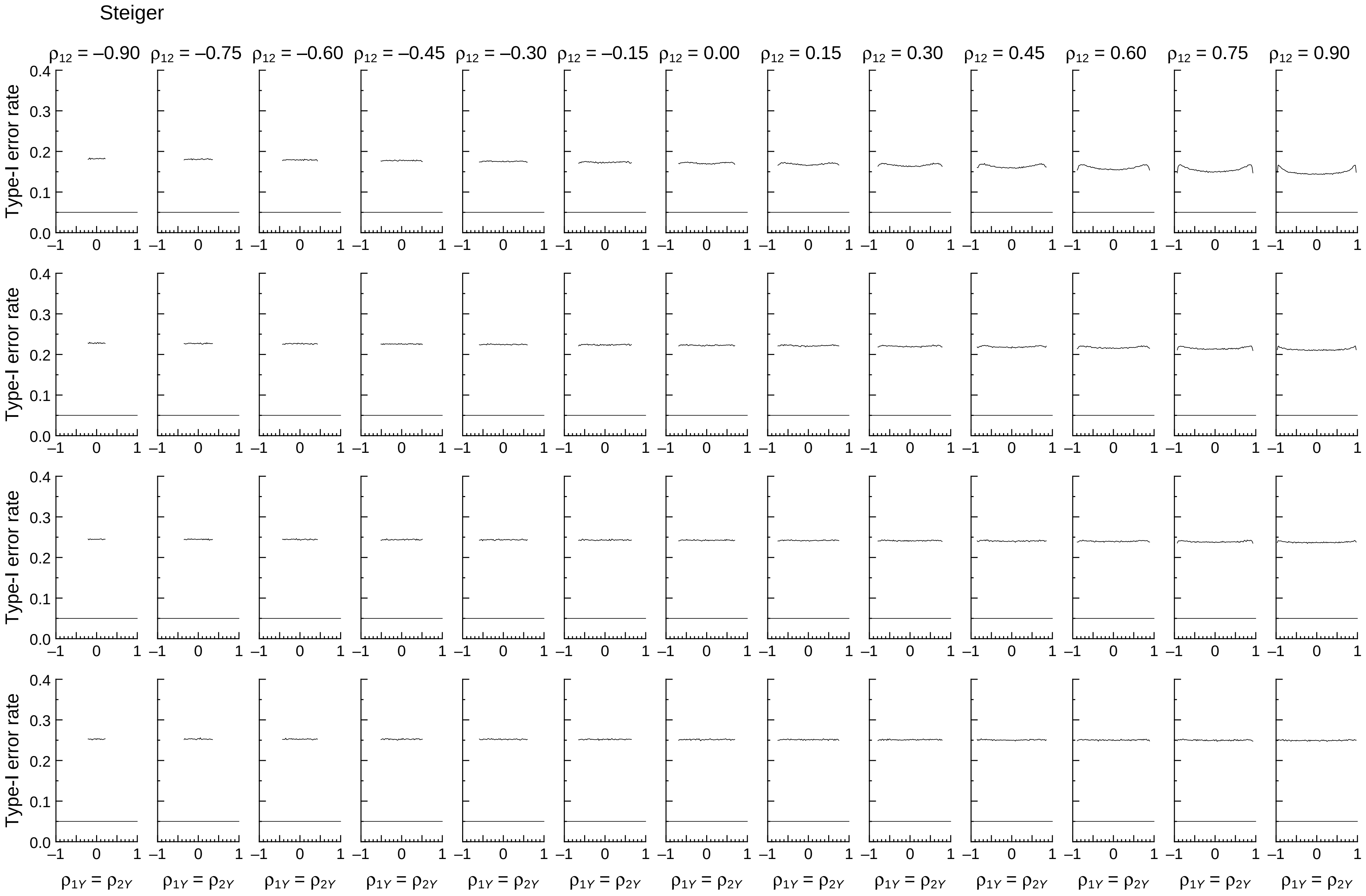

Section J: Type-I error rates of each test with mixture 0.9 N(0, 1) + 0.1 N(0, 10) data (sample size top to bottom: 20, 50, 100, 200)

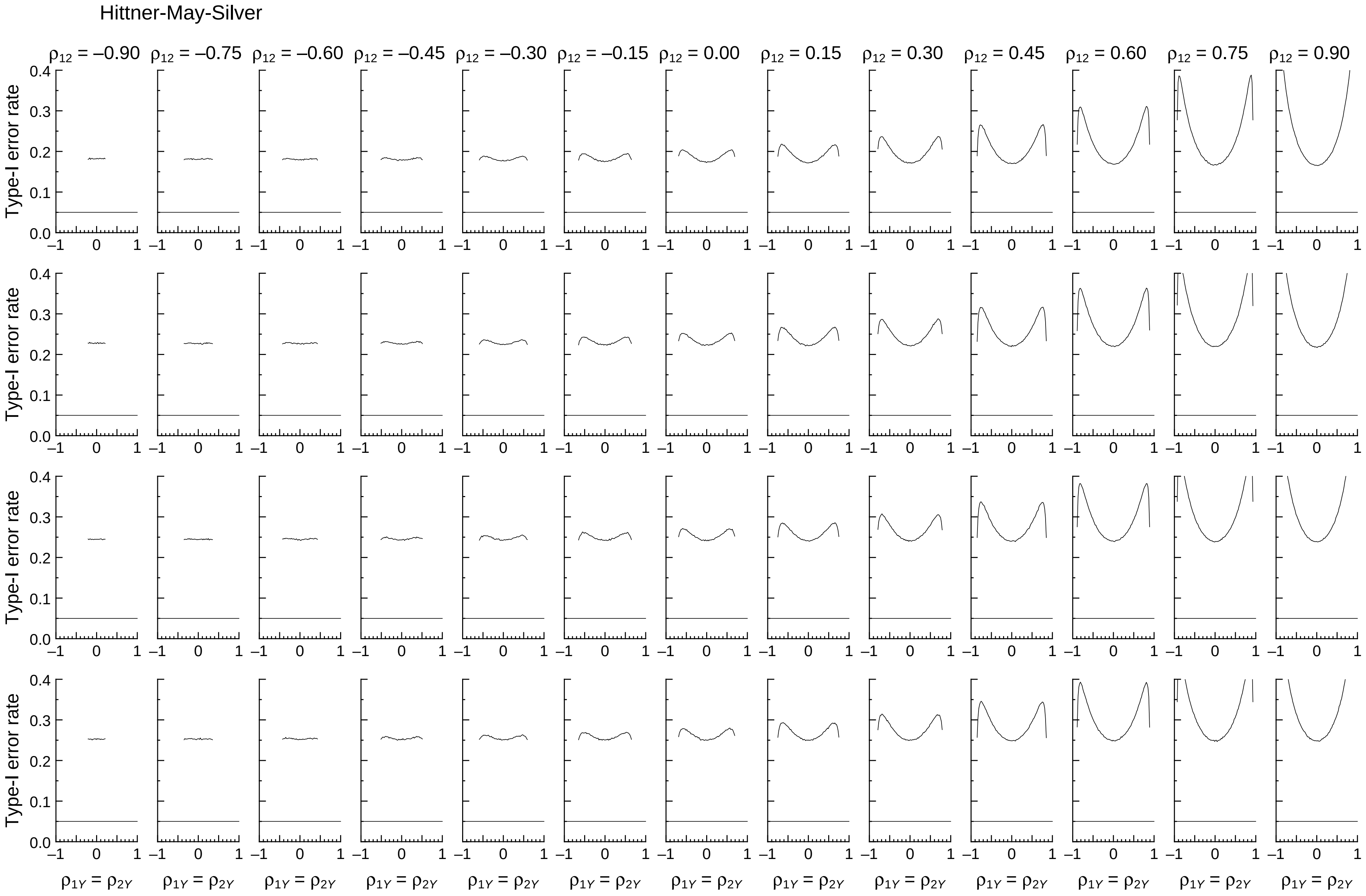

Section J: Type-I error rates of each test with mixture 0.9 N(0, 1) + 0.1 N(0, 10) data (sample size top to bottom: 20, 50, 100, 200)

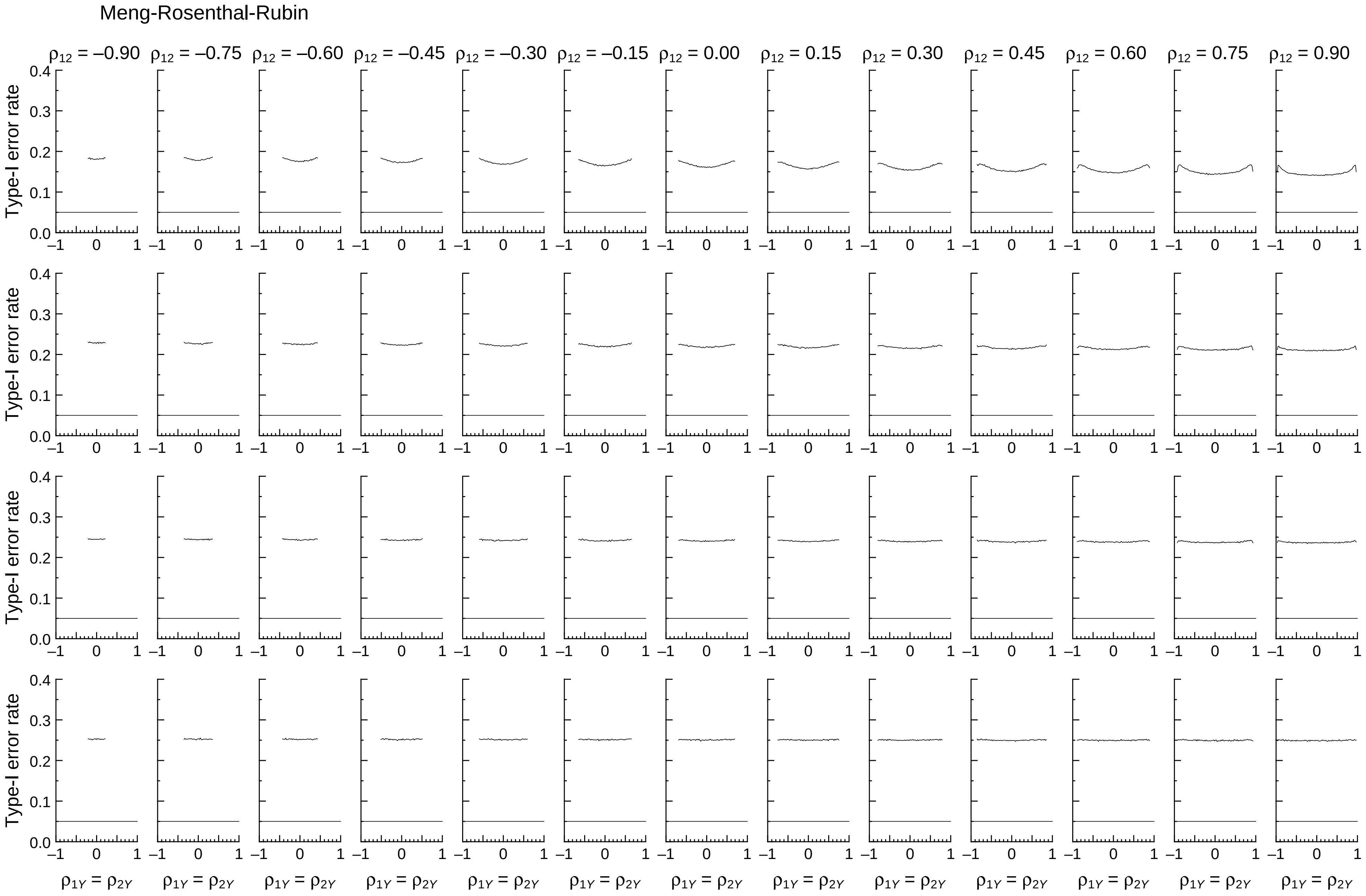

Section J: Type-I error rates of each test with mixture 0.9 N(0, 1) + 0.1 N(0, 10) data (sample size top to bottom: 20, 50, 100, 200)

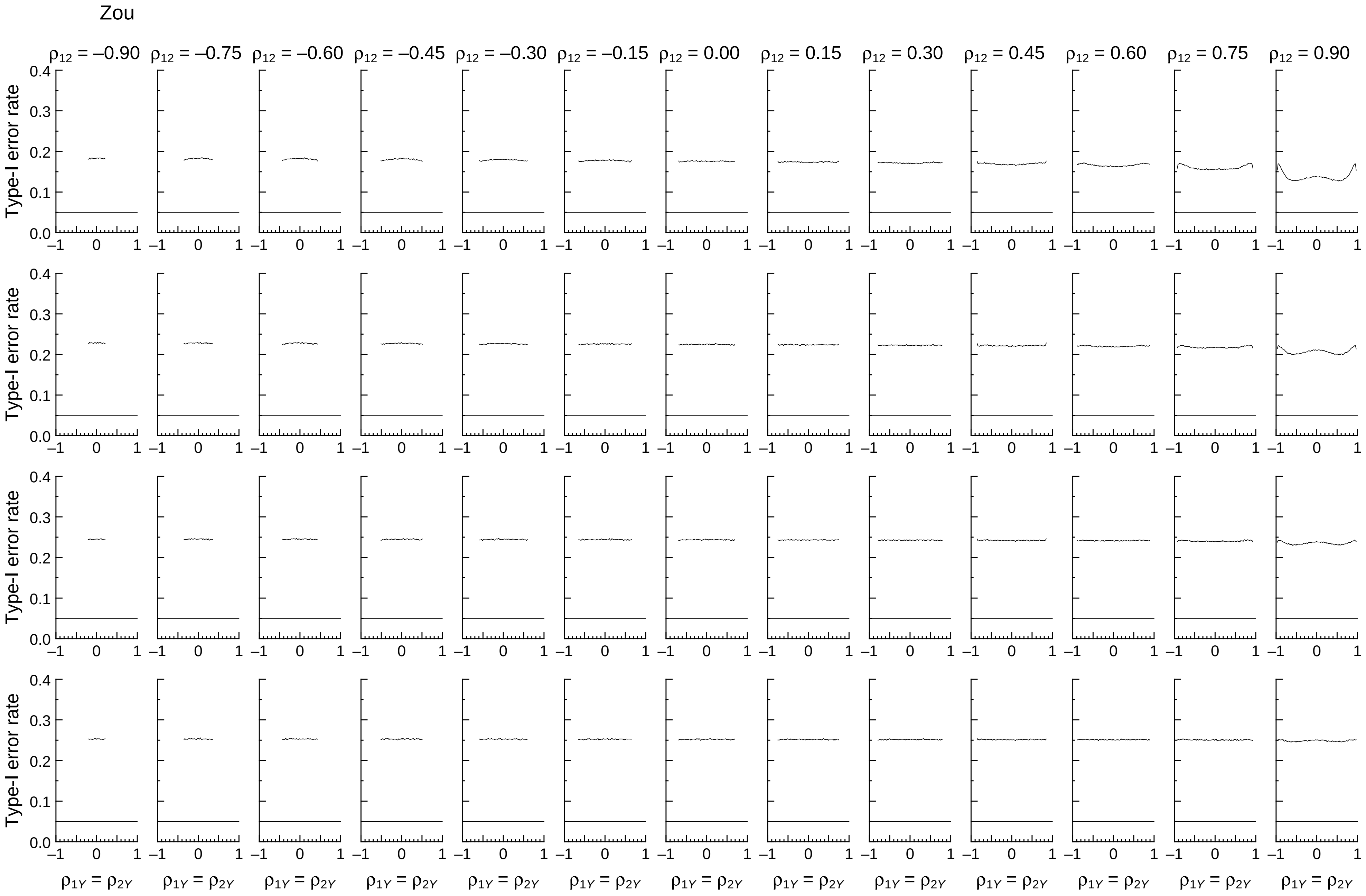

Supplement: Supplementary file 1 — Appendix S1. [file BMSP-78-112-s001.pdf]
